# A Chemo-Enzymatic Platform for Furanolide Synthesis and Functional Exploration

**DOI:** 10.1021/jacs.5c08354

**Published:** 2025-08-15

**Authors:** Xiaoqi Ji, Manuel Einsiedler, Paul M. D’Agostino, Jennifer Herrmann, Rolf Müller, Tobias A.M. Gulder

**Affiliations:** † Chair of Technical Biochemistry, 4356 Technische Universität Dresden, Bergstraße 66, 01069 Dresden , Germany; ‡ Department of Natural Product Biotechnology, Helmholtz Institute for Pharmaceutical Research Saarland (HIPS), PharmaScienceHub (PSH), Helmholtz Centre for Infection Research (HZI) and Department of Pharmacy at Saarland University, Campus E8.1, 66123 Saarbrücken, Germany; § Department of Microbial Natural Products, Helmholtz Institute for Pharmaceutical Research Saarland (HIPS), PharmaScienceHub (PSH), 443745 Helmholtz Centre for Infection Research (HZI) and Department of Pharmacy at Saarland University, Campus E8.1, 66123 Saarbrücken, Germany; ∥ German Centre for Infection Research (DZIF), Partner Site Hannover-Braunschweig, 38124 Braunschweig, Germany

## Abstract

Furanolides represent an emerging class of natural products known for their structural diversity and potent bioactivities, including antibiotic, cytotoxic, or algicidal effects. The systematic exploration of their biological activity profiles for the discovery of new hits for drug development thus constitutes a promising endeavor. However, their low natural abundance and the resulting difficulty in obtaining sufficient quantities have limited further in-depth investigations into their biological activity and structure–activity relationships (SARs). Building on our recent discovery of biosynthetic enzymes catalyzing furanolide core-structure assembly, we herein developed a cost-effective, one-pot enzymatic toolbox that enables the fast generation of hundreds of furanolide structural analogs. We systematically evaluated antimicrobial activities and cytotoxicity against the A549 lung cancer cell line for a representative selection of library congeners and explored their SARs. Several derivatives demonstrated significant cytotoxicity, particularly against lung cancer stem cells, offering promising insights into the development of furanolides as potential anticancer agents. Additionally, some analogs displayed promising antibacterial activity against important Gram-positive pathogens such as*Staphylococcus aureus*.

## Graphical abstract

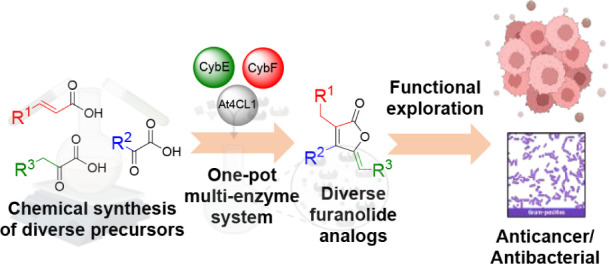

## Introduction

Furanolides represent a distinct class of natural products (NPs) characterized by a common trisubstituted γ-butyrolactone/furan-2­(5*H*)-one core structure. In all currently known natural representatives, R^1^ (α) and R^2^ (β) are either aromatic or aliphatic, and R^3^ (γ) is consistently aromatic (see common core structure; Figure 1). Furanolide NPs were discovered from phylogenetically diverse organisms, including freshwater cyanobacteria such as*Scytonema hofmanni*,^1^ marine myxobacteria such as*Plesiocystis pacifica*,^2^ and marine animals, e.g., the ascidian*Synoicum* sp.^3^ Furanolides display a broad spectrum of biological activities valuable for potential application in human medicine. For instance, enhygrolide A (**1**) has potent inhibitory activity against the Gram-positive bacterium*Arthrobacter crystallopoietes* with an MIC of 4 μg/mL.^4^ Nostoclide I (**2**) has demonstrated cytotoxicity (LC_50_ = 10 μg/mL) against Neuro-2a (CCL-131) and KB (CCL-17) cell lines,^5^ while cadiolide E (**3**) strongly inhibits the activity of*Candida albicans* isocitrate lyase (IC_50_ = 7.62 μm), highlighting its potential as an antifungal agent.^6^ In addition, the heavily functionalized cyanobacterin (**4**) is a strong inhibitor of photosynthesis, a biological activity relevant for development of agricultural agents.^7^

**Figure 1:**
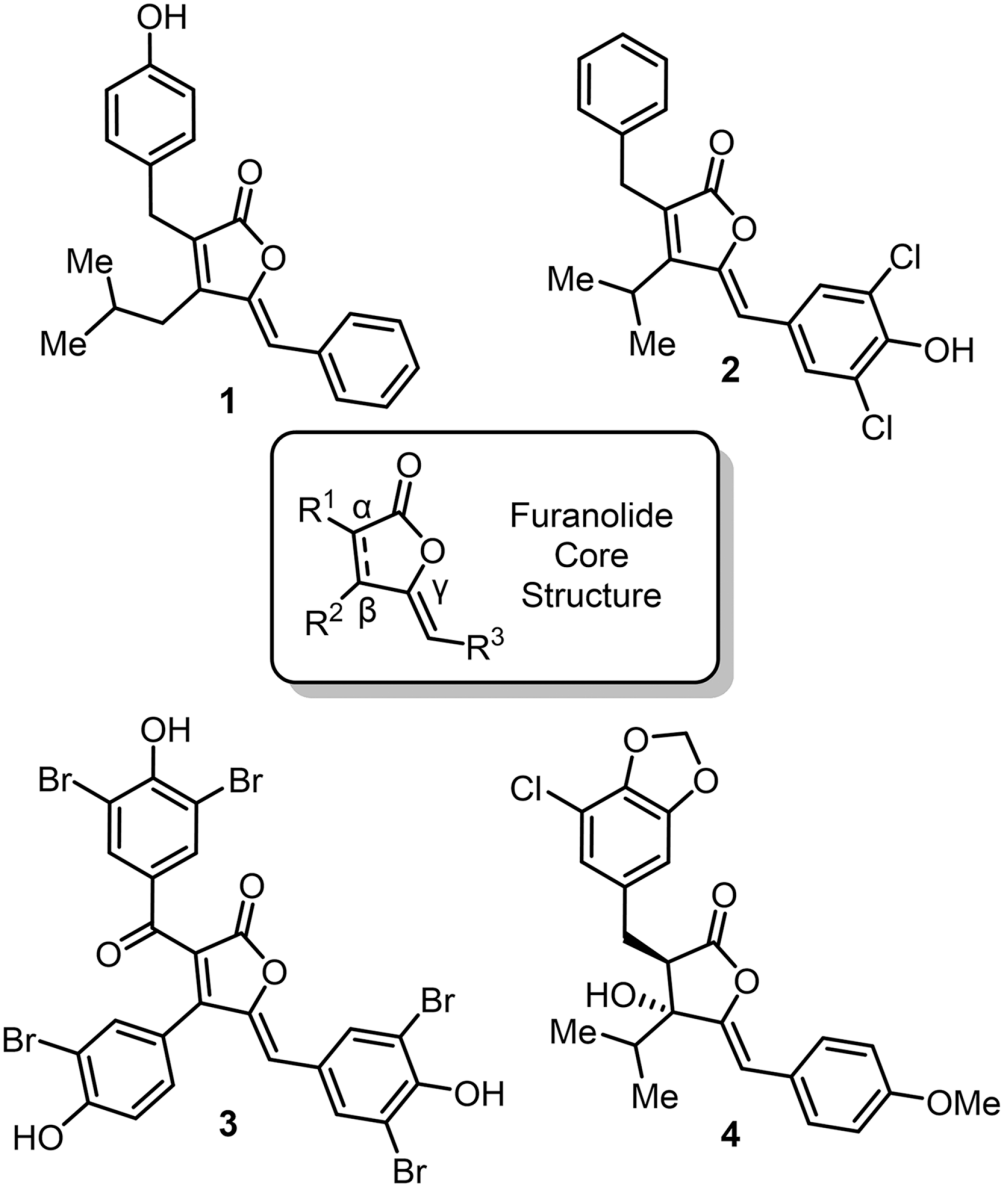
Furanolide core structure and representative examples of furanolide NPs: enhygrolide A (**1**), nostoclide I (**2**), cadiolide E (**3**), and cyanobacterin (**4**).

Despite these promising bioactivities reported across structurally diverse furanolide NPs, however, no systematic evaluation of their activity profiles has so far been conducted. Such investigations are primarily hampered by the relatively small number of furanolide structural analogs produced in sufficient amounts by a given natural producer and their tedious isolation from complex fermentation extracts. While chemical synthesis could help solve this problem in principle, current total synthetic approaches have not yet fulfilled this promise. This is a consequence of the often strictly linear synthetic routes that make structural variations of the furanolide substituents laborious. In addition, syntheses often rely on expensive transformations with low overall atom economy, even for the structurally least challenging furanolides. For enhygrolide A (**1**), for example, two total synthetic approaches currently exist, both of which requiring five linear steps. The first approach published by Muddala et al. (Figure 2A)^8^ utilizes tetronic acid **5** as the starting material, which is initially condensed with 4-methoxybenzaldehyde using an organocatalytic approach with l-proline and Hantzsch ester as hydride source, installing the R^1^ residue to give **6**. Acylation of **6** with pivaloyl chloride sets the stage for installation of the R^2^ substituent by iron-catalyzed addition of ^*i*^BuMgBr to yield **7**. Aldol condensation of **7** with benzaldehyde followed by BBr_3_-induced *O*-demethylation delivers **1** in 54% overall yield. The alternative approach by Sghairi et al. starts with alkyne **8** containing the later R^2^ residue.^9^ Terminal deprotonation of **8** using EtMgBr followed by addition to CO_2_ and dibromination of the triple bond delivers alkene **9**, which gets fused to phenylacetylene in a domino Sonogashira/oxacyclization sequence to give **10**. Installation of the R^1^ substituent is achieved by Suzuki coupling to *p*-methoxy benzyltrifluoroborate, followed by *O*-demethylation using BBr_3_. While this approach was also used to install slight structural variations (R^2^ = Me; R^1^ = benzyl, *m*-hydroxybenzyl), overall yields are even lower, e.g., 17% for **1**.

**Figure 2:**
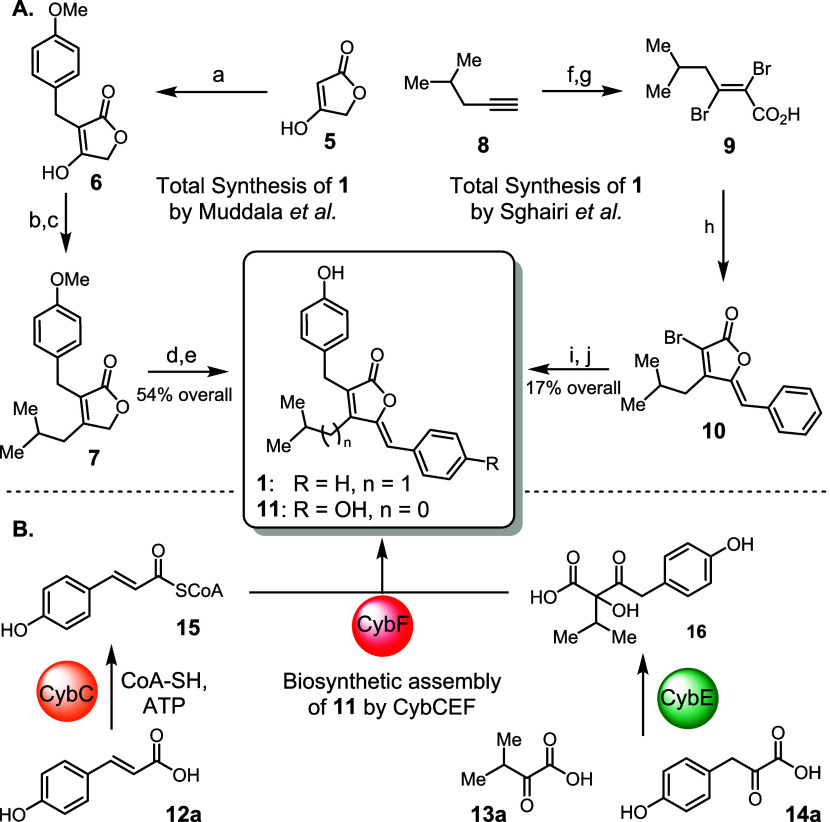
**A.** Total syntheses of enhygrolide A (**1**) compared to biosynthesis of precyanobacterin (**11**). Top left: synthesis by Muddala et al.^8^ Top right: synthesis by Sghairi et al.^9^ Conditions: **a.** 4-methoxybenzaldehyde (3 equiv), Hantzsch ester (1 equiv), l-proline (5 mol %), DCM, rt, *83%*; **b.** Tf_2_O (1.3 equiv), *92%* or PivCl (1.05 equiv), *86%*; **c** **.** ^*i*^BuMgBr, 10 mol % Fe­(acac)_3_, *N*-methylpyrrolidone/tetrahydrofuran (THF), – 40 °C, 1 h, *69%* or ^*i*^BuMgBr (2.4 equiv), 2 mol % FeCl_2_, THF, 0 °C, 1 h, *93%*; **d.** Na_2_CO_3_ (1 equiv), PhCHO (1.5 equiv), MeOH, 50 °C, 24 h, *82%*; **e.** BBr_3_ (3 equiv), *100%*; **f.** EtMgBr in THF (1 m, 1 equiv), THF, – 10 °C to reflux, 1 h then CO_2_ (gas), −10 °C, *85%*; **g.** Br_2_ (2 equiv), MeOH, 10 °C, 40 min, *70%*; **h.** phenylacetylene (2 equiv), CuI (1 equiv), ^*i*^Pr_2_NH, DMF, 80 °C, overnight, *66%*; **i.** *p*-methoxy benzyltrifluoroborate (3 equiv), [(dppf)­PdCl_2_] (15 mol %), Cs_2_CO_3_ (3 equiv), PhMe/water, 80 °C, overnight, *44%*; **j.** BBr_3_ (3 equiv), DCM, 0 °C, 2 h, *100%*. **B.** Biosynthesis of **11** from **12a−14a** by the three enzymes CybCEF.^10^

In contrast, the biosynthetic assembly of furanolides is very straightforward, although mechanistically complex. Previous studies in our laboratory on the biosynthesis of precyanobacterin (**11**; Figure 2B), an important member of the furanolide family, have provided in-depth insights into this process.^10^ The enzymatic machinery efficiently fuses the three biosynthetic building blocks 4-coumaric acid (**12a**, in turn derived of l-tyrosine by action of the pathway-specific ammonia lyase CybB), ketoisovaleric acid (**13a**), and 4-hydroxyphenylpyruvic acid (**14a**). First, the long-chain acyl-CoA synthetase CybC catalyzes the conversion of coumaric acid (**12a**) to coumaroyl-CoA (**15**), a process that can also be achieved using the homologous enzyme At4CL1 from*Arabidopsis thaliana*.^11^ The thiamine pyrophosphate (TPP)-dependent enzyme CybE then catalyzes the formation of carboxylic acid **16** by acyloin condensation of **13a** and **14a**, establishing the *C*,*C*-bond between the later β- and γ-positions of the furanolide core. Finally, the furanolide synthase CybF facilitates a reaction cascade involving an *O*-acylation, a formal Morita−Baylis−Hillman (MBH) reaction, and a final 1,4-hydride shift using **15** and **16** to build the *C*,*C*-bond between the later α- and β-positions and ultimately form **11**. This highly efficient biosynthetic route to **11**, involving only three biocatalysts, seemed attractive for the development of enzymatic syntheses toward structurally diverse furanolides.

Within this study, we systematically screened the substrate plasticity of CybE and CybF by variations of the building blocks **12**−**14**, leading to the development of a streamlined one-pot procedure for biocatalytic furanolide assembly. Evaluation of the bioactivity of a subset of the enzymatically synthesized furanolide derivatives identified potent effects on cancer stem cells and Gram-positive bacterial pathogens, highlighting the potential of our approach for hit discovery and thus biomedical applications.

## Results and Discussion

### Preparation of Natural Substrate Analogs

NPs often require further derivatization to enhance their biological activity,^12^ and even small structural modifications can lead to significant differences in bioactivity.^13,14^ The biosynthesis of **11** from three simple precursors offers the possibility to develop a modular chemo-enzymatic toolbox for generating focused furanolide compound libraries. Taking into account but also going beyond typical natural substitution patterns at the R^1^−R^3^ residues of the furanolide core structure (cf. Figure 1), we selected 12 different coumaric acid analogs **12a**–**l** translating into different R^1^ substitution, 11 building blocks **13a**–**k** for variations at R^2^, and eight substrate analogs **14a**–**h** targeting R^3^. While most of these substrates are commercially available, some of them had to be prepared synthetically to cover the desired structural space. For substrate analogs **12**, compounds **12h**, **12i**, and **12l** were synthesized. The synthesis of **12i** was realized by condensation of 5-chlorovanillin (**17**, Figure 3B) with commercially available ylide **18** (82%) and subsequent saponification in 97% yield. Subsequent *O*-demethylation of **12i** using BBr_3_ afforded **12h** in 61% yield. For the synthesis of **12l**, dibromination of 4-hydroxybenzaldehyde (**19**, Figure 3B) with *N*-bromosuccinimide (NBS) gave 3,5-dibromo-4-hydroxybenzaldehyde (**20**) in 91% yield. Condensation of **20** with **21** (Figure 3B) produced **12l** in 92% yield. For substrate analogs **13**, only **13k** required synthesis. Starting from l-4-hydroxyphenylglycine methyl ester (**22**, Figure 3C), **13k** was synthesized through a three-step process involving transamination, bromination, and saponification (43% overall yield).^15^ For the desired analogs **14**, **14c–g** needed to be prepared, initially following a general procedure.^16^ The corresponding amino acid precursors **24c**,**e**−**g** (Figure 3D) were reacted with trifluoroacetic anhydride (TFAA) to form trifluoromethyloxazolones **25c**,**e**−**g**, which were hydrolyzed with aqueous trifluoroacetic acid (TFA) to yield the desired substrates **14c**,**e**−**g** in 1% to 35% yield. Since particularly the yield of **14c** was unsatisfactory (1%), we adopted an alternative synthetic route for **14c** and **14d**.^17^ Using the Erlenmeyer synthesis, the corresponding aldehydes **26c**,**d** (Figure 3D), *N*-acetylglycine (**27**), and sodium acetate were reacted in acetic anhydride at elevated temperature to form oxazolones **28c**,**d**. Treatment of **28c**,**d** with an aqueous HCl solution provided **14c** and **14d** in yields of 73% and 52%, respectively. In total, 31 different substrates were finally available (Figure 3A), laying the foundation for the subsequent systematic study of CybE and CybF substrate plasticity in vitro.

**Figure 3:**
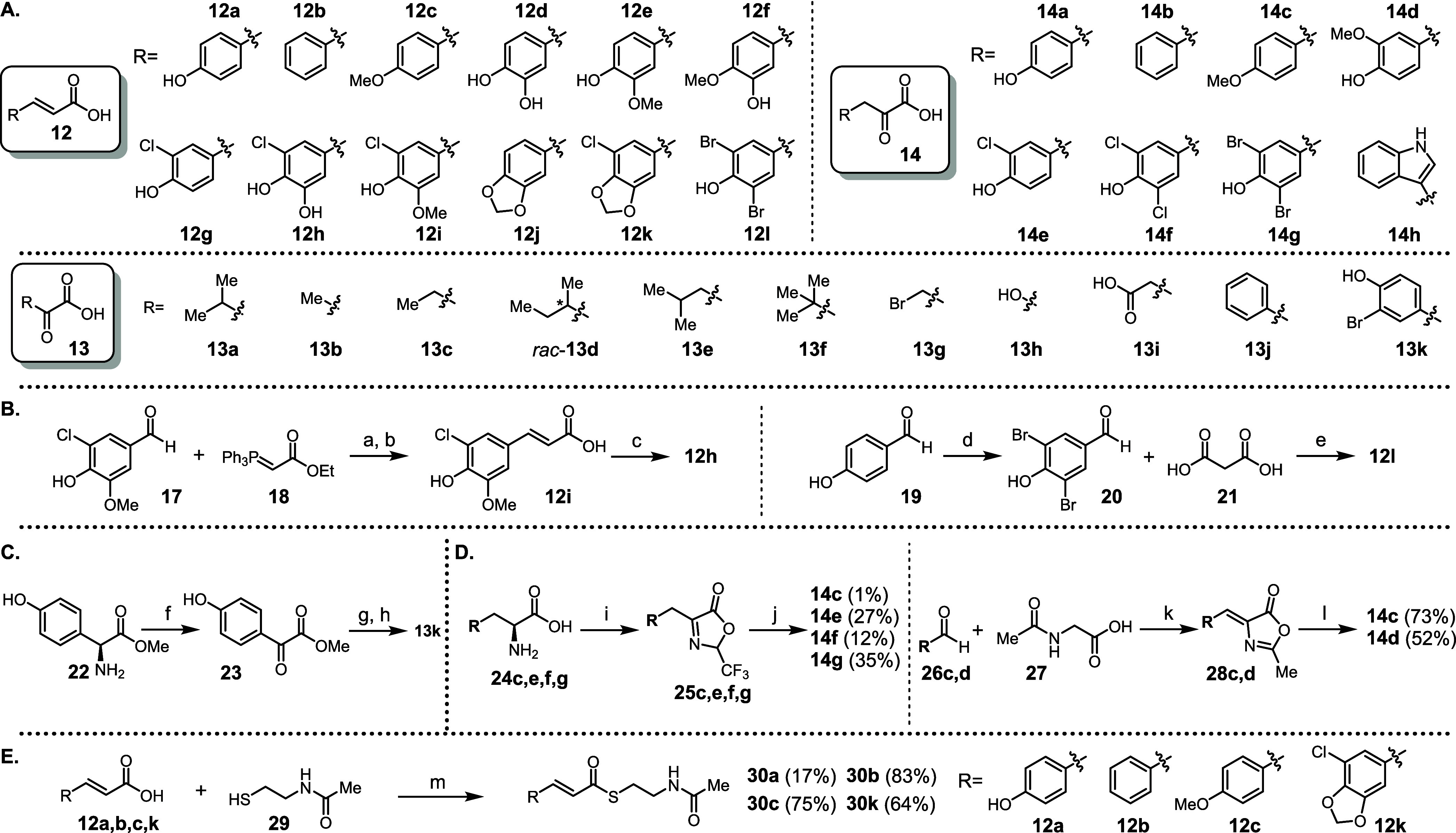
Structures and synthesis of natural substrate analogs. **A.** Structures of synthetic substrate analogs **12–14**. **B.** Synthesis of substrates **12h,i,l**. **C.** Synthesis of substrate analog **13k**. **D.** Synthesis of substrate analogs **14c**–**g**. **E.** Synthesis of acyl-SNACs **30a,b,c,k**. Conditions: **a.** (DCM) r.t., 1 d, *82%*; **b.** LiOH, KOH, (THF/H_2_O) r.t., 6 d, *97%*; **c.** BBr_3_, (DCM) 0 °C to r.t., 1 d, *61%*; **d.** NBS, (DCM/THF) 0 °C to r.t., 2 h, *91%*; **e.** C_5_H_11_N, (Py) 90 °C, 1 h; **f.** Glyoxylic acid, CuSO_4_ × 5 H_2_O, Py, AcOH, (H_2_O) r.t., 18 h; *55%*; **g.** Br_2_, (DCM) 0 °C to r.t., 18 h, *82%*; **h.** NaOH, (THF/H_2_O) 0 °C to r.t., 2 h, *quant.*; **i.** DMAP, (TFAA) 45 °C, 20 h; **j.** TFA, (H_2_O) r.t., 20 h, *1–35%*; **k.** NaOAc, (Ac_2_O) 140 °C, 3 h, *73%*/*80%*; **l.** (3 m HCl_aq_) 100 °C, 3 h, *52%*/*73%*; **m.** DIC, DMAP, (DCM) 0 °C to r.t., 1 d, *17–83%*.

### Evaluating CybE and CybF Substrate Plasticity In Vitro

With the substrate analogs **12a**–**l**, **13a**–**k**, and **14a**–**h** in hand, we first aimed to evaluate their utility for enzymatic conversion into the corresponding furanolide analogs and to determine individual conversion efficiency. The enzymatic assays for this analysis were conducted at an analytical scale of 500 μm substrate. The enzymatic reaction setup contained the enzymes CybE (5 μm) and CybF (6.5 μm) catalyzing all bond-forming reactions. At4CL1 (5 μm), a promiscuous homolog of CybB, was employed for activation of **12a**–**l** into their required CoA-thioesters **15a**–**l** (cf. Figure 2). The remaining additives were MgCl_2_ (5 mm), ATP (5 mm), coenzyme A (1 mm), and TPP (1 mm) in 100 mm Tris-HCl buffer (pH 7.8). All transformations were conducted in 100 μL scale at 25 °C overnight with subsequent detection of product formation and substrate consumption by high-resolution liquid chromatography-tandem mass spectrometry (HRLC-MS/MS). In the initial set of experiments, only substrate **12** was varied, while **13a** and **14a** were used across all assays. HRLC-MS/MS analysis revealed the formation of products with characteristic furanolide absorption at 330 ∼ 360 nm for all 12 substrate analogs **12**. The detected high-resolution molecular masses of all products matched those of the desired furanolides (Figure S2), with conversion rates ranging from acceptable 22.7% to excellent 99.3% (Figure 4). This clearly demonstrated that CybF is highly tolerant to alterations of the aromatic substitution pattern of substrates **12**. At the same time, these results also evidence the promiscuity of At4CL1, which successfully catalyzed thioester formation for all substrates **12**. The same enzymatic conversion protocol was applied to test substrate analogs **13** (using **12a** and **14a** as further building blocks). HRLC-MS/MS analyses of the assays using **13a**–**e** again showed conversion to the anticipated furanolide analogs, with yields ranging from 14.9% to 94.3% (Figure 4). However, for analogs **13g,i,k** no product formation was observed, and for **13f,h,j** only minute product amounts were visible by HRMS analysis when extracting the expected molecular ion peaks (Figure S3), with conversion rates thus approaching 0%. These results show that the enzymatic system exhibits substantial flexibility, yet only toward aliphatic substituents at the furanolide β position, successfully accommodating methyl (**13b**), ethyl (**13c**), isopropyl (**13a**), *sec*-butyl (*rac*-**13d**), and *iso*-butyl (**13e**) groups (Figure S4). Analysis of the assays with individual variation of substrate analogs **14** (using **12a** and **13a** as further building blocks) showed that **14a**–**g** with various modifications at the benzene ring were accepted to generate the corresponding furanolide products with conversion rates ranging from 5.3% to 93.2%. Only the conversion rate with substrate **14h** was very low (approximately 1.2%), proving that the indole group represents a challenge for CybE and CybF, likely due to the significantly increased size and rigidity when compared to substituted benzenes. Overall, these preliminary substrate-screening experiments to test the fidelity of CybE and CybF identified 12 suitable substrates **12**, five substrates **13**, and seven substrates **14** from the initial substrate portfolio. This opens up new possibilities for expanding the structural and functional space of furanolides through combinatorial biocatalytic approaches.

**Figure 4:**
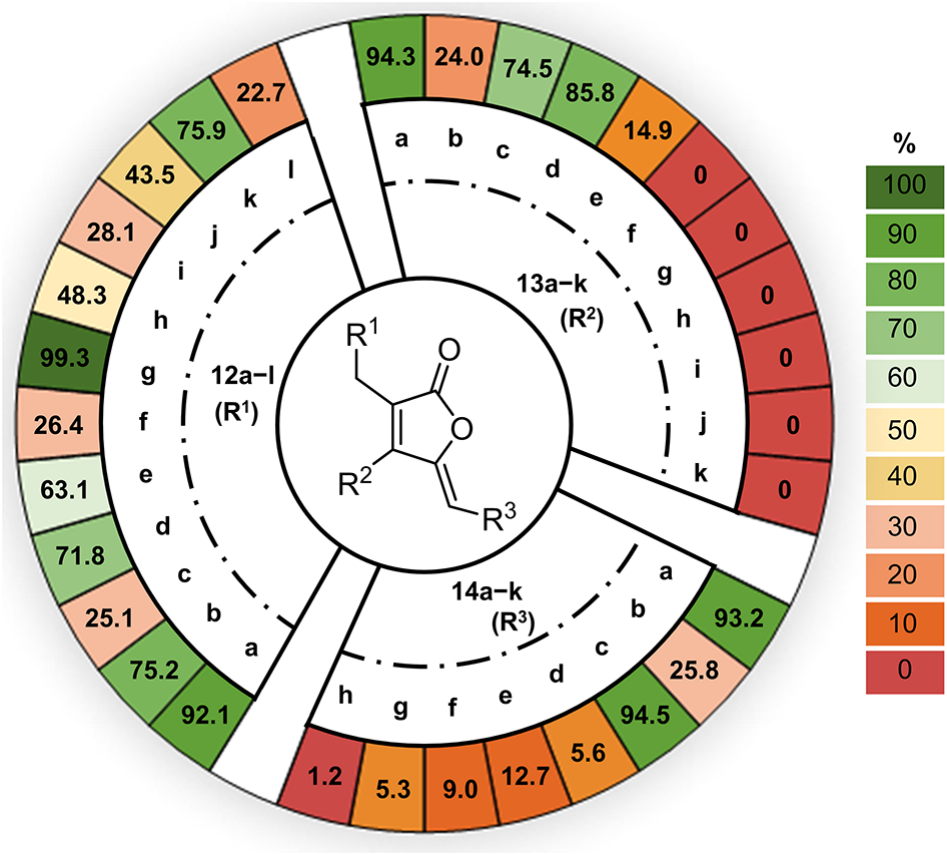
Enzymatic conversion of the substrate analogs. Assays were carried out with At4CL1 (5 μm), CybE (5 μm), CybF (6.5 μm), MgCl_2_ (5 mm), CoA (1 mm), ATP (5 mm), TPP (1 mm) and substrates (500 μm) in 100 μL of 100 mm Tris-HCl buffer (pH 7.8) at 25 °C, 450 rpm for 16 h. The reactions were analyzed by HRLC-MS/MS. Conversion (%) of substrates to the corresponding furanolide products were calculated based on HPLC peak area ratios.

### Combinatorial Enzymatic Synthesis Expanding Furanolide Structural Diversity

Given the above-established catalytic flexibility of CybE and CybF toward the tested substrates, we sought to further expand the structural diversity of furanolides and test construction of a comprehensive library of furanolide derivatives. To achieve this, a combinatorial enzymatic synthetic approach was employed, using all possible combinations of substrates **12a**–**l**, **13a**–**e**, and **14a**–**g** that have individually proven feasible during the previous experiments (**14h** was not included due to the very low conversion rate). Overall, this setup could theoretically yield 12 × 5 × 7, totaling 420 different furanolide derivatives (Figure 5A). All enzymatic reactions were conducted as described above and again analyzed by HRLC-MS/MS. To our delight, furanolide formation was detected in 385 out of the 420 combinatorial assays (Figure 5B), attesting to an excellent 91.7% overall hit rate (Figures S5-38). Aside from hundreds of new-to-nature analogs, this approach also covered the enzymatic total synthesis of previously discovered furanolide NPs, including enhygrolides A/B,^4^ deoxyenhygrolides A/B^18^ and C/D^2^, nostoclides N1 and N2,^19^ nostoclides I and II,^5^ and anhydrocyanobacterin.^20^ Remarkably, the corresponding derivatives were detected for all 35 combinatorial assays involving **12a** with all tested substrates **13a**–**e** (altering substituent R^2^) and **14a**–**g** (altering R^3^). The same catalytic proficiency was detected for all assays with **12b**, **12g**, and **12k**, consistent with the previously observed high catalytic efficiency of over 70% toward these substrate analogs of **12** (cf. Figure 4). However, some combinations – such as **13** **b**/**14f**, **13** **b**/**14g** with various substrates **12** – did not yield furanolides. A closer examination revealed that even in positive combined assays involving **13** **b**/**14f** or **13** **b**/**14g**, product formation was rather low, suggesting that CybE struggles to assemble these specific substrate pairs. Similarly, assays involving **12d** and **14d** in combination with all substrates **13** failed to produce furanolides, highlighting that the combination of **12d** and **14d** is not accepted by CybF. Furthermore, in the assays of **12** **d**/**13b** and **12** **d**/**13c**, furanolides were only generated when paired with **14a**–**c**, representing the most efficient substrates **14** in the initial substrate screening assays (cf. Figure 4). Compound **12h**, which differs from **12d** by a single chlorine substitution only, exhibited similar behavior but also showed differences, such as product formation in assays with **13** **a**/**14d** and **13** **e**/**14d**. Furthermore, no products were observed when combining **12** **h**/**13b** with any substrate **14**. Interestingly, the **12** **h**/**13** **c**/**14a** combination did not produce any product, but when natural substrate **14a** was replaced with **14b** or **14c**, products were observed. Taken together, this work established a furanolide biosynthetic enzyme−substrate activity matrix with the successful assembly of a compound library with 385 structurally diverse furanolide derivatives, providing a valuable resource for future one-pot enzymatic synthesis of these compounds.

**Figure 5:**
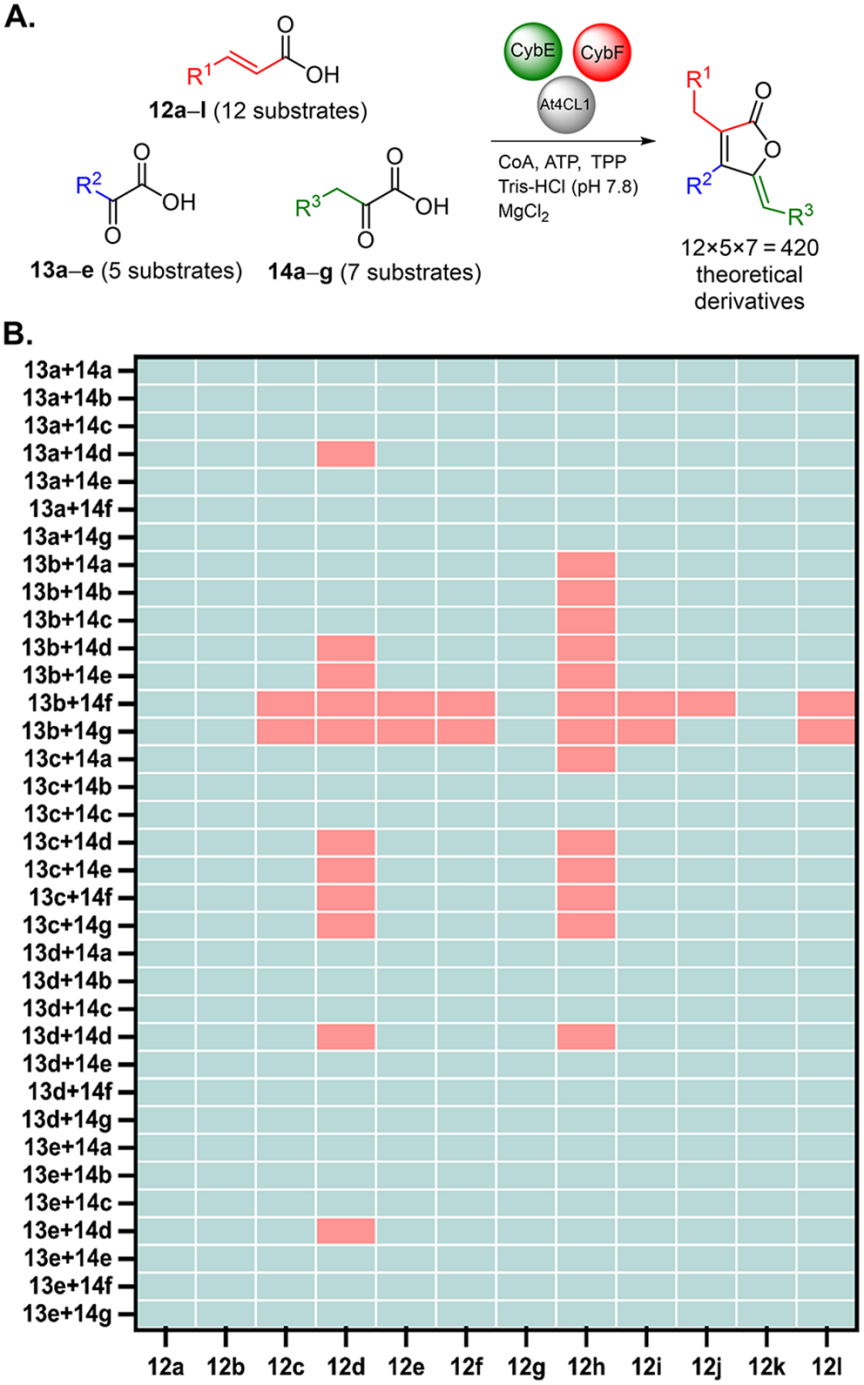
Combinatorial enzymatic synthesis of furanolides. **A.** Overview of all 420 theoretical combinations. **B.** Each small square represents an assay. Light blue indicates a positive result, with the corresponding furanolide product formed. Pink indicates a negative result with no significant furanolide production (negative results defined for experiments with relative product ion peak intensity below 0.2 × 10^4^ units). Assays were carried out with At4CL1 (5 μm), CybE (5 μm), CybF (6.5 μm), MgCl_2_ (5 mm), CoA (1 mm), ATP (5 mm), TPP (1 mm) and substrates (500 μm) in 100 μL of 100 mm Tris-HCl buffer (pH 7.8) at 25 °C, 450 rpm for 16 h. Reactions were analyzed by HRLC-MS/MS.

### Optimization of Cost-Efficiency of Enzymatic Furanolide Assembly

Before embarking on the chemo-enzymatic synthesis of natural and unnatural furanolide derivatives at a preparative scale enabling bioactivity profiling, we desired to reduce the high costs associated with the use of CoA, which can exceed 500€ per 0.1 mmol. CoA plays a pivotal role in enzymatic furanolide assembly, as it is required for activation of substrates **12** to **15** by CybB/At4CL1 to enable the initial esterification reaction catalyzed by CybF (cf. Figure 2B). To determine how CoA concentration influences product formation, we tested varying concentrations of CoA—10, 50, 250, 500, and 1000 μm—using a standard substrate concentration of 500 μm of each substrate **12a**, **13a**, and **14a**. The highest conversion rate was observed at 1000 μM CoA (defined as 100% conversion), corresponding to two equivalents CoA with respect to the substrates. At 500 μm, the conversion rate remained high at relative 93%, but gradually decreased at lower concentrations, with 66% conversion at 250 μm, 12.4% at 50 μm, and 6.3% at 10 μm (Figure S39). These findings indicate that CoA released from substrates **15** during biocatalytic furanolide assembly is not efficiently reused by CybB/At4CL1 during the reaction. CoA thus must be present in at least equimolar amounts to ensure high yields, an observation detrimental to cost-efficient, larger-scale enzymatic furanolide synthesis.

*N*-Acetyl cysteamine (**29**, SNAC) is frequently used as a cost-effective substitute for CoA when studying biosynthetic assembly of NPs.^21^ To investigate whether SNAC-activated coumaric acid analogs will serve as a viable substrate for CybF, we synthesized a small set of SNAC-thioesters for initial testing. The coumaric acids **12a**–**c** and the highly substituted **12k** were reacted overnight with *N*,*N*’-diisopropylcarbodiimide (DIC) and 4-dimethylaminopyridine (DMAP) in the presence of **29**, delivering the desired thioesters **30a**–**c** and **30k** with yields ranging from 17% to 83% (Figure 3E).

We then tested the applicability of **30a**–**c** and **30k** in enzyme assays together with substrates **13a** and **14a**, thereby not only eliminating the need for use of CoA, but also allowing to omit the thioester-forming enzyme At4CL1 and the reaction fuel ATP from the previously established enzymatic reaction system. Reactions with substrates **30a**–**c** yielded the expected products with yields of 92.5%, 74.8%, and 24.6%, respectively, which were hence comparable to the yields of the respective CoA-substrates formed in vitro using At4CL1 (Figure S40). However, **30k** exhibited poor solubility in the enzyme reaction buffer system. Even after addition of 50% (v/v) DMSO, precipitation of **30k** occurred, leading to only trace or undetectable amounts of product. Previous studies had demonstrated that fatty acyl-CoA ligases (FACLs) can catalyze the conversion of carboxylic acids into acyl-SNAC analogs,^22^ prompting us to evaluate whether At4CL1 is capable of coumaric acid activation as acyl-SNAC derivatives. To explore this, we replaced CoA with SNAC in our standard assay system containing the natural substrates **12a**, **13a**, and **14a**. The reaction indeed yielded the expected furanolide product in high yield of 91.2% (Figure S41), showcasing that At4CL1 can effectively catalyze the conversion of coumaric acid analogs into acyl-SNAC intermediates, thus offering a cost-effective pathway for the biocatalytic synthesis of furanolides. To probe the general applicability of this approach, all remaining substrates **12b**–**l** were also tested and all corresponding products were indeed formed as anticipated (Figure S41). Importantly, the enzymatic generation of activated SNAC thioesters in situ also solved the solubility issues observed for **30k** (Figure S41). These findings underscore the potential of SNAC thioesters as a functional substitute for CoA thioesters in the cost-efficient biocatalytic synthesis of furanolides, broadening the utility of At4CL1.

### Biocatalytic Total Synthesis of a Focused Furanolide Library and Its Biological Activity Evaluation

Leveraging our generated understanding of CybEF enzyme catalytic flexibility and the improved biocatalytic setup employing SNAC, we set out to preparatively synthesize a focused series of representative furanolide derivatives (**11**, **31**–**46**) (Figure 6). We initiated the synthesis with ten substrates **12a**−**h, 12j, 12k** combined with **13a** and **14a**, achieving excellent 85.5% yields for natural precyanobacterin (**11**) and acceptable to excellent yields (18.3–89.7%) for all other derivatives **31**–**39**. Subsequent additional variations using **13c** and *rac*-**13d** together with **12a**/**14a** afforded derivatives **40** (31.1%) and *rac*-**41** (57.1%), while alterations using **14b** or **14c** together with **12a**/**13a** led to compounds **42** (27.3%) and **43** (57.1%). We furthermore added three arbitrarily chosen substrate combinations with multiple changes, namely **12b**/*rac*-**13d**/**14a**, **12c**/*rac*-**13d**/**14a**, and **12k**/*rac*-**13d**/**14a**, giving rise to derivatives *rac*-**44** (59.8%), *rac*-**45** (39.8%), and *rac*-**46** (69.7%), respectively.

**Figure 6:**
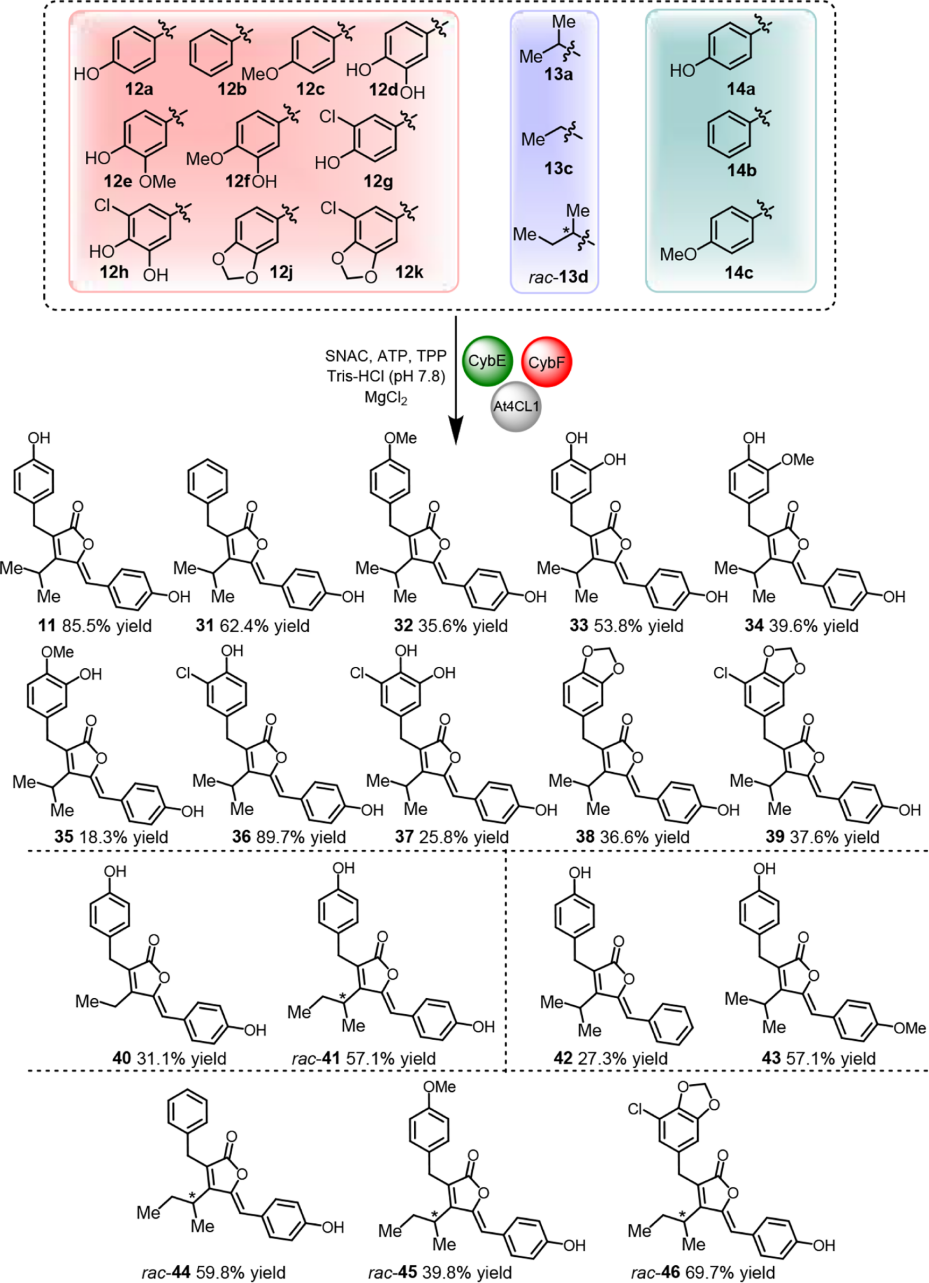
Chemo-enzymatic total synthesis of natural and unnatural furanolide derivatives. Large scale (40 mL reaction volume) in vitro reactions with At4CL1 (5 μm), CybE (5 μm), CybF (6.5 μm), MgCl_2_ (5 mm), SNAC (2 mm), ATP (5 mm), TPP (1 mm) and substrates (1 mm) in 100 mm Tris-HCl buffer (pH 7.8) were carried out at 25 °C, 450 rpm for 16 h. Isolated yields are given.

Given the reported cytotoxicity of furanolides against tumor cells,^5^ we conducted a cytotoxicity screening of the synthesized furanolide derivatives using the human lung adenocarcinoma cell line A549. In preliminary assays, we employed the MTT cell viability assay to assess the antiproliferative effects of furanolides across a range of concentrations. All derivatives exhibited inhibitory activity against A549 cells, with IC_50_ values spanning from 5.7 μm to 112.1 μm (Table S1). Notably, derivatives **39** and **43** demonstrated potent cytotoxicity, with IC_50_ values of 10.3 μm and 5.7 μm, respectively. Analysis of structure–activity relationships (SARs) revealed significant trends. Hydroxylation of the aromatic ring at the R^1^ position generally reduced potency. For instance, compound **31**, which features an unsubstituted benzene ring at R^1^, had an IC_50_ of 23.52 μm, while its hydroxylated counterparts, derivatives **11** (IC_50_ 28.28 μm, with one hydroxy group) and **33** (IC_50_ 112.1 μm, with two hydroxy groups), showed progressively diminished activity.

Conversely, chlorination of the aromatic ring at the R^1^ position significantly enhanced cytotoxicity. For example, derivative **39** (IC_50_ 10.34 μm), which contains a chlorine substitution, was nearly four times more potent than derivative **38** (IC_50_ 38.75 μm), which lacks this modification. Methylation of the phenolic hydroxy group at R^3^ resulted in a dramatic increase in cytotoxicity. For example, derivative **43** (IC_50_ 5.734 μm), which carries a methyl group within R^3^, exhibited a 5-fold increase in cytotoxicity compared to its parent compound **11** (IC_50_ 28.28 μm). Additionally, modifications such as chlorination^23^ and oxygen methylation, which are frequently observed in natural products, are known to improve both stability and bioavailability.^24^ This trend aligns with findings for other bioactive compounds; for instance, the oxygen-methylated resveratrol analog, pterostilbene, displayed a 1.9-fold increase in cytotoxicity against human cervical carcinoma (HeLa) cells compared to its unmodified counterpart.^25^ These results collectively underscore that chlorination and oxygen methylation are promising strategies for enhancing the biological activity of furanolide derivatives.

In addition, we also selected two highly active furanolide derivatives, **39** and **43**, along with two less active compounds, **33** and **37**, to evaluate their inhibitory effects on lung cancer stem cells (lung CSCs). The CSC population in A549 cells was identified using the CD133 surface marker labeled with green fluorescence for clear visualization. Through immunofluorescence imaging, we observed the morphology and density of A549 cells by staining their F-actin cytoskeleton. Compared with the DMSO control group, treatment with compounds **39** and **43** at a concentration of 10 μm resulted in a notable reduction in the number of lung cancer cells (Figure 7).

**Figure 7:**
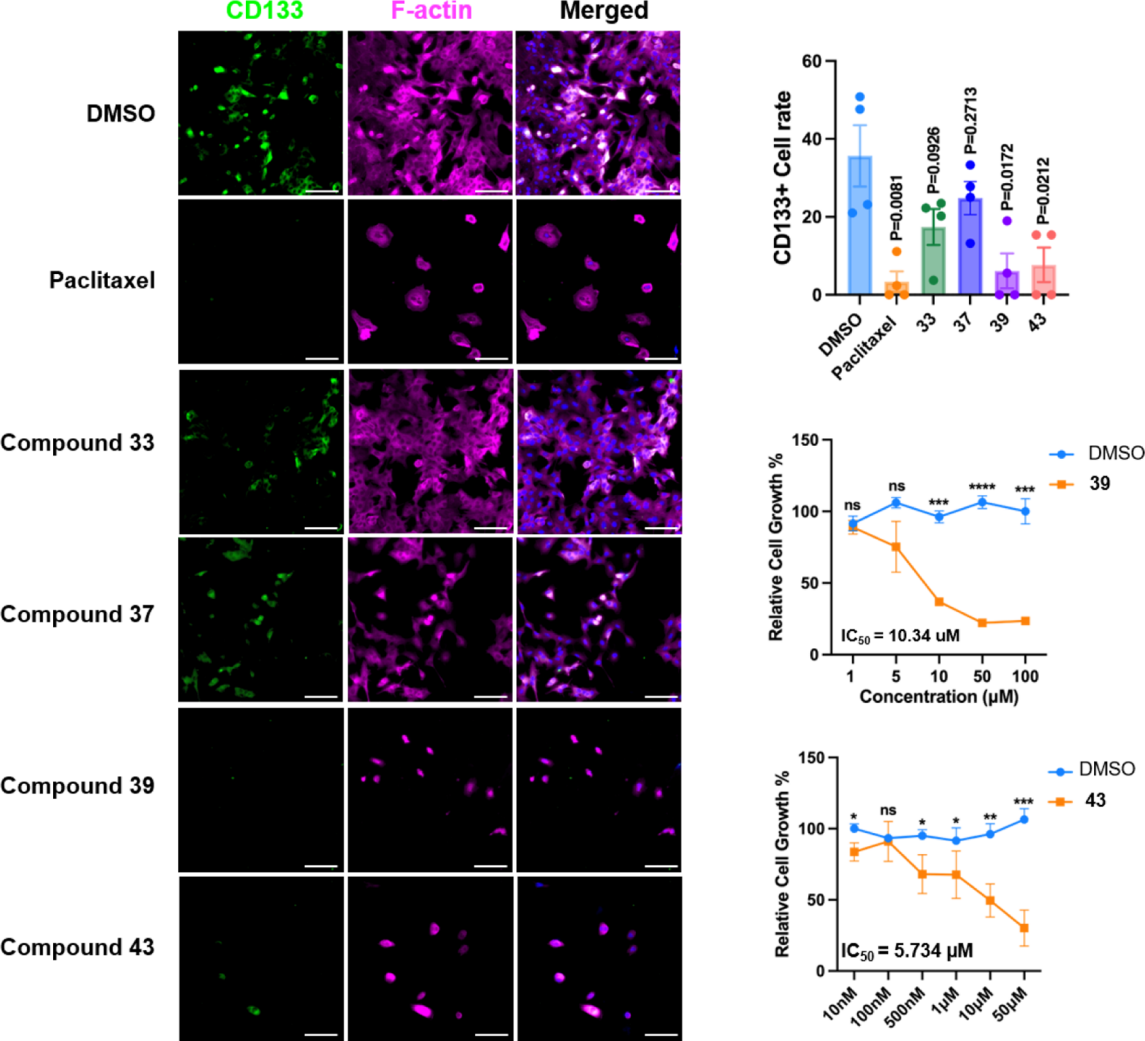
Fluorescence microscopy analysis of F-actin and CD133 in A549 cells. CD133 (green) was labeled with anti-CD133 antibody and visualized using Alexa Fluor 488-labeled antibody. F-actin (magenta) was visualized with phalloidin-Alexa Fluor 647. The nuclei were stained with DAPI (blue). Scale bar = 100 μm.

Notably, both compounds exhibited even stronger inhibitory effects on A549 cells than the clinical drug cisplatin (IC_50_ 33 μm).^26^ In contrast, no significant reduction was observed in the groups treated with compounds **33** and **37** at the same concentration. Further analysis revealed that CD133, a marker of lung CSCs, showed differential expression across treatments. In the DMSO control group, the rate of CD133+ cells within the A549 cell population was 38%. However, in the treatment groups with compounds **39** and **43**, the percentage of CD133+ cells were significantly reduced, indicating that these compounds effectively targeted lung CSCs. Conversely, treatment with compounds **33** and **37** showed no significant impact on the viability of CD133+ cells. This shows that furanolide derivatives **39** and **43** possess potent anticancer stem cell activity, highlighting their potential in targeting aggressive cancer cell populations that contribute to tumor progression and resistance.

Since some natural furanolide analogs display antimicrobial activity, we additionally tested the representative set of upscaled derivatives (**11**, **31**–**46**) in an antimicrobial testing panel. We did not find pronounced antifungal activity (*Candida albicans*) nor activity against Gram-negative*Klebsiella pneumoniae* and*Escherichia coli*, including an efflux-deficient strain (*E. coli* Δ*tolC*) with minimum inhibitory concentrations (MICs) > 64 μg/mL. Intriguingly, several derivatives efficiently inhibited the growth of often multidrug resistant (MDR) Gram-positive bacteria that are a major concern in the clinics (Table 1).

**Table 1:** Minimum Inhibitory Concentrations (MICs, in μg/mL) of Compounds **11**, **31**–**46** against Gram-Positive Bacteria^a^

Compound	*Enterococcus faecalis* ATCC29212	*E. faecium* ATCC51559 (MDR)	*Staphylococcus aureus str.* Newman	*S. aureus* ATCC29213	*Streptococcus pneumoniae*DSM20566
**11**	64	32− >64	32	>64	32
**31**	>64	>64	>64	>64	64− >64
**32**	>64	>64	16	>64	>64
**33**	>64	>64	>64	>64	64
**34**	>64	>64	>64	>64	64
**35**	64− >64	16	>64	>64	>64
**36**	16–32	8	8–16	64	8
**37**	16–32	32	>64	>64	64
**38**	>64	>64	16–32	>64	>64
**39**	>64	32− >64	16	>64	>64
**40**	>64	>64	>64	>64	>64
*rac*-**41**	64	16	16–32	64− >64	16–32
**42**	>64	16	32–64	32–64	16–32
**43**	>64	16–32	16–32	64− >64	16
*rac*-**44**	16	8	8	8	8
*rac*-**45**	>64	16–32	4–8	4	4
*rac*-**46**	64− >64	16	8	32	4
Vancomycin (ref.)	2	>64	2	1	1

a All values were determined in standard micro-broth dilution (N = 2).

Most of the tested furanolide derivatives showed moderate potency with MICs ≥ 16 μg/mL against*S. aureus*,*Enterococcus* spp. and*S. pneumoniae*. Importantly, also the two highly cytotoxic derivatives (**39** and **43**) were among the compounds with only moderate activity against the panel of tested bacteria, hinting toward specific mechanisms of action in eukaryotic versus prokaryotic cells.

Among the derivatives containing a hydroxy group at R^3^ and *iso*-propyl at R^2^ (**11**, **31**–**39**), derivative **36** with a monochlorinated and hydroxylated phenyl ring R^1^ was the most active derivative with particularly promising activity against MDR*E. faecium* and*S. pneumoniae* (MIC of 8 μg/mL). Overall, R^2^ appeared to be the main driver for antibacterial activity with *sec*-butyl as most favorable residue (incorporated in racemic **41**, **44**–**46**), while fine-tuning of activity might be achieved through variations in R^1^ and R^3^. While dihydroxylated *rac*-**41** displayed MICs ≥ 16 μg/mL across all tested organisms, compounds *rac*-**44**−**46** that do not contain a free phenol at R^1^ showed significantly improved antibacterial activity, which is in part species-specific. Overall, we found *rac*-**44** as the most promising antibacterial hit with broad-spectrum activity in the Gram-positive panel, approaching activity levels of vancomycin used as positive control in our screening.

## Conclusions

In conclusion, we systematically explored the substrate promiscuity of CybE, CybF, and At4CL1, demonstrating their remarkable biocatalytic versatility. We thereby assessed the catalytic efficiency of a diverse array of substrates, generating a comprehensive library of 385 furanolide derivatives by a combinatorial enzymatic reaction setup. This yielded an extensive data set of HR-LCMS/MS spectra, providing a valuable resource for future studies on furanolide identification from natural sources. As molecular networking techniques, particularly Global Natural Product Social Molecular Networking (GNPS),^27^ are increasingly applied to tandem mass spectrometry for natural product discovery, the availability of rich HR-LCMS/MS data is critical. Our data set significantly enhances the ability of researchers to accelerate the identification of novel furanolide derivatives. We also developed an efficient and cost-effective chemo-enzymatic approach to furanolide synthesis, utilizing SNAC as a low-cost alternative to CoA. Through this method, we successfully synthesized larger amounts of 17 representative furanolide derivatives, which demonstrated cytotoxic activity against the lung adenocarcinoma cell line A549. SAR analysis revealed that chlorination and *O*-methylation substantially increased the cytotoxic potency of these compounds. Notably, compound **39** exhibited strong cytotoxicity, with an IC_50_ of 5.73 μm, and showed activity against lung cancer stem cells (CSCs). Compound **43** also demonstrated potent anti-CSC activity. In addition, the antimicrobial profile of the compound library was assessed, revealing a promising broad-spectrum activity profile for *rac*-**44**, along with potent, more species-specific activities of **36** and *rac*-**45**/*rac*-**46**.

Overall, we established a streamlined one-pot chemo-enzymatic platform for the synthesis of diversely substituted furanolide core structures, and showed the potential of this approach for uncovering promising biological activities. Recent work by Ma and coworkers established valuable structural insights into two new CybE homologs, CsmA and BpmA, providing in-depth understanding of substrate selectivity and thus establishing a basis for targeted structural diversification of CybE-type α-hydroxy-β-keto acid products and downstream furanolide analogs.^28^ This further underlines the plasticity of furanolide biosynthetic enzymes and their resulting potential as biocatalysts for the streamlined synthesis of libraries of biomedically interesting small molecules. Further studies toward optimizing our initial bioactive hits are currently performed in our laboratories, particularly the chemo-enzymatic synthesis of antibiotic analogs with branched side chains at the R^2^-position and in-depth studies on stereochemically defined congeners.

## Supplementary Material



## Data Availability

The data that support the findings of this study are reported in the Supporting Information or available from the corresponding author upon request.

## Notes

Full experimental details and all confirmatory data (Tables and Figures) for chemical and biochemical methods, experimental procedures, additional figures including UV/VIS and IR spectra of selected compounds, MS/MS fragment analyses, HPLC/MS chromatograms including EICs, ESI+ MS, and MS/MS spectra, supplementary tables, and NMR spectra (PDF)
