# Supplementary material for: A Chemo-Enzymatic Platform for Furanolide Synthesis and Functional Exploration
Source: J Am Chem Soc. 2025 Aug 15;147(34):30979–88. doi: 10.1021/jacs.5c08354 (PMC12395478; doi:10.1021/jacs.5c08354)
Supplement: Supplementary file 1 [file ja5c08354_si_001.pdf]

A Chemo-Enzymatic Platform for Furanolide Synthesis and Functional Exploration

Xiaoqi Ji,[1] Manuel Einsiedler,[1] Paul M. D'Agostino,[1,2] Jennifer Herrmann,[3,4] Rolf Müller,[3,4] and Tobias A.M. Gulder[1,2] *

[1] Chair of Technical Biochemistry, Technische Universität Dresden, Bergstraße 66, 01069 Dresden, Germany.
[2] Department of Natural Product Biotechnology, Helmholtz Institute for Pharmaceutical Research Saarland (HIPS), Helmholtz Centre for Infection Research (HZI) and Department of Pharmacy at Saarland University, PharmaScienceHub (PSH), Campus E8.1, 66123 Saarbrücken, Germany.
[3] Department of Microbial Natural Products, Helmholtz Institute for Pharmaceutical Research Saarland (HIPS), Helmholtz Centre for Infection Research (HZI) and Department of Pharmacy at Saarland University, PharmaScienceHub (PSH), Campus E8.1, 66123 Saarbrücken, Germany.
[4] German Centre for Infection Research (DZIF), Partner Site Hannover-Braunschweig, 38124 Braunschweig, Germany.
* E-mail: tobias.gulder@helmholtz-hips.de

Supporting Information

Content

1. General Methods S2
1.1 Chemical/Analytical Methods S2
1.2 Biochemical/Molecular Biological Methods S3
2. Chemical Procedures S5
2.1 Substrate Synthesis S5
2.2 Synthesis of 30a–c and 30k S11
3. Enzymatic Assays S13
4. Biocatalytic Total Synthesis of Furanolides – Analytical Data of Isolated Products S35
5. Biological Activity Evaluation S41
6. NMR Spectra S43
7. UV and IR Spectra S80
8. Literature S98

1. General Methods

1.1 Chemical/Analytical Methods

Solvents and reagents

Solvents for HPLC and MS analysis, such as acetonitrile and methanol, were purchased from Fisher Scientific and VWR in a purity of over 99% (HPLC-grade). Water was purified and deionized using a TKA GenPure water treatment system. Dry solvents, such as dichloromethane, methanol, ethanol, and tetrahydrofuran, for procedures under inert atmosphere were prepared by distillation and dried over molecular sieves (3 Å or 4 Å). Commercial materials and other solvents were purchased at the highest commercially available quality from the providers Acros Organics, Ambeed, Alfa Aesar, BLDPharm, Carbolution, Carl Roth, Fluorochem, Merck, Sigma Aldrich, TCI Chemicals, Thermo Fisher Scientific and VWR.

Chromatography

Thin-layer chromatography (TLC) was performed on pre-coated plates of silica gel F254 (Merck) with UV detection at 254 and 365 nm. Column chromatography was performed on silica gel 60 Geduran® Si 60 (40-60 µm) (Merck). High Performance Liquid Chromatography (HPLC) analysis was performed on a Knauer system consisting of an UV-1575 Intelligent UV/VIS-Detector, DG-2080-53 3-Line Degasser, two PU-1580 Intelligent HPLC Pumps, AS-1550 Intelligent Sampler, HG-1580-32 Dynamic Mixer and a LC-NetII/ADC. The system was controlled by the Galaxie software. A reversed phase column (100-3 C18 A, 150 × 4.6 mm) with integrated pre-column manufactured by Knauer at 25 °C was used with the following solvents: A = H_2O + 0.05% TFA, B = ACN + 0.05% TFA. The separation method consisted of the following gradient system: 0–2 min: 95% A, 2–25 min: 95–5% A, 25–28 min: 5% A, 28–31 min: 95% A, with a flow rate of 1 mL/min. All traces were monitored at 220 nm. Purification of compounds was performed on a semi-preparative Medium Pressure Liquid Chromatography (MPLC) device (Reveleris X2) manufactured by Grace with reversed phase columns (C18, 12 g). The system was controlled by the Reveleris Navigator software and the eluent system consisted of A and B. The separation method used the following gradient system: 0–2 min: 95% A, 2–21 min: 95–5% A, 21–23 min: 5% A, 23–25 min: 95% A, with a flow rate of 28 mL/min.

UV and IR

UV spectra were measured on a Biochrom Ultrospec 2100 pro spectrophotometer using 1 cm path length quartz cuvettes. Compounds were dissolved in methanol at a concentration of ~0.025 mg/mL, and spectra were recorded from 200 to 500 nm at room temperature. Methanol was used as the reference solvent. All compounds were analyzed under identical conditions.

IR spectra were recorded on a Thermo Nicolet Avatar 360 FTIR spectrometer using the attenuated total reflectance technique.

HR-MS

For high resolution mass spectrometry (HR-MS) a XEVO G2X Q-TOF with electrospray ionization (ESI) manufactured by Waters, as well as an Agilent Q-TOF mass spectrometer 6538 with ESI and microchannel plate detector were used.

NMR

^{1}H and ^{13}C Nuclear Magnetic Resonance Spectra (NMR) were recorded on Bruker AVANCE 300, Ascend 500 and AVANCE 600 spectrometers at 298 K. The chemical shifts are given in δ-values (ppm) relative to TMS, and were referenced to the (residual proton) peak of the deuterated solvent ($CDCl_3$: δ_H = 7.26 ppm, δ_C = 77.16 ppm; DMSO-d_6: δ_H = 2.50 ppm, δ_C = 39.52 ppm, MeOD-d_4: δ_H = 3.31 ppm, δ_C = 49.00 ppm). The coupling constants *J* are given in Hertz [Hz]. The following abbreviations were used for the allocation of signal multiplicities, assuming first-order spin–spin coupling: bs – broad singlet, vbs – very broad singlet, s – singlet, d – doublet, bd – broad doublet, dd – doublet of doublets, ddd – doublet of doublets of doublets, t – triplet, dt – doublet of triplets, q – quartet, dq – doublet of quartets, p – pentet, h – heptet, sept – septet, m – multiplet.

1.2 Biochemical/Molecular Biological Methods

Recombinant CybE, CybF and At4CL1 were produced as follows:

Bacterial cultures

E. coli BL21 (DE3) carrying pHis8-TEV-cybE or cybF or At4CL1 were incubated overnight at 37 °C in LB medium containing 50 µg/mL kanamycin. The overnight seed culture was inoculated into 3 L of TB medium containing 50 µg/mL kanamycin and grown at 37 °C to OD_{600} ≈ 0.6–0.8. The cells were then cooled on ice for 60 min and induced with 0.4 mM isopropyl β-D-thiogalactopyranoside (IPTG). The cells were grown for another 20 h at 16 °C. The cells were harvested by centrifugation (7000 rpm, 10 min), washed with binding buffer (50 mM Tris-HCl, 150 mM NaCl, 10 mM imidazole, and 10% glycerol, pH 8.0), and stored as pellets in Falcon tubes at –80 °C.

Protein purification

The cell pellets were resuspended in binding buffer (3 mL binding buffer per 1 mL pellet) and sonicated on ice. Cell debris was removed by centrifugation (12,000 rpm, 60 min, 4 °C). 4 mL of nickel NTA resin suspension was applied on a HisPur NTA column from Thermo Fisher Scientific. The supernatant was loaded onto a HisPur NTA column.

For the purification of the three enzymes, three different methods were used:

For CybE, the protein-loaded nickel beads were first washed with 15 mL of binding buffer, then with 15 mL of wash buffer 1 (50 mM Tris-HCl, 150 mM NaCl, 35 mM imidazole, 10% glycerol, pH 8.0), then with 10 mL of wash buffer 2 (50 mM Tris-HCl, 150 mM NaCl, 55 mM imidazole, 10% glycerol, pH 8.0), and finally eluted with 30 mL elution buffer (50 mM Tris-HCl, 150 mM NaCl, 500 mM imidazole, 10% glycerol, pH 8.0). Fractions containing the target protein were collected, desalted on Sephadex G-25 PD-10 columns from GE Healthcare, and concentrated by ultrafiltration (Millipore membrane, 30 kDa cut-off size).

For CybF, the protein-loaded nickel beads were first washed with 15 mL of binding buffer, then with 10 mL of wash buffer 1, then with 6 mL of wash buffer 2, and finally eluted with 10 mL elution buffer. Fractions containing the target protein were collected, desalted on Sephadex G-25 PD-10 columns from GE Healthcare, and concentrated by ultrafiltration (Millipore membrane, 10 kDa cut-off size).

For At4CL1, the protein-loaded nickel beads were first washed with 15 mL of binding buffer, then with 10 mL of wash buffer 1, and finally eluted with 15 mL elution buffer. Fractions containing the target protein were collected, desalted on Sephadex G-25 PD-10 columns from GE Healthcare, and concentrated by ultrafiltration (Millipore membrane, 30 kDa cut-off size).

The purified proteins were analyzed by SDS-PAGE (Figure S1) and stored in storage buffer (50 mM Tris-HCl, 150 mM NaCl, 10% glycerol, pH 8.0) at −80 °C. The concentration of the purified proteins was determined using a NanoDrop system.

Enzymatic conversion analysis of CybE and CybF to test substrate specificity

The activity assays were carried out with At4CL1 (5 µM), CybE (5 µM), CybF (6.5 µM), $MgCl_2$ (5 mM), CoA (1 mM), ATP (5 mM), TPP (1 mM) and substrates (500 µM) in 100 µL of 100 mM Tris-HCl buffer (pH 7.8) at 25 °C, 450 rpm for 16 h.[1] The reaction was stopped by adding 100 µL of MeOH, and the precipitated protein was removed by centrifugation (13,000 rpm, 10 min, 25 °C). The supernatant was subjected to HRLC-MS/MS analyses. For the analysis of substrates functionalized with SNAC, ATP, CoA and At4CL1 were omitted from the assay. For reactions performed with in situ enzymatic SNAC activation, CoA was replaced with SNAC in the assays.

Chemo-enzymatic synthesis of furanolide derivatives

The enzymatic synthesis was carried out with At4CL1 (5 µM), CybE (5 µM), CybF (6.5 µM), $MgCl_2$ (5 mM), SNAC (2 mM), ATP (5 mM), TPP (1 mM) and substrates (1 mM) in 30 mL of 100 mM Tris-HCl buffer (pH 7.8) at 25 °C, 450 rpm for 16 h. The product was extracted ethyl acetate (3 × 60 mL), the organic solvent evaporated in vacuo, and the resuspended material purified by MPLC.

Bioactivity testing of furanolide derivatives against A549 cells

The human lung carcinoma epithelial cell line (A549) was obtained from Sigma-Aldrich (86012804) and maintained in Dulbecco's Modified Eagle Medium (DMEM) (Gibco, 41965039) supplemented with 10% fetal bovine serum (FBS) (BioConcept, 2-02F110-I) and 1× Penicillin/Streptomycin (Thermo Fisher Scientific, 15140122). Cells were seeded at a density of 1 × 10^4 cells per well in 96-well plates and underwent a 12-hour starvation period using DMEM containing 1% FBS to synchronize cell growth phases. Following starvation, cells were exposed to increasing concentrations of the compounds to be tested (1, 5, 10, and 50 µM) and cultured in fresh DMEM (containing 10% FBS, 1× Penicillin/Streptomycin). Each assay was replicated four times. Control wells received an equivalent volume of dimethylsulfoxide (DMSO) (Sigma, 4720.4). Paclitaxel (Sigma-Aldrich, Y0000700) served as the positive control. The cells were incubated at 37 °C in a humidified atmosphere containing 5% CO_2. After 48 hours of treatment, cell viability was quantified using the MTT Cell Proliferation Assay Kit (Biozol Diagnostica, OZB-MT01000), with absorbance measured on a SpectraMax® i3x spectrophotometer.

Immunofluorescence staining

A549 cells were plated at a density of 2 × 10^3 cells per well on 8-well Chamber Slides (Life Technologies, 177402PK) pre-coated with 0.01% poly-L-lysine solution bioreagent (Sigma-Aldrich, P4832) for 30 minutes to enhance cell adhesion. Cells underwent a starvation period in DMEM containing 1% FBS and 1× Penicillin/Streptomycin (Thermo Fisher Scientific, 15140122) for 12 hours to synchronize their growth phase. Test compounds were administered at a concentration of 10 µM in DMSO, with three replicates per treatment. Following this, the culture medium was replaced with DMEM supplemented with 10% FBS. After a 48-hour treatment period, cells were fixed with 2% paraformaldehyde (PFA, VWR, 43368.9 M) for 10 minutes and subsequently washed three times with fresh PBS (Thermo Fisher). The slides were then rinsed three times with PBS containing 0.05% Tween-20 (PBST) and blocked for one hour at room temperature with 10% serum in PBST. An overnight incubation at 4 °C with a 1:200 dilution of CD133 primary antibody (Biozol Diagnostica, BYT-ORB99113-100-ICH) in blocking buffer was followed by three washes in PBST. Slides were incubated with an Alexa Fluor 488-conjugated secondary antibody (1:500 dilution, Life Technologies) and phalloidin (1:500 dilution, Biomol, ABD-23127) in blocking solution for 60 minutes at room temperature. After the final washes, slides were mounted with fluorescent mounting media containing 4,6-diamidino-2-phenylindole (DAPI). Samples were imaged using a ZEISS AxioImager.Z2m (Carl Zeiss) with a 40× objective.

Antimicrobial testing

Bacterial strains were either purchased from the American Type Culture Collection (ATCC), the German Collection of Microorganisms and Cell Cultures (DSMZ), Coli Genetic Stock Center (CGSC), or were part of our internal strain collection. Minimal inhibitory concentrations (MICs) were determined using standard broth microdilution according to EUCAST guidelines (ISO20776-1:2019) in round-bottom 96-well plates. Regular quality control was performed using appropriate reference antibiotics. In brief, serially diluted compounds (0.03-64 µg/mL) were tested against *Candida albicans* DSM-1665, *Escherichia coli* BW25113 (K12 WT), TolC-deficient *Escherichia coli* K12, *Klebsiella pneumoniae* DSM-30104, *Enterococcus faecalis* ATCC29212, *Enterococcus faecium* ATCC51559, *Staphylococcus aureus* str. Newman, *Staphylococcus aureus* ATCC29213, and *Streptococcus pneumoniae* DSM20566. An initial inoculum of approximately 5 × 10^5 colony-forming units (CFU)/mL was adjusted, and microorganisms were grown for 18–24 hours at 37 °C prior to determining MIC. Results were recorded as the lowest concentration of antimicrobial agent that inhibited visible growth of the bacteria.

2. Chemical Procedures

2.1 Substrate Synthesis

ethyl (*E*)-3-(3-chloro-4-hydroxy-5-methoxyphenyl)acrylate

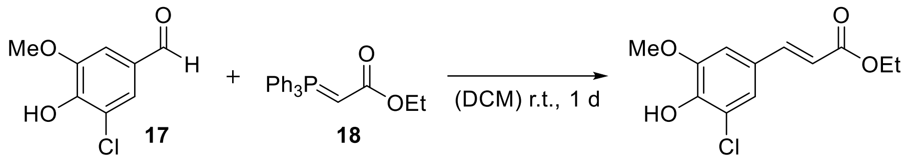

In a flame-dried 50 mL Schlenk flask, 2.0 g 5-chlorovanillin (**17**, 10.7 mmol, 1.0 eq.) was dissolved in DCM (14 mL) and 5.6 g ethyl (triphenylphosphoranylidene)acetate (**18**, 16.1 mmol, 1.5 eq.) was added in three portions. The solution was stirred at room temperature for one day and the solvent was removed under reduced pressure. The residue was purified by column chromatography (pentane:EtOAc = 3:1) to yield 2.26 g of the desired product as white crystals (8.8 mmol, 82%).

^{1}H-NMR (300 MHz, $CDCl_3$): δ [ppm] = 7.53 (d, *J* = 15.9 Hz, 1H), 7.15 (d, *J* = 1.9 Hz, 1H), 6.93 (d, *J* = 1.9 Hz, 1H), 6.30 (d, *J* = 15.9 Hz, 1H), 6.30 (bs, 1H), 4.25 (q, *J* = 7.1 Hz, 2H), 3.94 (s, 3H), 1.33 (t, *J* = 7.1 Hz, 3H).

^{13}C{^{1}H}-NMR (75.5 MHz, $CDCl_3$): δ [ppm] = 167.0, 147.7, 144.0, 143.5, 127.2, 122.9, 120.1, 117.4, 108.2, 60.7, 56.6, 14.5.

R_f (pentane/EtOAc = 3/1): 0.41

HR-MS (ESI+): *m/z* calcd. for $C_{12}H_{13}ClO_4$ $[M+Na]^+$: 279.0395; found: 279.0398.

(*E*)-3-(3-chloro-4-hydroxy-5-methoxyphenyl)acrylic acid (**12i**)

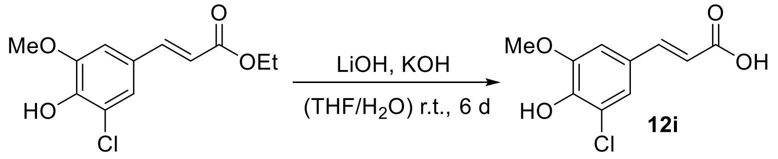

1.5 g ethyl (*E*)-3-(3-chloro-4-hydroxy-5-methoxyphenyl)acrylate (5.8 mmol, 1.0 eq.) was dissolved in a mixture of THF and water (16 mL/8 mL). 0.42 g LiOH (17.5 mmol, 3.0 eq.) was added and the mixture was stirred at room temperature. As after one day only little consumption of starting material was detected by TLC, 110 mg of LiOH was added. After another day of stirring, still a large amount of starting material was present in the mixture. Next, 0.2 g KOH (5.0 mmol) was added, and the yellow suspension stirred at room temperature for another four days. Afterwards, TLC showed complete substrate consumption. The mixture was diluted with 20 mL water and extracted with 30 mL of Et_2O. The aqueous phase was acidified to pH 1 with 4 M HCl_{aq} and extracted with EtOAc (2 × 60 mL). Combined organic extracts were washed with brine (50 mL), dried over Na_2SO_4, filtered and the solvents removed under reduced pressure to yield 1.3 g of a yellowish solid (**12i**, 5.7 mmol, 97%), which was used without further purification.

^{1}H-NMR (600 MHz, DMSO-*d*$_6$): δ [ppm] = 12.26 (bs, 1H), 9.95 (bs, 1H), 7.47 (d, *J* = 15.9 Hz, 1H), 7.313 (d, *J* = 2.0 Hz, 1H), 7.307 (d, *J* = 2.0 Hz, 1H), 6.47 (d, *J* = 15.9 Hz, 1H), 3.87 (s, 3H).

^{13}C{^{1}H}-NMR (151 MHz, DMSO-*d*$_6$): δ [ppm] = 167.8, 148.8, 144.8, 143.2, 126.0, 122.8, 120.0, 117.5, 109.5, 56.4.

HR-MS (ESI+): *m/z* calcd. for $C_{10}H_9ClO_4$ $[M+Na]^+$: 251.0082; found: 251.0085.

(*E*)-3-(3-chloro-4,5-dihydroxyphenyl)acrylic acid (**12h**)

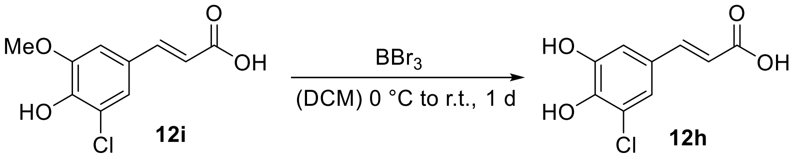

In a flame-dried 100 mL Schlenk flask, 0.4 g of (*E*)-3-(3-chloro-4-hydroxy-5-methoxyphenyl)acrylic acid (**12i**, 1.75 mmol, 1.0 eq.) was suspended in 5 mL of DCM and BBr_3 (1 M in DCM, 10.5 mL, 10.5 mmol, 6.0 eq.) was

added slowly at 0 °C. The resulting solution was stirred at room temperature for one day and poured on ice water (50 mL). The organic phase of the suspension was separated, and the volatiles removed under reduced pressure. The aqueous phase was extracted with EtOAc (15 mL). The residue of the organic phases was taken up in 30 mL EtOAc and combined with the extract from the first step. Residual water was removed, and the organic phase washed with brine (20 mL), dried over Na_2SO_4, filtered and the solvent removed under reduced pressure. The residue was purified by MPLC to yield 0.23 g of **12h** as a slightly red solid (1.07 mmol, 61%).

^{1}H-NMR (300 MHz, DMSO-*d*$_6$): δ [ppm] = 12.23 (vbs, 1H), 9.86 (vbs, 2H), 7.39 (d, *J* = 15.9 Hz, 1H), 7.19 (d, *J* = 2.0 Hz, 1H), 7.00 (d, *J* = 2.0 Hz, 1H), 6.24 (d, *J* = 15.9 Hz, 1H).
^{13}C{^{1}H}-NMR (75.5 MHz, DMSO-*d*$_6$): δ [ppm] = 167.6, 146.7, 144.2, 143.3, 125.9, 121.0, 120.5, 116.9, 113.0.
HR-MS (ESI+): *m/z* calcd. for $C_9H_7ClO_4$ $[M+Na]^+$: 236.9926; found: 236.9927.

3,5-dibromo-4-hydroxybenzaldehyde (**20**)

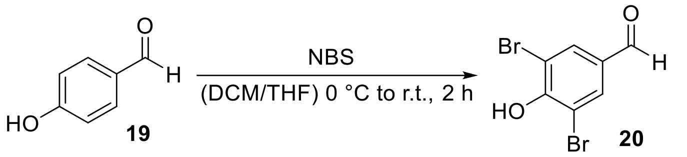

In a 100 mL round-bottomed flask, 4-hydroxybenzaldehyde (**19**, 1.50 g, 12.3 mmol, 1.0 eq.) was dissolved in a mixture of DCM and THF (20 mL/10 mL), cooled to 0 °C, and *N*-bromosuccinimide (4.59 g, 25.8 mmol, 2.1 eq.) was added. The resulting solution was stirred at room temperature for 2 hours, then water (15 mL) was added. The organic phase was separated, and the aqueous phase extracted with DCM (25 mL). Combined organic extracts were washed with saturated $Na_2S_2O_3$ solution (2 × 10 mL) and brine (25 mL), dried over Na_2SO_4, filtered and the solvents removed under reduced pressure. The product **20** was obtained as yellowish solid (3.12 g, 11.1 mmol, 91%) and used without further purification.

^{1}H-NMR (300 MHz, $CDCl_3$): δ [ppm] = 9.80 (s, 1H), 8.00 (s, 2H), 6.41 (bs, 1H).
Analytical data matched those reported in literature.[2]

(*E*)-3-(3,5-dibromo-4-hydroxyphenyl)acrylic acid (**12l**)

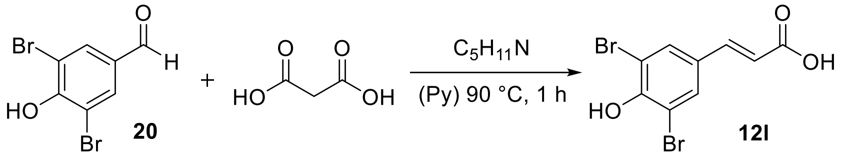

In a 50 mL round-bottomed flask, 1.0 g of 3,5-dibromo-4-hydroxybenzaldehyde (**20**, 3.57 mmol, 1.0 eq.) and 0.82 g of malonic acid (7.86 mmol, 2.2 eq.) were dissolved in 6 mL of pyridine. Piperidine (0.15 mL, 0.13 g, 1.51 mmol, 0.42 eq.) was added and the mixture heated to 90 °C for one hour. After cooling to room temperature, the solvents were removed under reduced pressure and the residue acidified by addition of 1 M HCl_{aq}. The formed solid was filtered, washed with water and dried by lyophilization to afford 0.92 g of the crude product **12l** as a slightly pink solid. A small amount was purified by preparative HPLC to yield 76 mg of a white solid.

^{1}H-NMR (300 MHz, DMSO-*d*$_6$): δ [ppm] = 12.36 (bs, 1H), 10.41 (bs, 1H), 7.94 (s, 2H), 7.46 (d, *J* = 16.0 Hz, 1H), 6.49 (d, *J* = 16.0 Hz, 1H).
^{13}C-NMR (75.5 MHz, DMSO-*d*$_6$): δ [ppm] = 167.5, 152.2, 141.2, 132.1 (2C), 129.0, 119.1, 112.1 (2C).
HR-MS (ESI+): *m/z* calcd. for $C_9H_6Br_2O_3$ $[M+H]^+$: 322.8736; found: 322.8729.

methyl 2-(4-hydroxyphenyl)-2-oxoacetate[3] (**23**)

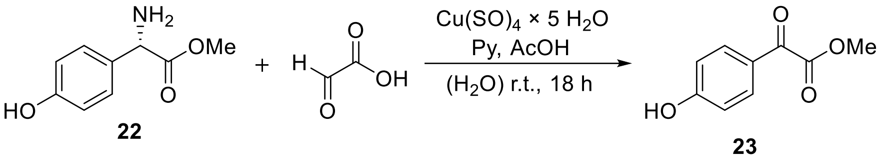

22 **23**

In a 100 mL round-bottomed flask, 2.0 g L-4-hydroxyphenylglycine methyl ester hydrochloride (**22**, 9.2 mmol, 1.0 eq.) was added to a solution of 8.5 g of glyoxylic acid (92 mmol, 10 eq.) and 2.3 g $CuSO_4$ × 5 H_2O (9.2 mmol, 1.0 eq.) in 30 mL of an aqueous buffer containing 2.5 M pyridine and 0.5 M acetic acid. The resulting mixture was stirred at room temperature for 18 hours, before being extracted with DCM (3 × 30 mL). Combined organic extracts were washed with 0.5 M HCl_{aq}, dried over Na_2SO_4, filtered and the solvent removed under reduced pressure. The residue was purified by column chromatography to yield 0.92 g of **23** as a yellowish solid (5.1 mmol, 55%).

^{1}H-NMR (300 MHz, $CDCl_3$): δ [ppm] = 7.97 (d, *J* = 8.8 Hz, 2H), 6.93 (d, *J* = 8.8 Hz, 2H), 6.16 (bs, 1H), 3.96 (s, 3H).
^{13}C{^{1}H}-NMR (75.5 MHz, $CDCl_3$): δ [ppm] = 184.7, 164.5, 162.1, 133.2 (2C), 125.6, 116.1 (2C), 52.9.
***R*f** (pentane/EtOAc = 1/1): 0.53
HR-MS (ESI+): *m/z* calcd. for $C_9H_8O_4$ $[M+Na]^+$: 203.0315; found: 203.0314.

methyl 2-(3-bromo-4-hydroxyphenyl)-2-oxoacetate

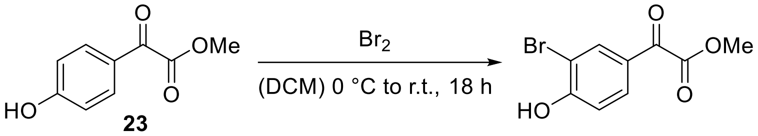

23

In a flame-dried 50 mL Schlenk flask, 0.5 g methyl 2-(4-hydroxyphenyl)-2-oxoacetate (**23**, 2.77 mmol, 1.0 eq.) was dissolved in 10 mL DCM. At 0 °C, a solution of 135 µL bromine (0.42 g, 2.64 mmol, 0.95 eq.) in 3 mL DCM was slowly added. The solution was stirred at room temperature under argon for 18 hours. After dilution with 20 mL of DCM, the solution was washed with saturated $Na_2S_2O_3$ solution (30 mL). The aqueous phase was extracted with 20 mL DCM and combined organic phases were washed with brine (40 mL), dried over Na_2SO_4, filtered and the solvent removed under reduced pressure. The residue was purified by column chromatography to yield 0.6 g of a yellowish solid containing unreacted starting material. As separation by chromatography was not possible, the yield was calculated by NMR (2.27 mmol, 82%) and the product used in the next step without further purification.

^{1}H-NMR (300 MHz, $CDCl_3$): δ [ppm] = 8.24 (d, *J* = 2.1 Hz, 1H), 7.96 (dd, *J* = 8.6, 2.1 Hz, 1H), 7.11 (d, *J* = 8.6 Hz, 1H), 6.37 (bs, 1H), 3.97 (s, 3H).
^{13}C{^{1}H}-NMR (75.5 MHz, $CDCl_3$): δ [ppm] = 183.1, 163.6, 158.2, 135.0, 132.2, 126.9, 116.5, 111.1, 53.1.
***R*f** (pentane/EtOAc = 2/1): 0.53
HR-MS (ESI+): *m/z* calcd. for $C_9H_7BrO_4$ $[M+Na]^+$: 280.9420, 282.9400; found: 280.9422, 282.9402.

2-(3-bromo-4-hydroxyphenyl)-2-oxoacetic acid (**13k**)

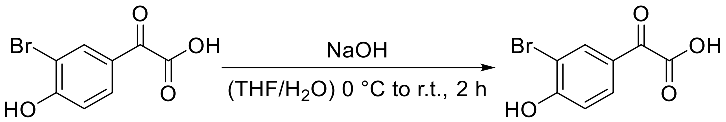

13k

In a 50 mL round-bottomed flask, 0.4 g of methyl 2-(3-bromo-4-hydroxyphenyl)-2-oxoacetate (1.54 mmol, 1.0 eq.) was dissolved in THF (12 mL). After addition of 1 M $NaOH_{aq}$ (3.9 mL, 3.9 mmol, 2.5 eq.) at 0 °C, the mixture was vigorously stirred at room temperature. After complete consumption of starting material as indicated by TLC, the mixture was diluted with water (15 mL) and extracted with Et_2O (20 mL). The organic phase was extracted with water (10 mL) and the combined aqueous phases were acidified to pH 1 by addition of 1 M HCl_{aq}. After extraction with EtOAc (2 × 30 mL), organic extracts were washed with brine (20 mL), dried over Na_2SO_4, filtered and the

volatiles removed under reduced pressure. The crude residue (0.38 g in total) was purified by preparative HPLC to yield 70 mg of **13k** as a yellowish powder.

^{1}H-NMR (300 MHz, DMSO-*d*$_6$): δ [ppm] = 14.28 (vbs, 1H), 11.76 (bs, 1H), 8.03 (d, *J* = 2.1 Hz, 1H), 7.82 (dd, *J* = 8.6, 2.1 Hz, 1H), 7.12 (d, *J* = 8.6 Hz, 1H).
^{13}C{^{1}H}-NMR (151 MHz, DMSO-*d*$_6$): δ [ppm] = 185.9, 166.0, 160.4, 134.6, 131.4, 124.8, 116.6, 110.1.
HR-MS (ESI+): *m/z* calcd. for $C_8H_5BrO_4$ $[M+Na]^+$: 266.9264, 268.9243; found: 266.9262, 268.9242; (ESI–): *m/z* calcd. $[M–H]^-$: 242.9298, 244.9277; found: 242.9303, 244.9280.

2-Hydroxy-3-arylacrylic acids from amino acids
General procedure[4]

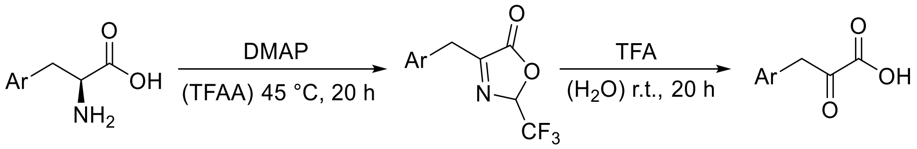

The corresponding amino acid (1.0 eq.) and a small amount of DMAP (around 10 mg) were suspended in 4 mL of trifluoroacetic anhydride and heated to 50 °C under argon for 20 hours, during which time the material completely dissolved. The solvent was removed under a stream of nitrogen, the oily residue was dissolved in 6 mL of 70% TFA and stirred at room temperature for 16 hours. A white precipitate was formed, and the mixture was left to stand at 4 °C for 6 hours before being filtered. The solid was washed with a small amount of cold water and dried by lyophilization.

(*Z*)-2-hydroxy-3-(4-methoxyphenyl)acrylic acid (**14c**)

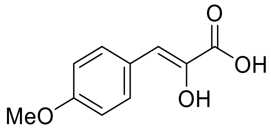

14c

The product was synthesized from L-*O*-methyltyrosine and obtained as 5.0 mg of a white solid (26 µmol, 1%).

^{1}H-NMR (300 MHz, DMSO-*d*$_6$): δ [ppm] = 13.02 (vbs, 1H), 8.96 (bs, 1H), 7.71 (d, *J* = 8.8 Hz, 2H), 6.92 (d, *J* = 8.8 Hz, 2H), 6.37 (s, 1H), 3.76 (s, 3H).
^{13}C{^{1}H}-NMR (75.5 MHz, DMSO-*d*$_6$): δ [ppm] = 166.5, 158.4, 140.0, 130.8 (2C), 127.6, 113.8 (2C), 109.8, 55.1.
HR-MS (ESI+): *m/z* calcd. for $C_{10}H_{10}O_4$ $[M+Na]^+$: 217.0472; found: 217.0475.

(*Z*)-3-(3-chloro-4-hydroxyphenyl)-2-hydroxyacrylic acid (**14e**)

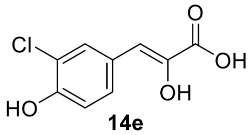

14e

The product was synthesized from L-3-chlorotyrosine and obtained as 23.0 mg of a white solid (107 µmol, 8%).

^{1}H-NMR (600 MHz, DMSO-*d*$_6$): δ [ppm] = 13.09 (bs, 1H), 10.35 (s, 1H), 9.14 (bs, 1H), 7.86 (d, *J* = 2.2 Hz, 1H), 7.46 (dd, *J* = 8.6, 2.1 Hz, 1H), 6.93 (d, *J* = 8.5 Hz, 1H), 6.31 (s, 1H).
^{13}C{^{1}H}-NMR (151 MHz, DMSO-*d*$_6$): δ [ppm] = 166.4, 152.3, 140.5, 130.4, 129.5, 127.4, 119.6, 116.5, 108.8.
HR-MS (ESI–): *m/z* calcd. for $C_9H_7ClO_4$ $[M–H]^-$: 212.9960; found: 212.9959.

(*Z*)-2-hydroxy-3-(3,5-dichloro-4-hydroxyphenyl)acrylic acid (**14f**)

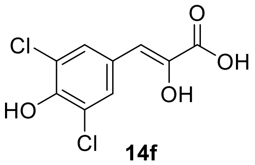

14f

The product was synthesized from L-3,5-dichlorotyrosine and obtained as 70 mg of a white solid (0.28 mmol, 23%).

^{1}H-NMR (600 MHz, DMSO-d_6): δ [ppm] = 13.27 (vbs, 1H), 10.27 (vbs, 1H), 9.55 (vbs, 1H), 7.79 (s, 2H), 6.32 (s, 1H).
^{13}C{^{1}H}-NMR (151 MHz, DMSO-d_6): δ [ppm] = 166.0, 147.9, 141.8, 129.0 (2C), 128.4, 122.0 (2C), 107.2.
HR-MS (ESI+): *m/z* calcd. for $C_9H_6Cl_2O_4$ $[M+Na]^+$: 270.9536, 272.9506; found: 270.9531, 272.9506.

(*Z*)-2-hydroxy-3-(3,5-dibromo-4-hydroxyphenyl)acrylic acid (**14g**)

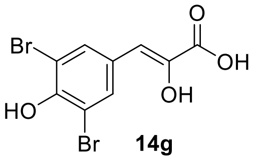

14g

The product was synthesized from L-3,5-dibromotyrosine and obtained as 106 mg of a white solid (0.31 mmol, 35%).

^{1}H-NMR (500 MHz, DMSO-d_6): δ [ppm] = 13.24 (vbs, 1H), 10.05 (bs, 1H), 9.44 (s, 1H), 7.97 (s, 2H), 6.31 (s, 1H).
^{13}C{^{1}H}-NMR (126 MHz, DMSO-d_6): δ [ppm] = 166.0, 149.5, 141.8, 132.7 (2C), 129.9, 111.7 (2C), 107.0.
HR-MS (ESI–): *m/z* calcd. for $C_9H_6Br_2O_4$ $[M-H]^-$: 336.8539; found: 336.8537.

Erlenmeyer synthesis of oxazolones

General procedure[5]

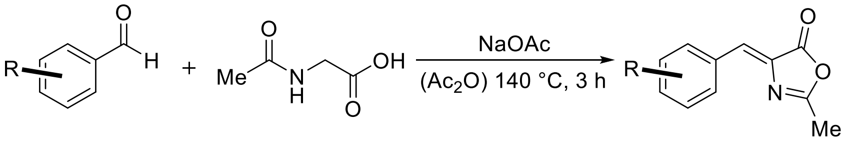

In a 50 mL round-bottomed flask, the corresponding aldehyde (1.0 eq.), *N*-acetylglycine (1.3 eq.) and sodium acetate (1.3 eq.) were suspended in acetic anhydride (0.45 mL/mmol aldehyde) and the mixture heated to reflux for three hours. After cooling to room temperature, the slurry was poured into 200 mL of cold water and stirred for one hour. The suspension was filtered and dried by lyophilization to yield the corresponding oxazolone, which was used without further purification.

(*Z*)-4-(4-methoxybenzylidene)-2-methyloxazol-5(4*H*)-one (**28c**)

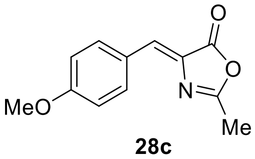

28c

Compound **28c** was synthesized from 4-hydroxybenzaldehyde, yielding 2.43 g of a brown solid (10.7 mmol, 73%), which was contaminated with 6 mol-% of starting material but used in the next step without further purification.

^{1}H-NMR (300 MHz, $CDCl_3$): δ [ppm] = 8.06 (d, J = 8.9 Hz, 2H), 7.11 (s, 1H), 6.96 (d, J = 8.9 Hz, 2H), 3.87 (s, 3H), 2.39 (s, 3H).
^{13}C{^{1}H}-NMR (75.5 MHz, $CDCl_3$): δ [ppm] = 168.3, 165.0, 162.2, 134.4 (2C), 131.7, 130.5, 126.3, 114.6 (2C), 55.6, 15.8. Analytical data matched those reported in the literature.[6]

(*Z*)-2-methoxy-4-((2-methyl-5-oxooxazol-4(5*H*)-ylidene)methyl)phenyl acetate[5]

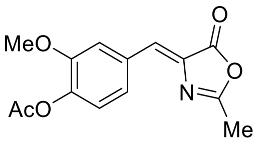

This compound was synthesized from vanillin, yielding 4.34 g of an orange solid (15.8 mmol, 80%).

^{1}H-NMR (300 MHz, $CDCl_3$): δ [ppm] = 7.93 (d, *J* = 1.9 Hz, 1H), 7.55 (dd, *J* = 8.2, 1.9 Hz, 1H), 7.10 (d, *J* = 8.2 Hz, 1H), 7.09 (s, 1H), 3.90 (s, 3H), 2.40 (s, 3H), 2.33 (s, 3H).

^{13}C{^{1}H}-NMR (151 MHz, $CDCl_3$): δ [ppm] = 168.8, 167.9, 166.3, 151.4, 142.2, 132.6, 132.1, 130.7, 125.9, 123.3, 115.4, 56.1, 20.8, 15.9.

Analytical data matched those reported in the literature.[5]

Oxazolone hydrolysis

General procedure[5]

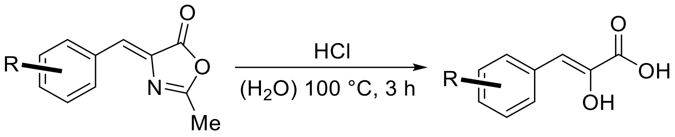

In a 50 mL round-bottomed flask, the corresponding oxazolone (1.0 eq.) was suspended in 20 mL of 3 M HCl_{aq} and the mixture heated to reflux for three hours. After cooling to room temperature, the flask was stored at 4 °C for four days, before precipitated was isolated by filtration. The collected solid was washed with water and dried by lyophilization to yield the corresponding 2-hydroxyacrylic acid that was used without further purification.

(*Z*)-2-hydroxy-3-(4-methoxyphenyl)acrylic acid (**14c**)

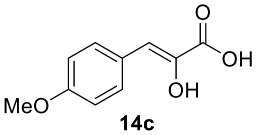

14c

Compound **14c** was synthesized from (*Z*)-4-(4-methoxybenzylidene)-2-methyloxazol-5(4*H*)-one, yielding 0.32 g of a brown solid (1.66 mmol, 73%).

^{1}H-NMR (300 MHz, DMSO-d_6): δ [ppm] = 13.02 (bs, 1H), 8.96 (bs, 1H), 7.71 (d, *J* = 8.8 Hz, 2H), 6.92 (d, *J* = 8.8 Hz, 2H), 6.37 (s, 1H), 3.76 (s, 3H).

NMR data matched those of (*Z*)-2-hydroxy-3-(4-methoxyphenyl)acrylic acid, as described above.

(*Z*)-2-hydroxy-3-(4-hydroxy-3-methoxyphenyl)acrylic acid (**14d**)

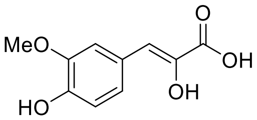

14d

Compound **14d** was synthesized from (*Z*)-2-methoxy-4-((2-methyl-5-oxooxazol-4(5*H*)-ylidene)methyl)phenyl acetate, yielding 0.40 g of a red-brown solid (1.9 mmol, 52%).

^{1}H-NMR (600 MHz, DMSO-d_6): δ [ppm] = 12.94 (vbs, 1H), 9.19 (vbs, 1H), 8.86 (bs, 1H), 7.40 (d, *J* = 2.0 Hz, 1H), 7.19 (dd, *J* = 8.3, 2.0 Hz, 1H), 6.75 (d, *J* = 8.3 Hz, 1H), 6.35 (s, 1H), 3.75 (s, 3H).

^{13}C{^{1}H}-NMR (151 MHz, DMSO-d_6): δ [ppm] = 166.6, 147.2, 146.3, 139.4, 126.5, 123.0, 115.4, 113.5, 110.7, 55.5.

HR-MS (ESI+): *m/z* calcd. for $C_{10}H_{10}O_5$ $[M+Na]^+$: 233.0421; found: 233.0425.

2.2 Synthesis of 30a–c and 30k

General procedure:

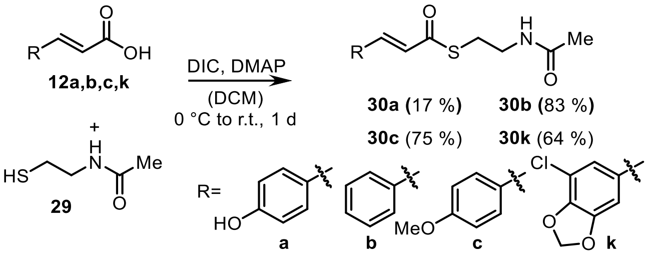

The corresponding acid (1 mmol, 1.0 eq.), SNAC (1 mmol, 1.0 eq.), along with *N*,*N'*-diisopropylcarbodiimide (DIC, 1.1 mmol, 1.1 eq.) and 4-dimethylaminopyridine (DMAP, 0.01 mmol, 1 mol %), were suspended in DCM and reacted overnight. Following the reaction, aqueous hydrochloric acid (HCl) and additional DCM were added to extract the product. The organic layer, which contained the desired product, was washed with saturated NaCl solution to remove residual water. The solvent was then removed under reduced pressure, and the crude product was purified by preparative HPLC.

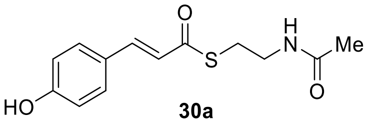

30a

Product **30a** was synthesized from 1**2a** and obtained as 45 mg of a white solid (0.17 mmol, 17%).

^{1}H-NMR (600 MHz, DMSO-*d*$_6$) : δ [ppm] = 10.12 (s, 1H), 8.10 (t, *J* = 5.7 Hz, 1H), 7.62 – 7.59 (m, 2H), 7.51 (d, *J* = 15.7 Hz, 1H), 6.81 – 6.79 (m, 2H), 6.78 (d, *J* = 11.2 Hz, 1H), 3.23 – 3.19 (m, 2H), 3.00 (t, *J* = 6.9 Hz, 2H), 1.80 (s, 3H).
^{13}C{^{1}H}-NMR (151 MHz, DMSO-*d*$_6$): δ [ppm] = 188.5, 169.3, 160.3, 140.9, 131.0 (2C), 124.7, 121.4, 115.9 (2C), 38.4, 28.0, 22.6.
HR-MS (ESI+): *m/z* calcd. for $C_{13}H_{15}NO_3S$ [M+H]$^+$: 266.0846; found: 266.0845.

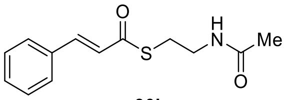

30b

Product **30b** was synthesized from **12b** and obtained as 206 mg of a white solid (0.83 mmol, 83%).

^{1}H-NMR (600 MHz, DMSO-*d*$_6$): δ [ppm] = 8.11 (t, *J* = 5.8 Hz, 1H), 7.77 (dd, *J* = 7.6, 2.1 Hz, 2H), 7.60 (d, *J* = 15.9 Hz, 1H), 7.47 – 7.41 (m, 3H), 7.03 (d, *J* = 15.9 Hz, 1H), 3.23 (q, *J* = 6.2 Hz, 2H), 3.04 (t, *J* = 6.8 Hz, 2H), 1.80 (s, 3H).
^{13}C{^{1}H}-NMR (151 MHz, DMSO-*d*$_6$): δ [ppm] = 188.9, 169.3, 140.5, 133.7, 130.8, 129.0 (2C), 128.8 (2C), 125.0, 28.2, 23.3, 22.6.
HR-MS (ESI+): *m/z* calcd. for $C_{13}H_{15}NO_2S$ [M+H]$^+$: 250.0897; found: 250.0897.

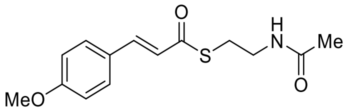

30c

Product **30c** was synthesized from **12c** and obtained as 209 mg of a white solid (0.75 mmol, 75%).

^{1}H-NMR (600 MHz, DMSO-*d*$_6$): δ [ppm] = 8.10 (t, *J* = 5.2 Hz, 1H), 7.73 (d, *J* = 8.8 Hz, 2H), 7.56 (d, *J* = 15.8 Hz, 1H), 6.99 (d, *J* = 8.8 Hz, 2H), 6.88 (d, *J* = 15.8 Hz, 1H), 3.80 (s, 3H), 3.22 (q, *J* = 6.2 Hz, 2H), 3.01 (t, *J* = 6.8 Hz, 2H), 1.80 (s, 3H).

^{13}C{^{1}H}-NMR (151 MHz, DMSO-d_6): δ [ppm] = 188.7, 169.3, 161.5, 140.5, 130.7 (2C), 126.3, 122.5, 114.5 (2C), 55.4, 38.3, 28.1, 22.6.
HR-MS (ESI+): *m/z* calcd. for $C_{14}H_{17}NO_3S$ $[M+H]^+$: 280.1002; found: 280.1001.

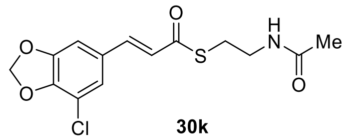

30k

Product **30k** was synthesized from **12k** and obtained as 209 mg of a white solid (0.64 mmol, 64%).

^{1}H-NMR (600 MHz, DMSO-d_6): δ [ppm] = 8.10 (t, *J* = 5.7 Hz, 1H), 7.51 – 7.43 (m, 3H), 6.99 (d, *J* = 15.8 Hz, 1H), 6.20 (s, 2H), 3.22 (q, *J* = 6.8 Hz, 2H), 3.02 (t, *J* = 6.8 Hz, 2H), 1.80 (s, 3H).
^{13}C{^{1}H}-NMR (151 MHz, DMSO-d_6): δ [ppm] = 188.7, 169.3, 149.1, 146.0, 139.1, 129.4, 125.2, 124.4, 112.7, 106.1, 102.8, 38.2, 28.2, 22.6.
HR-MS (ESI+): *m/z* calcd. for $C_{14}H_{14}ClNO_4S$ $[M+H]^+$: 328.0405; found: 328.0404.

3. Enzymatic Assays

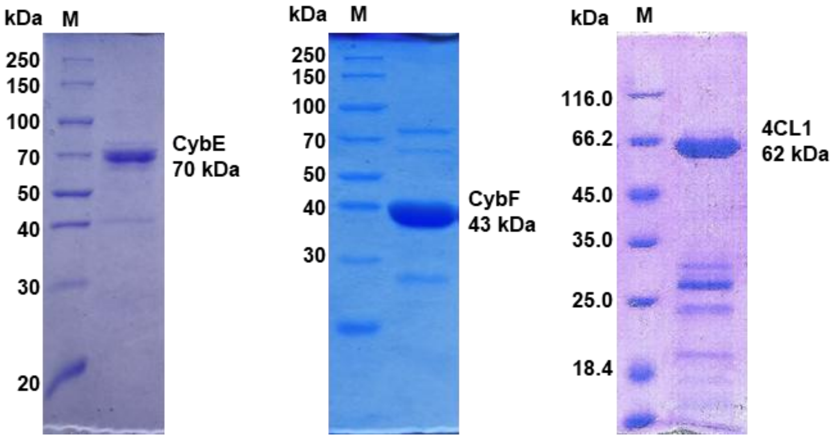

Figure S1. SDS-PAGE analysis of purified recombinant CybE, CybF, and At4CL1 used for *in vitro* assays.

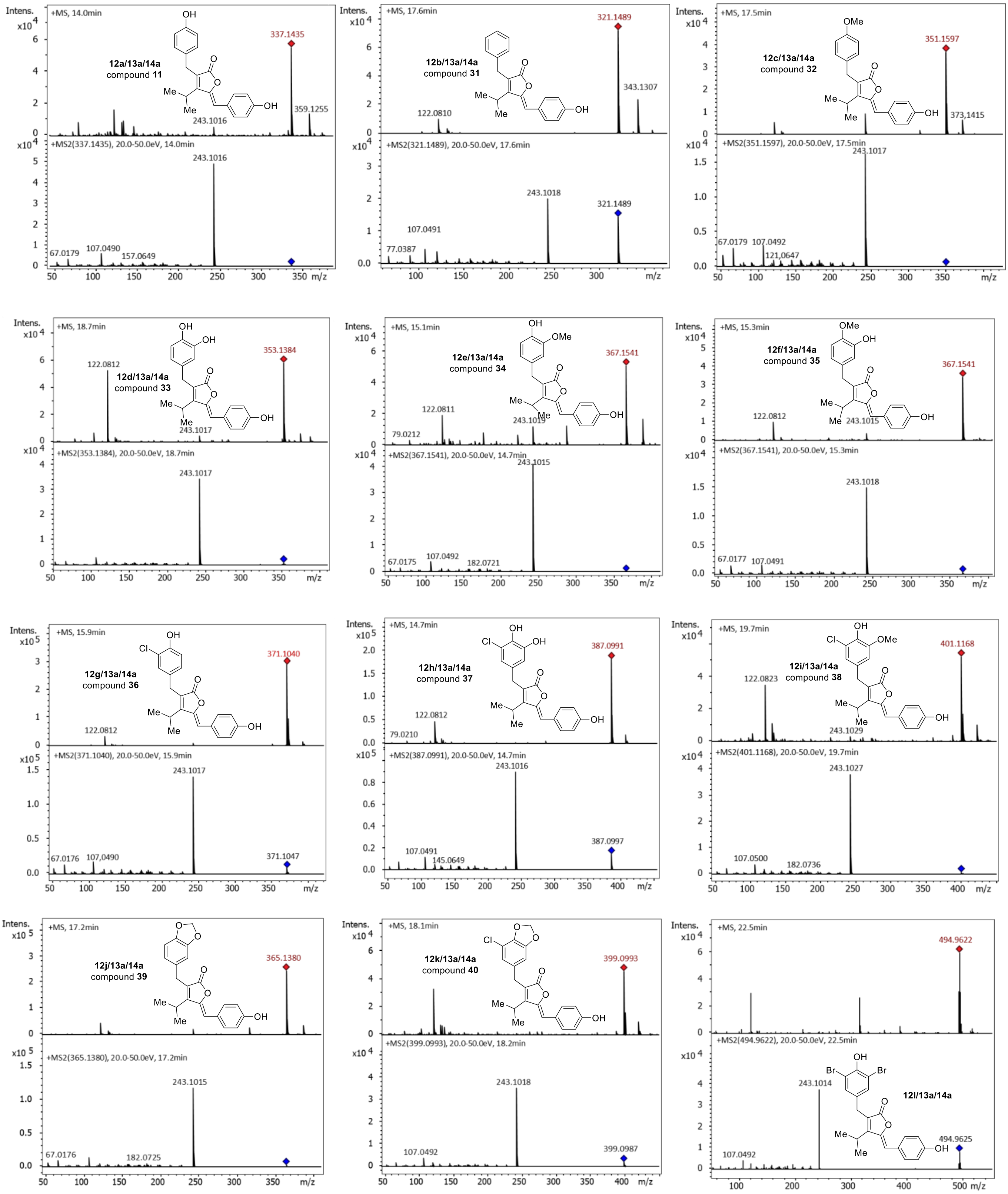

Figure S2. HRLC-MS/MS analysis of products arising from reactions of **12a–l** combined with **13a**/**14a**.

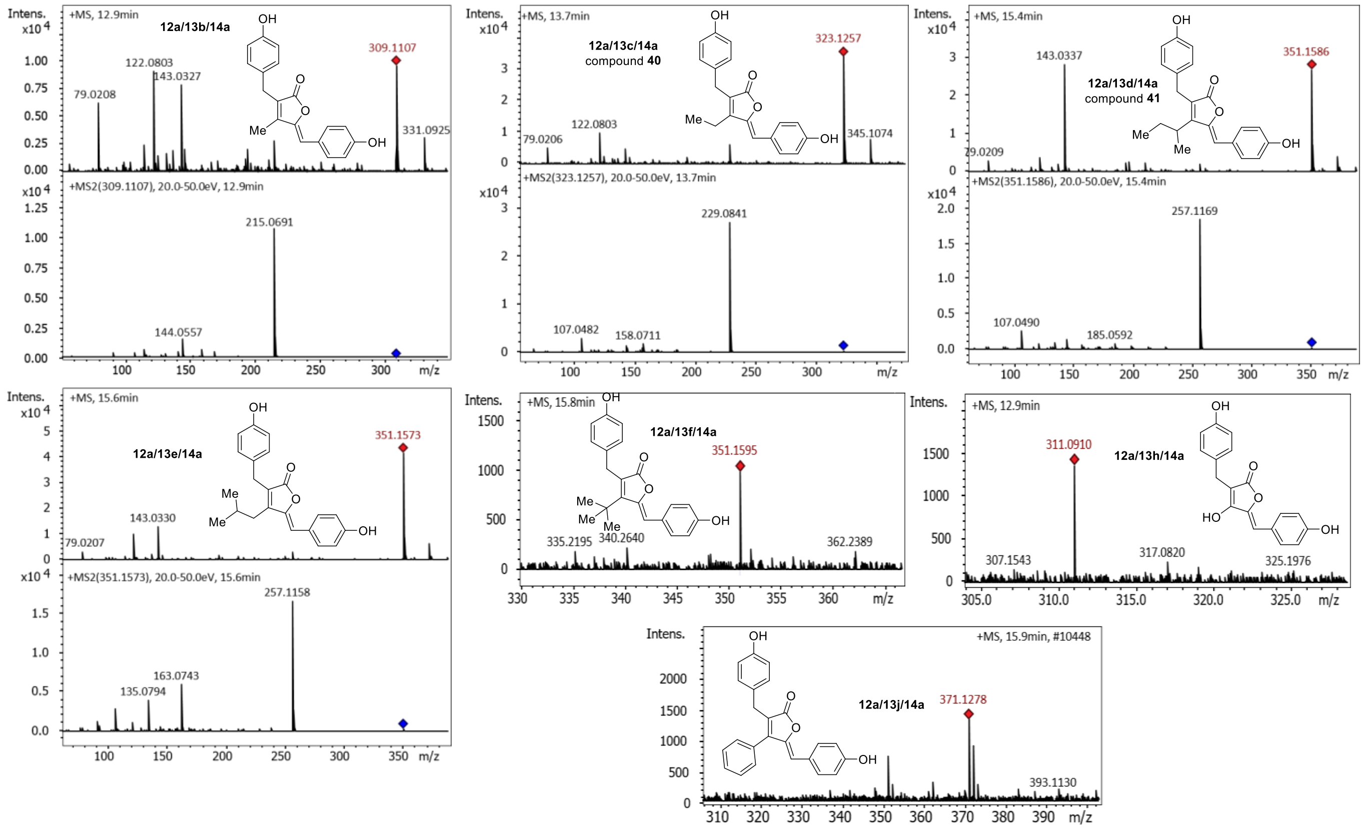

Figure S3. HRLC-MS/MS analysis of products arising from reactions of **13b–f,h,j** combined with **12a**/**14a**.

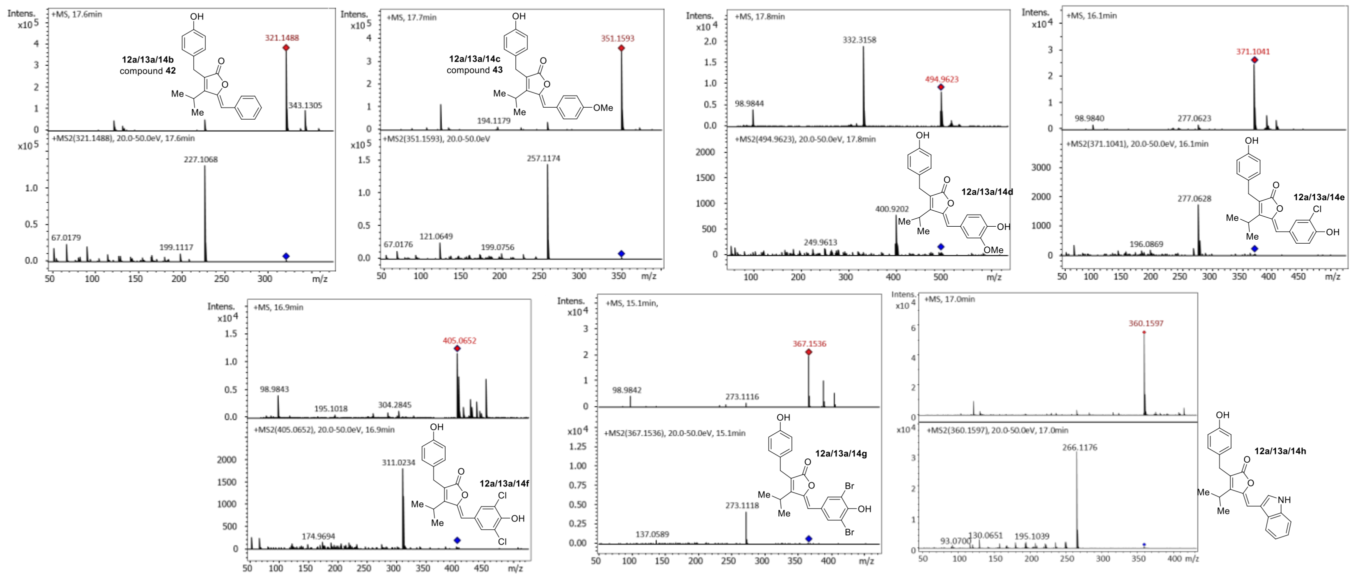

Figure S4. HRLC-MS/MS analysis of products arising from reactions of **14b–h** combined with **12a**/**13a**.

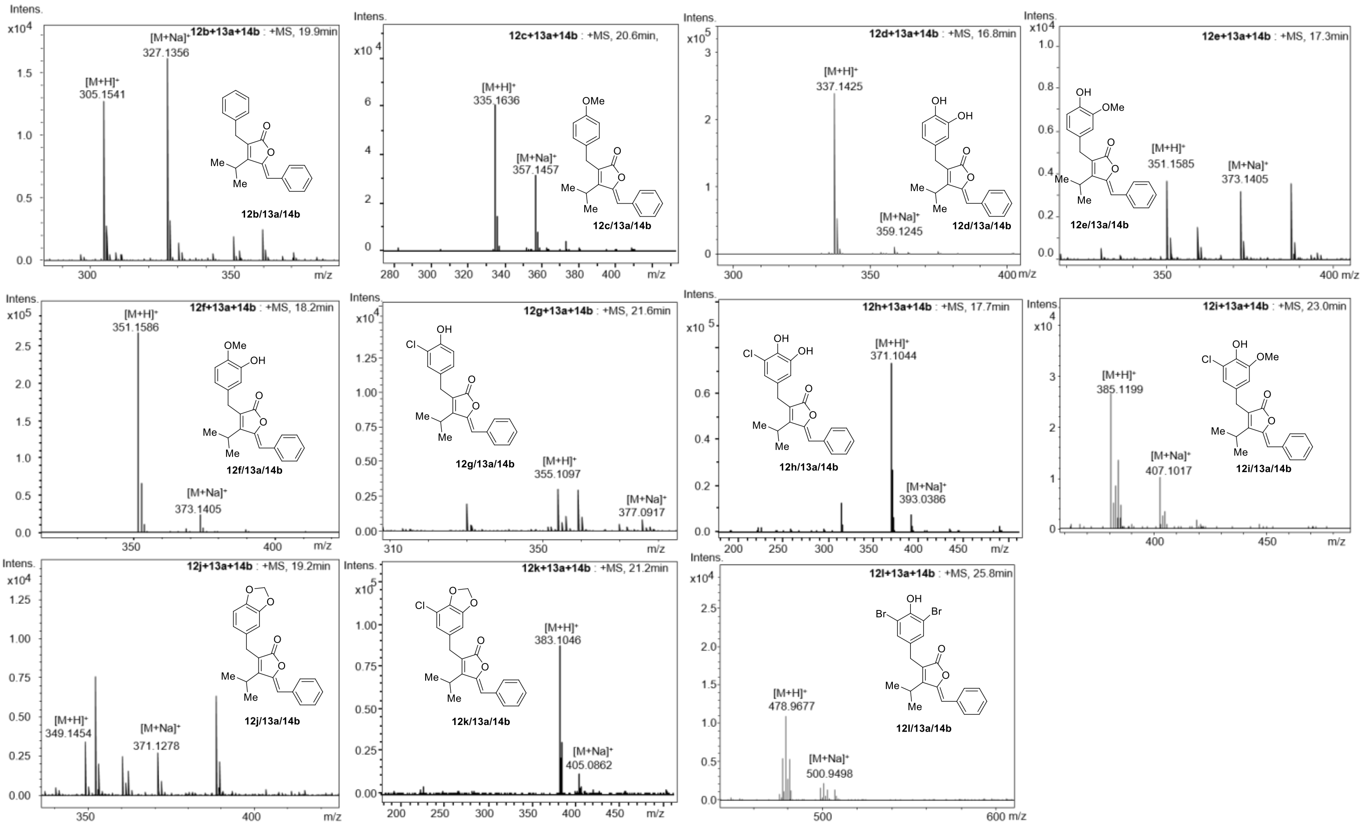

Figure S5. HRLC-MS/MS analysis of products arising from reactions of **12b–l** combined with **13a/14b**.

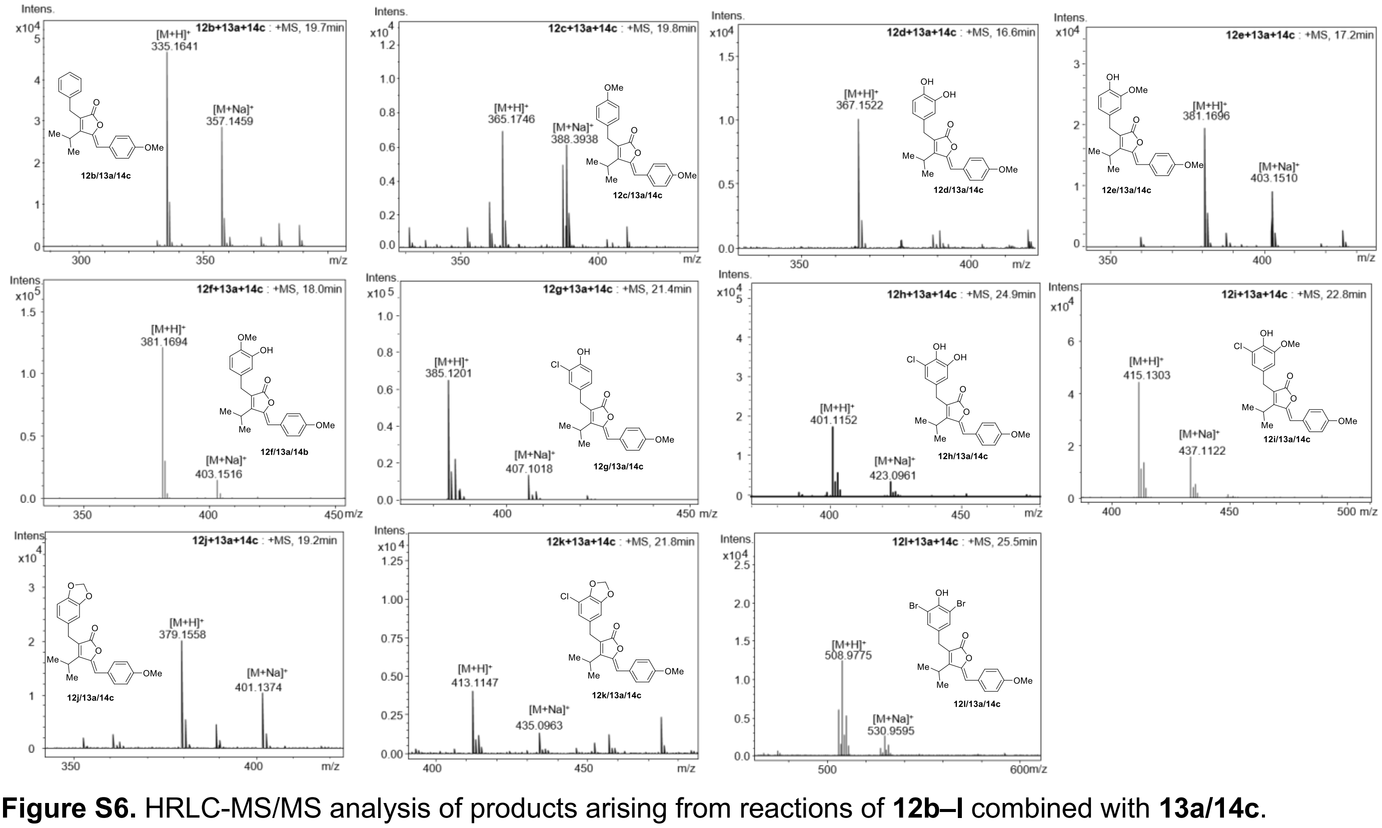

Figure S6. HRLC-MS/MS analysis of products arising from reactions of **12b–l** combined with **13a/14c**.

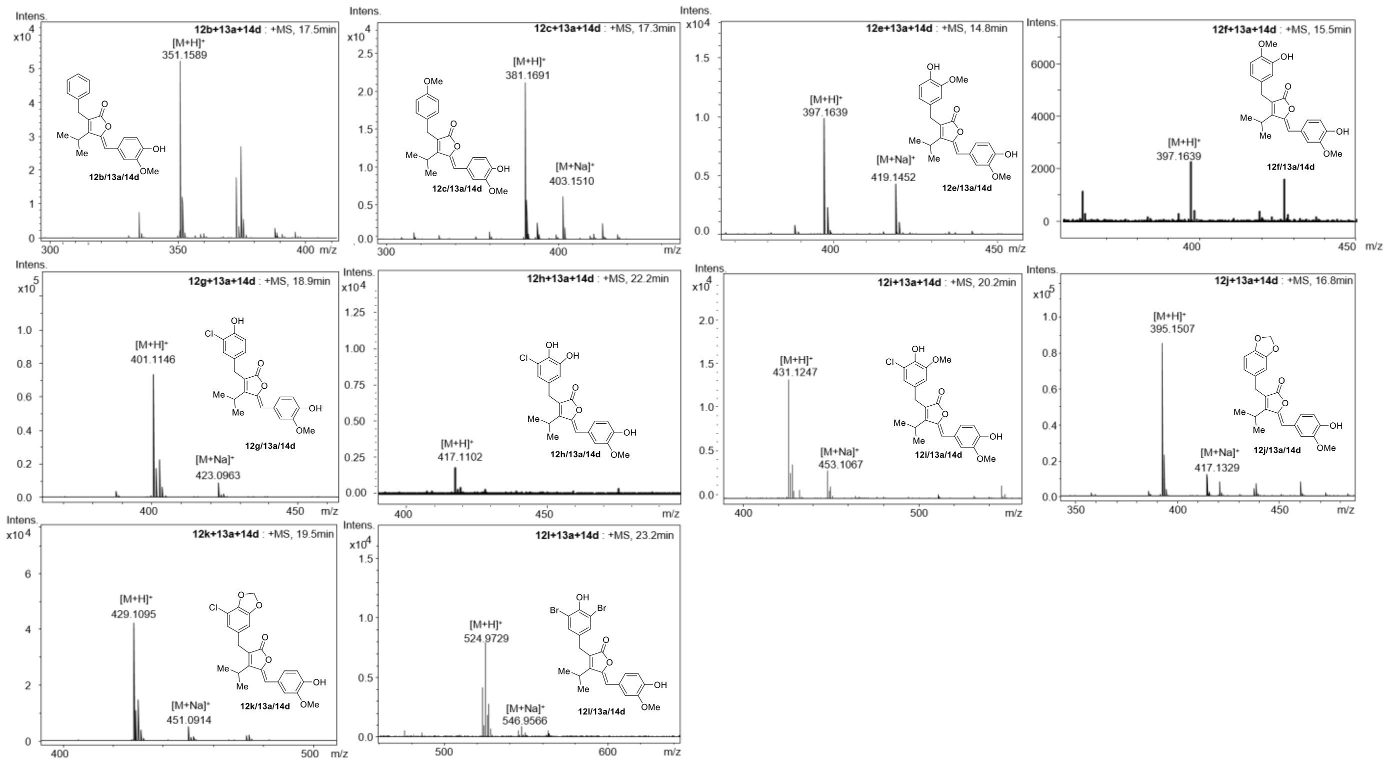

Figure S7. HRLC-MS/MS analysis of products arising from reactions of **12b,c,e–l** combined with **13a/14d**.

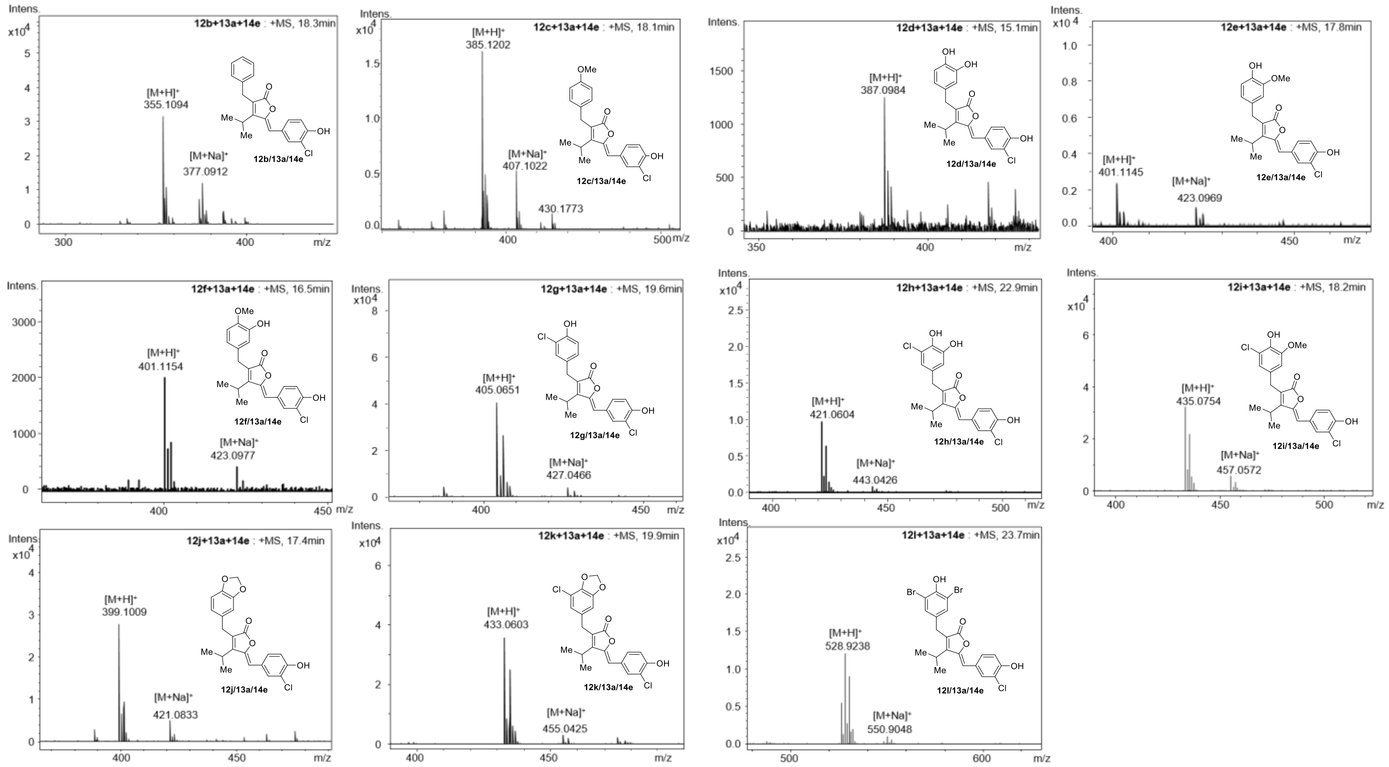

Figure S8. HRLC-MS/MS analysis of products arising from reactions of **12b–l** combined with **13a/14e**.

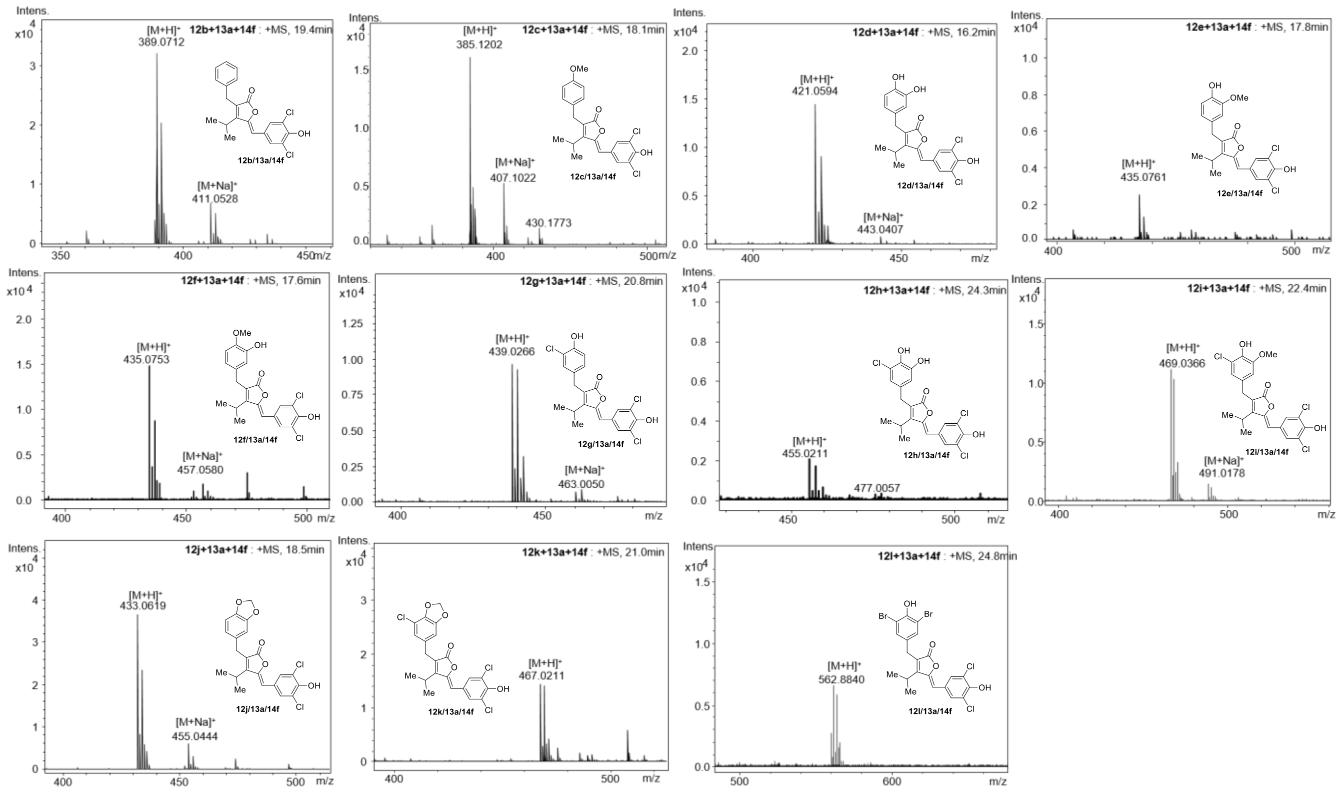

Figure S9. HRLC-MS/MS analysis of products arising from reactions of **12b–l** combined with **13a/14f**.

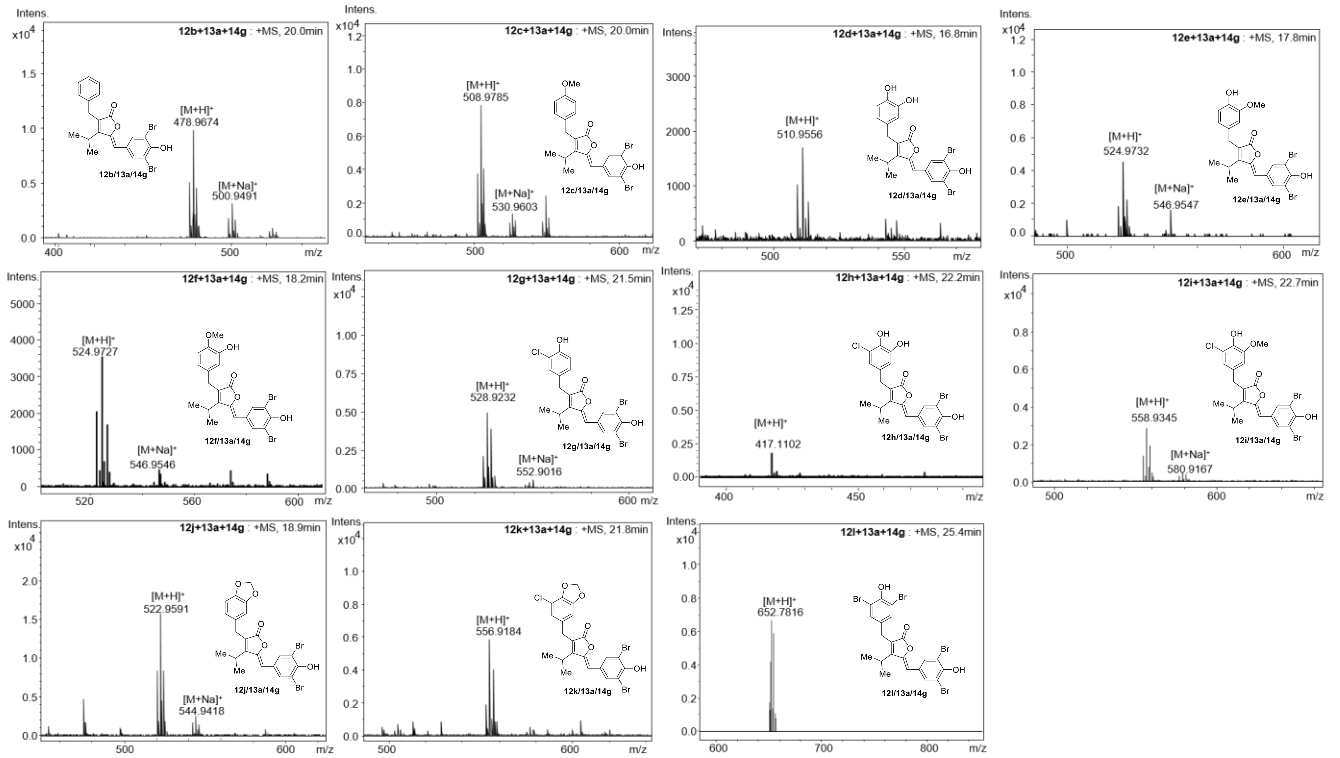

Figure S10. HRLC-MS/MS analysis of products arising from reactions of **12b–l** combined with **13a/14g**.

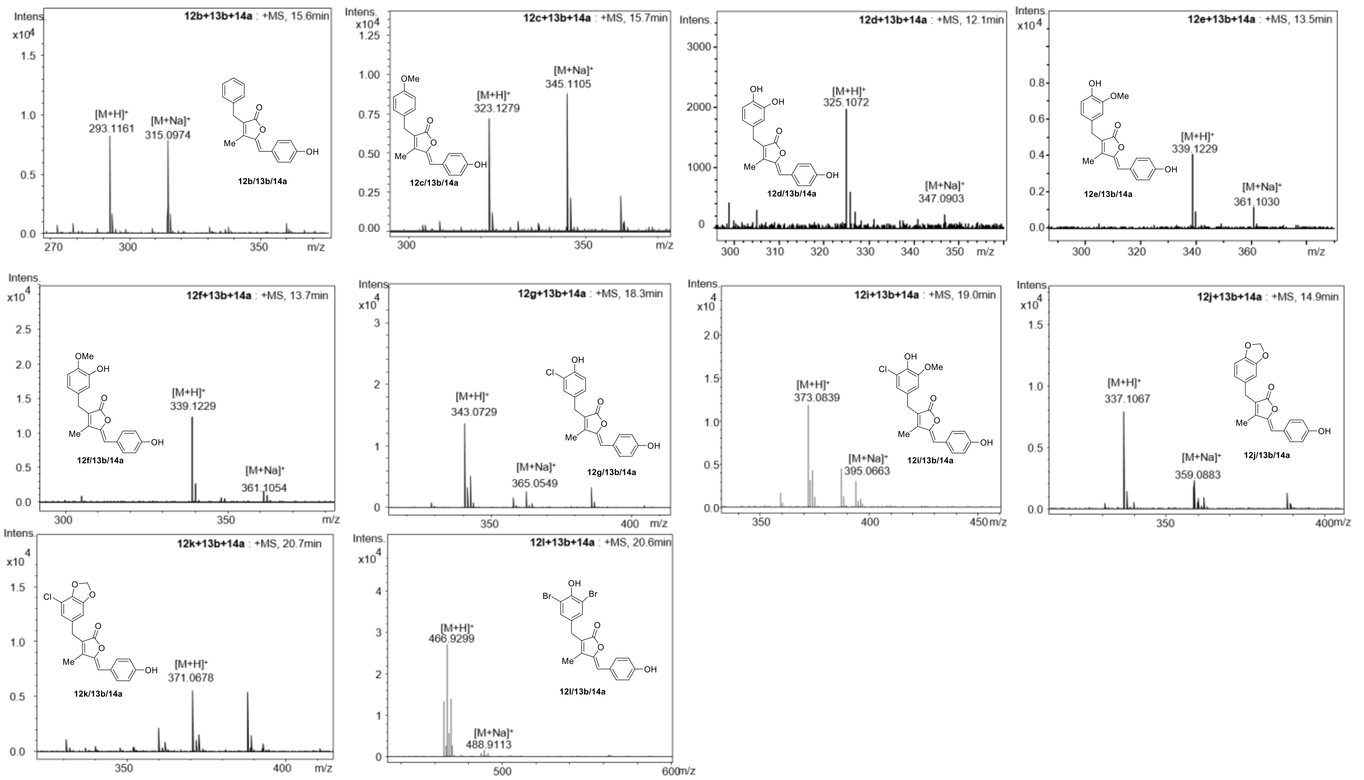

Figure S11. HRLC-MS/MS analysis of products arising from reactions of **12b-g,i–l** combined with **13b/14a**.

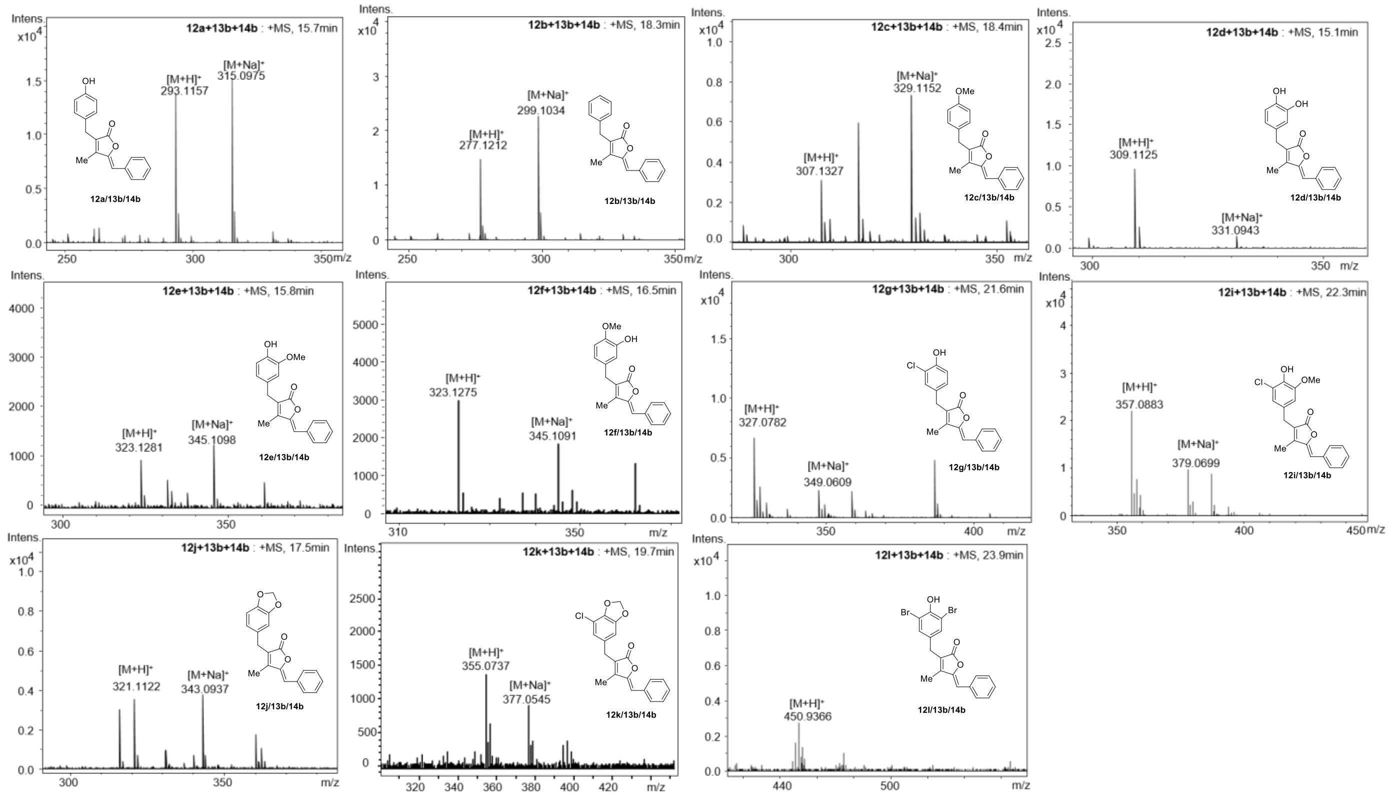

Figure S12. HRLC-MS/MS analysis of products arising from reactions of **12b-g,i–l** combined with **13b/14b**.

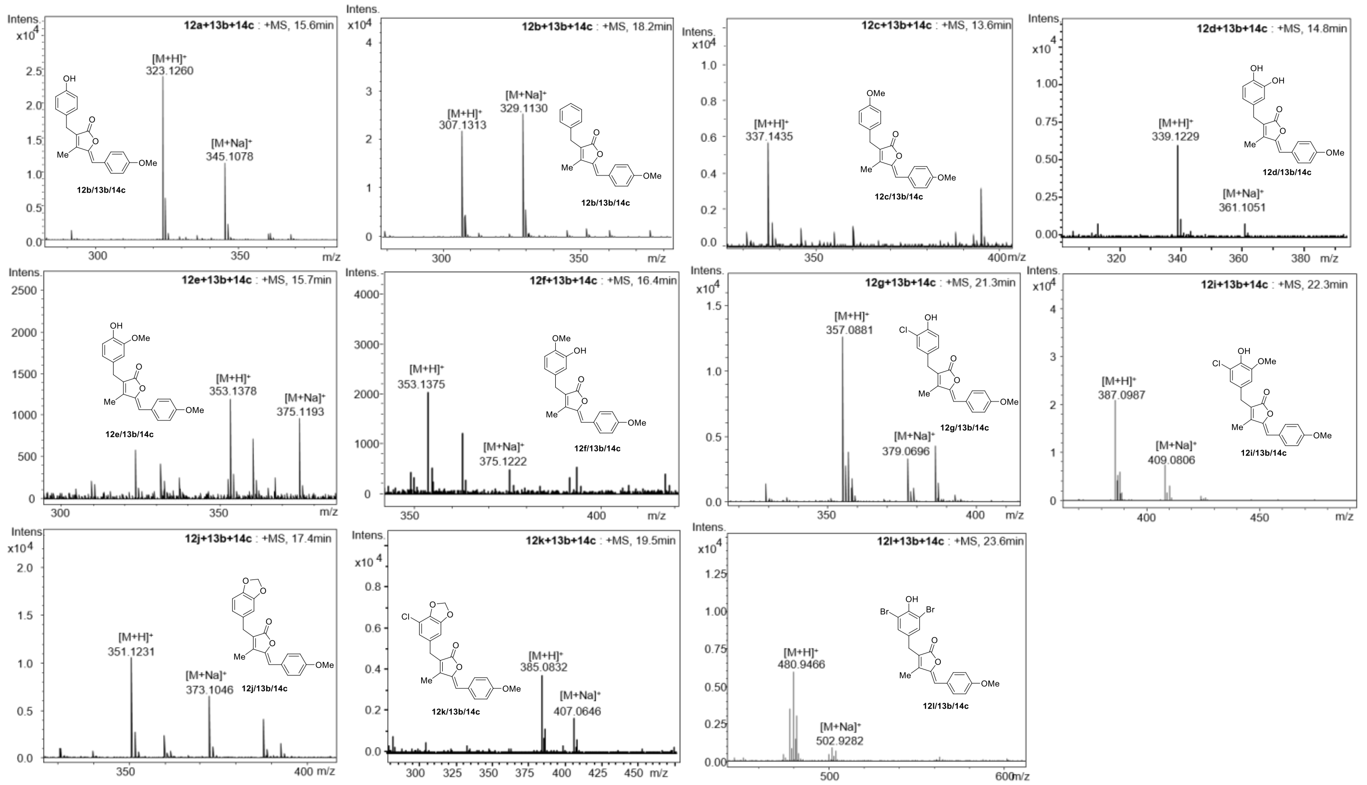

Figure S13. HRLC-MS/MS analysis of products arising from reactions of **12b-g,i–l** combined with **13b/14c**.

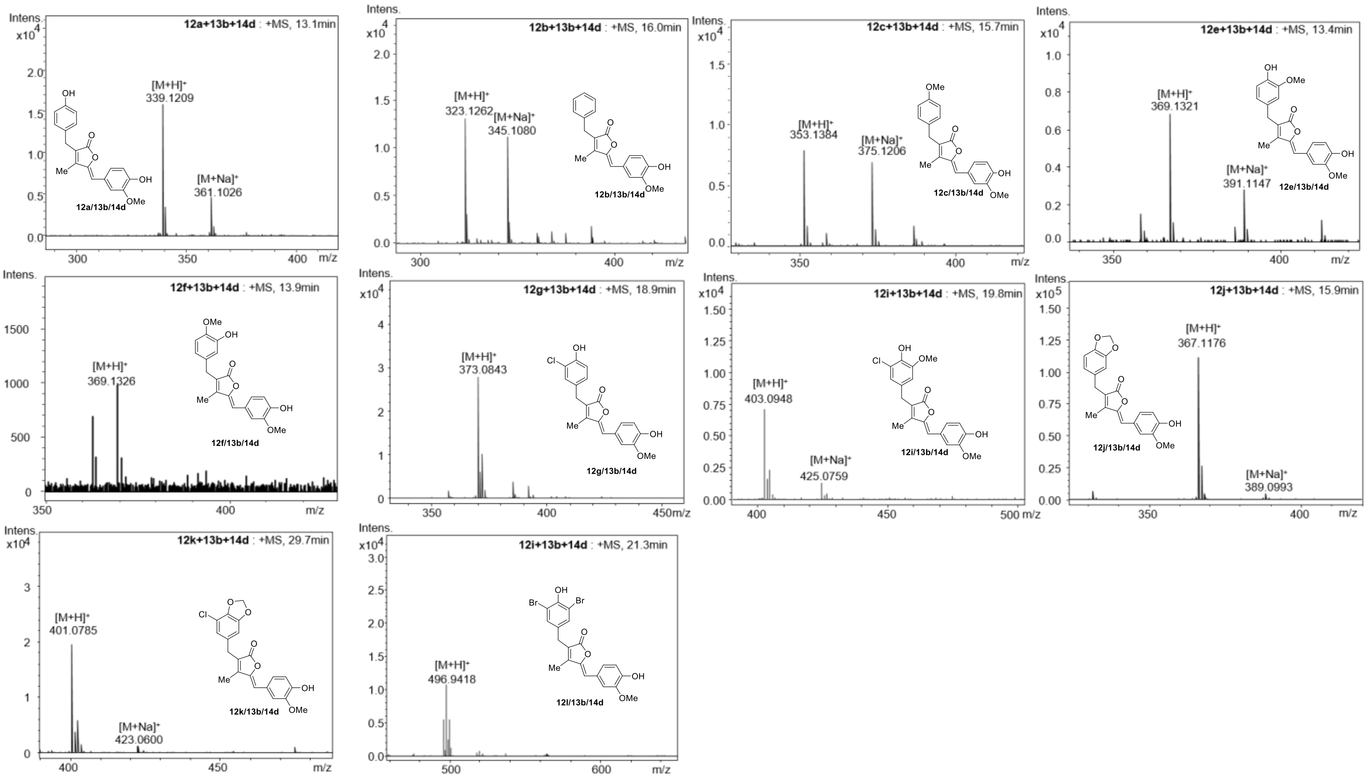

Figure S14. HRLC-MS/MS analysis of products arising from reactions of **12a-c,e-g,i–l** combined with **13b/14d**.

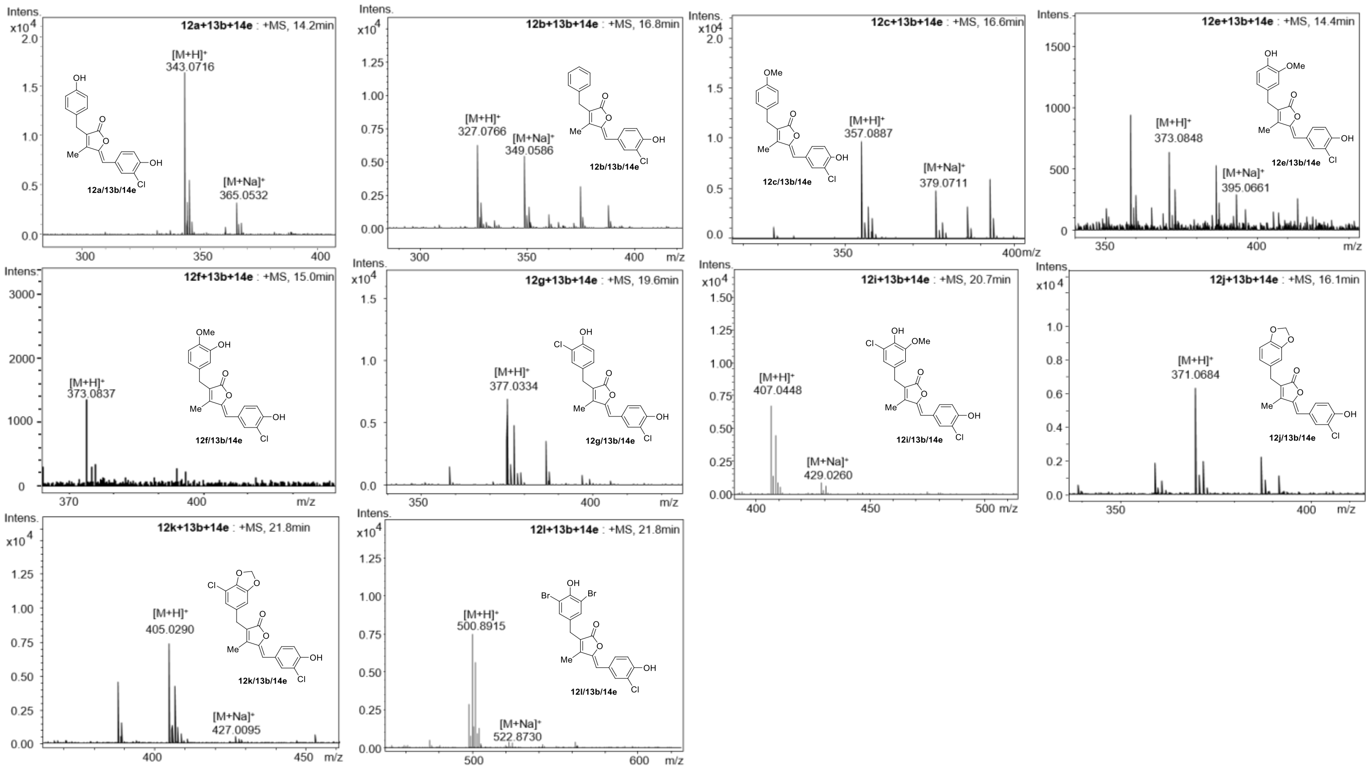

Figure S15. HRLC-MS/MS analysis of products arising from reactions of **12a-c,e-g,i–l** combined with **13b/14e**.

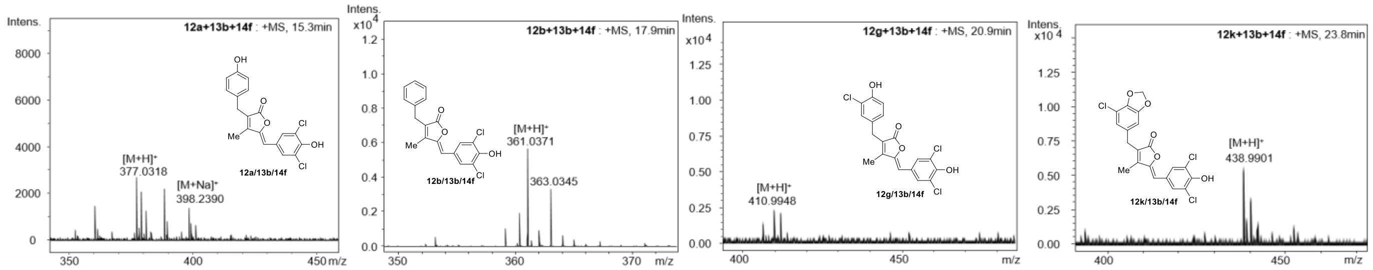

Figure S16. HRLC-MS/MS analysis of products arising from reactions of **12a,b,g,k** combined with **13b/14f**.

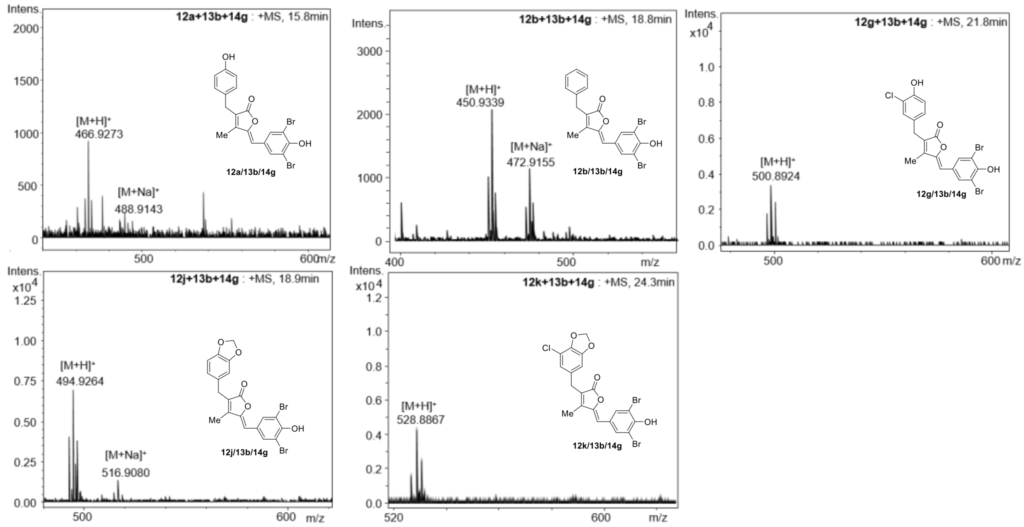

Figure S17. HRLC-MS/MS analysis of products arising from reactions of **12a,b,g,j,k** combined with **13b/14f**.

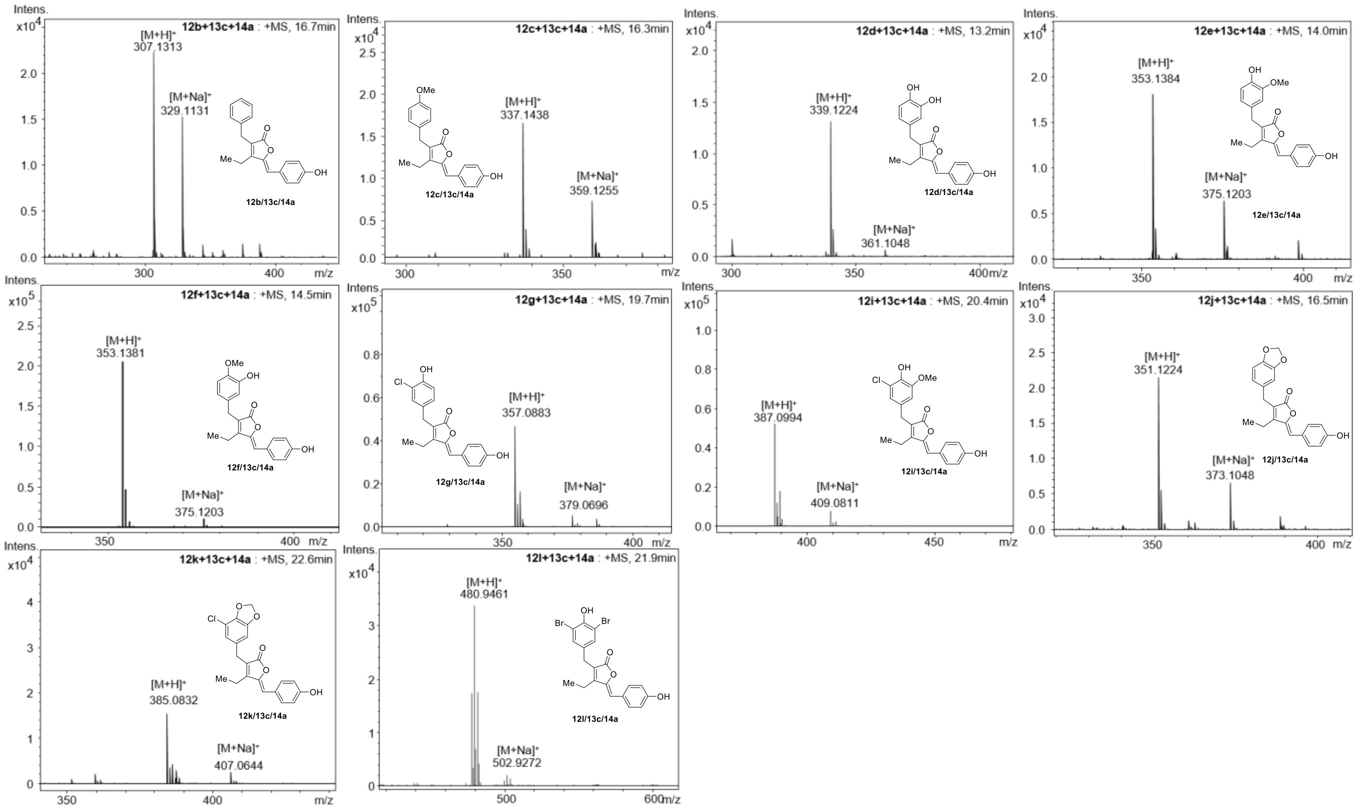

Figure S18. HRLC-MS/MS analysis of products arising from reactions of **12b-g,i–l** combined with **13c/14a**.

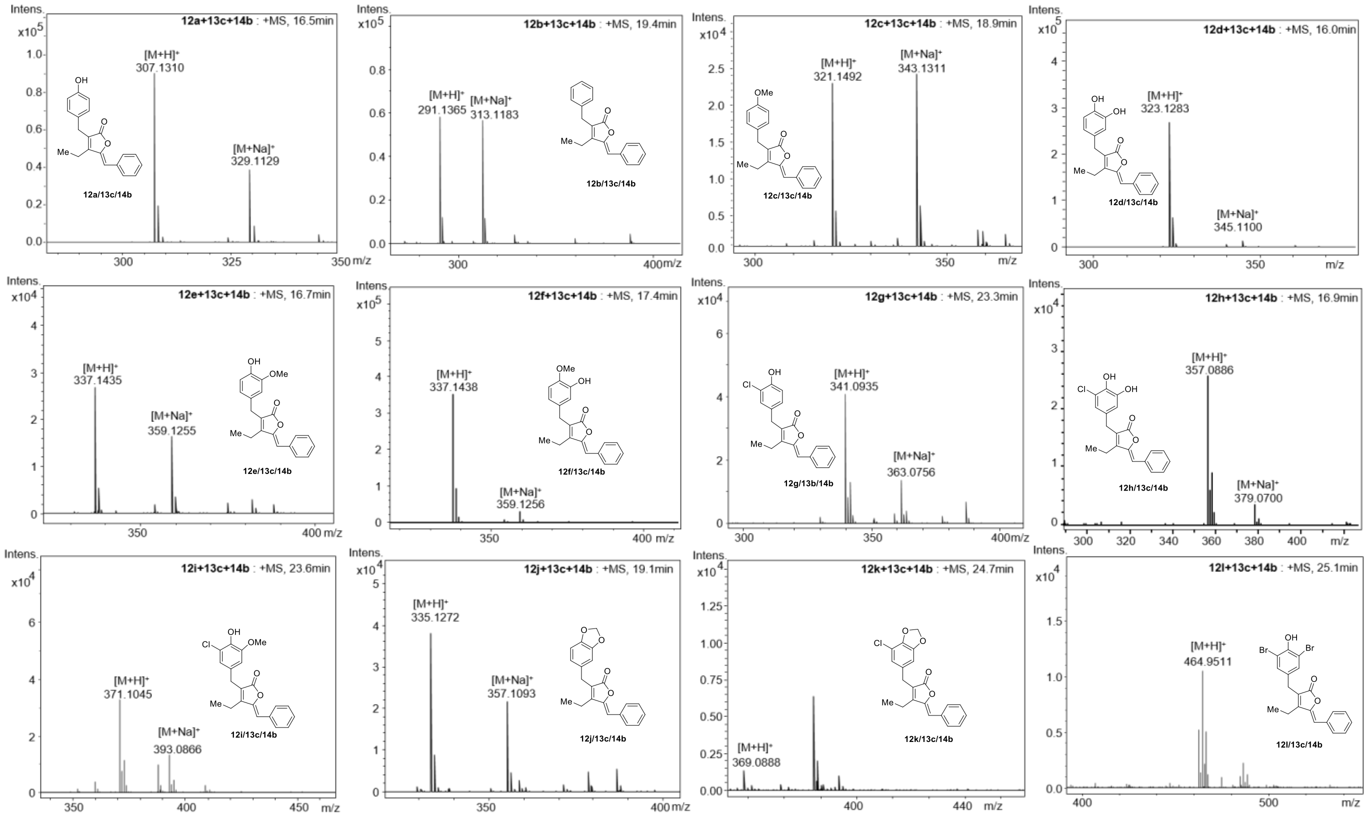

Figure S19. HRLC-MS/MS analysis of products arising from reactions of **12a–l** combined with **13c/14b**.

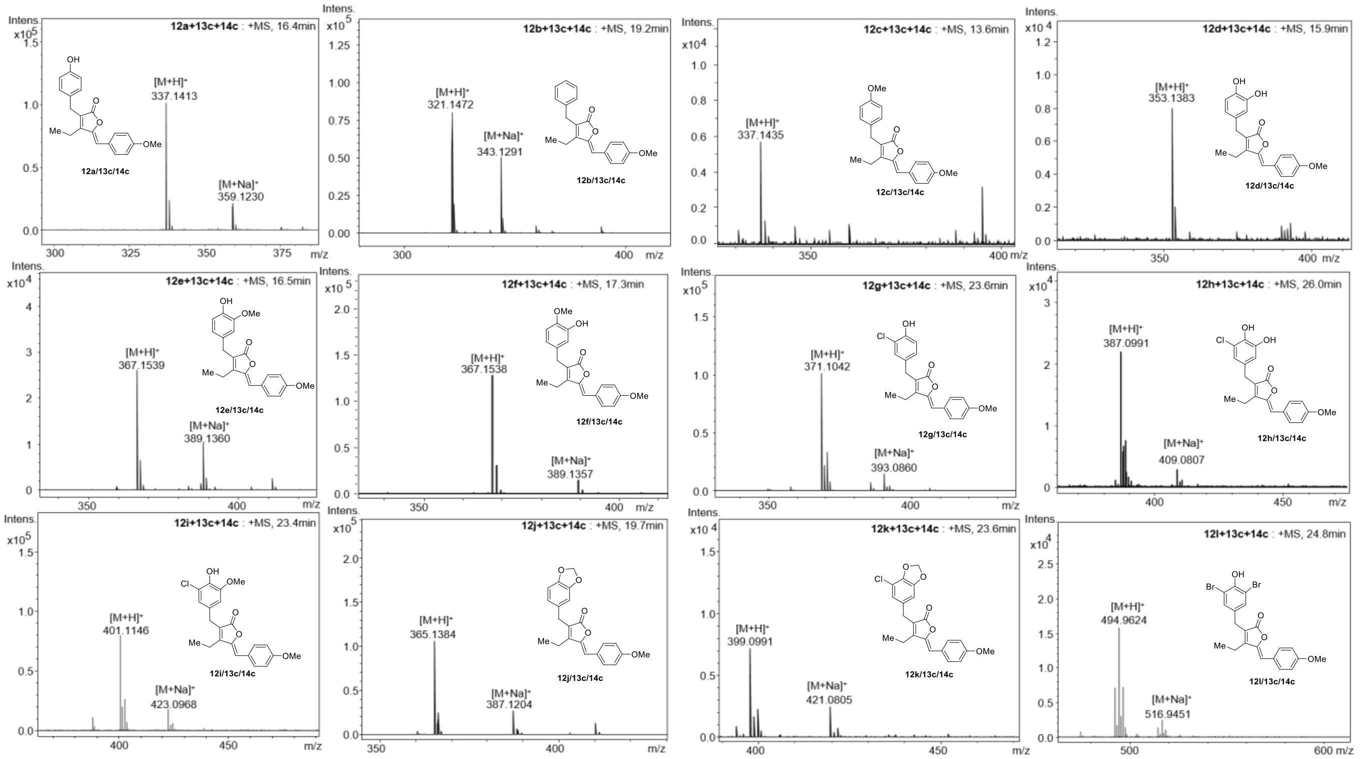

Figure S20. HRLC-MS/MS analysis of products arising from reactions of **12a–l** combined with **13c/14c**.

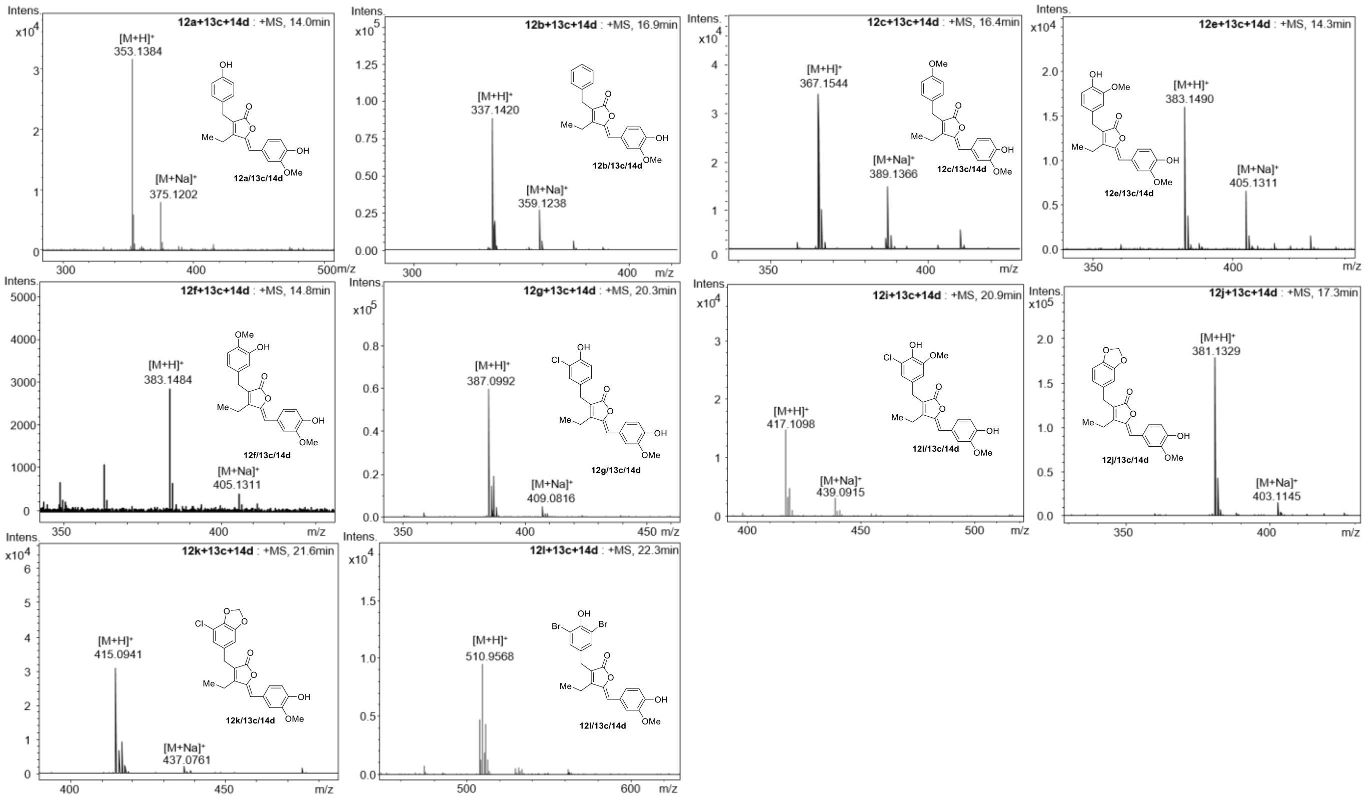

Figure S21. HRLC-MS/MS analysis of products arising from reactions of **12a–c,e-g,i–l** combined with **13c/14d**.

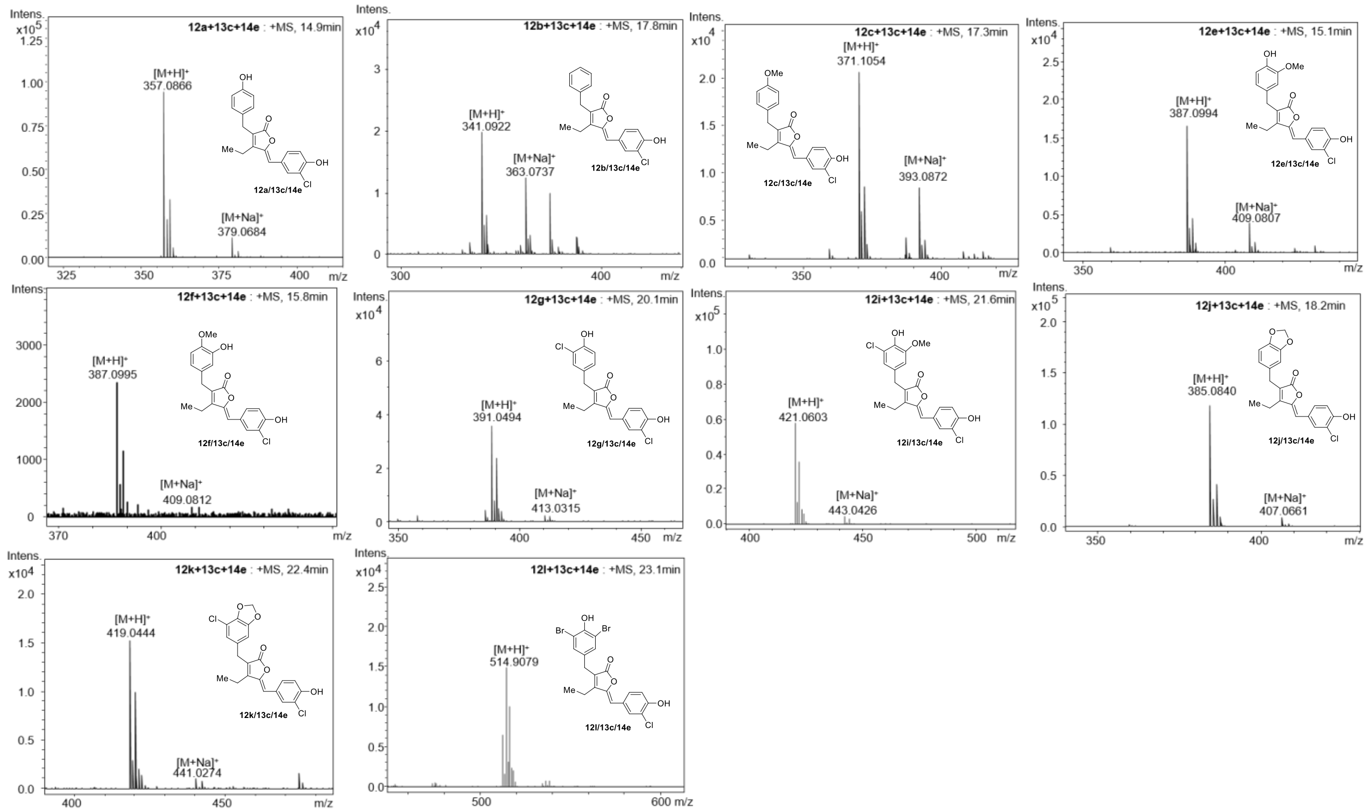

Figure S22. HRLC-MS/MS analysis of products arising from reactions of **12a–c,e-g,i–l** combined with **13c/14e**.

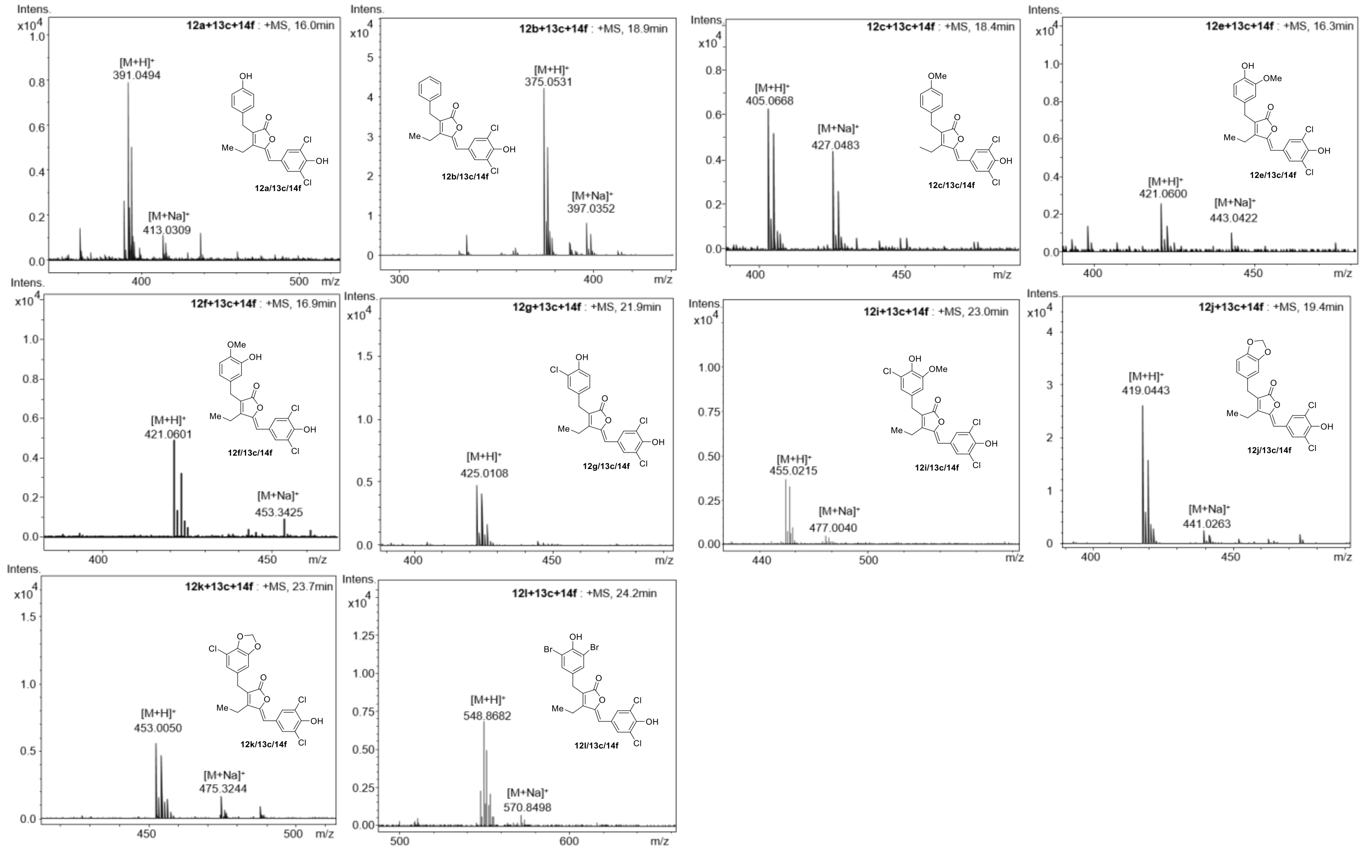

Figure S23. HRLC-MS/MS analysis of products arising from reactions of **12a–c,e-g,i–l** combined with **13c/14f**.

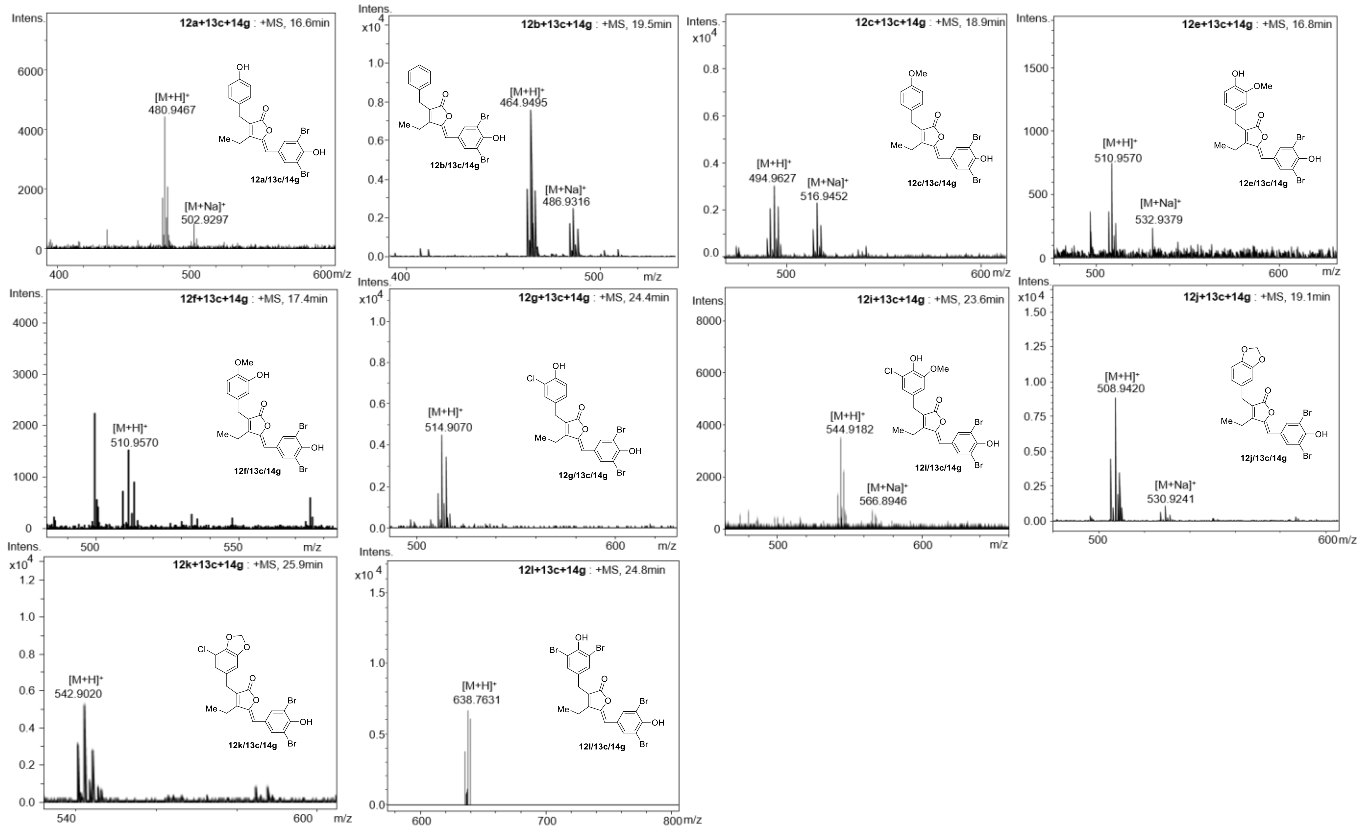

Figure S24. HRLC-MS/MS analysis of products arising from reactions of **12a–c,e-g,i–l** combined with **13c/14g.**

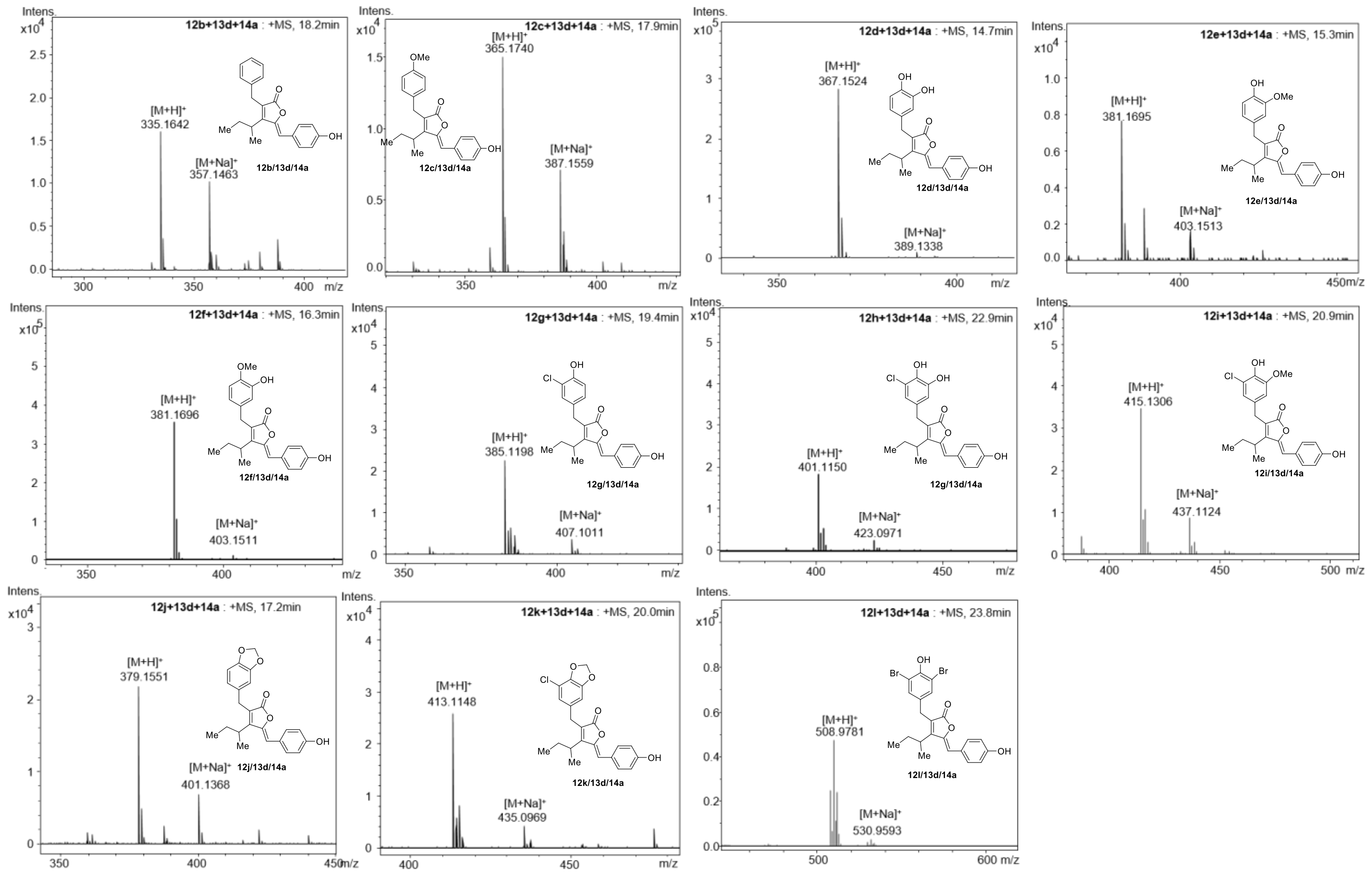

Figure S25. HRLC-MS/MS analysis of products arising from reactions of **12b–l** combined with **13d/14a**.

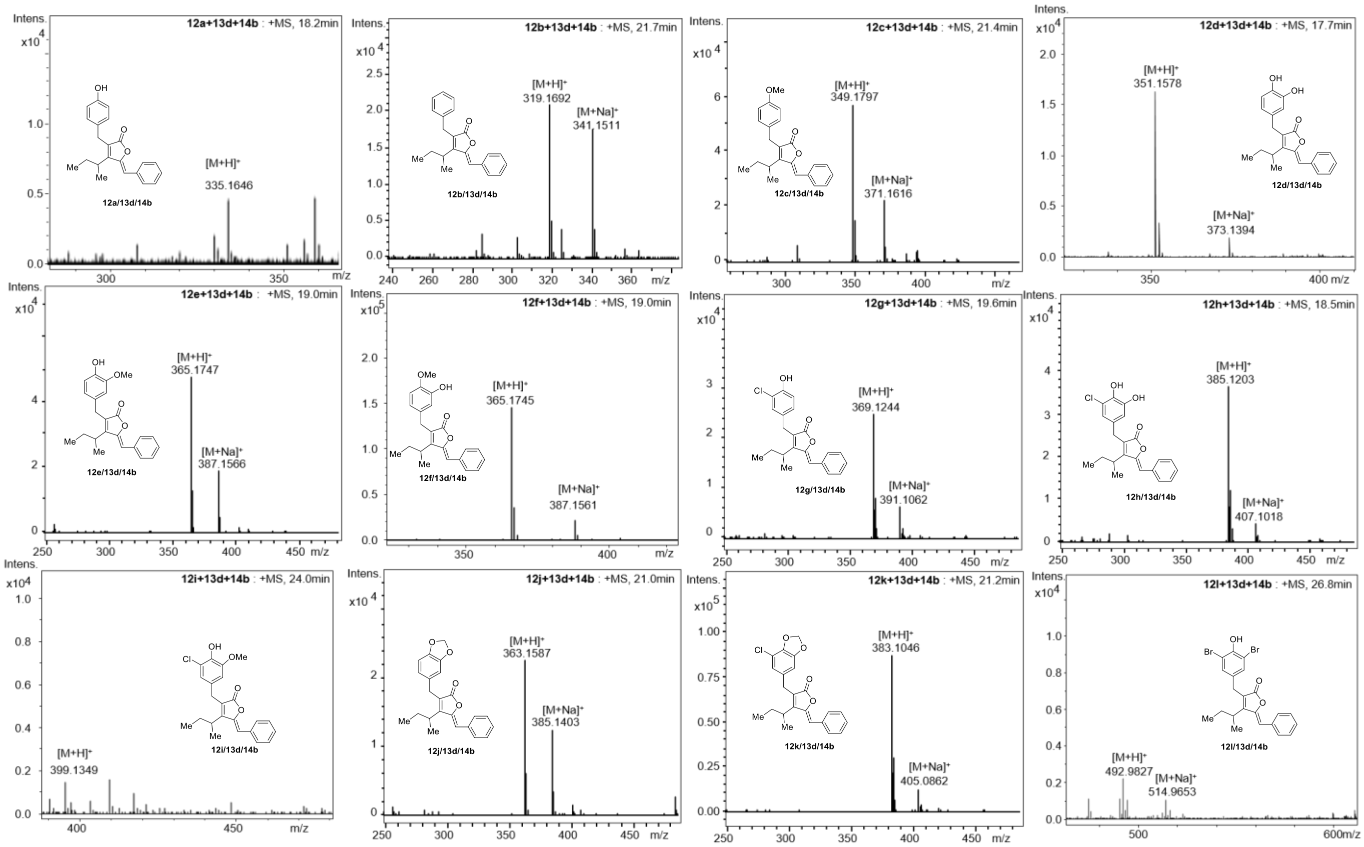

Figure S26. HRLC-MS/MS analysis of products arising from reactions of **12a–l** combined with **13d/14b**.

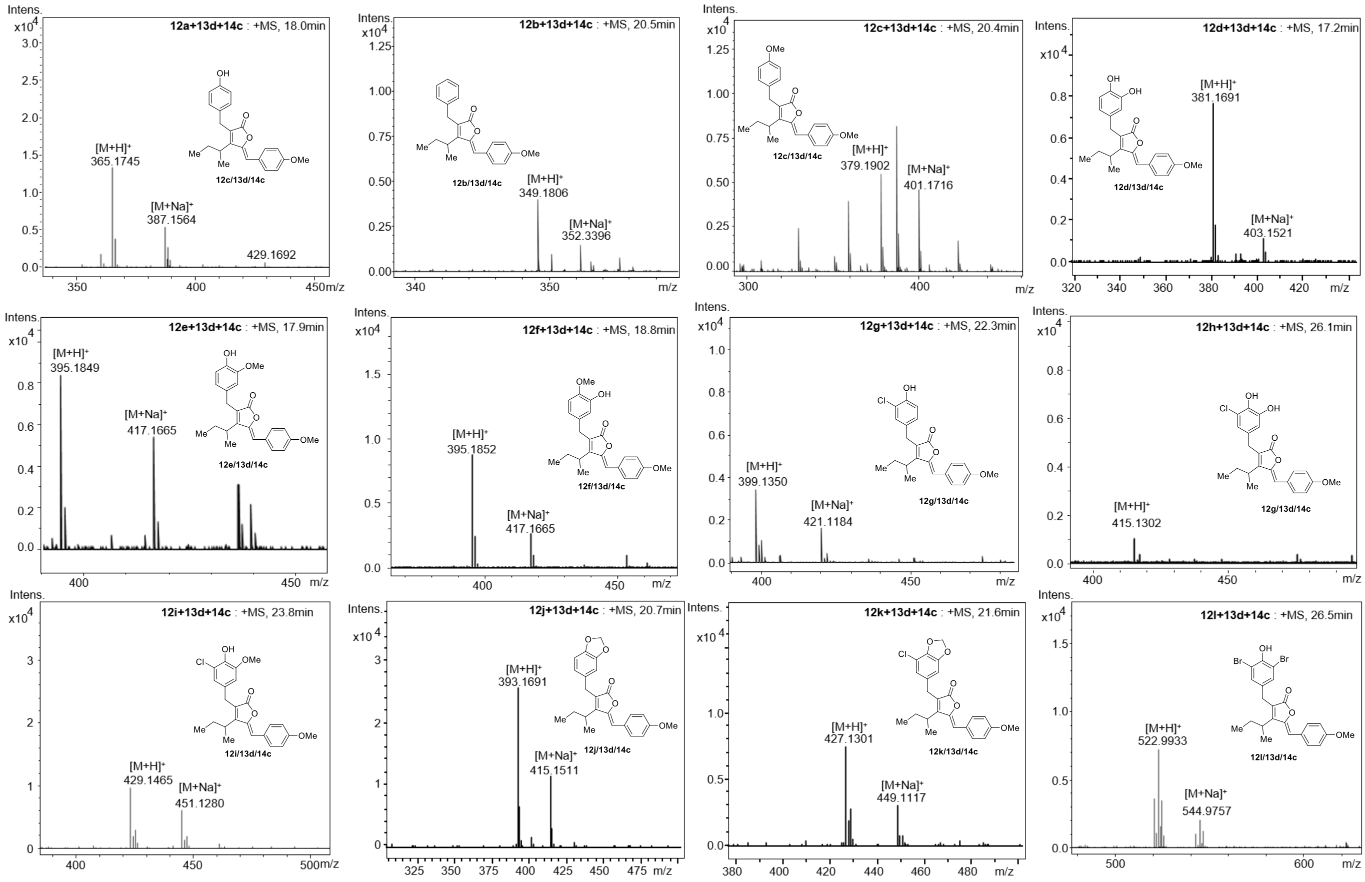

Figure S27. HRLC-MS/MS analysis of products arising from reactions of **12b–l** combined with **13d/14c**.

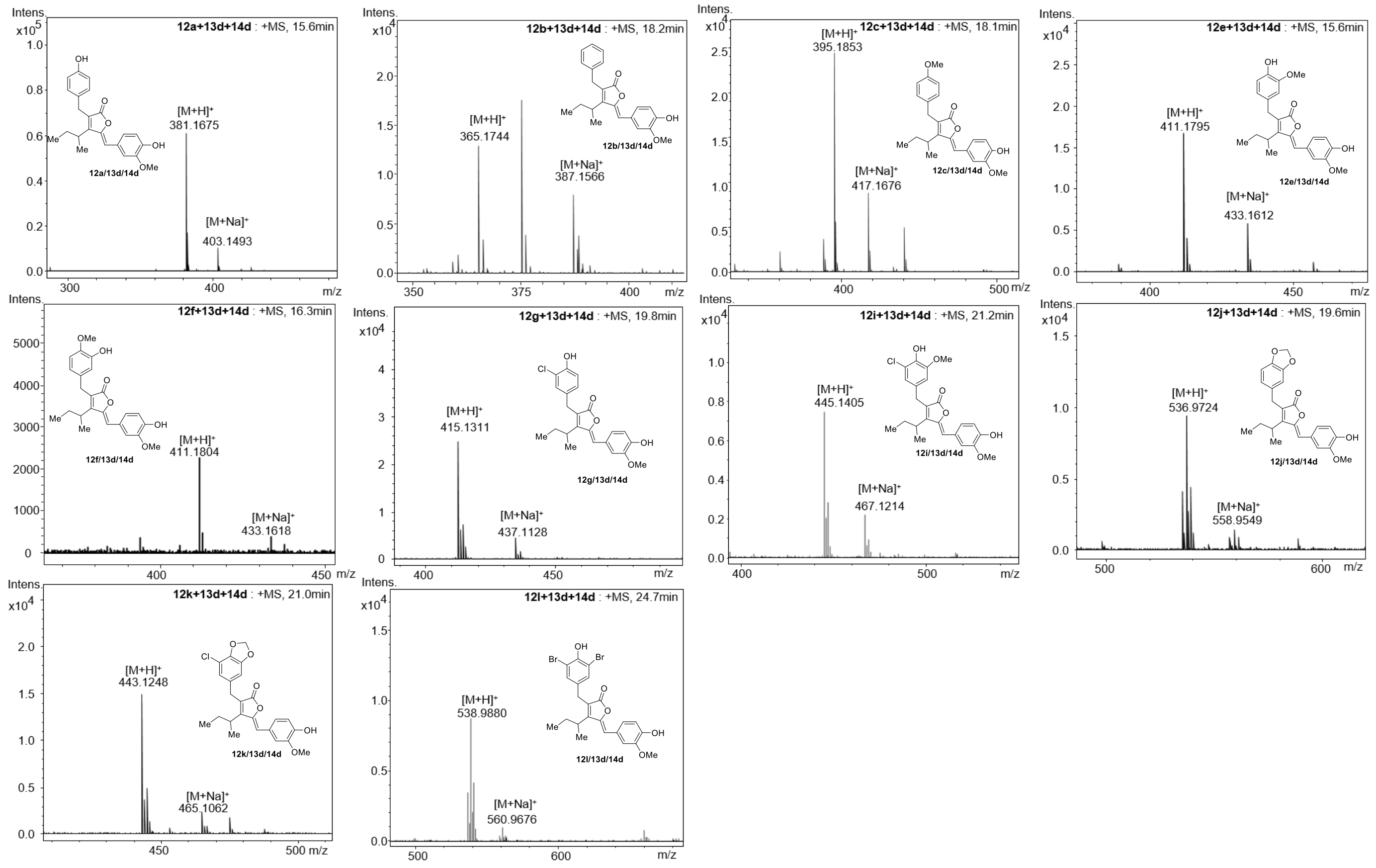

Figure S28. HRLC-MS/MS analysis of products arising from reactions of **12a–c,e-g,i–l** combined with **13d/14d**.

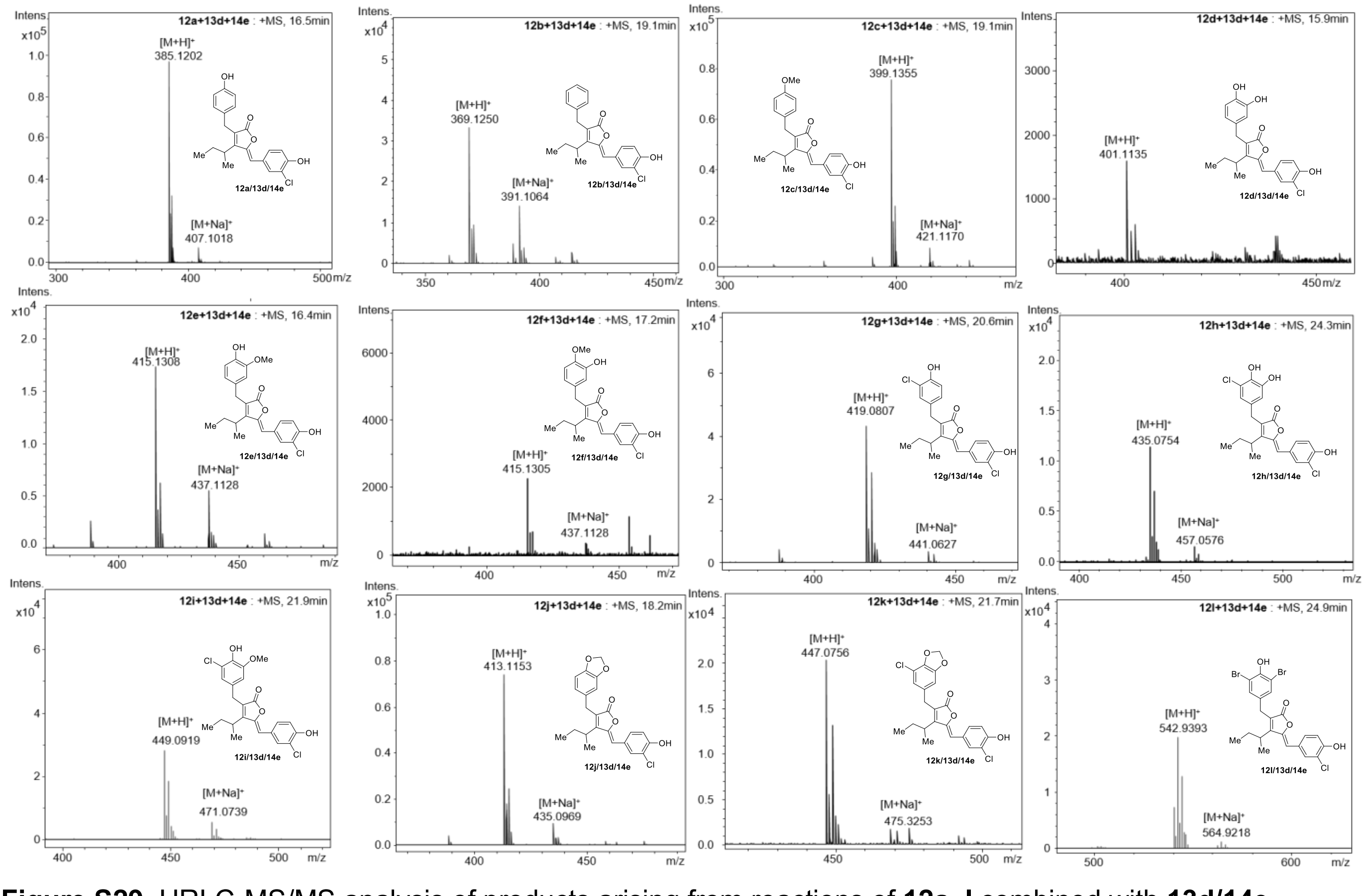

Figure S29. HRLC-MS/MS analysis of products arising from reactions of **12a–l** combined with **13d/14e**.

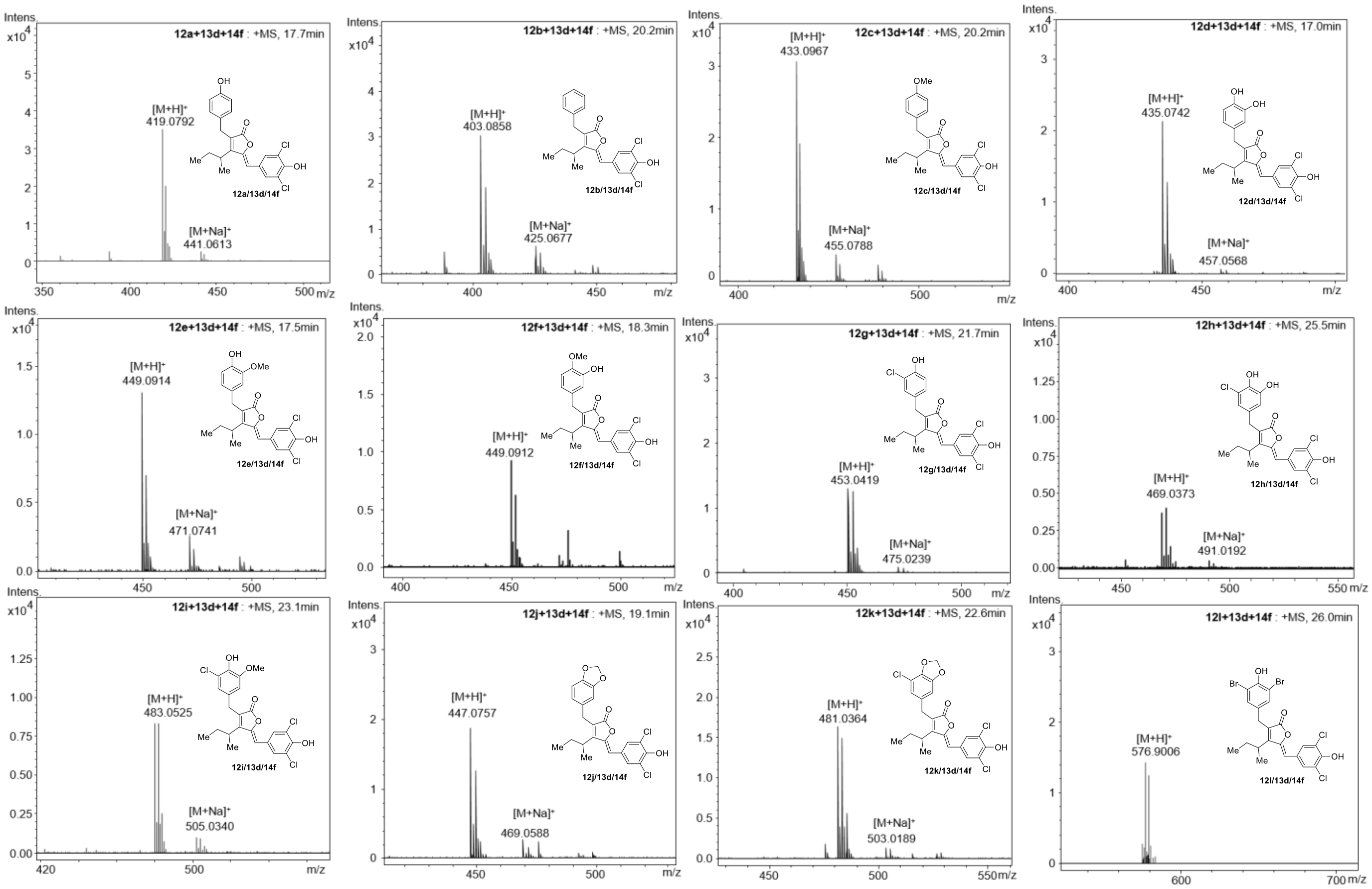

Figure S30. HRLC-MS/MS analysis of products arising from reactions of **12a–l** combined with **13d/14f**.

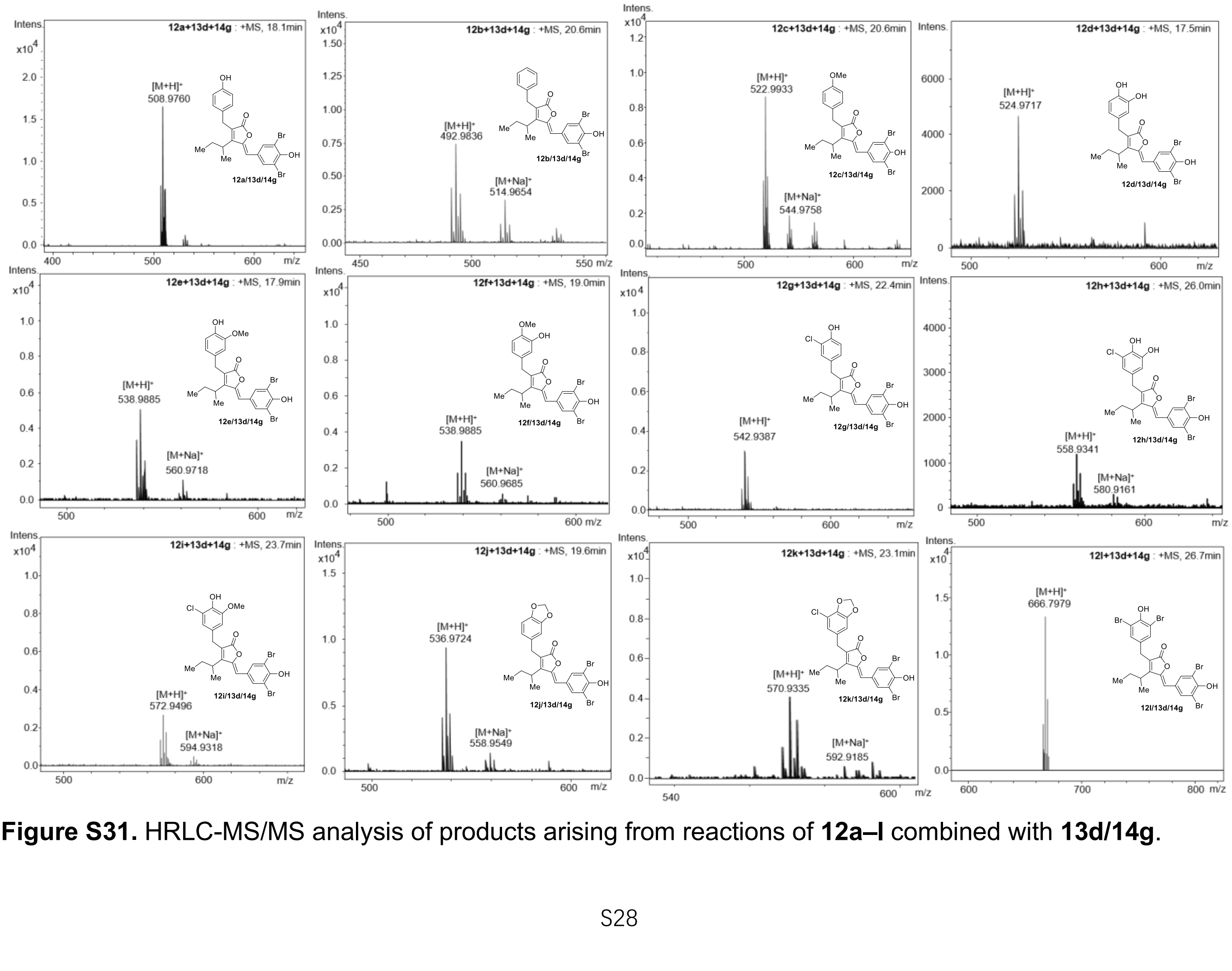

Figure S31. HRLC-MS/MS analysis of products arising from reactions of **12a–l** combined with **13d/14g**.

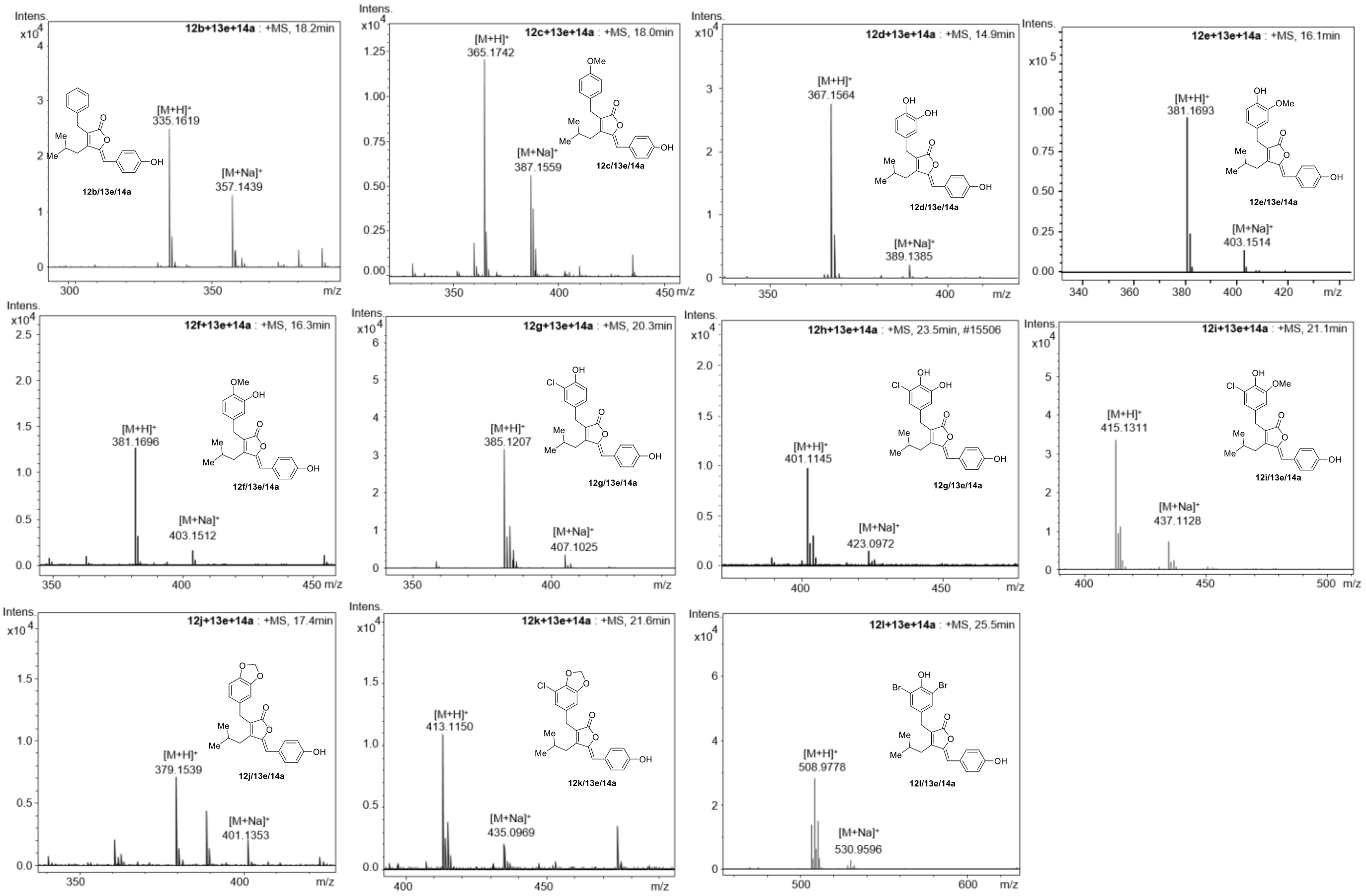

Figure S32. HRLC-MS/MS analysis of products arising from reactions of **12b–l** combined with **13e/14a**.

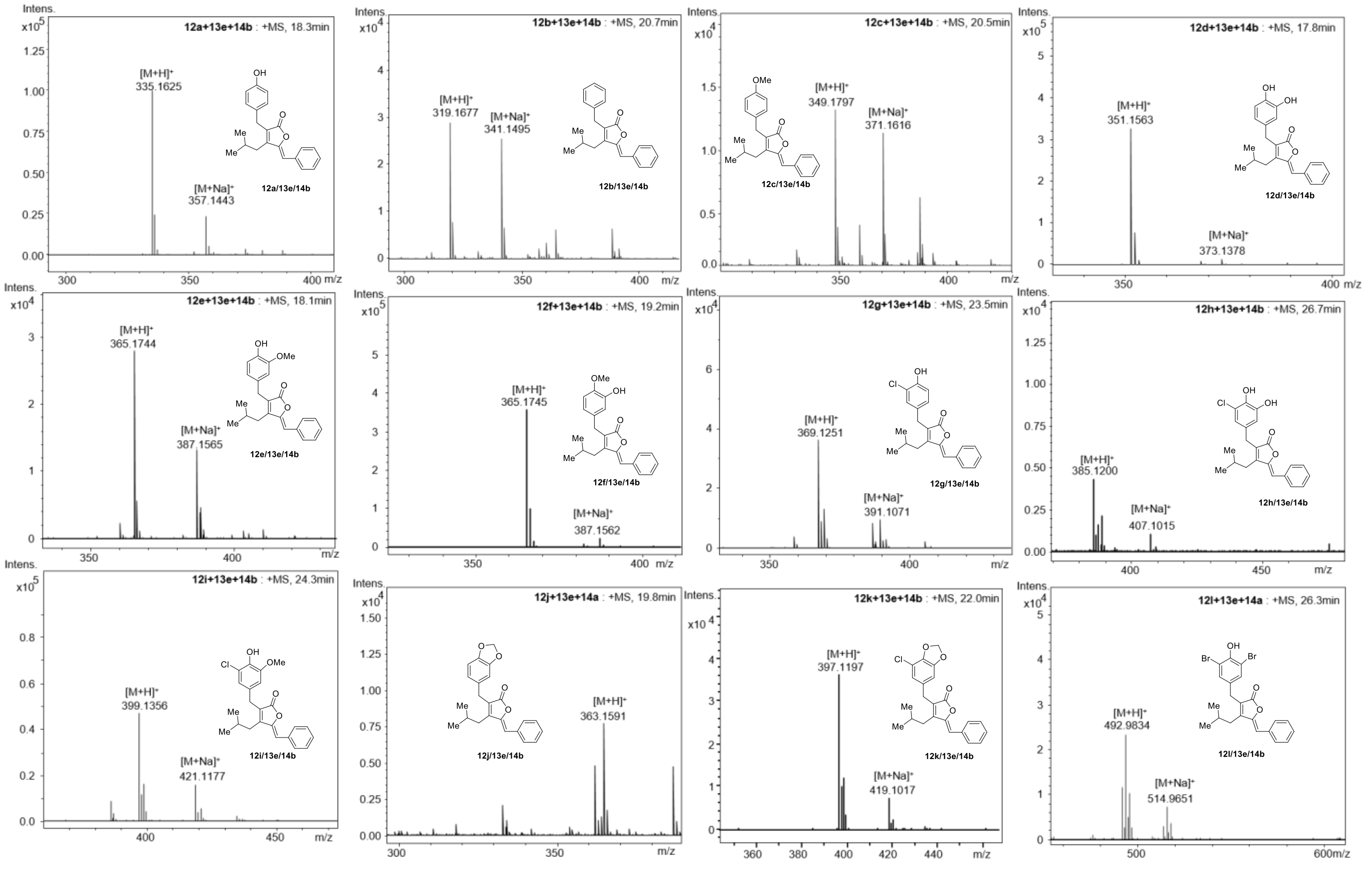

Figure S33. HRLC-MS/MS analysis of products arising from reactions of **12a–l** combined with **13e/14b**.

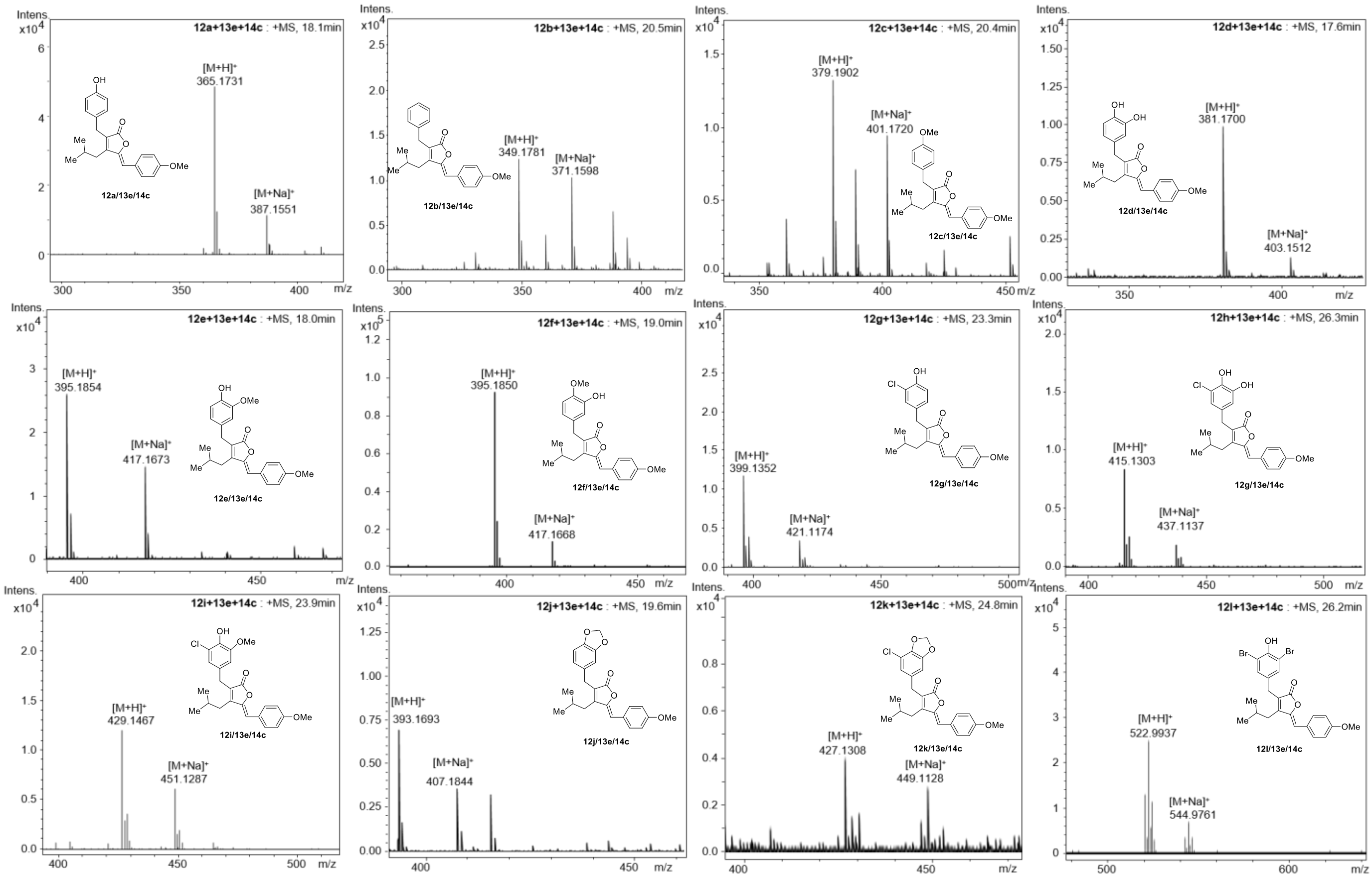

Figure S34. HRLC-MS/MS analysis of products arising from reactions of **12a–l** combined with **13e/14c**.

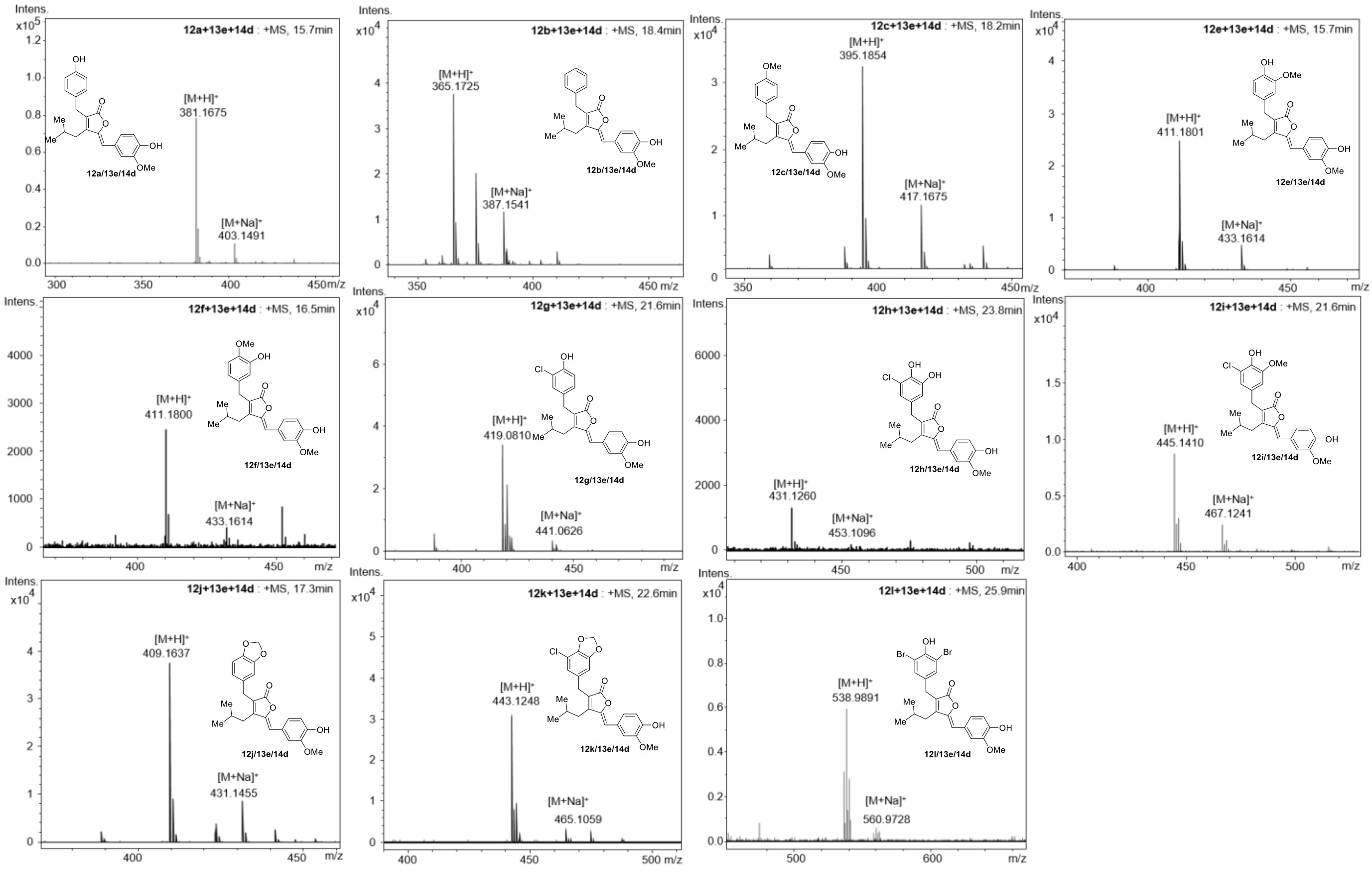

Figure S35. HRLC-MS/MS analysis of products arising from reactions of **12a–c,e–l** combined with **13e/14d**.

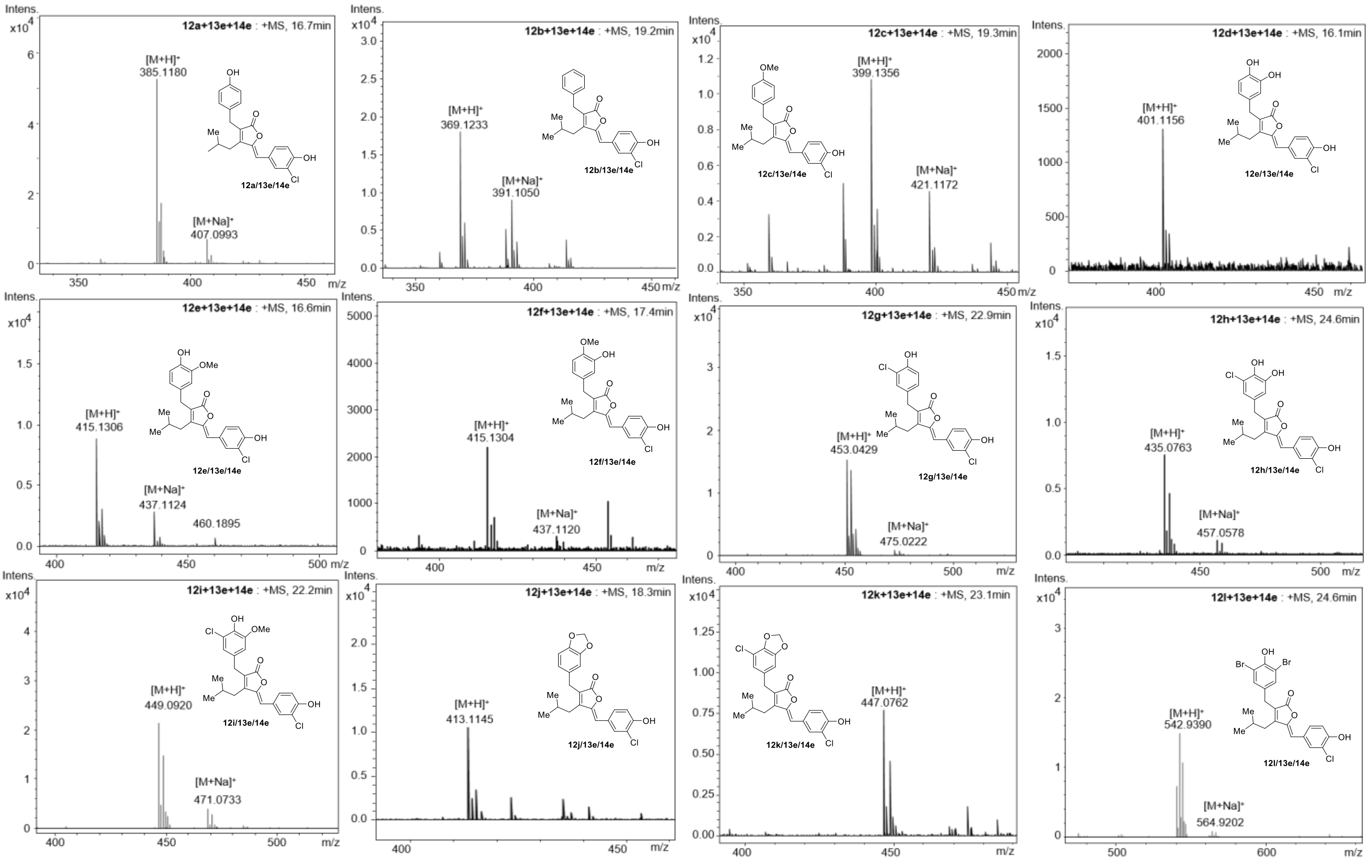

Figure S36. HRLC-MS/MS analysis of products arising from reactions of **12a–l** combined with **13e/14e**.

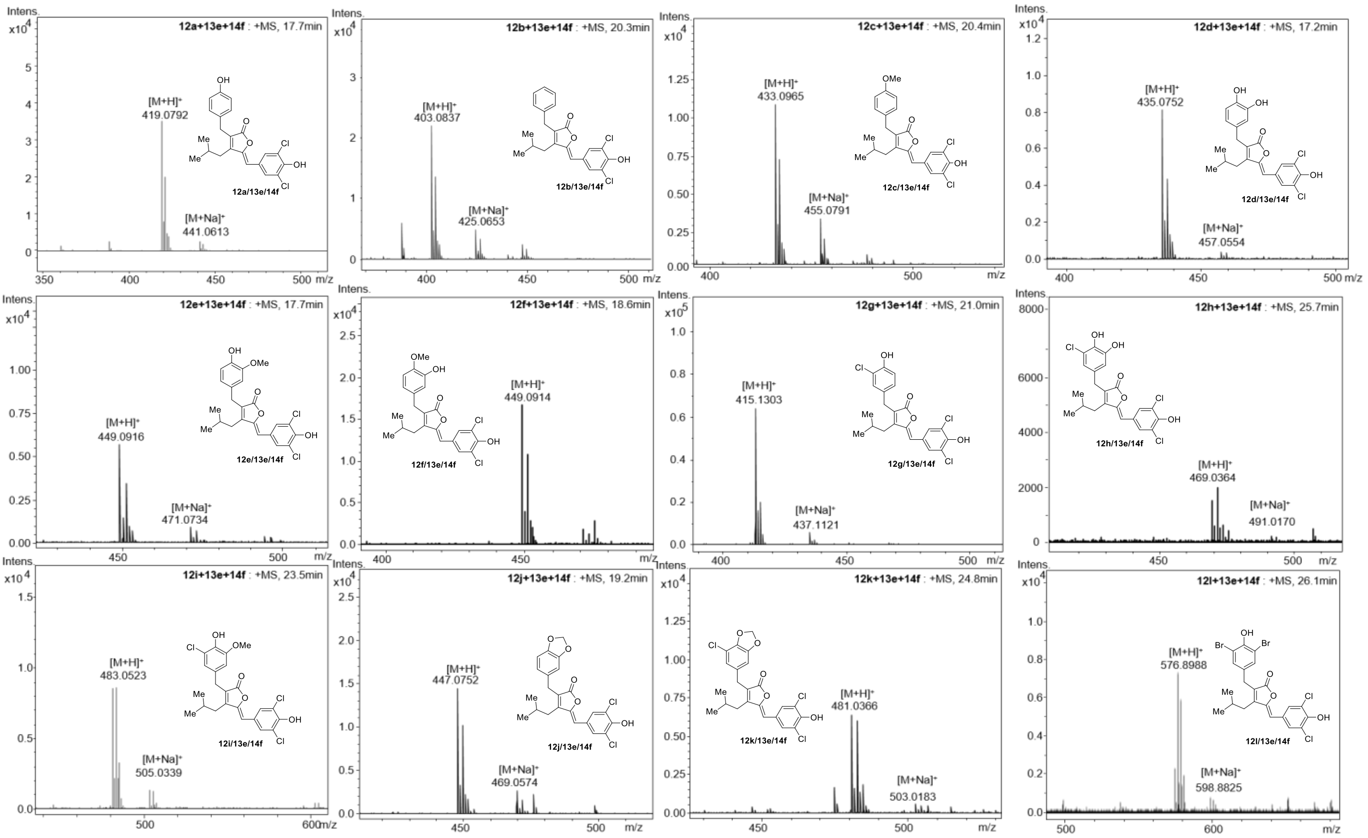

Figure S37. HRLC-MS/MS analysis of products arising from reactions of **12a–l** combined with **13e/14f**.

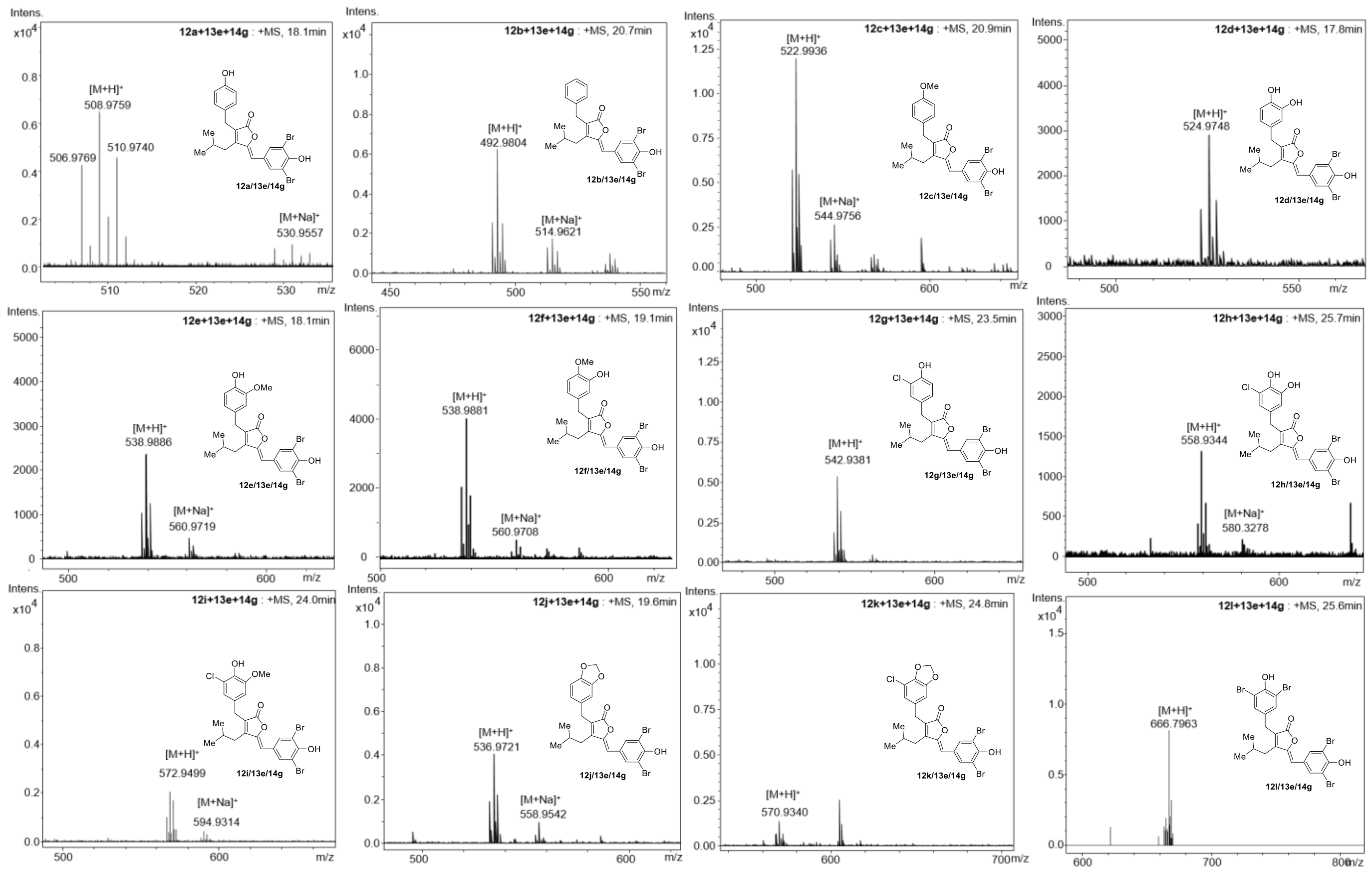

Figure S38. HRLC-MS/MS analysis of products arising from reactions of **12a–l** combined with **13e/14g**.

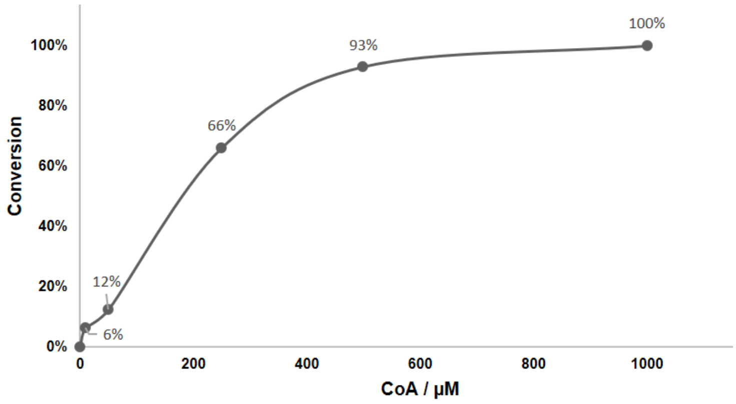

Figure S39. Determination of conversion rate at different concentrations of CoA using **12a, 13a** and **14a** as substrates.

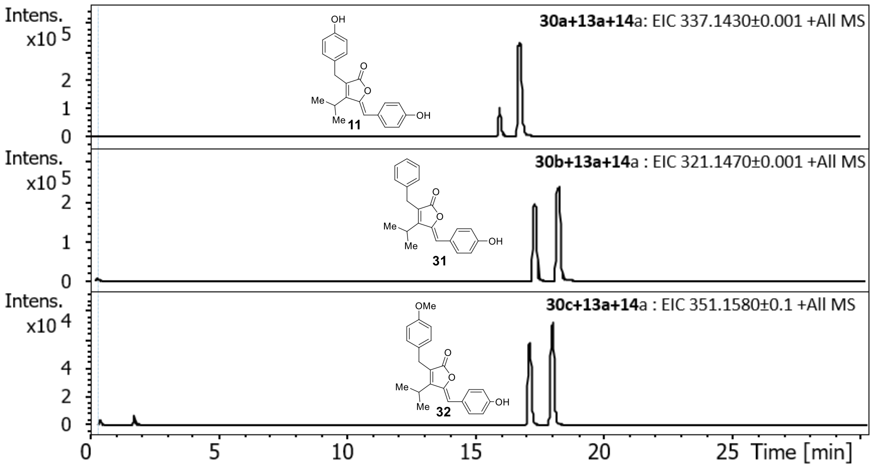

Figure S40. Results of the assays using SNAC-thioesters **30a–c** with **13a** and **14a**.

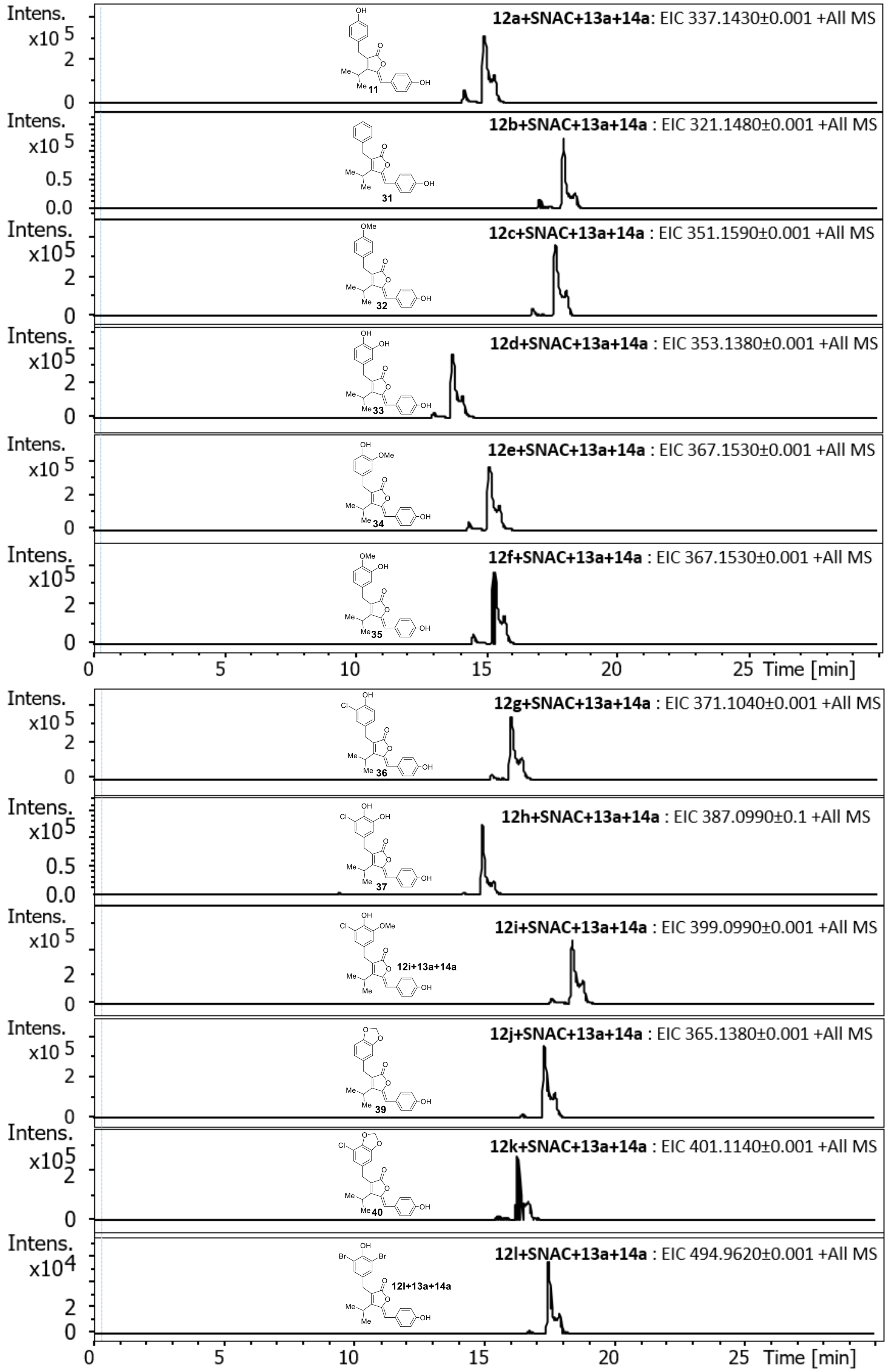

Figure S41. Results of assays using substrates **12a–l**, **13a**, **14a** with SNAC replacing CoA.

4. Biocatalytic Total Synthesis of Furanolides – Analytical Data of Isolated Products

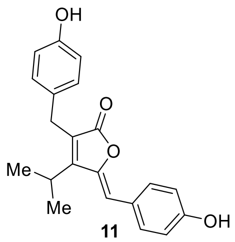

11

^{1}H-NMR (600 MHz, DMSO-d_6): δ [ppm] = 9.92 (s, 1H), 9.23 (s, 1H), 7.67 (d, *J* = 8.8 Hz, 2H), 6.99 (d, *J* = 8.6 Hz, 2H), 6.82 (d, *J* = 8.8 Hz, 2H), 6.67 (d, *J* = 8.5 Hz, 2H), 6.44 (s, 1H), 3.63 (s, 2H), 3.21 (sept , *J* = 7.1 Hz, 1H), 1.24 (d, *J* = 7.1 Hz, 6H).
^{13}C{^{1}H}-NMR (151 MHz, DMSO-d_6): δ [ppm] = 170.1, 158.3, 157.8, 155.7, 144.4, 132.3 (2C), 129.0 (2C), 128.5, 124.4, 123.7, 115.8 (2C), 115.3 (2C), 110.4, 28.0, 25.5, 21.3 (2C).
HR-MS (ESI+): *m/z* calcd. for $C_{21}H_{20}O_4$ $[M+H]^+$: 337.1435; found: 337.1435.

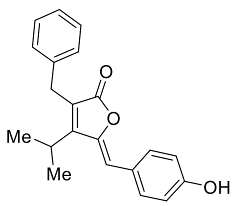

31

^{1}H-NMR (600 MHz, DMSO-d_6, 4:1 *E*/*Z* isomers): δ [ppm] = 9.94 (s, 1H major), 9.94 (s, 0.24H minor), 7.68 (d, *J* = 8.7 Hz, 2H major), 7.31 (d, *J* = 8.5 Hz 0.56H minor), 7.30–7.17 (m, 6.25H for two isomers), 6.83 (d, *J* = 8.8 Hz, 2H major), 6.79 (d, *J* = 8.5 Hz, 0.56H), 6.47 (s, 1H major), 6.95 (s, 0.25H minor), 3.76 (s, 2H major), 3.83 (s, 0.52H minor), 3.24 (sept, *J* = 7.1 Hz, 1H major), 2.82 (sept, *J* = 7.1 Hz, 0.27H minor), 1.25 (d, *J* = 7.1 Hz, 6H major), 1.04 (d, *J* = 7.1 Hz, 1.59H minor).
^{13}C{^{1}H}-NMR (151 MHz, DMSO-d_6, 4:1 *E*/*Z* isomers): δ [ppm] = 170.1, 158.4, 158.3, 144.4, 138.6, 132.4 (2C), 128.5 (2C), 128.1 (2C), 126.3, 124.3, 123.1, 115.8 (2C), 110.7, 28.9, 25.6, 21.3 (2C) (major); 169.7, 157.8, 156.6, 147.4, 138.1, 130.8 (2C), 128.5 (2C), 128.0 (2C), 126.3, 124.3, 123.3, 115.8, 115.2 (2C), 29.7, 25.4, 20.8 (2C) (minor).
HR-MS (ESI+): *m/z* calcd. for $C_{21}H_{20}O_3$ $[M+H]^+$: 321.1486; found: 321.1486.

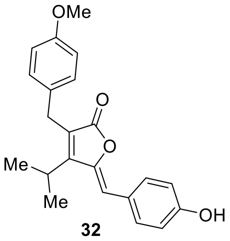

32

^{1}H-NMR (600 MHz, DMSO-d_6, 4:1 *E*/*Z* isomers): δ [ppm] = 9.93 (s, 1H major), 9.77 (s, 0.25H minor), 7.67 (d, *J* = 8.8 Hz, 2H major), 7.20 (d, *J* = 7.8 Hz, 0.53H minor), 7.11 (d, *J* = 8.4 Hz, 2.53H for two isomers), 6.87–6.77 (m, 5H for two isomers), 6.46 (s, 1H major), 6.93 (s, 0.26H minor), 3.71 (s, 3H major), 3.71 (s, 0.77H minor) 3.68 (s, 2H major), 3.75 (s, 0.5H minor), 3.23 (sept, *J* = 7.1 Hz, 1H major), 2.84 – 2.78 (sept, 0.29H minor), 1.25 (d, *J* = 7.1 Hz, 6H major), 1.05 (d, *J* = 7.1 Hz, 1.59H minor).
^{13}C{^{1}H}-NMR (151 MHz, DMSO-d_6): δ [ppm] = 170.1, 158.3, 158.0, 157.7, 144.4, 132.3 (2C), 132.3, 129.1 (2C), 124.4, 123.5, 115.8 (2C), 113.9 (2C), 110.6, 55.0, 28.0, 25.5, 21.3 (2C) (major).
HR-MS (ESI+): *m/z* calcd. for $C_{22}H_{22}O_4$ $[M+H]^+$: 351.1591; found: 351.1591.

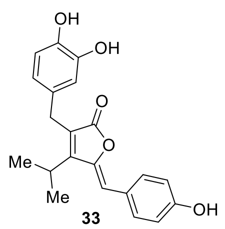

33

^{1}H-NMR (600 MHz, DMSO-d_6): δ [ppm] = 9.93 (s, 1H), 8.81 (s, 1H), 8.67 (s, 1H), 7.67 (d, *J* = 8.8 Hz, 2H), 6.83 (d, *J* = 8.8 Hz, 2H), 6.62 (d, *J* = 7.9 Hz, 1H), 6.56 (d, *J* = 2.2 Hz, 1H), 6.45 (s, 1H), 6.44 (d, *J* = 2.2 Hz, 1H), 3.57 (s, 2H), 3.20 (sept, *J* = 7.1 Hz, 1H), 1.25 (d, *J* = 7.1 Hz, 6H).
^{13}C{^{1}H}-NMR (151 MHz, DMSO-d_6): δ [ppm] = 170.2, 158.3, 157.7, 145.2, 144.4, 143.7, 132.3 (2C), 129.2, 124.4, 123.8, 118.8, 115.8 (2C), 115.6, 115.4, 110.4, 28.1, 25.6, 21.3 (2C).
HR-MS (ESI+): *m/z* calcd. for $C_{21}H_{20}O_5$ $[M+H]^+$: 353.1384; found: 353.1384.

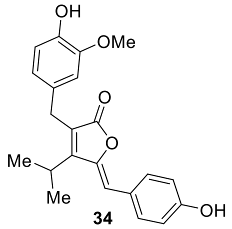

34

^{1}H-NMR (600 MHz, DMSO-d_6, 2:1 *E*/*Z* isomers): δ [ppm] = 9.93 (s, 1H major), 9.76 (s, 0.46H minor), 8.80 (s, 1.44H for two isomers), 7.67 (d, *J* = 8.8 Hz, 2H major), 7.19 (d, *J* = 7.7 Hz, 0.97H minor), 6.84–6.77 (m, 4.48H for two isomers), 6.67 (d, *J* = 8.1 Hz, 1.51H for two isomers), 6.54 (dd, *J* = 17.8, 7.1 Hz, 1.52H for two isomers), 6.45 (s, 1H major), 6.92 (s, 0.46H minor), 3.72 (d, 4.49H for two isomers), 3.64 (s, 2H major), 3.71 (s, 1H minor), 3.24 (sept, *J* = 7.1 Hz, 1H major), 2.81 (sept, *J* = 7.1 Hz, 0.51H minor), 1.26 (d, *J* = 7.1 Hz, 6H major), 1.05 (d, *J* = 7.1 Hz, 3H minor).
^{13}C{^{1}H}-NMR (151 MHz, DMSO-d_6, 2:1 *E*/*Z* isomers): δ [ppm] = 170.1, 158.3, 157.9, 147.5, 145.0, 144.4, 132.3 (2C), 130.8, 124.4, 123.6, 120.2, 115.8 (2C), 115.2, 112.4, 110.5, 55.6, 28.4, 25.6, 21.3 (2C) (major); 169.8 157.7, 156.1, 147.5, 147.5, 145.0, 129.2 (2C), 129.0, 128.7, 123.4, 120.1, 115.5, 115.5 (2C), 115.4, 112.6, 55.6, 29.3, 25.4, 20.9 (2C) (minor).
HR-MS (ESI+): *m/z* calcd. for $C_{22}H_{22}O_5$ $[M+H]^+$: 367.1540; found: 367.1540.

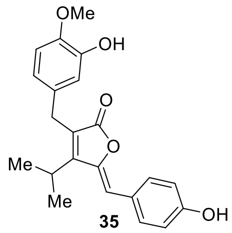

35

^{1}H-NMR (600 MHz, DMSO-d_6): δ [ppm] = 9.94 (s, 1H), 8.92 (s, 1H), 7.67 (d, *J* = 8.8 Hz, 2H), 6.84–6.80 (m, 3H), 6.61 (d, *J* = 2.2 Hz, 1H), 6.57 (dd, *J* = 8.2, 2.2 Hz, 1H), 6.46 (s, 1H), 3.71 (s, 3H), 3.61 (s, 2H), 3.21 (sept, *J* = 7.1 Hz, 1H), 1.25 (d, *J* = 7.1 Hz, 6H).
^{13}C{^{1}H}-NMR (151 MHz, DMSO-d_6): δ [ppm] = 170.1, 158.3, 158.0, 146.5, 146.1, 144.4, 132.3 (2C), 131.0, 124.4, 123.5, 118.6, 115.8 (2C), 115.3, 112.4, 110.5, 55.6, 28.1, 25.6, 21.3 (2C).
HR-MS (ESI+): *m/z* calcd. for $C_{22}H_{22}O_5$ $[M+H]^+$: 367.1540; found: 367.1540.

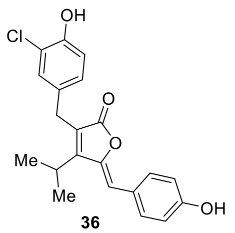

36

^{1}H-NMR (600 MHz, DMSO-d_6): δ [ppm] = 10.01 (s, 1H), 9.94 (s, 1H), 7.68 (d, *J* = 8.8 Hz, 2H), 7.16 (d, *J* = 2.2 Hz, 1H), 6.96 (dd, *J* = 8.4, 2.2 Hz, 1H), 6.88 (d, *J* = 8.3 Hz, 1H), 6.83 (d, *J* = 8.8 Hz, 2H), 6.47 (s, 1H), 3.65 (s, 2H), 3.23 (sept, *J* = 7.1 Hz, 1H), 1.25 (d, *J* = 7.1 Hz, 6H).
^{13}C{^{1}H}-NMR (151 MHz, DMSO-d_6): δ [ppm] = 170.1, 158.3, 158.3, 151.4, 144.4, 132.4 (2C), 130.2, 129.3, 127.6, 124.3, 123.0, 119.4, 116.7, 115.8 (2C), 110.8, 27.6, 25.5, 21.3 (2C).
HR-MS (ESI+): *m/z* calcd. for $C_{21}H_{19}ClO_4$ $[M+H]^+$: 371.1045; found: 371.1045.

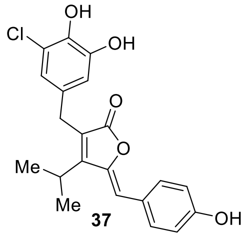

37

^{1}H-NMR (600 MHz, Methanol-d_4, 1:0.35 *E*/*Z* isomers): δ [ppm] = 9.95 (s, 1H major), 9.78 (s, 0.36H minor), 9.67 (s, 1.26H for two isomers), 8.95 (s, 1.31H for two isomers), 7.68 (d, *J* = 8.8 Hz, 2H major), 7.20 (d, *J* = 8.7 Hz, 0.69H minor), 6.83 (d, *J* = 8.8 Hz, 2H major), 6.79 (d, *J* = 8.5 Hz, 0.74H minor), 6.63 (d, *J* = 2.1 Hz, 1H major), 6.62 (d, *J* = 2.1 Hz, 0.39H minor), 6.55 (d, *J* = 2.0 Hz, 1.34H for two isomers), 6.49 (s, 1H major), 6.95 (s, 0.34H minor), 3.59 (s, 2H major), 3.65 (s, 0.70H minor), 3.21 (sept, *J* = 7.1 Hz, 1H major), 2.81 (sept, *J* = 7.1 Hz, 0.44H minor), 1.26 (d, *J* = 7.1 Hz, 6H major), 1.05 (d, *J* = 7.1 Hz, 1.96H minor).
^{13}C{^{1}H}-NMR (151 MHz, Methanol-d_4, 1:0.35 *E*/*Z* isomers): δ [ppm] = 170.1, 158.4, 158.3, 146.6, 144.4, 140.2, 132.4 (2C), 129.8, 124.3, 123.0, 120.0, 119.3, 115.9 (2C), 113.7, 110.8, 27.8, 25.5, 21.3 (2C) (major); 169.7, 157.8, 156.5, 147.4, 146.6, 130.8 (2C), 129.3, 128.6, 123.3, 120.0, 119.3, 115.9, 115.2 (2C), 113.8, 113.7, 28.7, 25.4, 20.9 (2C) (minor).
HR-MS (ESI+): *m/z* calcd. for $C_{21}H_{19}ClO_5$ $[M+H]^+$: 387.0994; found: 387.0994.

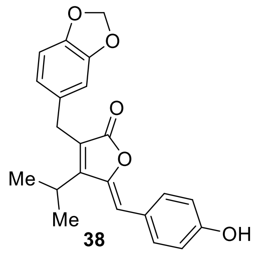

38

^{1}H-NMR (600 MHz, Methanol-d_4, 1:0.57 *E*/*Z* isomers): δ [ppm] = 9.94 (s, 1H major), 9.77 (s, 0.57H minor), 7.67 (d, *J* = 8.8 Hz, 2H major), 7.20 (d, *J* = 7.7 Hz, 1.15H minor), 6.84–6.77 (m, 6.26H for two isomers), 6.64 (dd, *J* = 12.6, 6.2 Hz, 1.64H for two isomers), 6.47 (s, 1H major), 6.94 (s, 0.52H minor), 5.97 (s, 2H major), 5.97 (s, 1.13H minor), 3.67 (s, 2H major), 3.73 (s, 1.18H minor), 3.23 (sept, *J* = 7.1 Hz, 1H major), 2.81 (sept, *J* = 7.1 Hz, 0.59H minor), 1.26 (d, *J* = 7.1 Hz, 6H major), 1.05 (d, *J* = 7.1 Hz, 3.44H minor).
^{13}C{^{1}H}-NMR (151 MHz, Methanol-d_4, 1:0.57 *E*/*Z* isomers): δ [ppm] = 170.1, 158.3, 158.2, 147.3, 145.7, 132.4 (2C), 132.3, 124.4, 123.2, 120.9, 115.8 (2C), 115.2, 108.6, 108.3, 100.8 (2C), 28.5, 25.5, 21.3 (2C) (major); 169.7, 156.8, 156.5, 147.4,147.3, 144.4, 131.8, 130.8 (2C), 128.6, 123.4, 120.9, 115.8 (2C), 110.7 (2C),108.6, 108.2, 29.3, 25.4, 20.9 (2C) (minor).
HR-MS (ESI+): *m/z* calcd. for $C_{22}H_{20}O_5$ $[M+H]^+$: 365.1384; found: 365.1384.

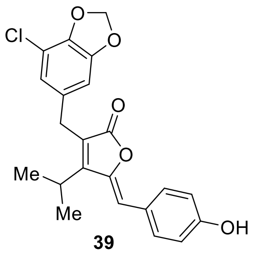

39

^{1}H-NMR (600 MHz, $CDCl_3$) : δ [ppm] = 7.72 (d, *J* = 8.7 Hz, 2H), 6.96 (d, *J* = 8.7 Hz, 2H), 6.68 (s, 1H), 6.64 (s, 1H), 6.19 (s, 1H), 6.00 (s, 2H), 3.70 (s, 2H), 3.13 (sept, *J* = 7.1 Hz, 1H), 1.34 (d, *J* = 7.2 Hz, 6H).
^{13}C{^{1}H}-NMR (151 MHz, $CDCl_3$): δ [ppm] = 171.4, 158.8, 157.2, 148.9, 145.5, 143.1, 133.2, 132.7 (2C), 125.7, 123.7, 121.9, 116.1 (2C), 113.9, 111.7, 107.6, 101.9, 29.2, 26.7, 22.0 (2C).
HR-MS (ESI+): *m/z* calcd. for $C_{22}H_{19}ClO_5$ $[M+H]^+$: 399.0994; found: 399.0994.

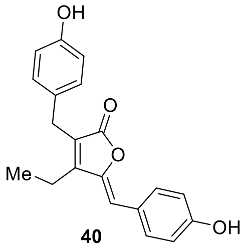

40

^{1}H-NMR (600 MHz, Methanol-*d*$_4$, 1:0.83 *E*/*Z* isomers): δ [ppm] = 9.92 (s, 1H major), 9.74 (s, 0.83H minor), 9.24 (s, 0.80H minor), 9.24 (s, 1H major), 7.63 (d, *J* = 8.8 Hz, 2H major), 7.21 (d, *J* = 8.6 Hz, 1.71H minor), 7.02 (d, *J* = 8.5 Hz, 2H major), 7.00 (d, *J* = 8.5 Hz, 1.63H minor), 6.82 (d, *J* = 8.8 Hz, 2H major), 6.78 (d, *J* = 8.5 Hz, 1.72H minor), 6.67 (d, *J* = 6.2 Hz, 2H major), 6.66 (d, *J* = 6.2 Hz, 1.67H minor), 6.35 (s, 1H major), 6.87 (s, 0.83H minor), 3.56 (s, 2H major), 3.54 (s, 1.57H minor), 2.61 (q, *J* = 7.6 Hz, 2H major), 2.40 (q, *J* = 7.6 Hz, 1.81H minor), 1.05 (t, *J* = 7.6 Hz, 3H major), 0.60 (t, *J* = 7.5 Hz, 2.53H minor).
^{13}C{^{1}H}-NMR (151 MHz, Methanol-*d*$_4$, 1:0.83 *E*/*Z* isomers): δ [ppm] = 170.0, 158.3, 155.8, 155.0, 145.2, 129.9, 129.2 (4C), 128.1, 124.3, 115.3 (4C), 109.3, 27.9, 18.7, 14.2 (major); 169.2, 157.8, 155.8, 152.0, 147.7, 132.1 (2C), 130.7 (2C), 128.4, 124.2, 123.1, 115.9 (2C), 115.2 (2C), 115.3, 27.9, 17.3, 12.5 (minor).
HR-MS (ESI+): *m/z* calcd. for $C_{20}H_{18}O_4$ $[M+H]^+$: 323.1278; found: 323.1278.

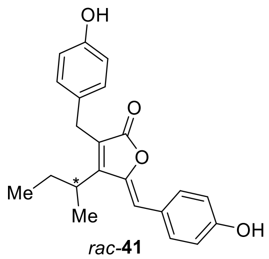

rac-**41**

^{1}H-NMR (600 MHz, Methanol-*d*$_4$): δ [ppm] = 9.95 (s, 1H), 9.24 (s, 1H), 7.67 (d, *J* = 8.8 Hz, 2H), 6.99 (d, *J* = 8.5 Hz, 2H), 6.82 (d, *J* = 8.8 Hz, 2H), 6.67 (d, *J* = 8.5 Hz, 2H), 6.42 (s, 1H), 3.61 (d, *J* = 7.3 Hz, 1H), 2.96 (h, *J* = 7.2 Hz, 1H), 1.65 (p, *J* = 7.5 Hz, 2H), 1.22 (d, *J* = 7.2 Hz, 3H), 0.78 (t, *J* = 7.4 Hz, 3H).
^{13}C{^{1}H}-NMR (151 MHz, Methanol-*d*$_4$): δ [ppm] = 170.1, 158.3, 156.9, 155.7, 144.7, 132.3, 130.7, 129.0 (2C), 128.6, 124.4, 124.4, 115.8 (2C), 115.3 (2C), 110.6, 32.7, 28.2, 28.1, 19.4, 12.4.
HR-MS (ESI+): *m/z* calcd. for $C_{22}H_{22}O_4$ $[M+H]^+$: 351.1591; found: 351.1591.

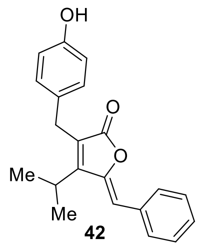

42

^{1}H-NMR (600 MHz, DMSO-d_6): δ [ppm] = 9.25 (s, 1H), 7.81 (d, J = 8.2 Hz, 2H), 7.44 (t, J = 7.7 Hz, 2H), 7.34 (t, J = 7.4 Hz, 1H), 7.00 (d, J = 8.5 Hz, 2H), 6.68 (d, J = 8.5 Hz, 2H), 6.56 (s, 1H), 3.66 (s, 2H), 3.26 (sept, J = 7.2 Hz, 1H), 1.27 (d, J = 7.1 Hz, 6H).
^{13}C{^{1}H}-NMR (151 MHz, DMSO-d_6): δ [ppm] = 169.9, 157.7, 155.8, 146.6, 133.3, 130.4 (2C), 129.1 (2C), 128.8 (2C), 128.7, 128.3, 125.3, 115.3 (2C), 109.9, 28.1, 25.6, 21.2 (2C).
HR-MS (ESI+): *m/z* calcd. for $C_{21}H_{20}O_3$ $[M+H]^+$: 321.1486; found: 321.1486.

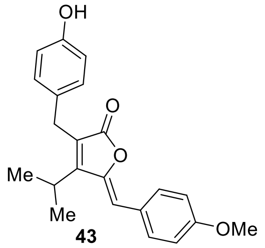

43

^{1}H-NMR (600 MHz, DMSO-d_6, 1:0.13 *E*/*Z* isomers): δ [ppm] = 9.24 (s, 1H major), 9.19 (s, 0.06H minor), 7.79 (s, 2H major), 7.32 (d, J = 8.8 Hz, 0.41H minor), 7.04–6.95 (m, 4.75H for two isomers), 6.70–6.65 (m, 2.36H for two isomers), 6.51 (s, 1H major), 6.96 (s, 0.2H minor), 3.79 (s, 3H major), 3.78 (s, 0.6H minor), 3.64 (s, 2H major), 3.70 (s, 0.4H minor), 3.23 (sept, J = 7.1 Hz, 1H major), 2.76 (sept, J = 7.1 Hz, 0.24H minor), 1.26 (d, J = 7.1 Hz, 6H major), 1.03 (d, J = 7.1 Hz, 1.13H minor).
^{13}C{^{1}H}-NMR (151 MHz, DMSO-d_6, 1:0.13 *E*/*Z* isomers): δ [ppm] = 170.1, 159.6, 157.8, 155.8, 145.0, 132.1 (2C), 129.0 (2C), 128.4, 125.9, 124.2, 115.3 (2C), 114.4 (2C), 109.9, 55.3, 28.0, 25.6, 21.3 (2C) (major); 169.7, 159.5, 156.0, 155.8, 147.8, 130.7 (2C), 129.4, 129.0 (2C), 128.0, 125.1, 115.2 (2C), 114.9, 113.8 (2C), 55.2, 28.9, 25.5, 20.8 (2C) (minor).
HR-MS (ESI+): *m/z* calcd. for $C_{22}H_{22}O_4$ $[M+H]^+$: 351.1591; found: 351.1591.

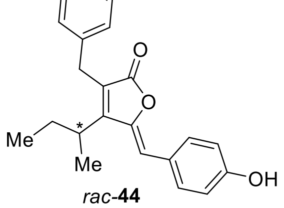

rac-**44**

^{1}H-NMR (600 MHz, DMSO-d_6): δ [ppm] = 9.90 (s, 1H), 7.68 (d, J = 8.8 Hz, 2H), 7.31 – 7.17 (m, 5H), 6.83 (d, J = 8.8 Hz, 2H), 6.45 (s, 1H), 3.75 (d, J = 6.7 Hz, 2H), 2.97 (h, J = 7.3 Hz, 1H), 1.69 – 1.61 (m, 2H), 1.23 (d, J = 7.1 Hz, 3H), 0.78 (t, J = 7.4 Hz, 3H).
^{13}C{^{1}H}-NMR (151 MHz, DMSO-d_6): δ [ppm] = 170.0, 158.4, 157.5, 144.7, 138.6, 132.4 (2C), 128.5 (2C), 128.1 (2C), 126.3, 124.3, 123.8, 115.8 (2C), 110.8, 32.7, 28.9, 28.2, 19.4, 12.3.
HR-MS (ESI+): *m/z* calcd. for $C_{22}H_{22}O_3$ $[M+H]^+$: 335.1642; found: 335.1642.

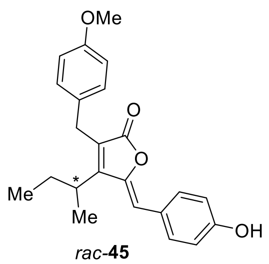

rac-**45**

^{1}H-NMR (600 MHz, DMSO-d_6): δ [ppm] = 9.95 (s, 1H), 7.68 (d, *J* = 8.8 Hz, 2H), 7.12 (d, *J* = 8.7 Hz, 2H), 6.84 (dd, *J* = 11.8, 8.7 Hz, 4H), 6.44 (s, 1H), 3.70 (s, 3H), 3.67 (d, *J* = 3.3 Hz, 2H), 3.01 – 2.92 (m, 1H), 1.65 (p, *J* = 7.1 Hz, 2H), 1.23 (d, *J* = 7.1 Hz, 3H), 0.79 (t, *J* = 7.3 Hz, 3H).

^{13}C{^{1}H}-NMR (151 MHz, DMSO-d_6): δ [ppm] = 170.0, 158.3, 157.7, 157.1, 144.7, 132.4 (2C), 130.3, 129.1 (2C), 124.4, 124.2, 115.8 (2C), 113.9 (2C), 110.7, 55.0, 32.7, 28.2, 28.0, 19.4, 12.3.

HR-MS (ESI+): *m/z* calcd. for $C_{23}H_{24}O_4$ $[M+H]^+$: 365.1748; found: 365.1748.

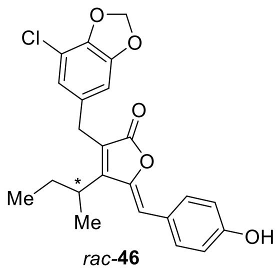

rac-**46**

^{1}H-NMR (600 MHz, DMSO-d_6, 1:0.35 *E*/*Z* isomers): δ [ppm] = 9.95 (s, 1H major), 9.76 (s, 0.35H minor), 7.69 (d, *J* = 8.8 Hz, 2H major), 7.20 (d, 0.71H minor), 6.83 (d, *J* = 8.8 Hz, 2H major), 6.79 (d, *J* = 8.5 Hz, 0.71H minor), 6.77 (d, *J* = 4.9 Hz, 1.3H for two isomers), 6.76 (s, 1H major), 6.72 (s, 0.36H minor), 6.47 (s, 1H major), 6.97 (s, 0.34H minor), 6.09 (s, 2H major), 6.10 (s, 0.73H minor), 3.66 (d, J = 2.5 Hz, 2H major), 3.71 (d, *J* = 2.8 Hz, 0.69H minor), 2.99 (m, *J* = 7.3 Hz, 1H major), 2.56 (m, *J* = 7.2 Hz, 0.41H minor), 1.66 (p, *J* = 7.4 Hz, 2H major), 1.52 (p, 0.45H minor), 1.25 (d, *J* = 7.1 Hz, 3H major), 1.06 (d, 1.04H minor), 0.80 (t, *J* = 7.4 Hz, 3H major), 0.57 (t, *J* = 7.3 Hz, 1.05H minor).

^{13}C{^{1}H}-NMR (151 MHz, DMSO-d_6, 1:0.35 *E*/*Z* isomers): δ [ppm] = 170.0, 158.4, 157.8, 148.5, 144.6, 142.4, 133.8, 132.5 (2C), 124.3, 123.3, 121.1, 115.8 (2C), 112.2, 111.1, 107.6, 102.0, 32.6, 28.3, 28.2, 19.4, 12.3 (major); 169.6, 157.8, 156.4, 148.5, 148.0, 133.3, 130.8 (2C), 127.8, 123.4, 121.1, 116.1, 115.2 (2C), 112.2, 111.1, 107.6, 102.0, 32.3, 29.2, 27.9, 18.3, 12.1 (minor).

HR-MS (ESI+): *m/z* calcd. for $C_{23}H_{21}ClO_5$ $[M+H]^+$: 413.1151; found: 413.1151.

5. Biological activity evaluation

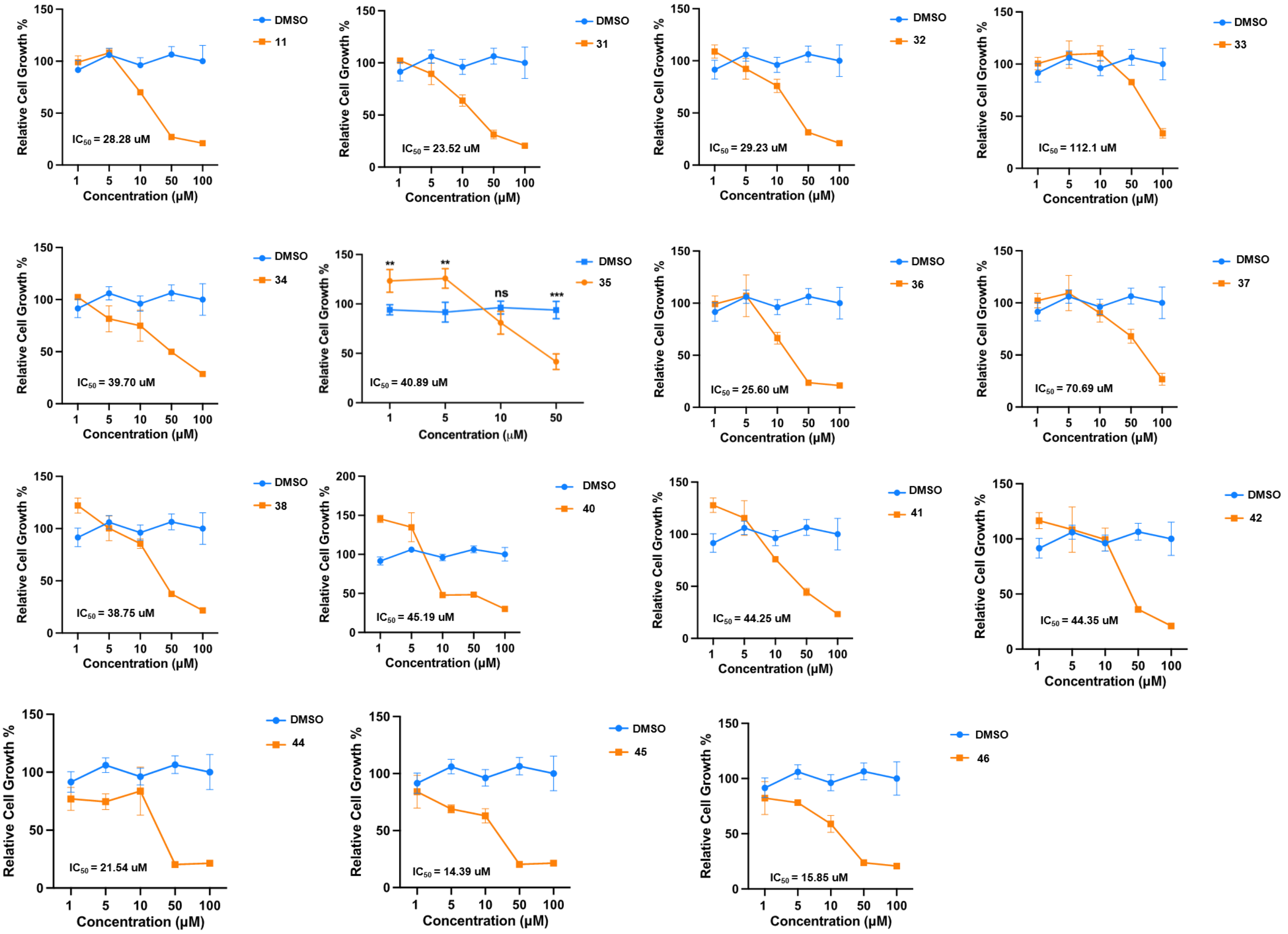

Figure S42. Determination of IC_{50} values of compounds **11, 31–38, 40–42, 44–46** against A549 human lung cancer cell line. MTT proliferation assays were carried out in four technical replicates. The activity of compounds against A549 human lung cancer cell line after 48 h (n = 4) is shown with respective IC_{50}-values, derived in GraphPad Prism 10 after log transformation of raw values, followed by non-linear regression analysis of dose-response inhibition.

Table S1. The SAR heatmap of compounds **11**, **31–46** against Gram-positive bacteria and A549 human lung cancer cell line. Compound **11** shows the general structure of tested furanolides. The most common substituent at each position R¹–R³ is shown. Analogs depicted in the table are structurally altered at the residues shown, colored according to their position with respect to the general structure.

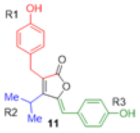

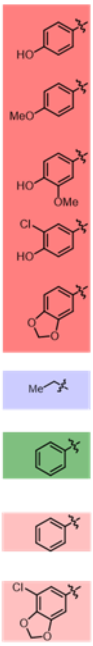

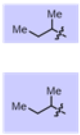

Compound	*Enterococcus faecalis* ATCC29212 (MICs µg/mL)	*E. faecium* ATCC51559 (MDR) (MICs µg/mL)	*Staphylococcus aureus* str. Newman (MICs µg/mL)	*S. aureus* ATCC29213 (MICs µg/mL)	*Streptococcus pneumoniae* DSM20566 (MICs µg/mL)	A549 cells (IC_{50} µM)	Compound
**11**	64	32->64	32	>64	32	28.28	**11**
**31**	>64	>64	>64	>64	64->64	23.52	**31**
**32**	>64	>64	16	>64	>64	29.23	**32**
**33**	>64	>64	>64	>64	64	112.1	**33**
**34**	>64	>64	>64	>64	64	39.70	**34**
**35**	64->64	16	>64	>64	>64	40.89	**35**
**36**	16-32	8	8-16	64	8	25.60	**36**
**37**	16-32	32	>64	>64	64	70.69	**37**
**38**	>64	>64	16-32	>64	>64	39.75	**38**
**39**	>64	32->64	16	>64	>64	10.24	**39**
**40**	>64	>64	>64	>64	>64	45.19	**40**
**41**	64	16	16-32	64->64	16-32	44.25	**41**
**42**	>64	16	32-64	32-64	16-32	44.35	**42**
**43**	>64	16-32	16-32	64->64	16	5.734	**43**
**44**	16	8	8	8	8	21.54	**44**
**45**	>64	16-32	4-8	4	4	14.39	**45**
**46**	64->64	16	8	32	4	15.85	**46**
Vancomycin (ref.)	2	>64	2	1	1	Cisplatin(ref.) 33	

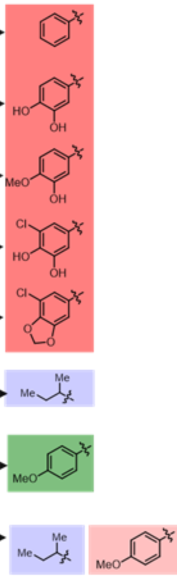

6. NMR Spectra

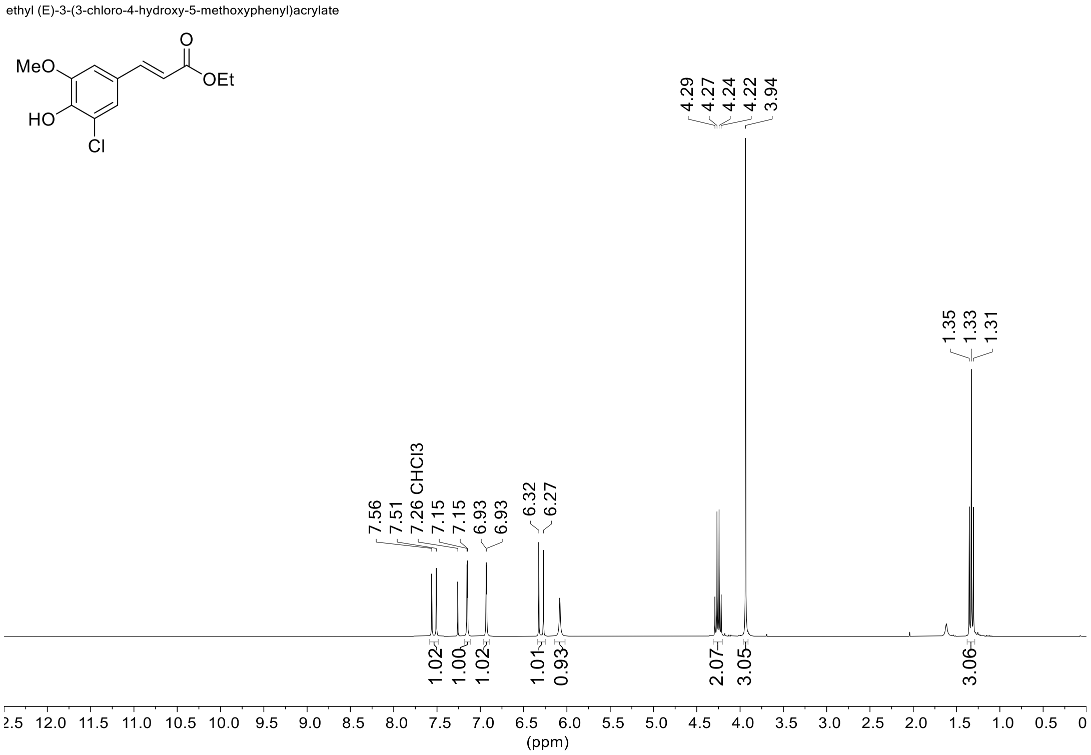

Figure S43. ^{1}H-NMR spectrum (300 MHz) of ethyl (*E*)-3-(3-chloro-4-hydroxy-5-methoxyphenyl)acrylate.

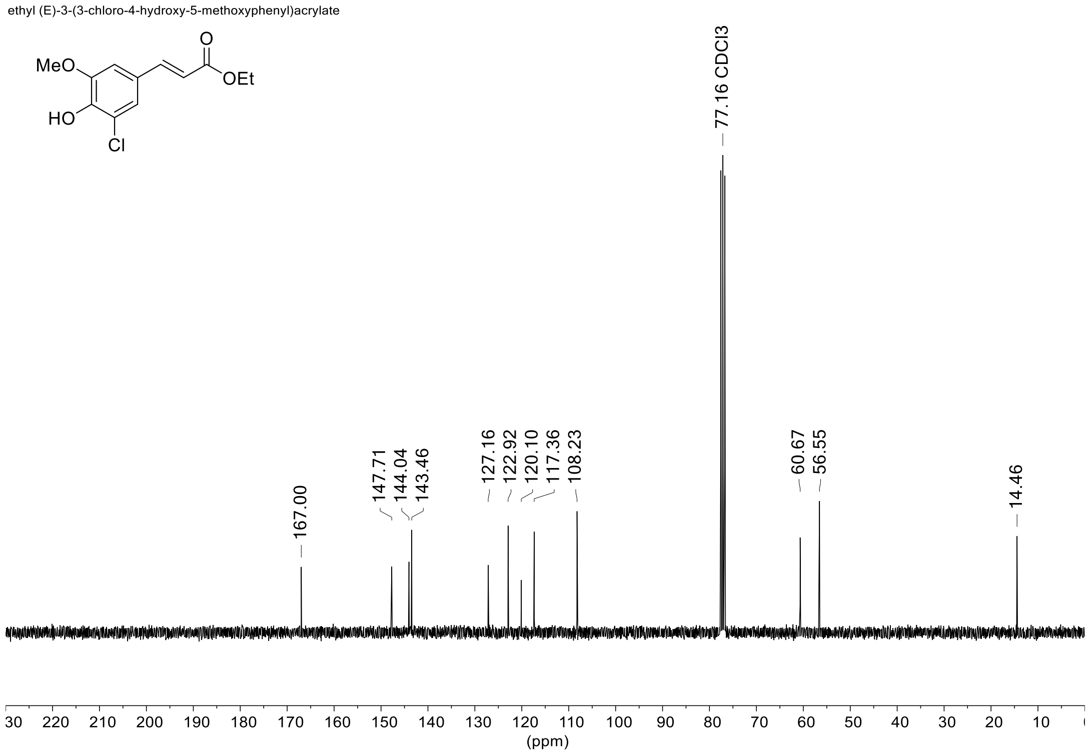

Figure S44. ^{13}C{^{1}H}-NMR spectrum (75.5 MHz) of ethyl (*E*)-3-(3-chloro-4-hydroxy-5-methoxyphenyl)acrylate.

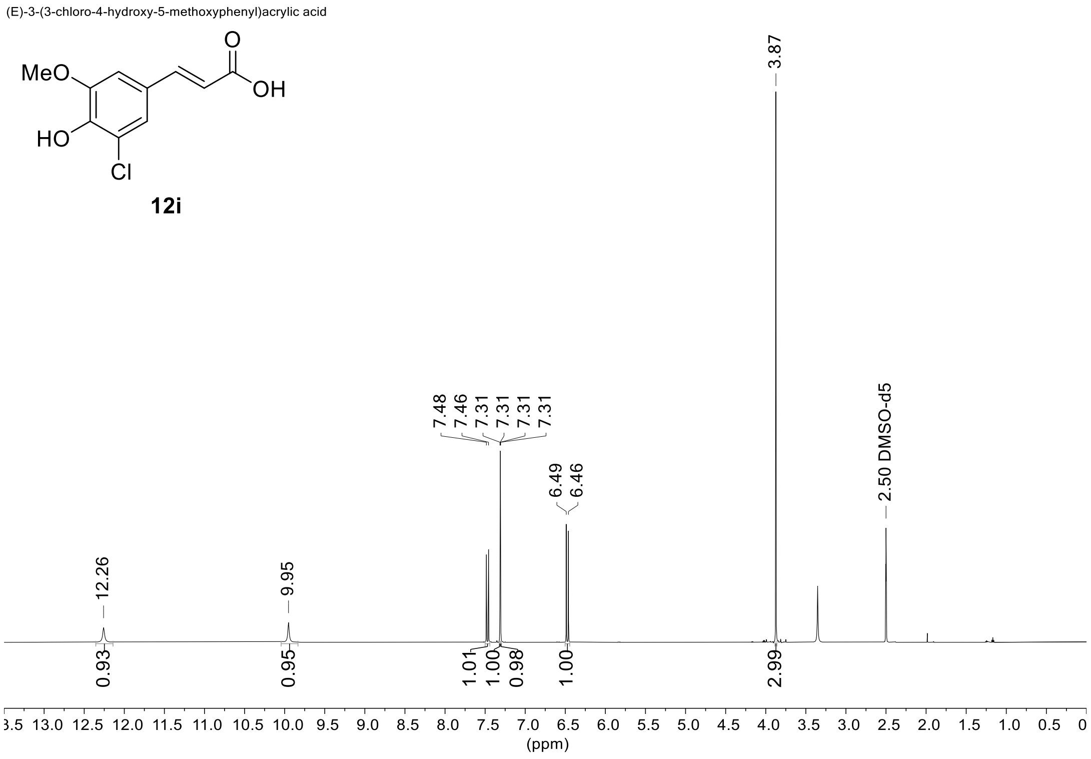

Figure S45. ^{1}H-NMR spectrum (600 MHz) of compound **12i**.

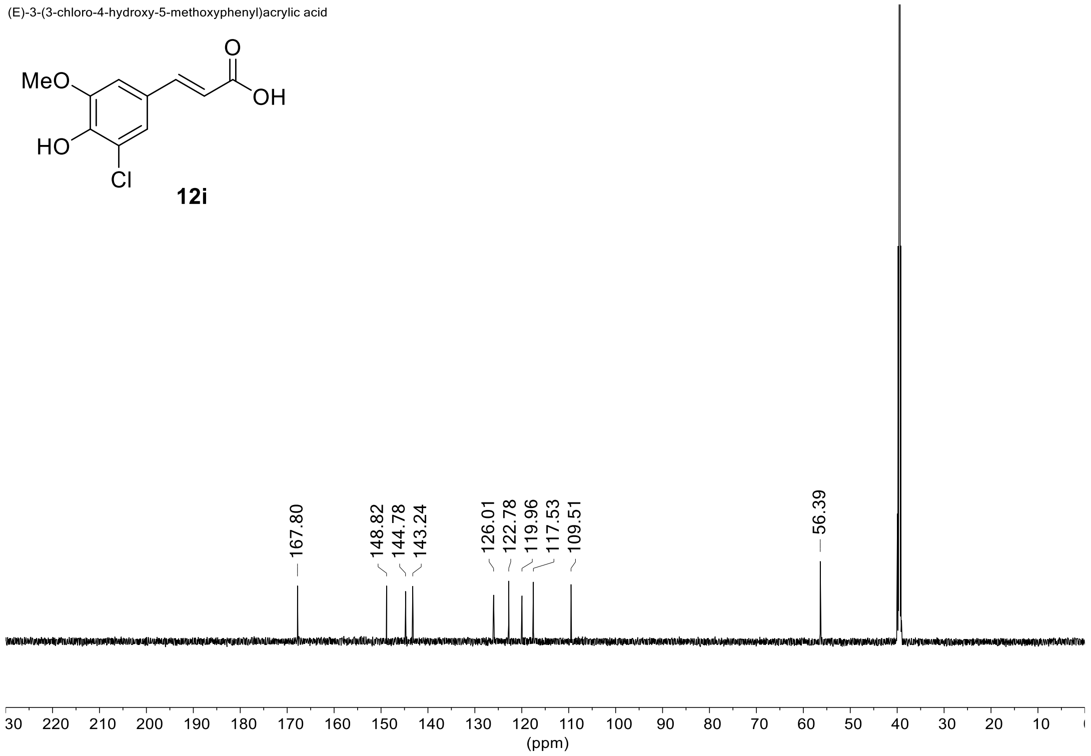

Figure S46. ^{13}C{^{1}H}-NMR spectrum (151 MHz) of compound **12i**.

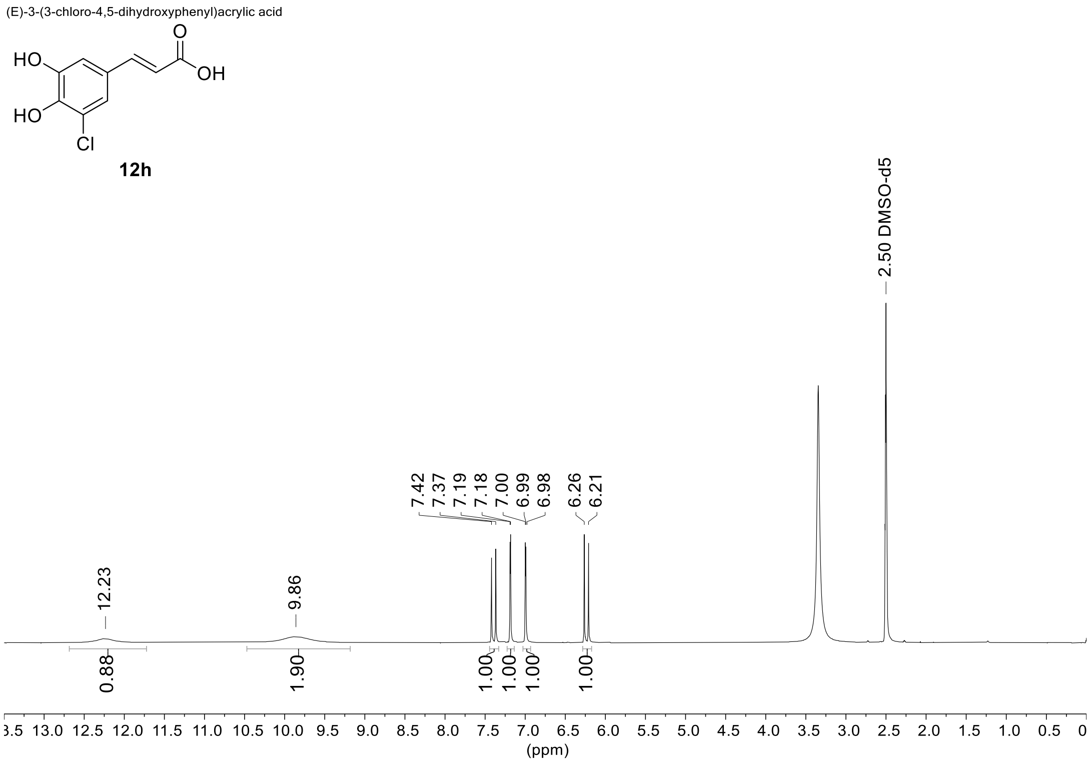

Figure S47. ^{1}H-NMR spectrum (300 MHz) of compound **12h**.

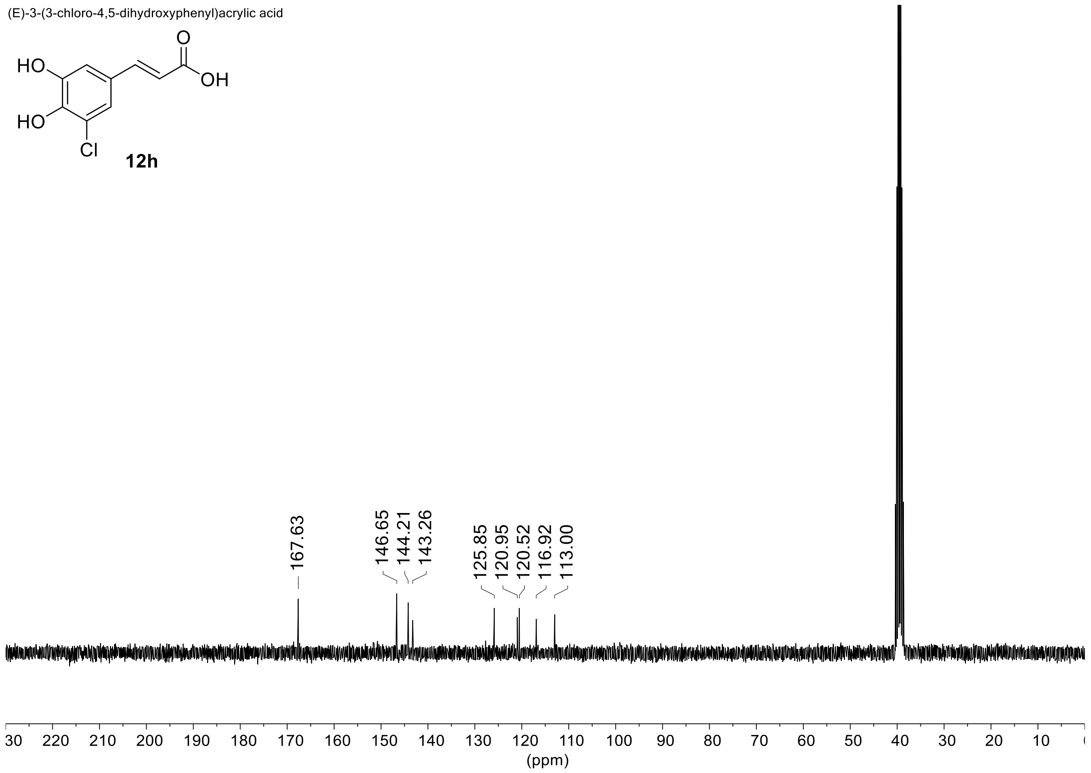

Figure S48. ^{13}C{^{1}H}-NMR spectrum (75.5 MHz) of compound **12h**.

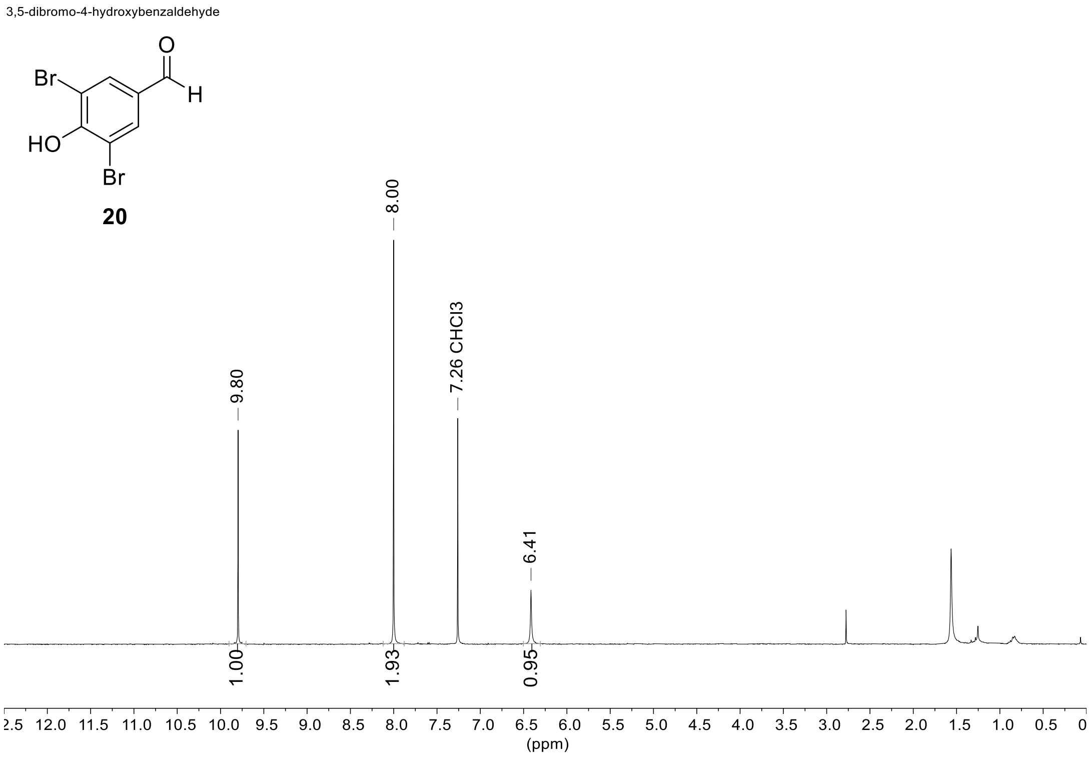

Figure S49. ^{1}H-NMR (300 MHz) spectrum of compound **20**.

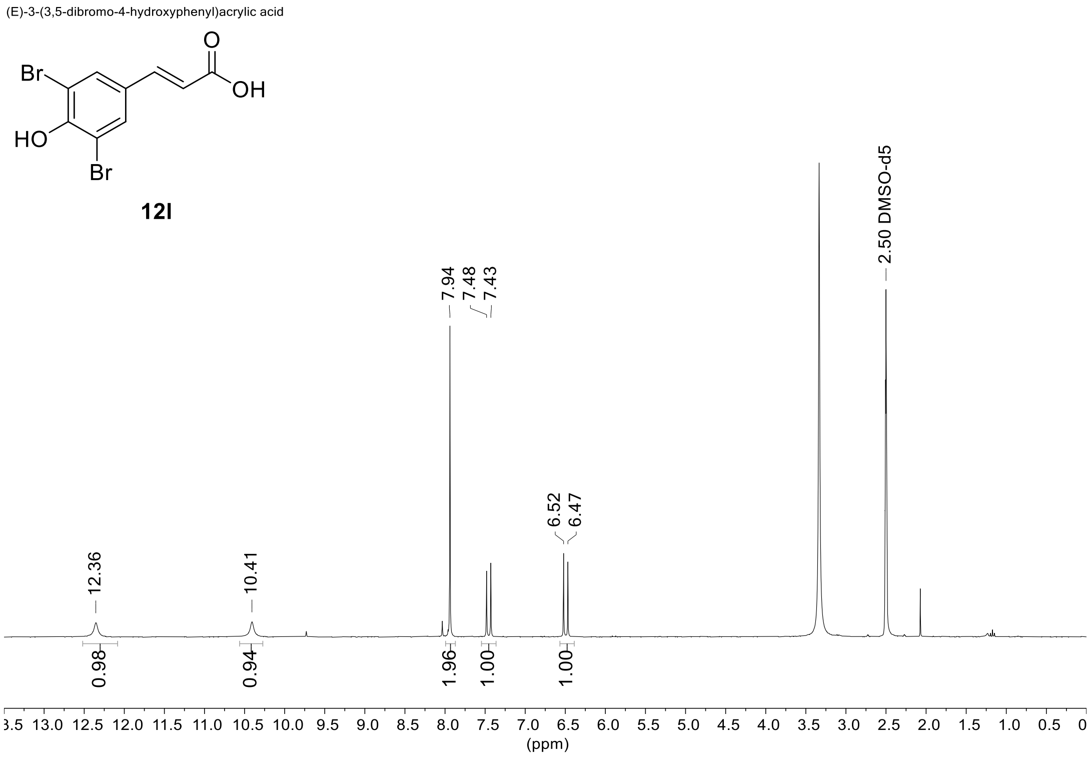

Figure S50. ^{1}H-NMR spectrum (300 MHz) of compound **12l**.

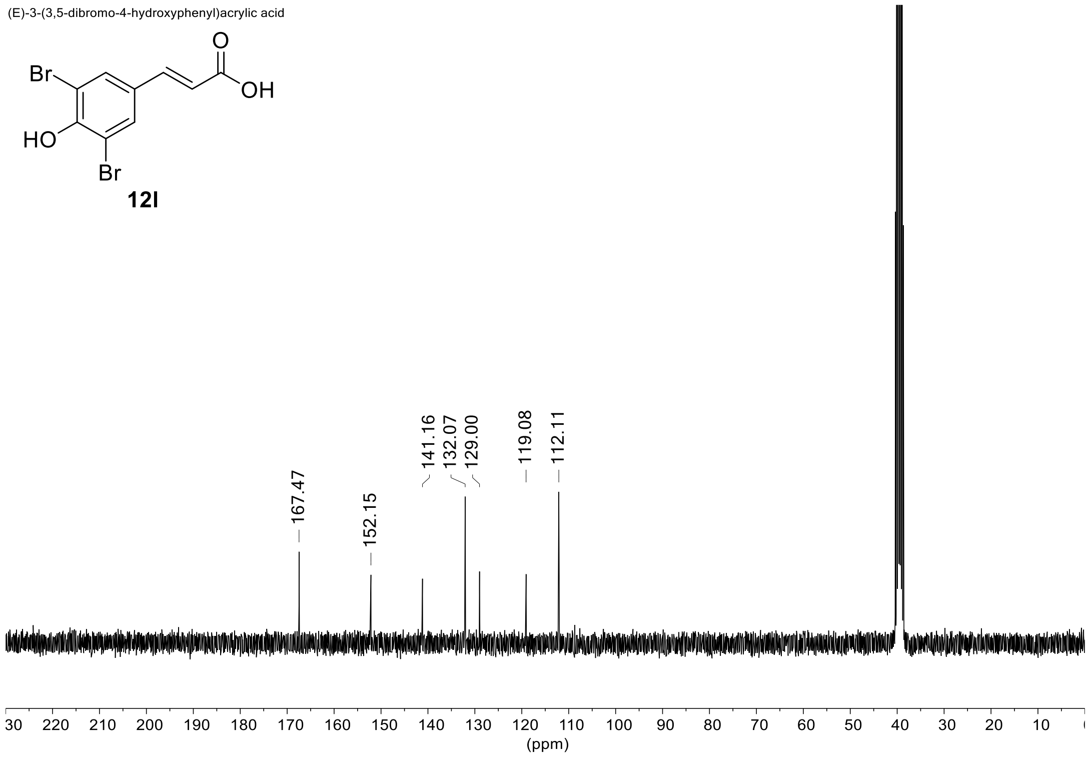

Figure S51. $^{13}C\{^{1}H\}$-NMR spectrum (75.5 MHz) of compound **12l**.

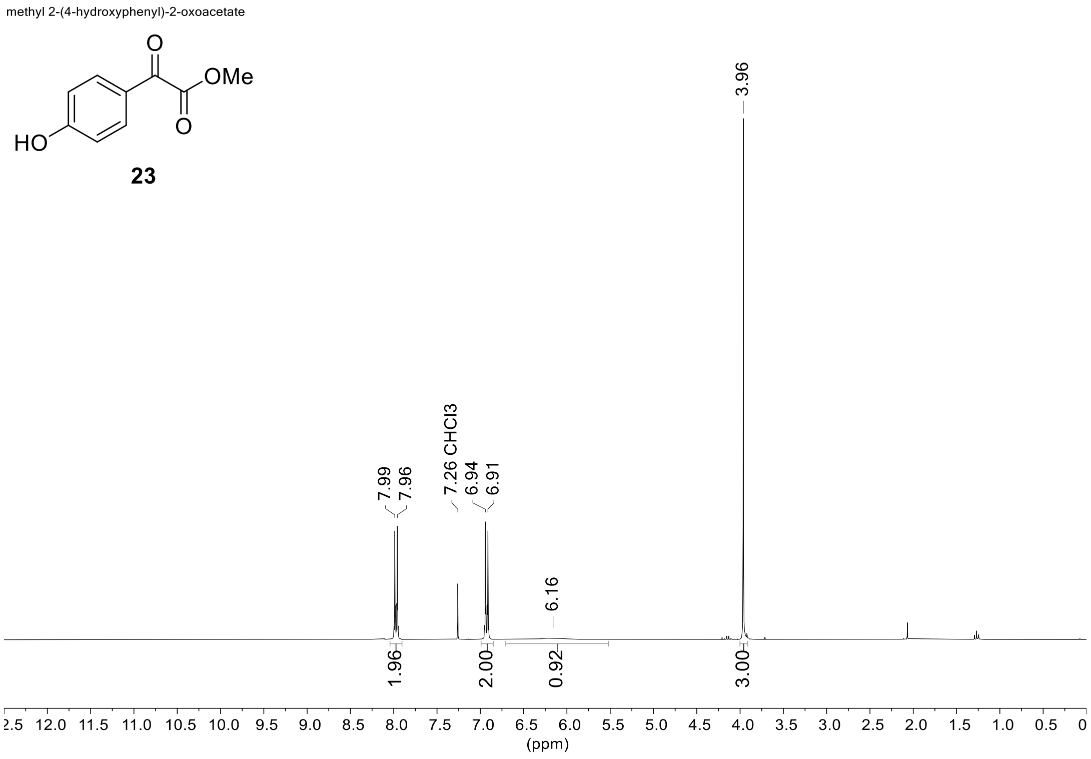

Figure S52. ^{1}H-NMR spectrum (300 MHz) of compound **23**.

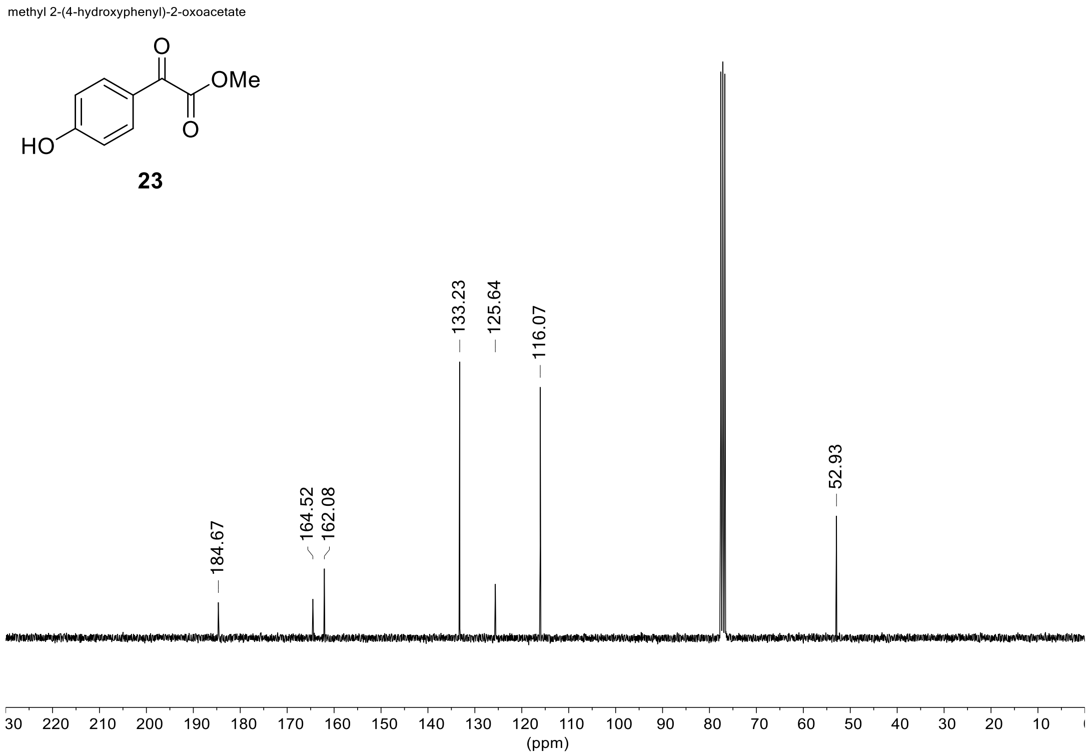

Figure S53. ^{13}C{^{1}H}-NMR spectrum (75.5 MHz) of compound **23**.

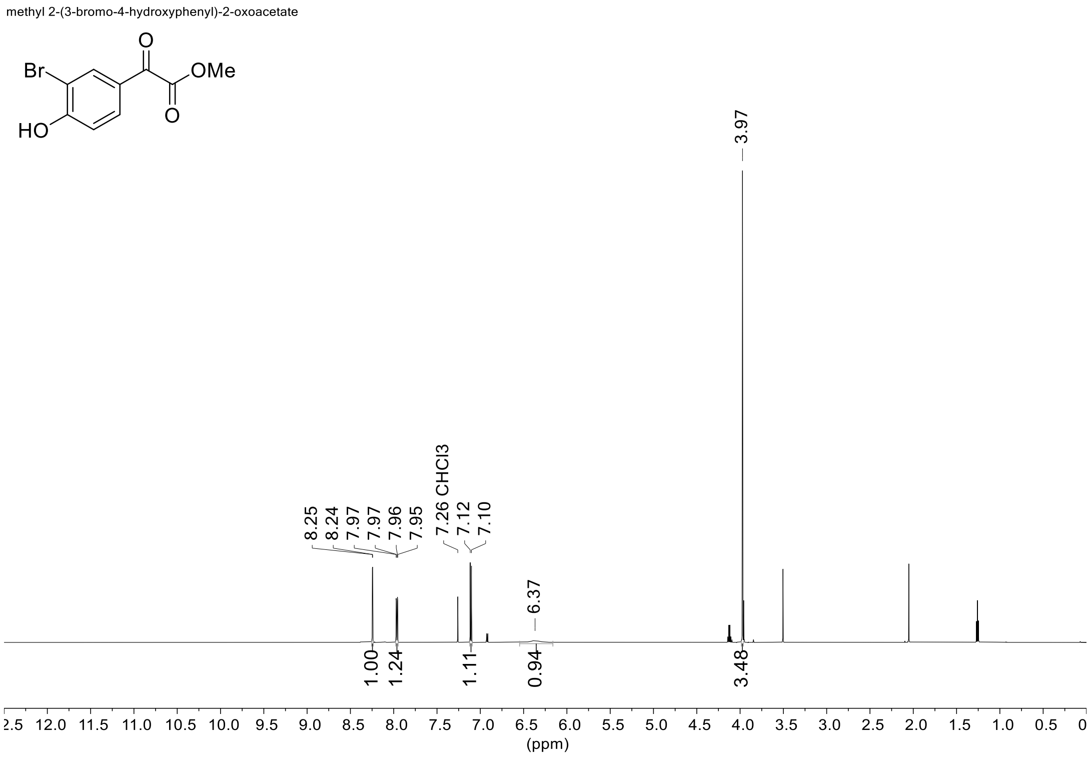

Figure S54. ^{1}H-NMR spectrum (300 MHz) of methyl 2-(3-bromo-4-hydroxyphenyl)-2-oxoacetate.

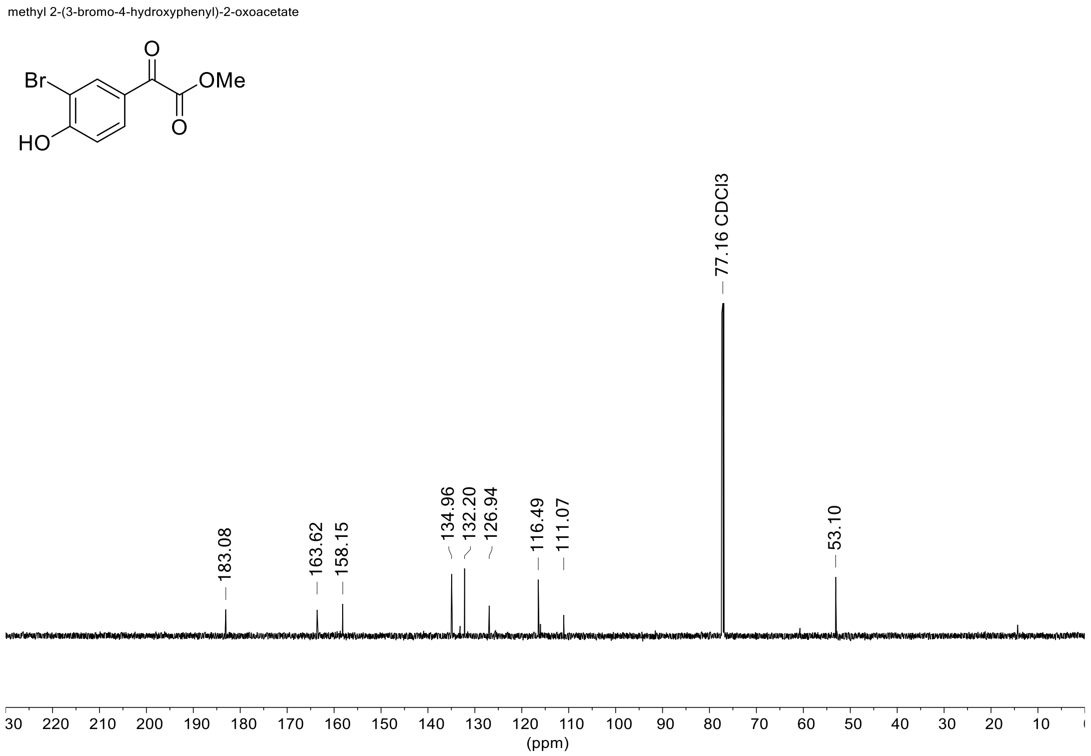

Figure S55. ^{13}C{^{1}H}-NMR spectrum (75.5 MHz) of methyl 2-(3-bromo-4-hydroxyphenyl)-2-oxoacetate.

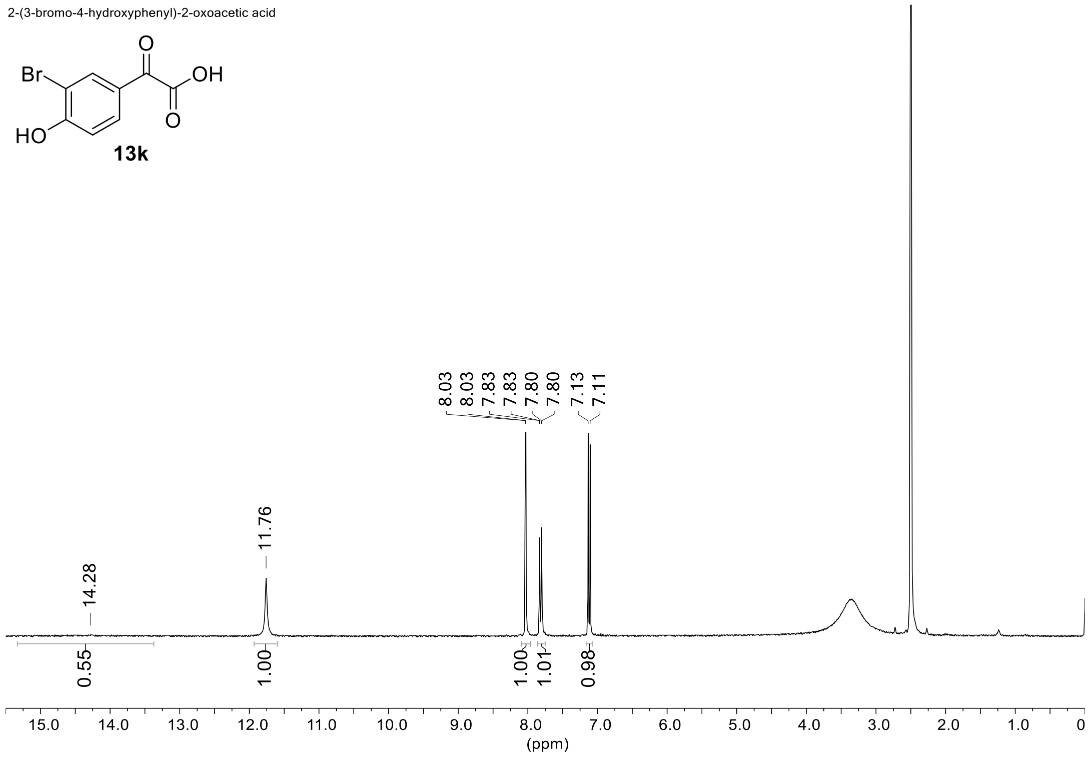

Figure S56. ^{1}H-NMR spectrum (300 MHz) of compound **13k**.

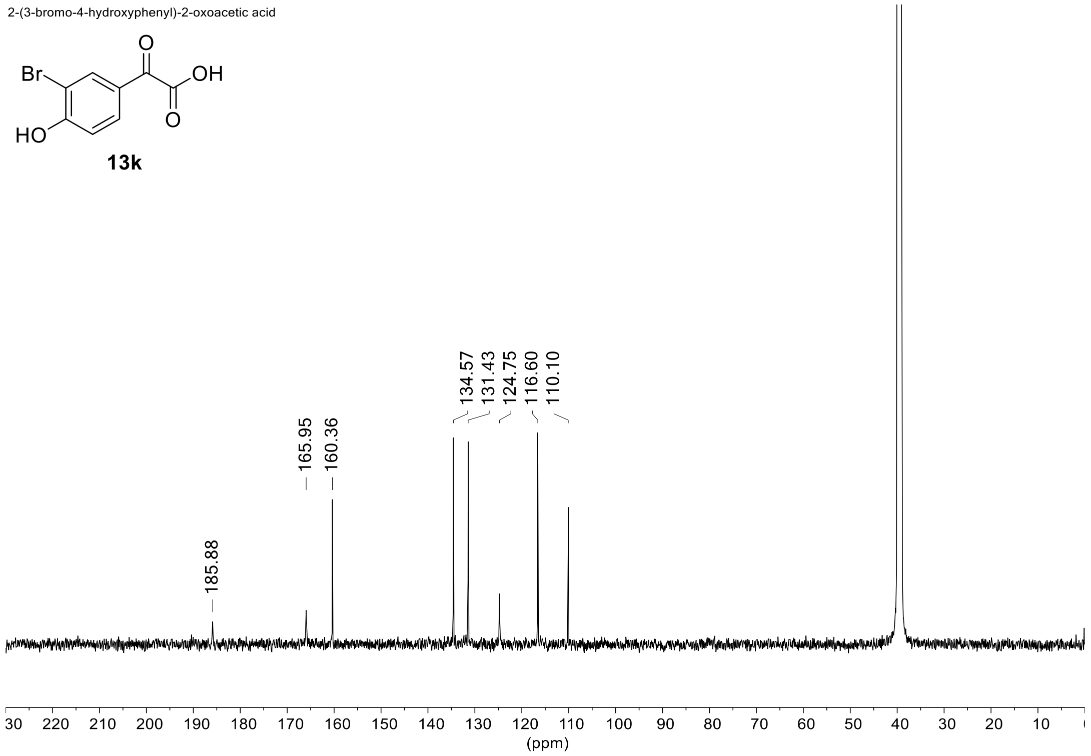

Figure S57. $^{13}C\{^{1}H\}$-NMR spectrum (151 MHz) of compound **13k**.

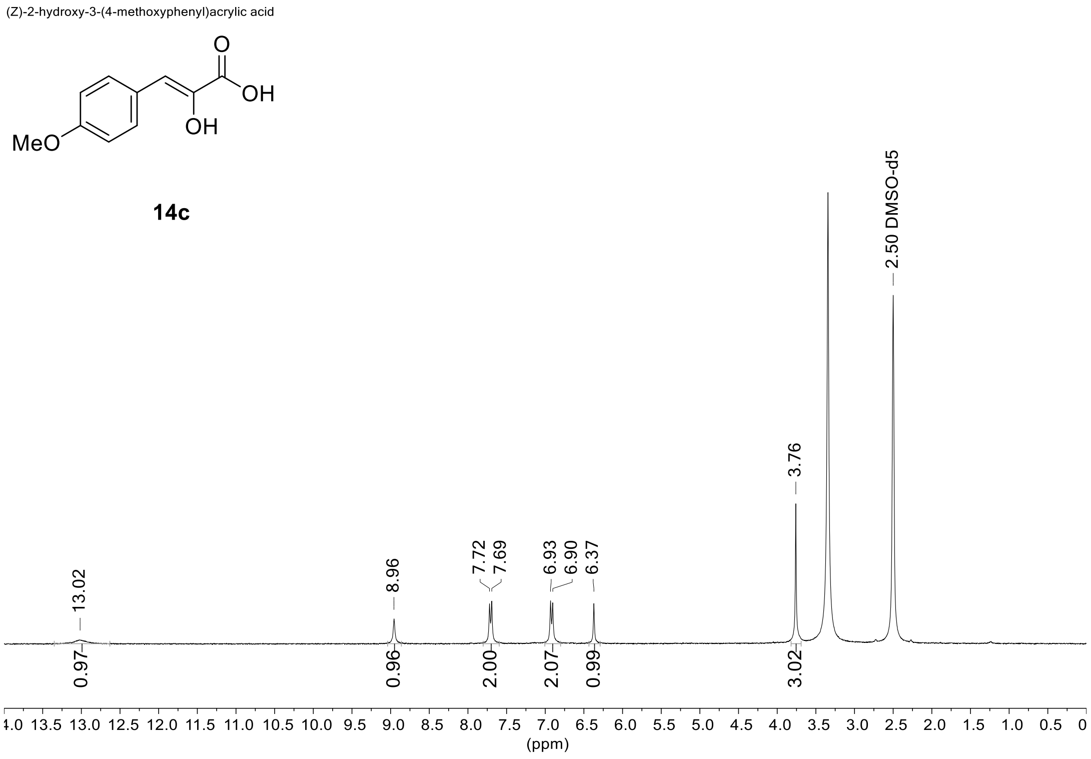

Figure S58. ^{1}H-NMR spectrum (300 MHz) of compound **14c**.

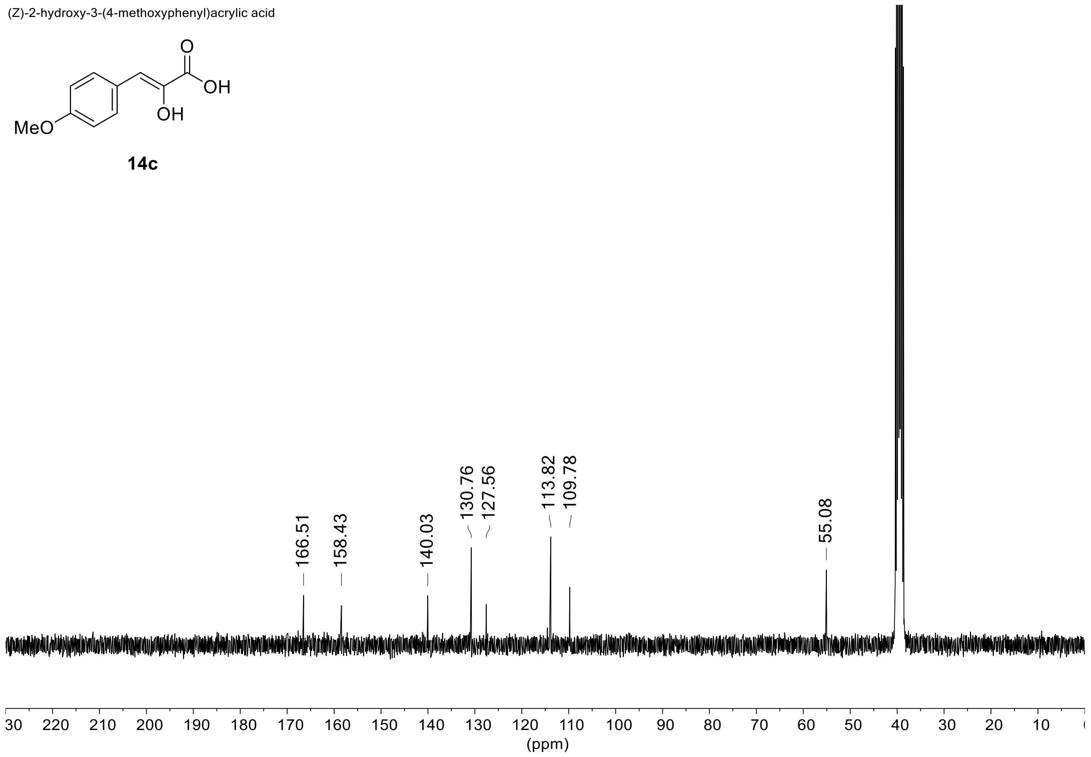

Figure S59. ^{13}C{^{1}H}-NMR spectrum (75.5 MHz) of compound **14c**.

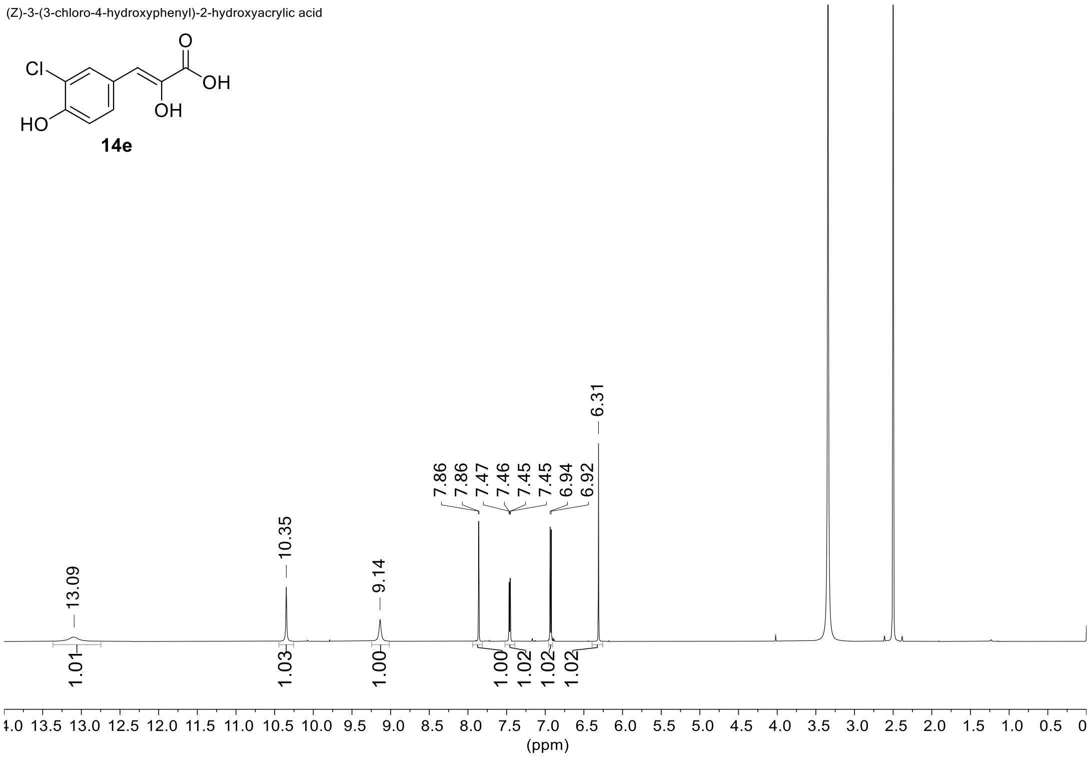

Figure S60. ^{1}H-NMR spectrum (600 MHz) of compound **14e**.

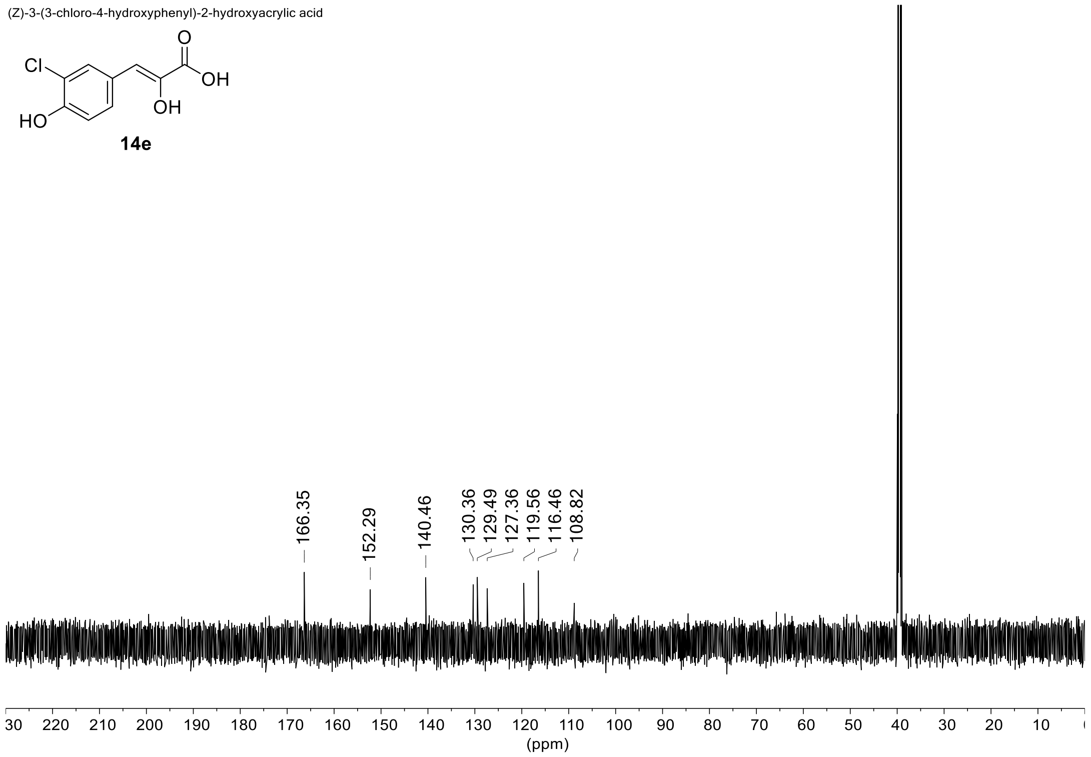

Figure S61. ^{13}C{^{1}H}-NMR spectrum (151 MHz) of compound **14e**.

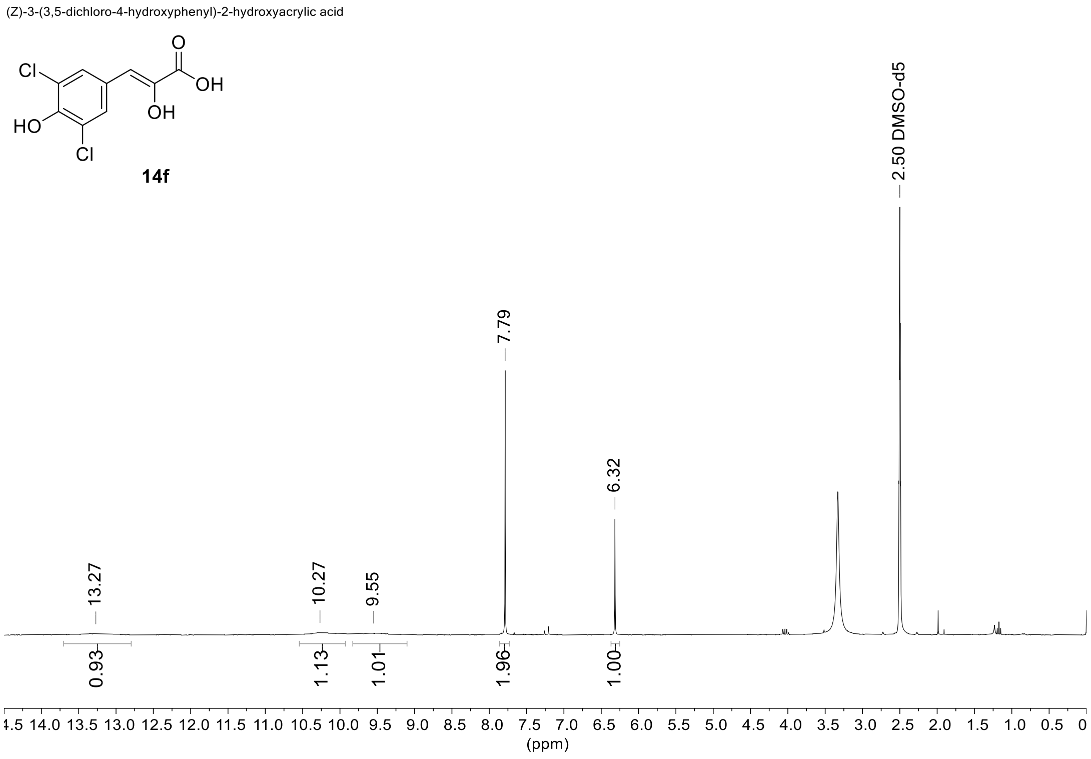

Figure S62. ^{1}H-NMR spectrum (600 MHz) of compound **14f**.

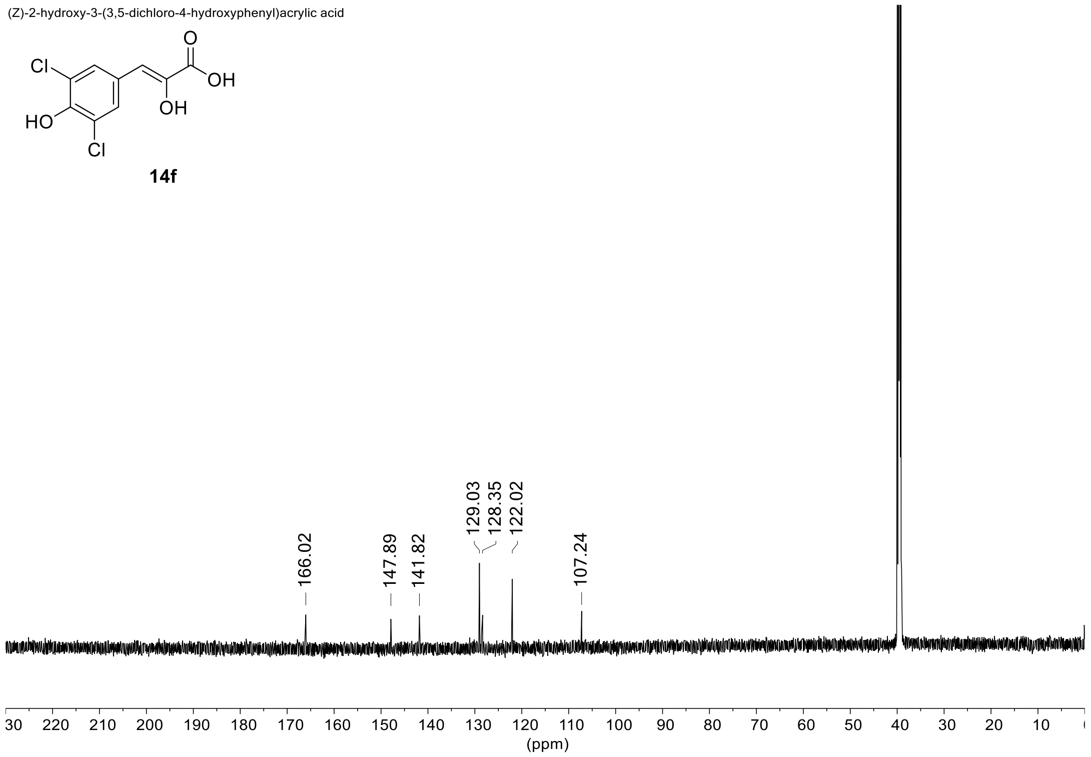

Figure S63. ^{13}C{^{1}H}-NMR spectrum (151 MHz) of compound **14f**.

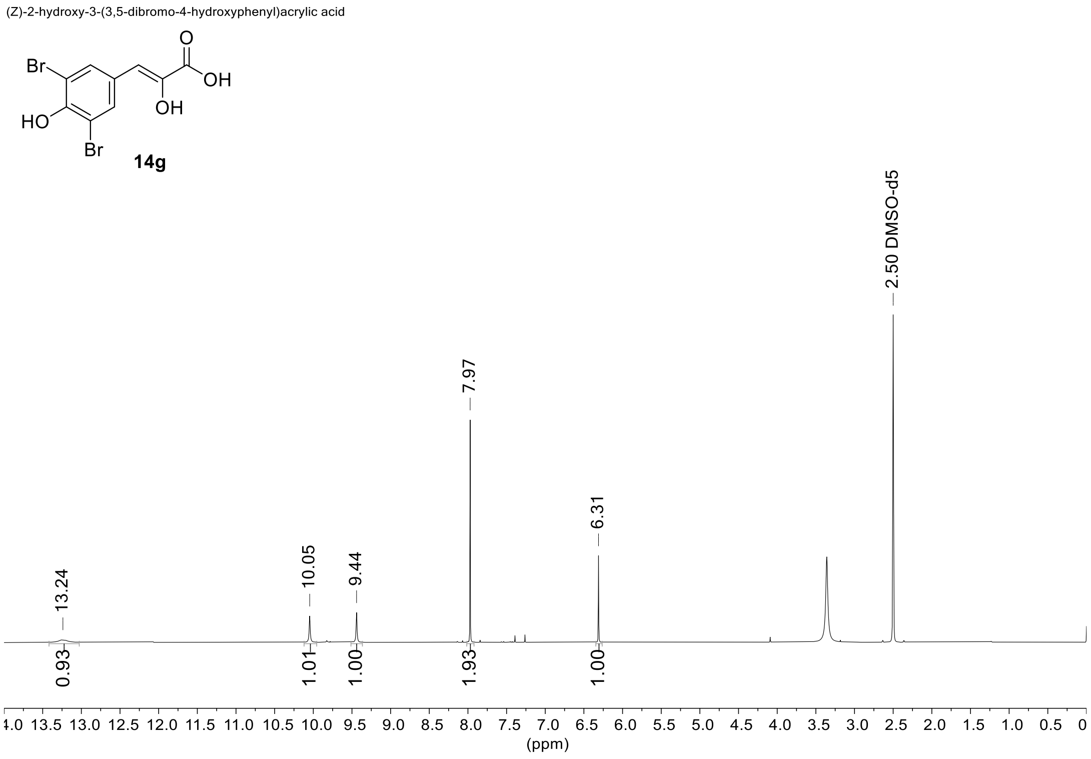

Figure S64. ^{1}H-NMR spectrum (500 MHz) of compound **14g**.

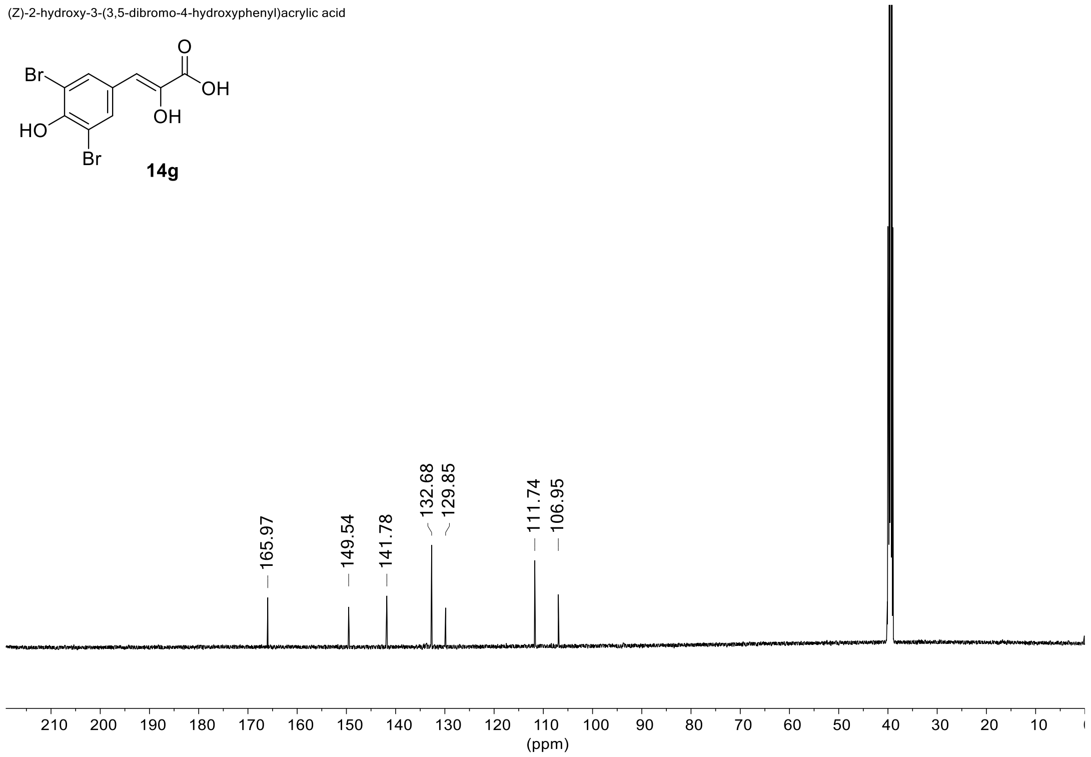

Figure S65. ^{13}C{^{1}H}-NMR spectrum (126 MHz) of compound **14g**.

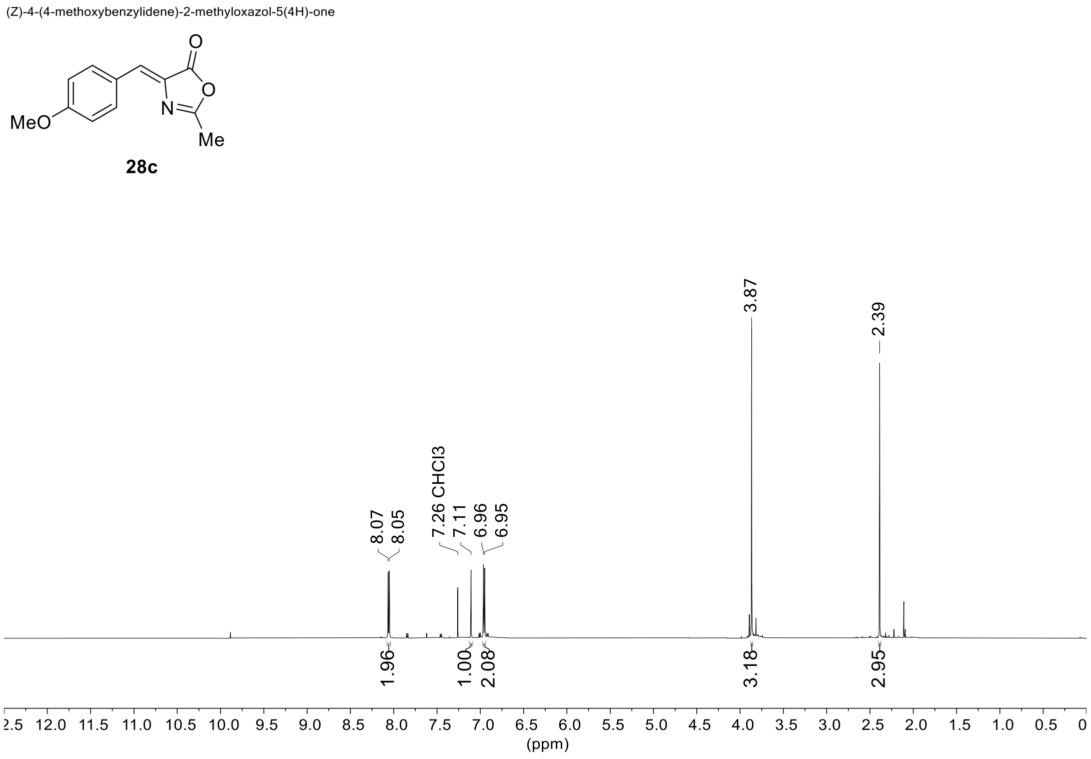

Figure S66. ^{1}H-NMR spectrum (300 MHz) of compound **28c**.

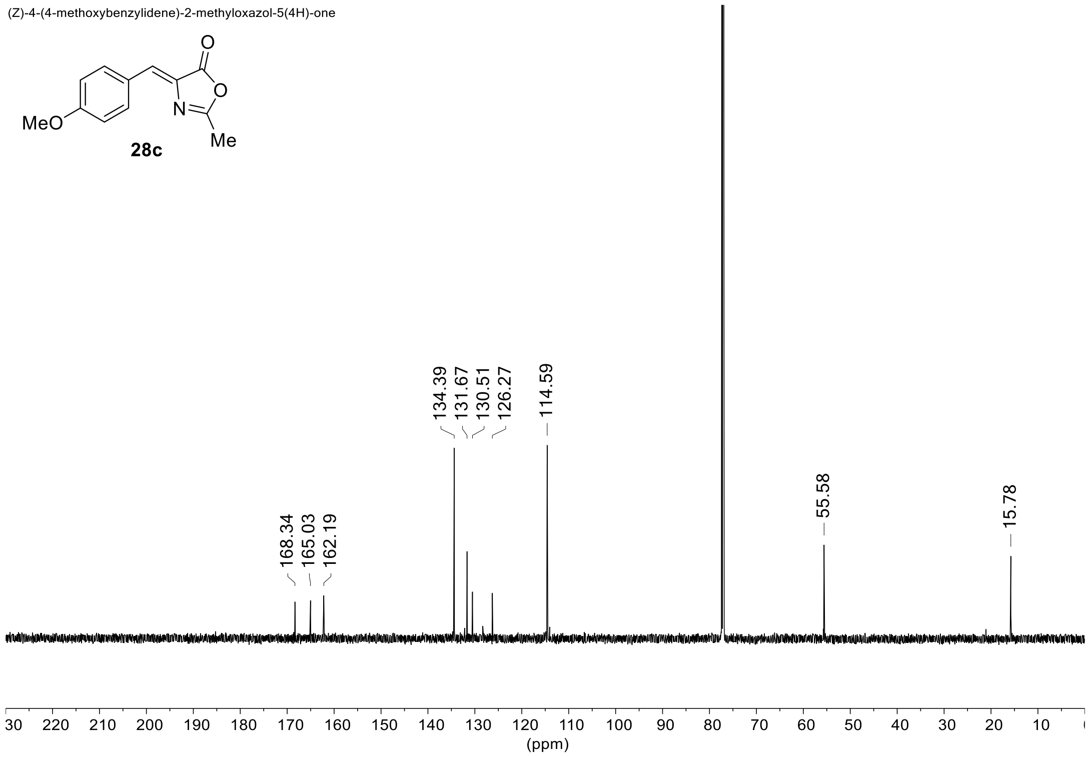

Figure S67. $^{13}C\{^{1}H\}$-NMR spectrum (75.5 MHz) of compound **28c**.

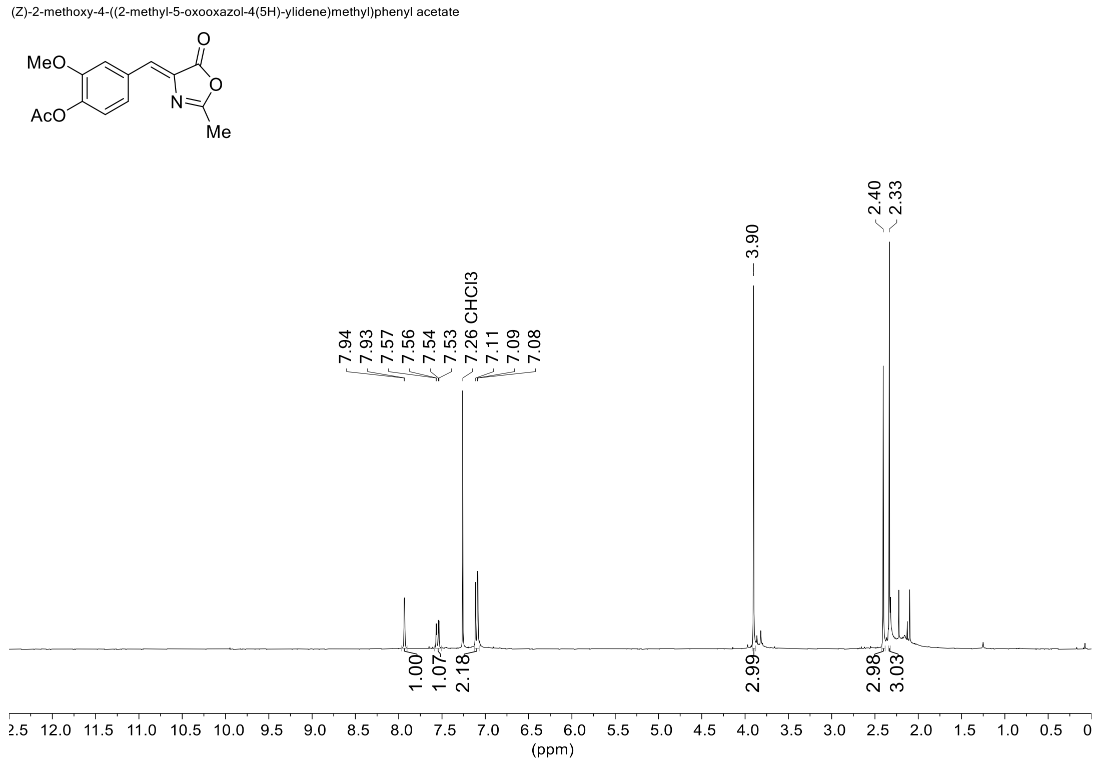

Figure S68. ^{1}H-NMR spectrum (300 MHz) of (*Z*)-2-methoxy-4-((2-methyl-5-oxooxazol-4(5*H*)-ylidene)methyl) phenyl acetate.

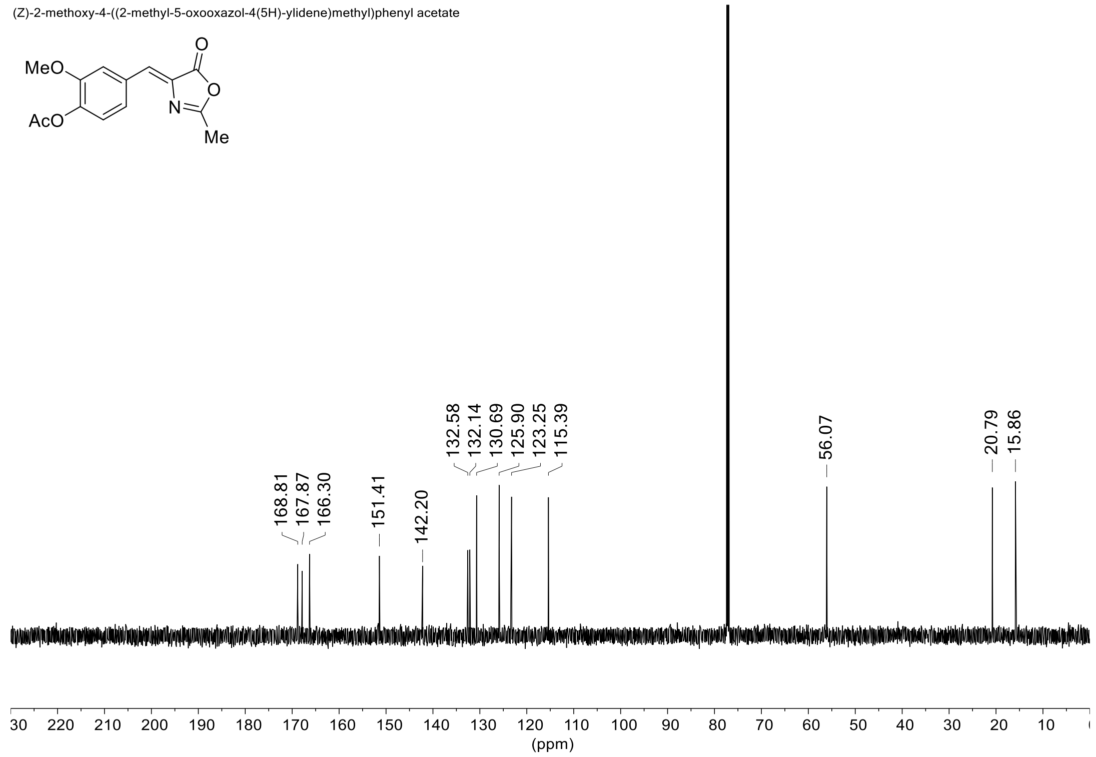

Figure S69. ^{13}C{^{1}H}-NMR spectrum (151 MHz) of (*Z*)-2-methoxy-4-((2-methyl-5-oxooxazol-4(5*H*)-ylidene)methyl)phenyl acetate.

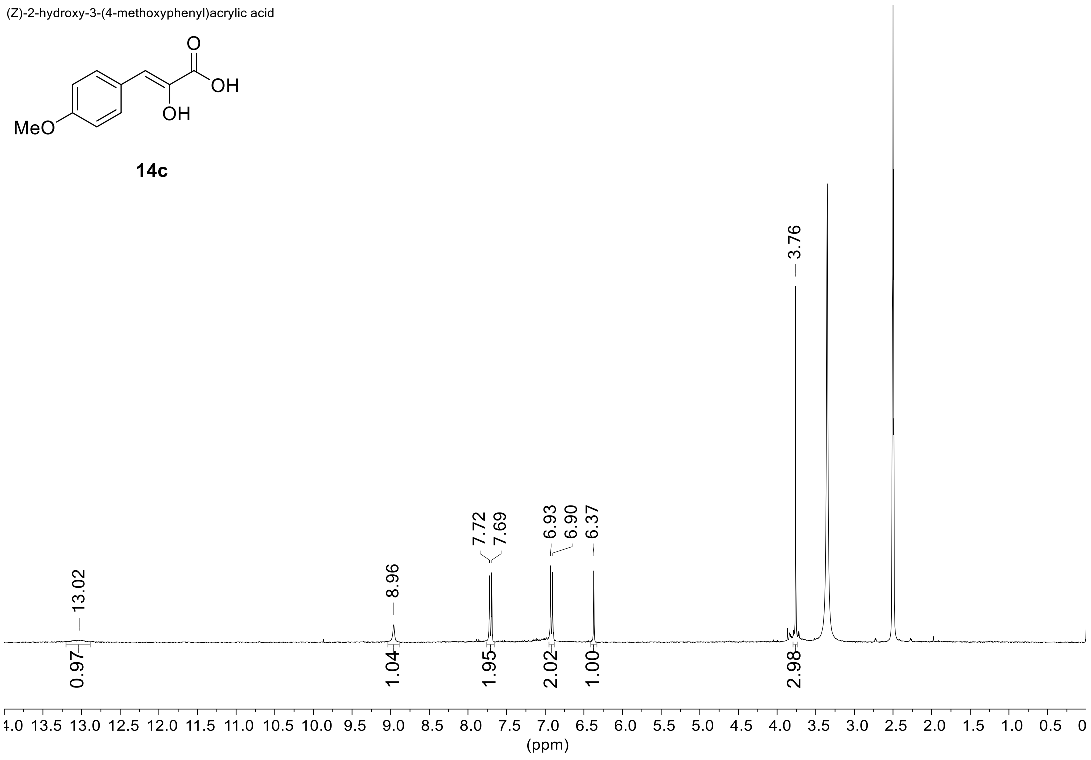

Figure S70. ^{1}H-NMR spectrum (300 MHz) of compound **14c**, synthesized *via* Erlenmeyer oxazolone synthesis.

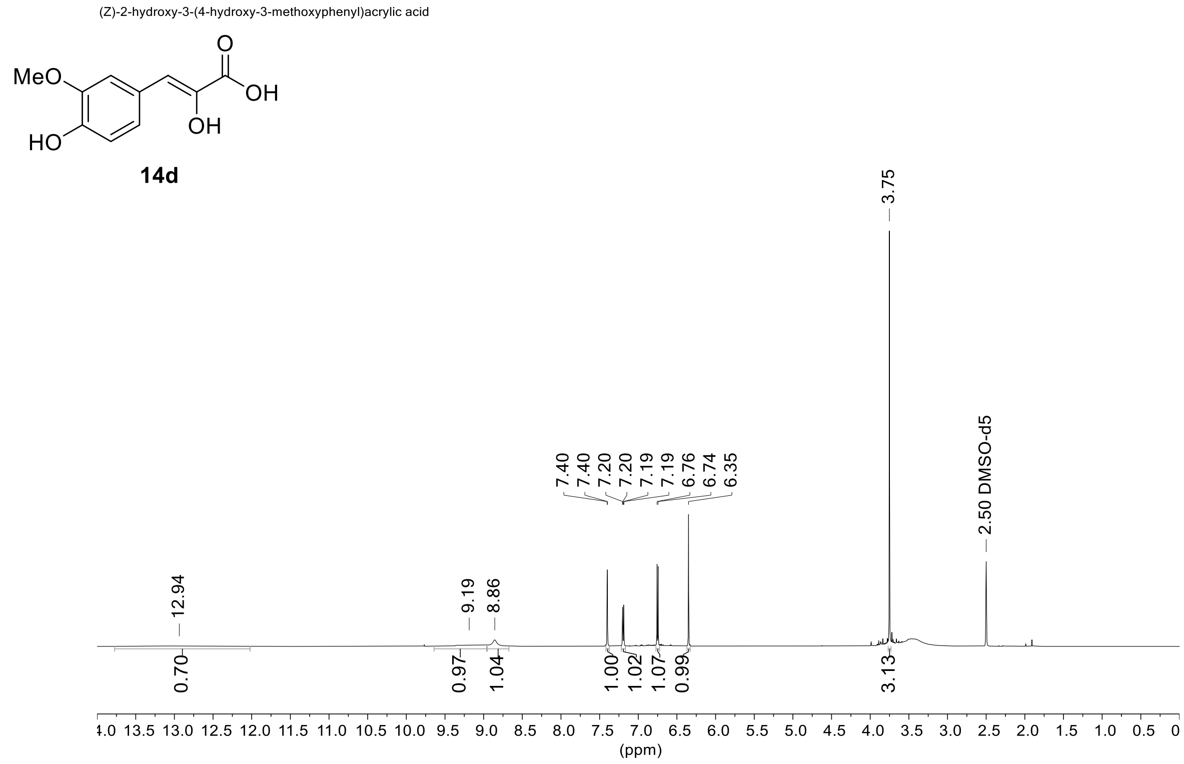

Figure S71. ^{1}H-NMR spectrum (600 MHz) of compound **14d**.

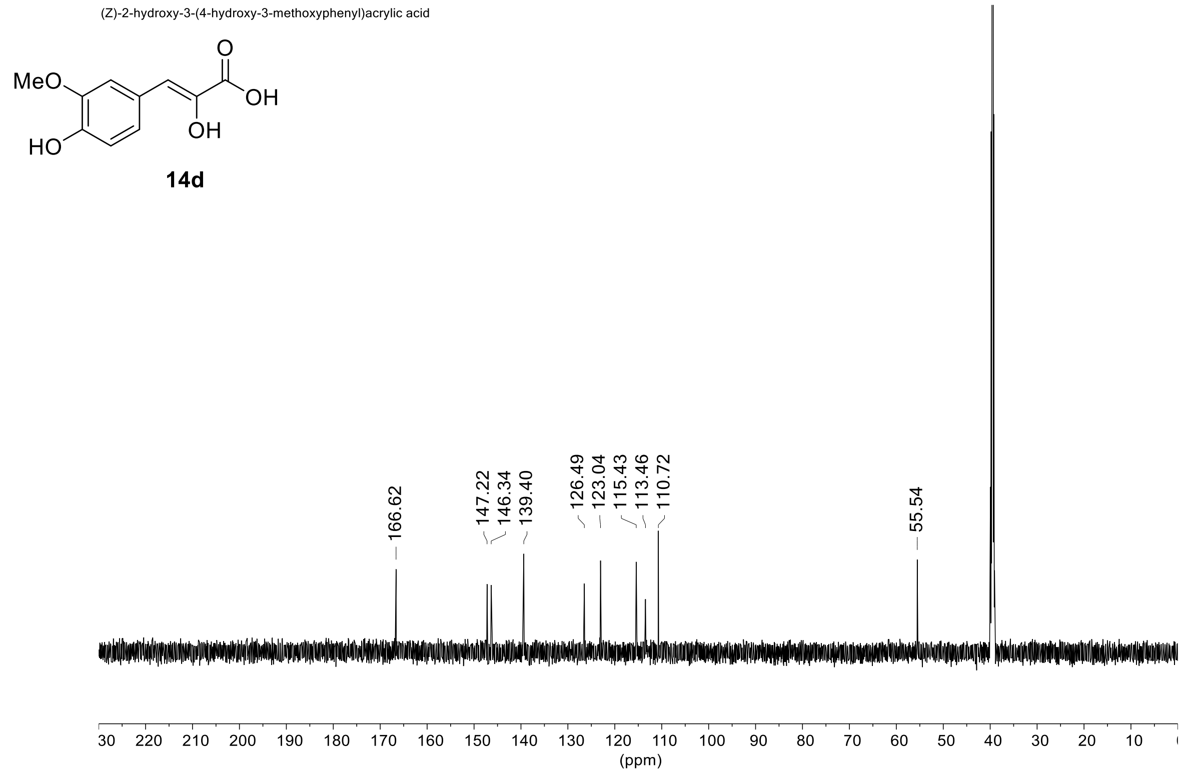

Figure S72. ^{13}C{^{1}H}-NMR spectrum (151 MHz) of compound **14d**.

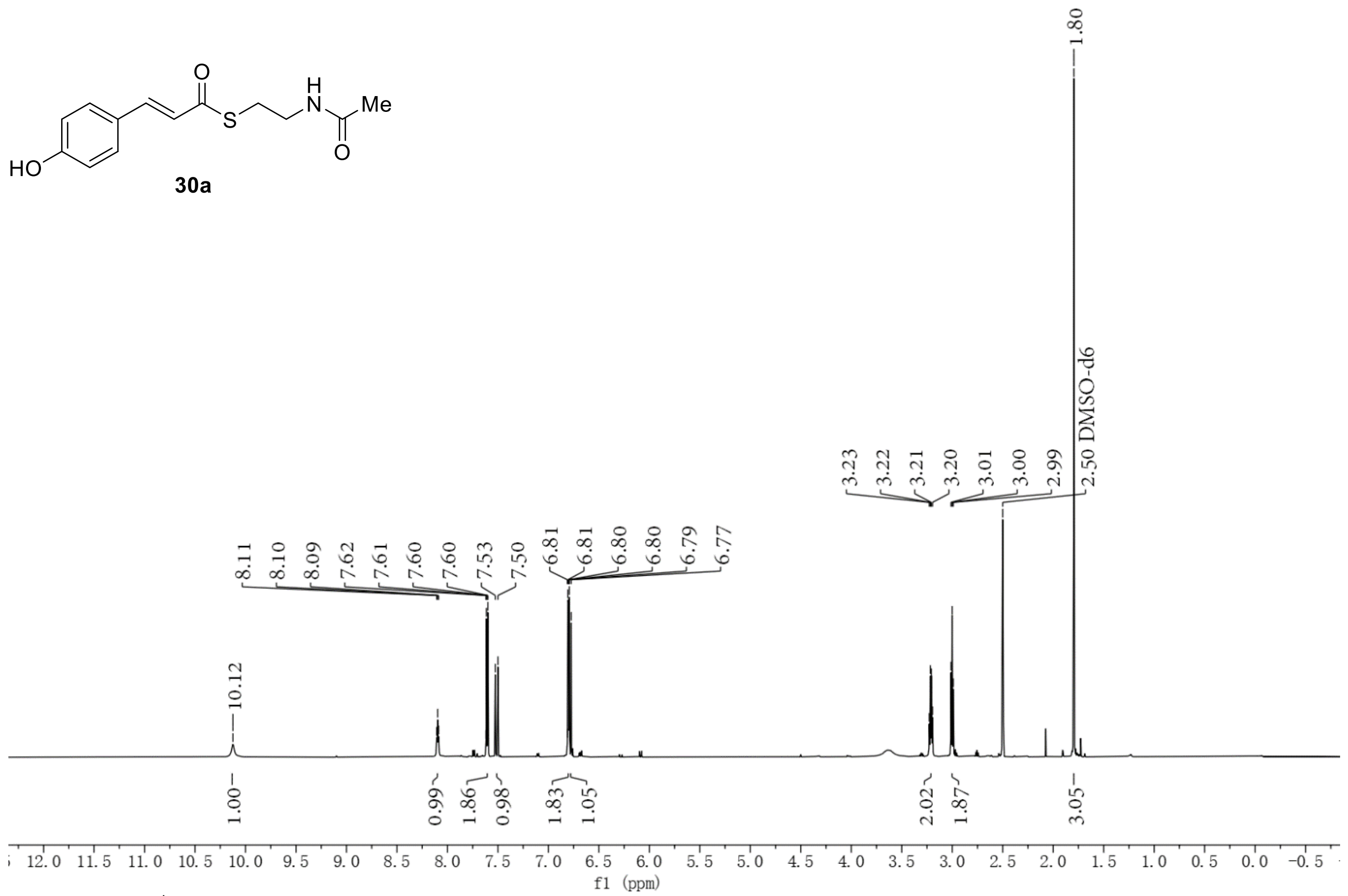

Figure S73. ^{1}H-NMR spectrum (600 MHz) of compound **30a**.

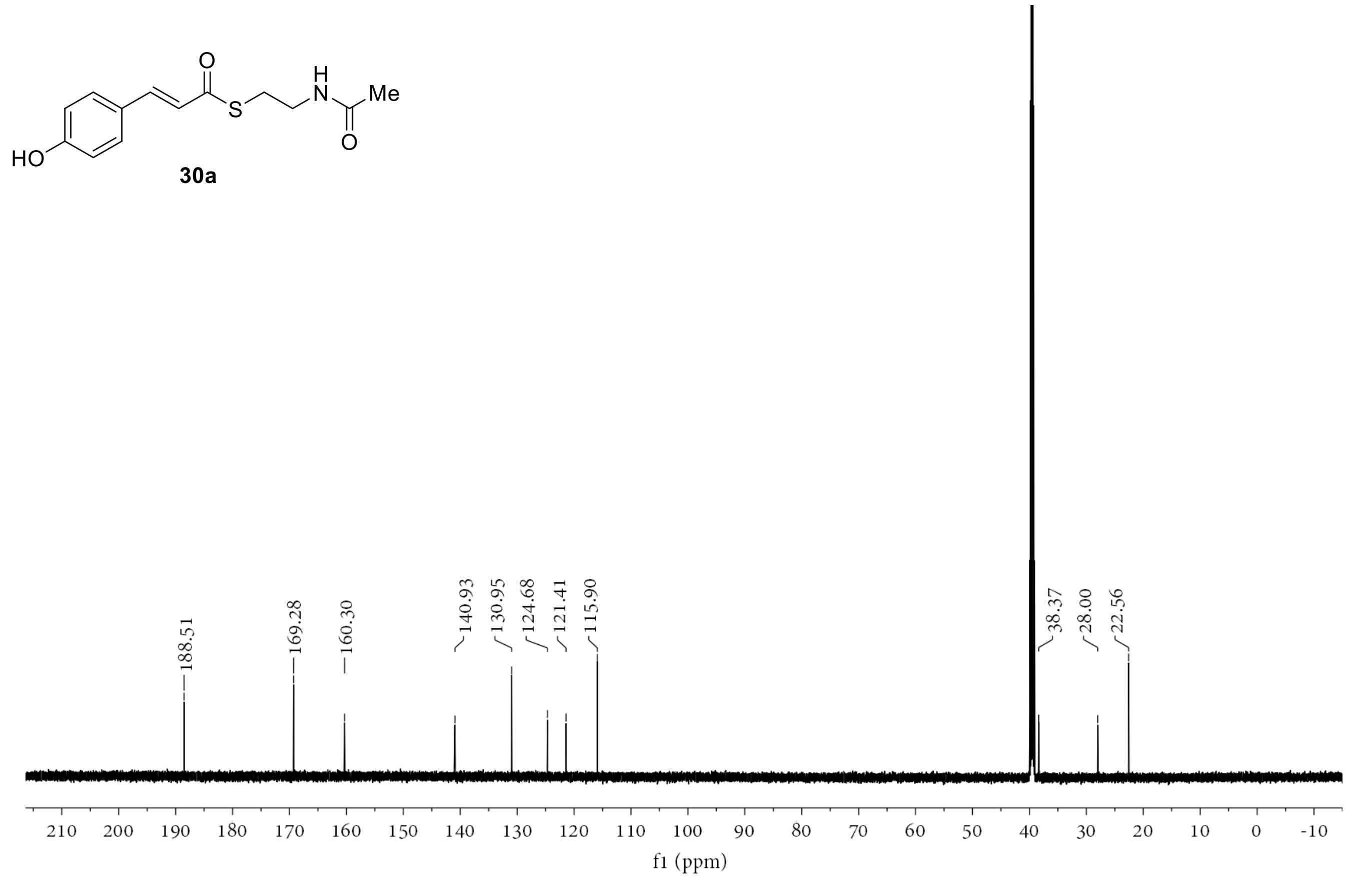

Figure S74. $^{13}C\{^{1}H\}$-NMR spectrum (151 MHz) of compound **30a**.

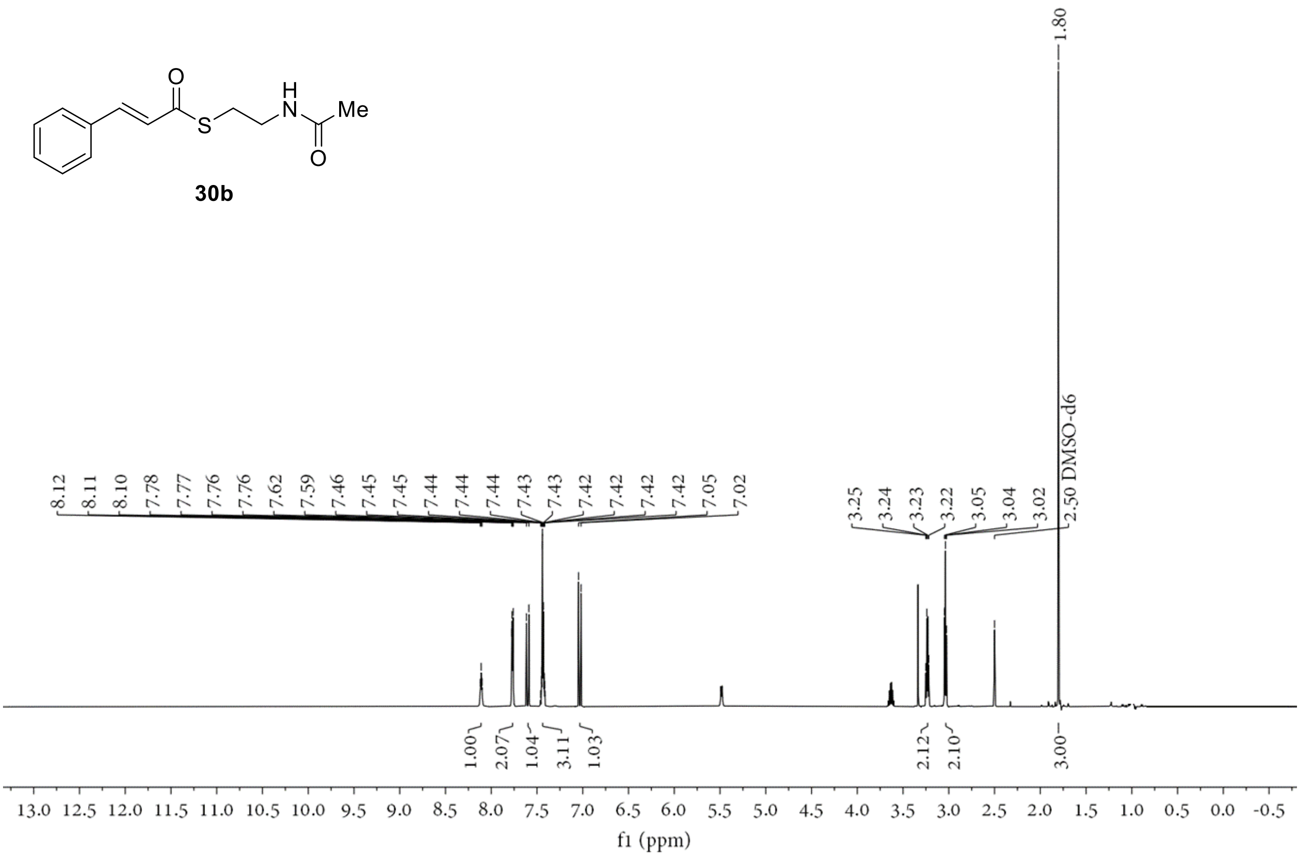

Figure S75. ^{1}H-NMR spectrum (600 MHz) of compound **30b**.

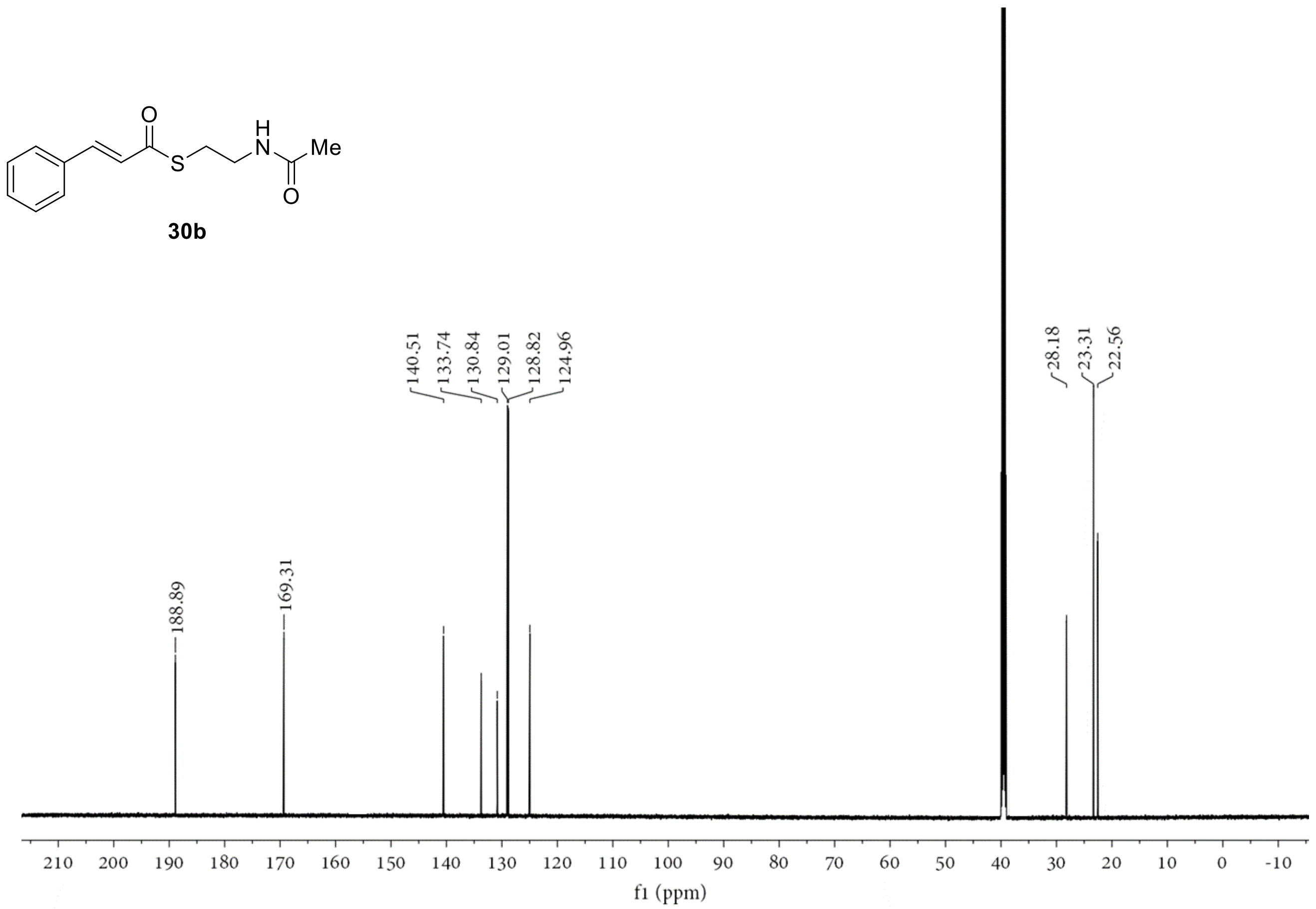

Figure S76. $^{13}C\{^{1}H\}$-NMR spectrum (151 MHz) of compound **30b**.

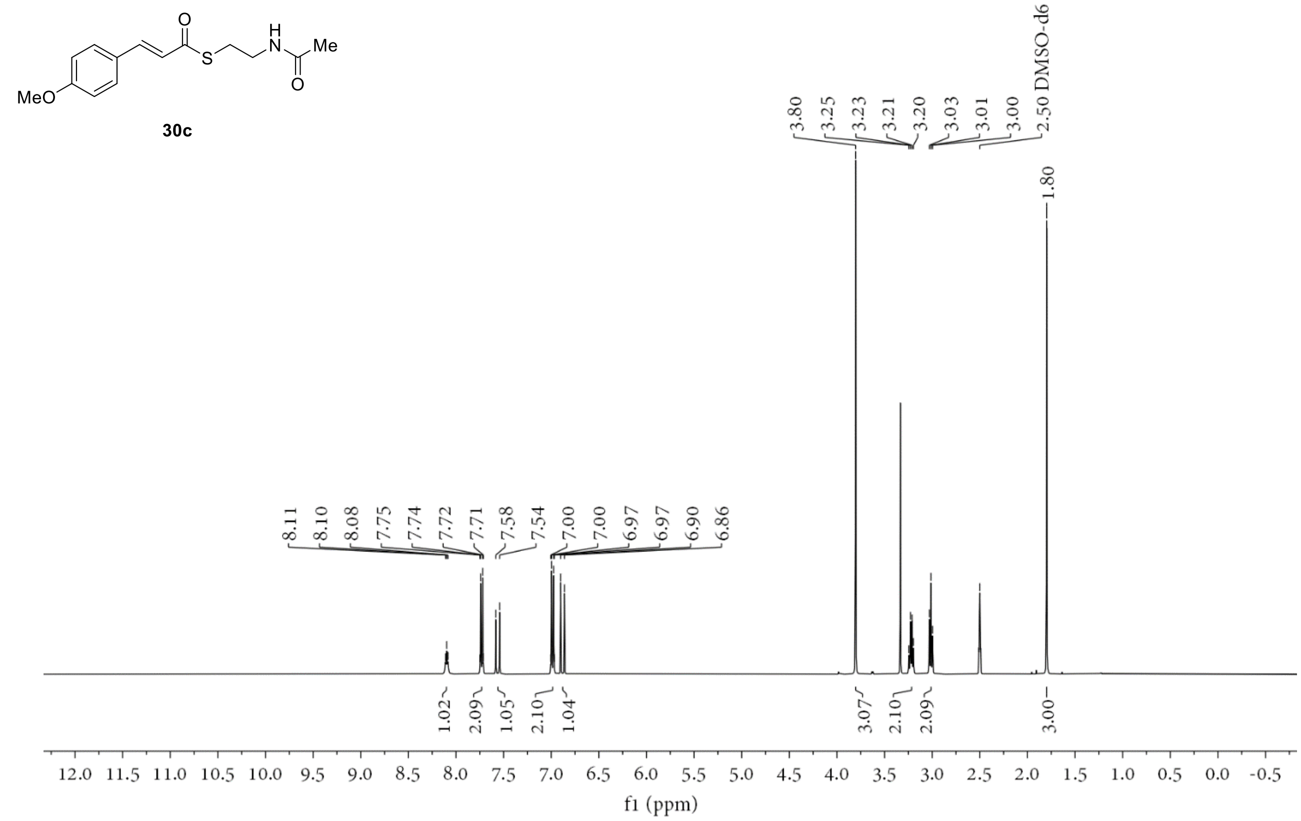

Figure S77. ^{1}H-NMR spectrum (600 MHz) of compound **30c**.

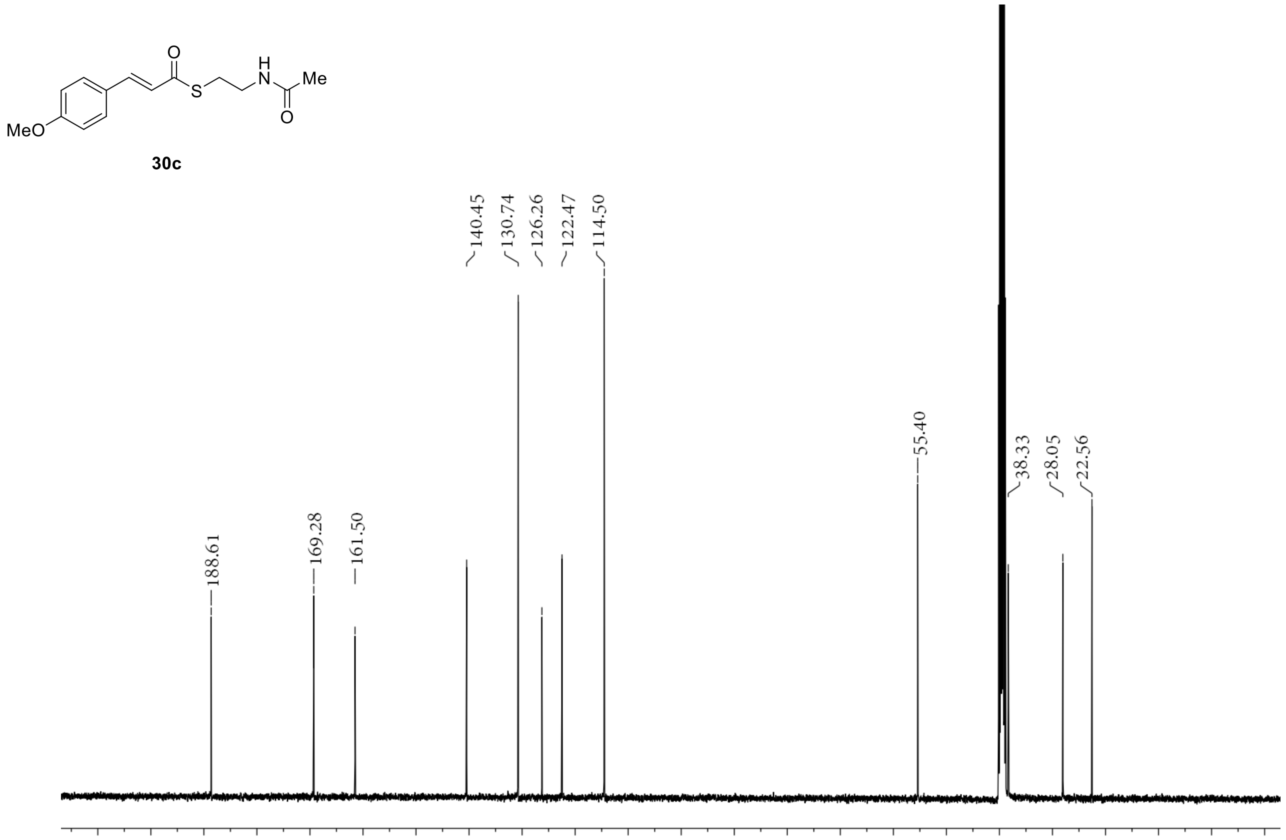

Figure S78. ^{13}C{^{1}H}-NMR spectrum (151 MHz) of compound **30c**.

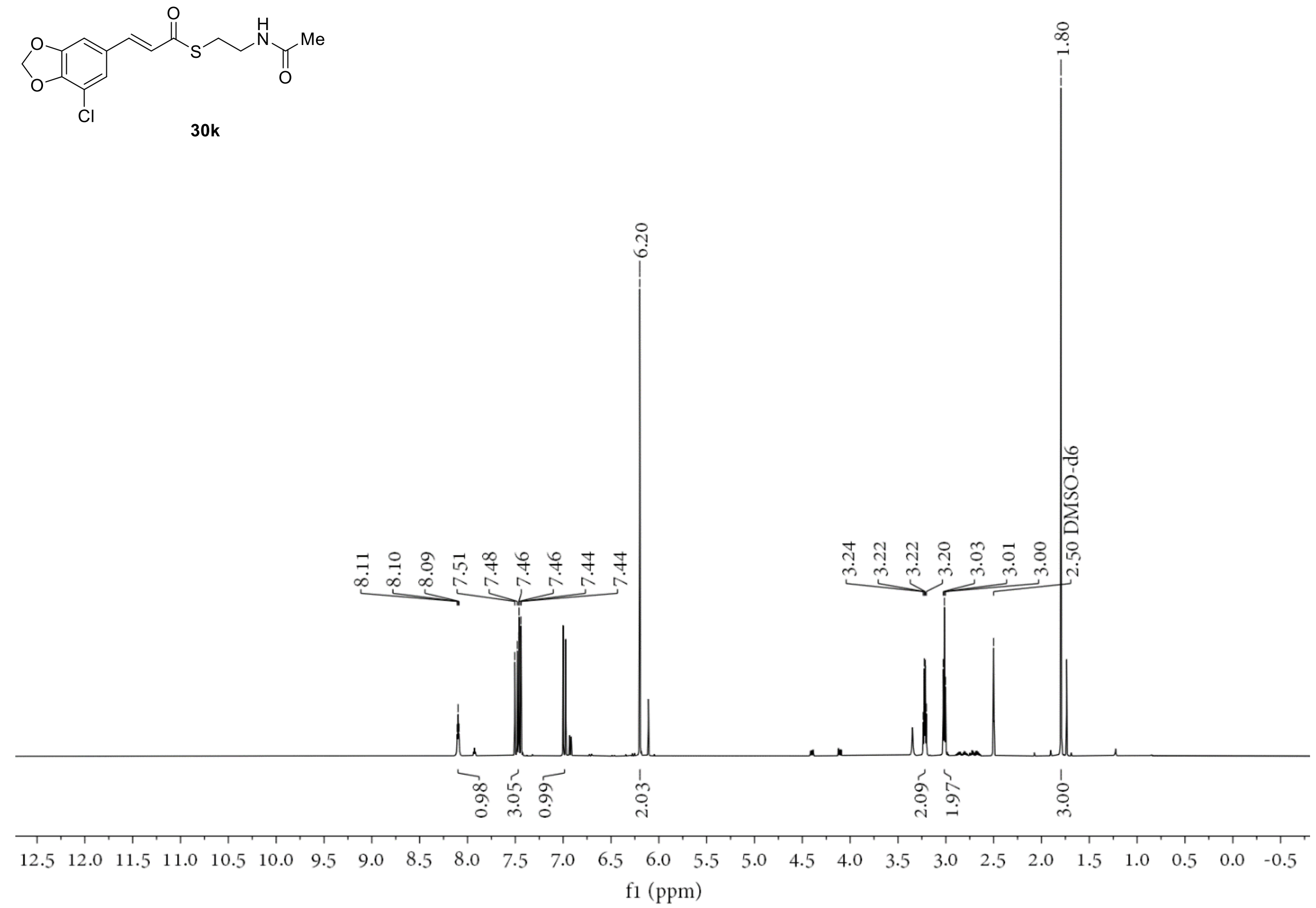

Figure S79. ^{1}H-NMR spectrum (600 MHz) of compound **30k**.

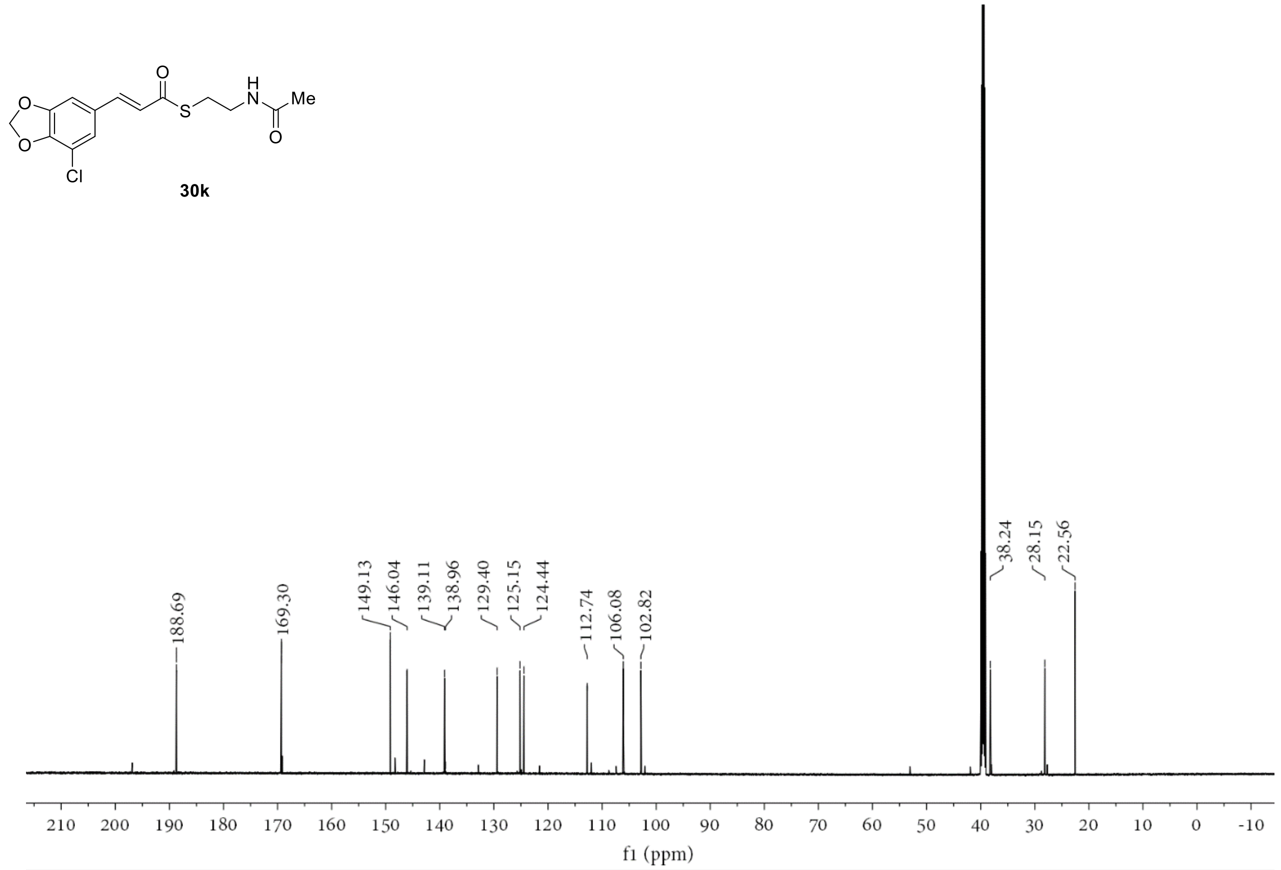

Figure S80. $^{13}C\{^{1}H\}$-NMR spectrum (151 MHz) of compound **30k**.

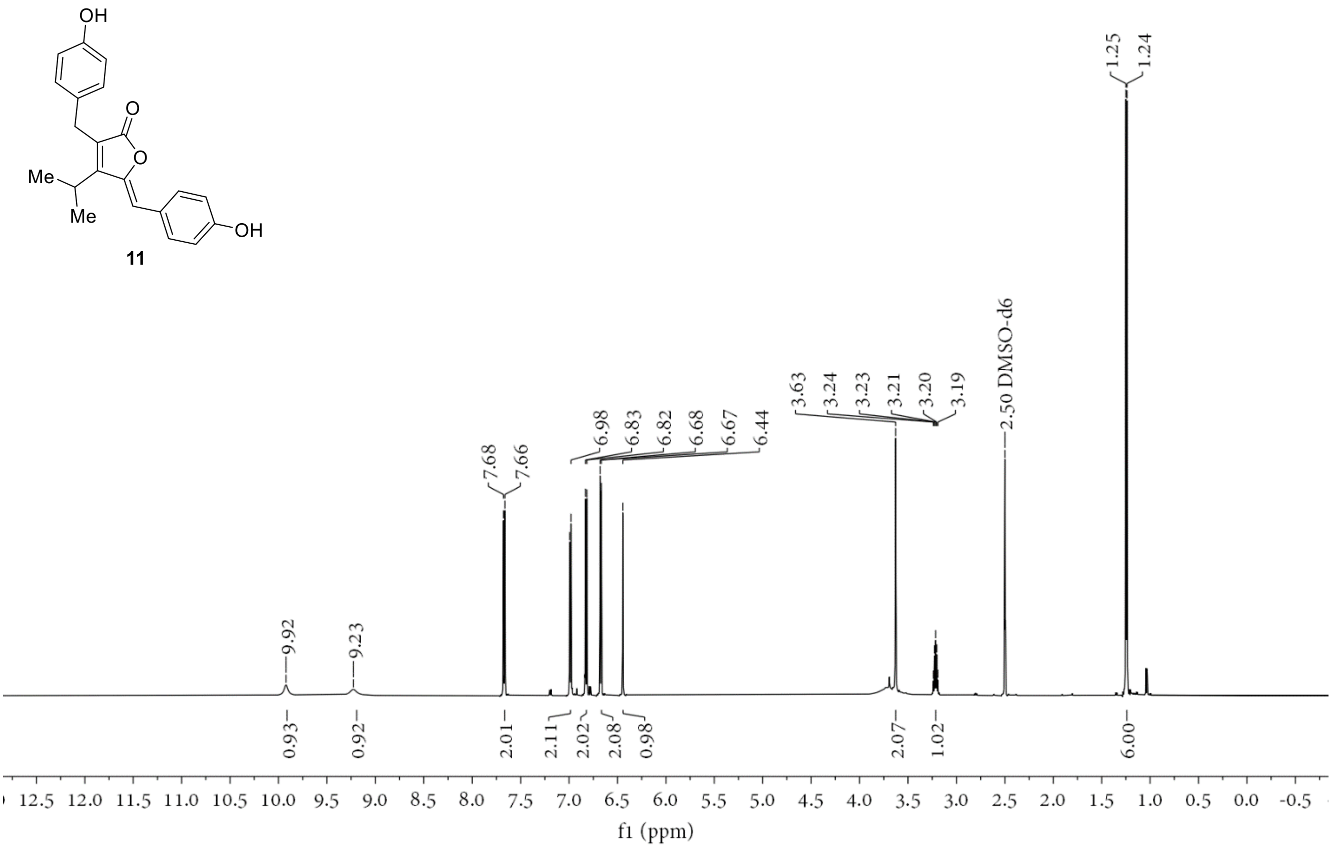

Figure S81. ^{1}H-NMR spectrum (600 MHz) of compound **11**.

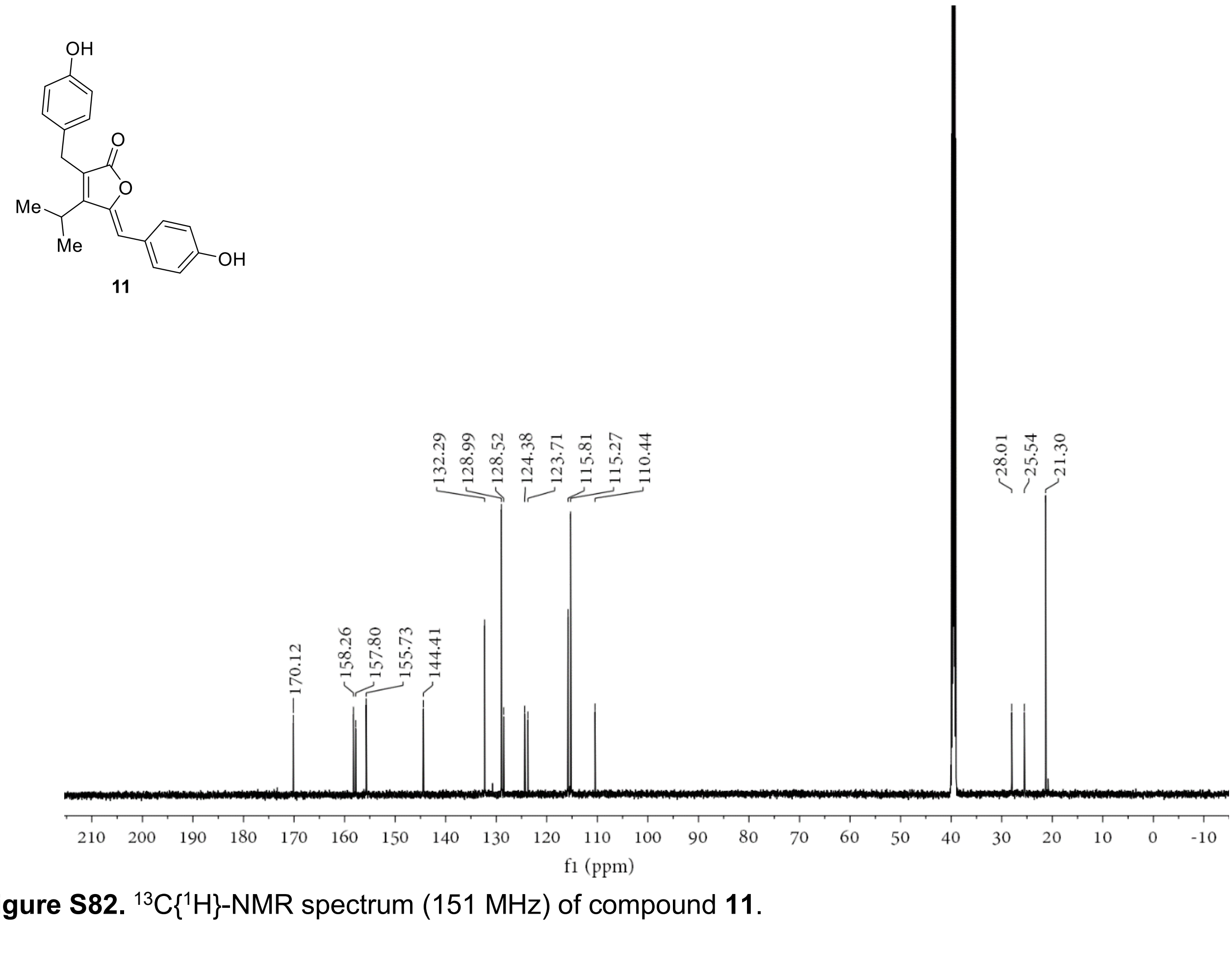

Figure S82. $^{13}C\{^{1}H\}$-NMR spectrum (151 MHz) of compound **11**.

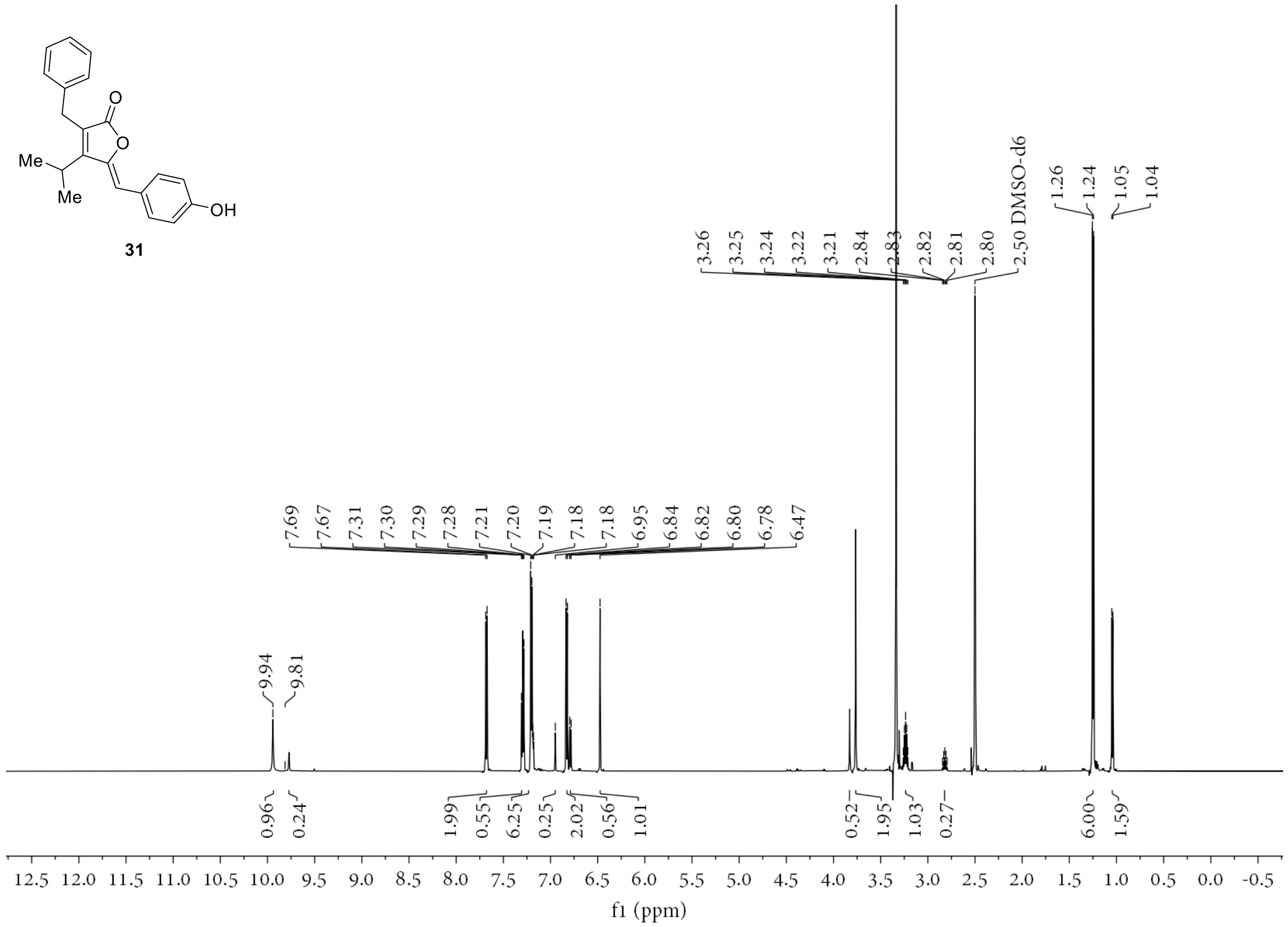

Figure S83. ^{1}H-NMR spectrum (600 MHz) of compound **31**.

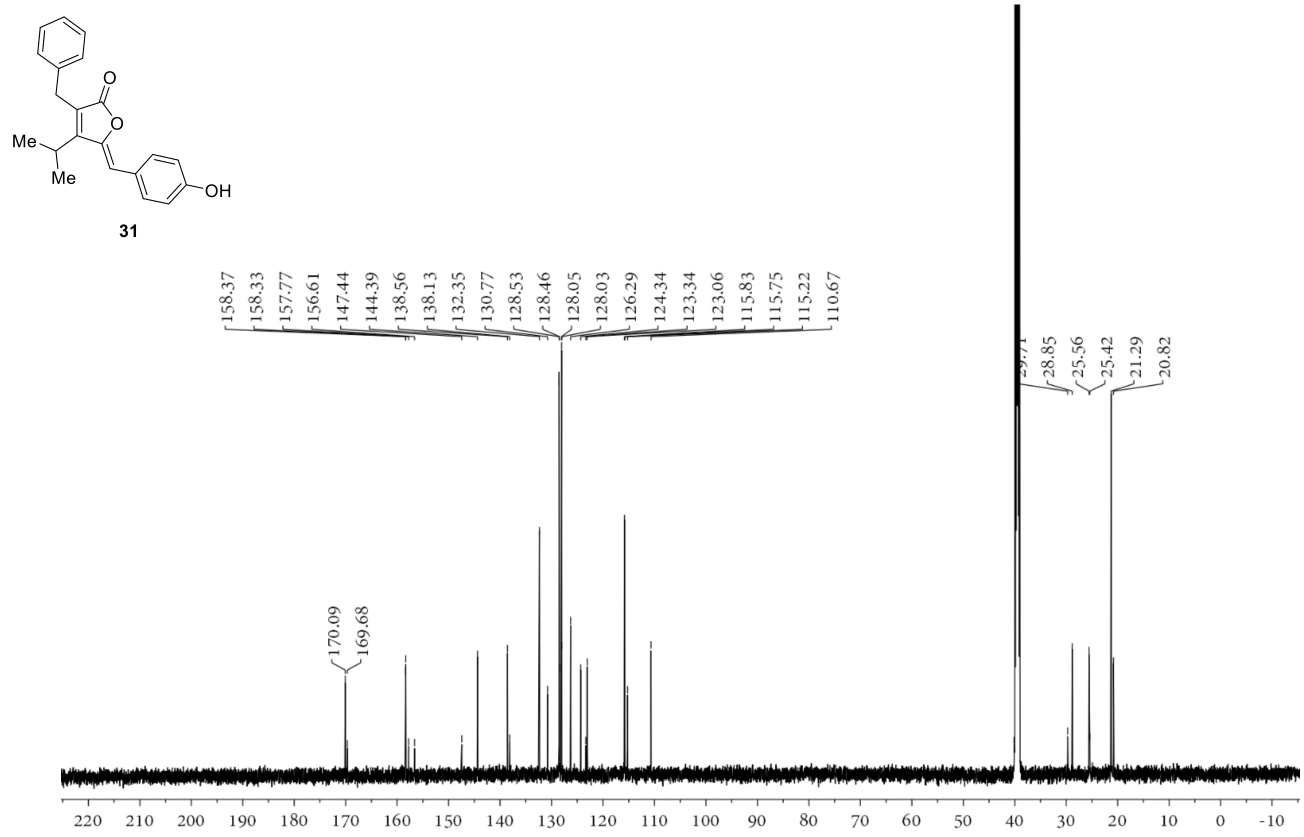

Figure S84. ^{13}C{^{1}H}-NMR spectrum (151 MHz) of compound **31**.

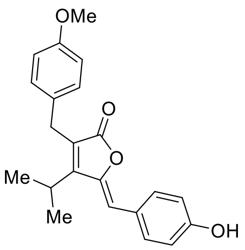

32

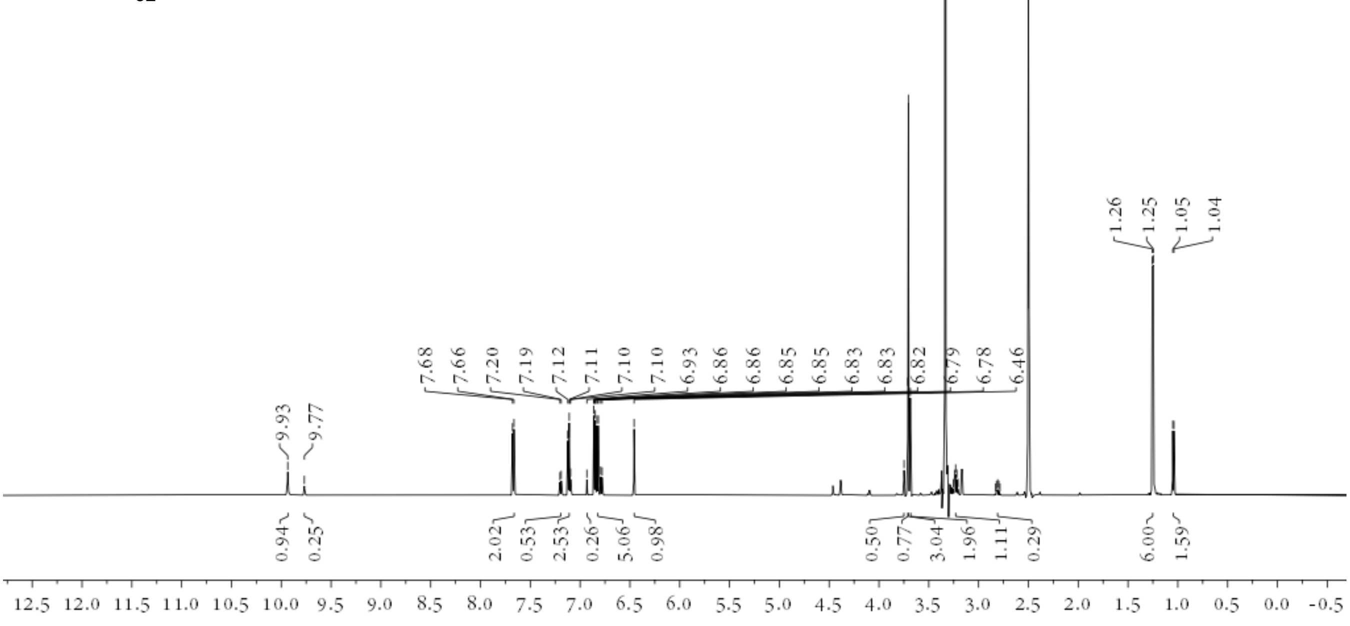

Figure S85. ^{1}H-NMR spectrum (600 MHz) of compound **32**.

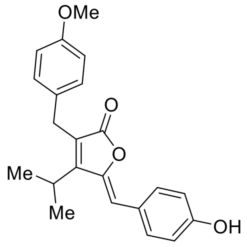

32

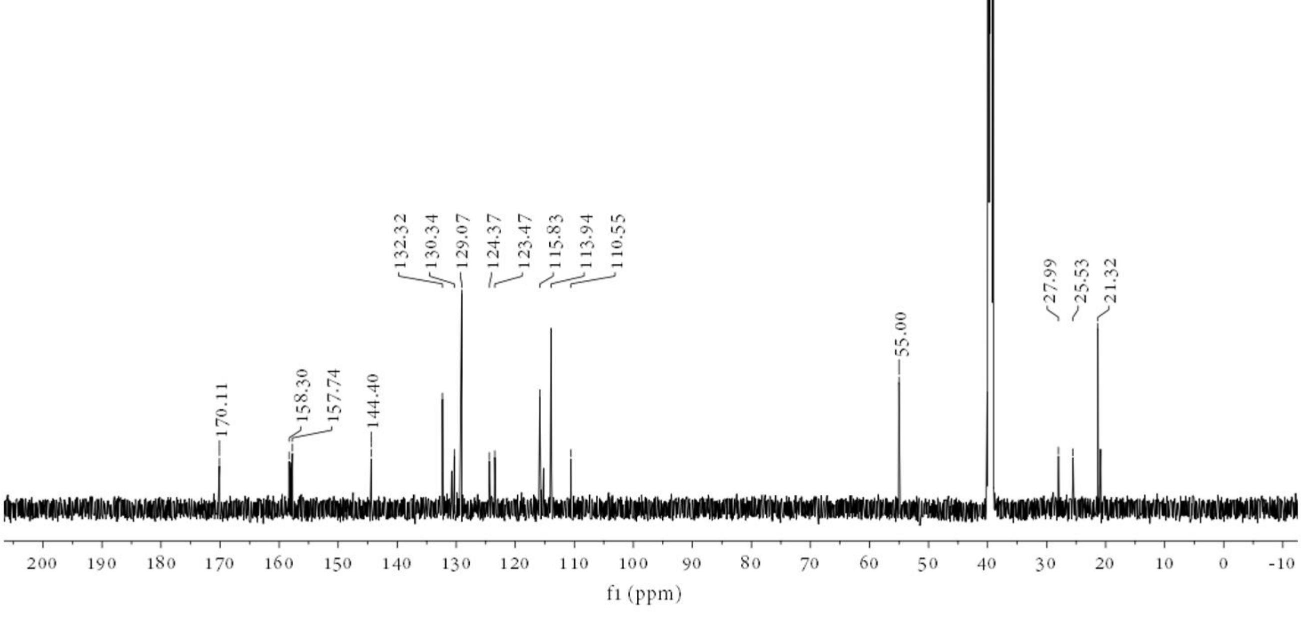

Figure S86. $^{13}C\{^{1}H\}$-NMR spectrum (151 MHz) of compound **32**.

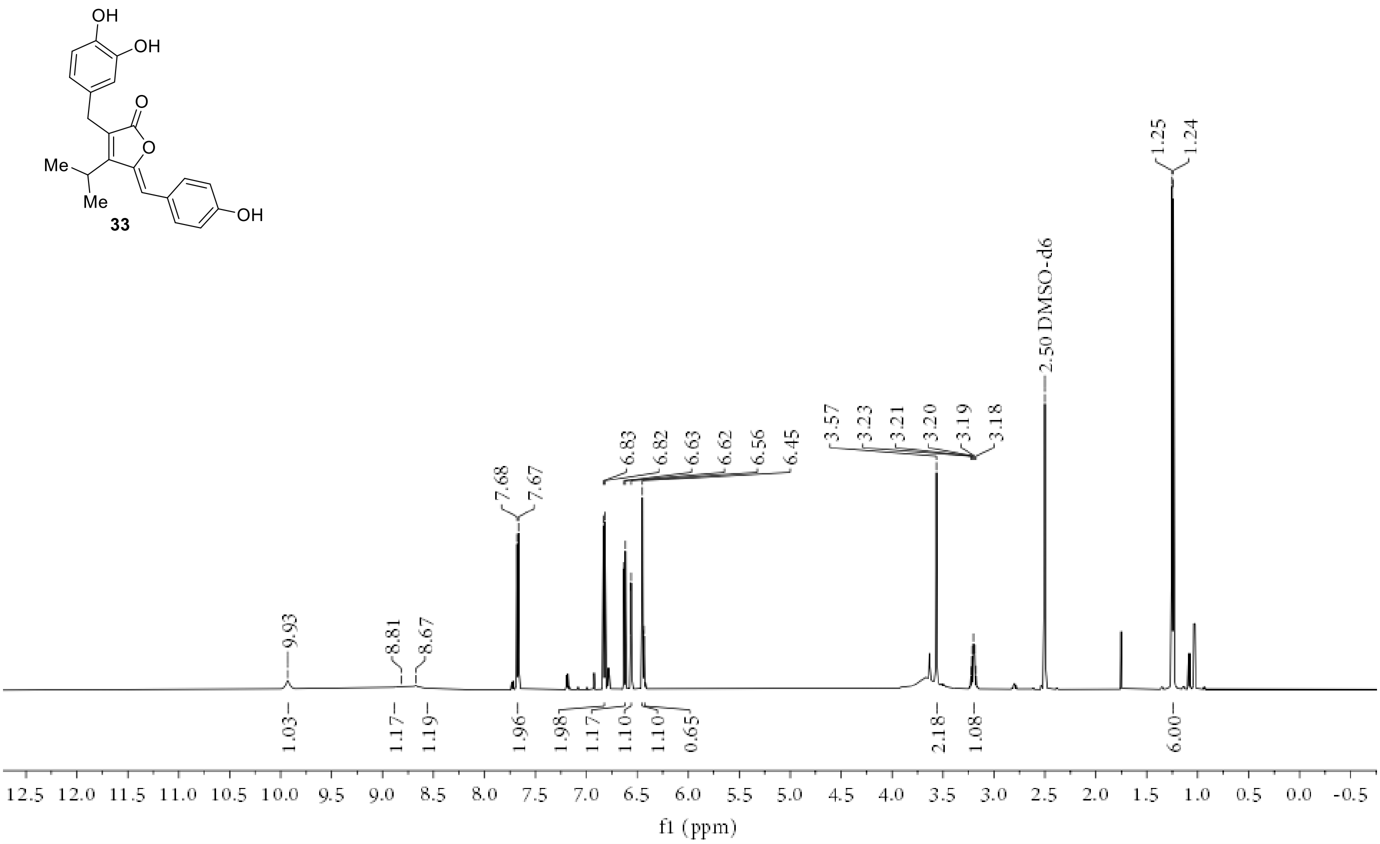

Figure S87. ^{1}H-NMR spectrum (600 MHz) of compound **33**.

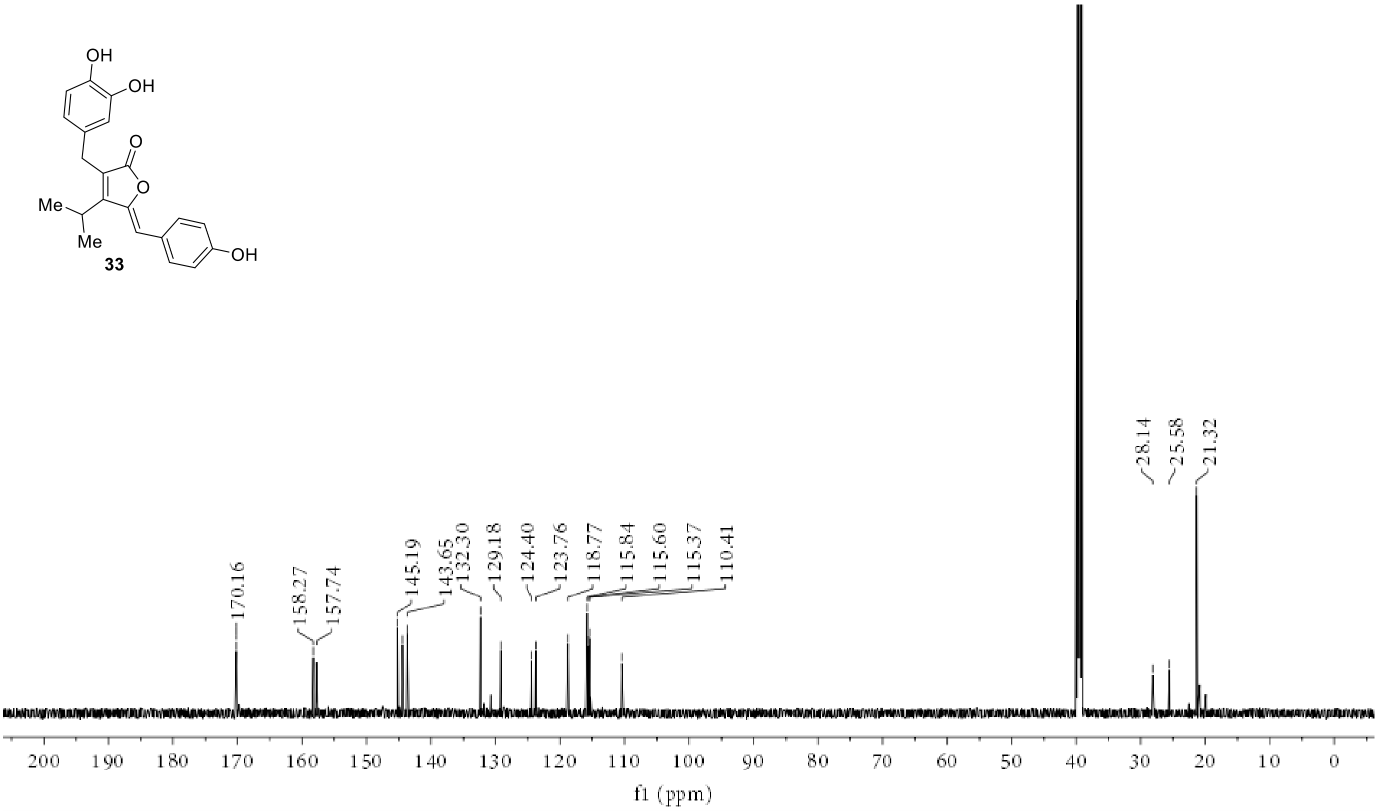

Figure S88. $^{13}C\{^{1}H\}$-NMR spectrum (151 MHz) of compound **33**.

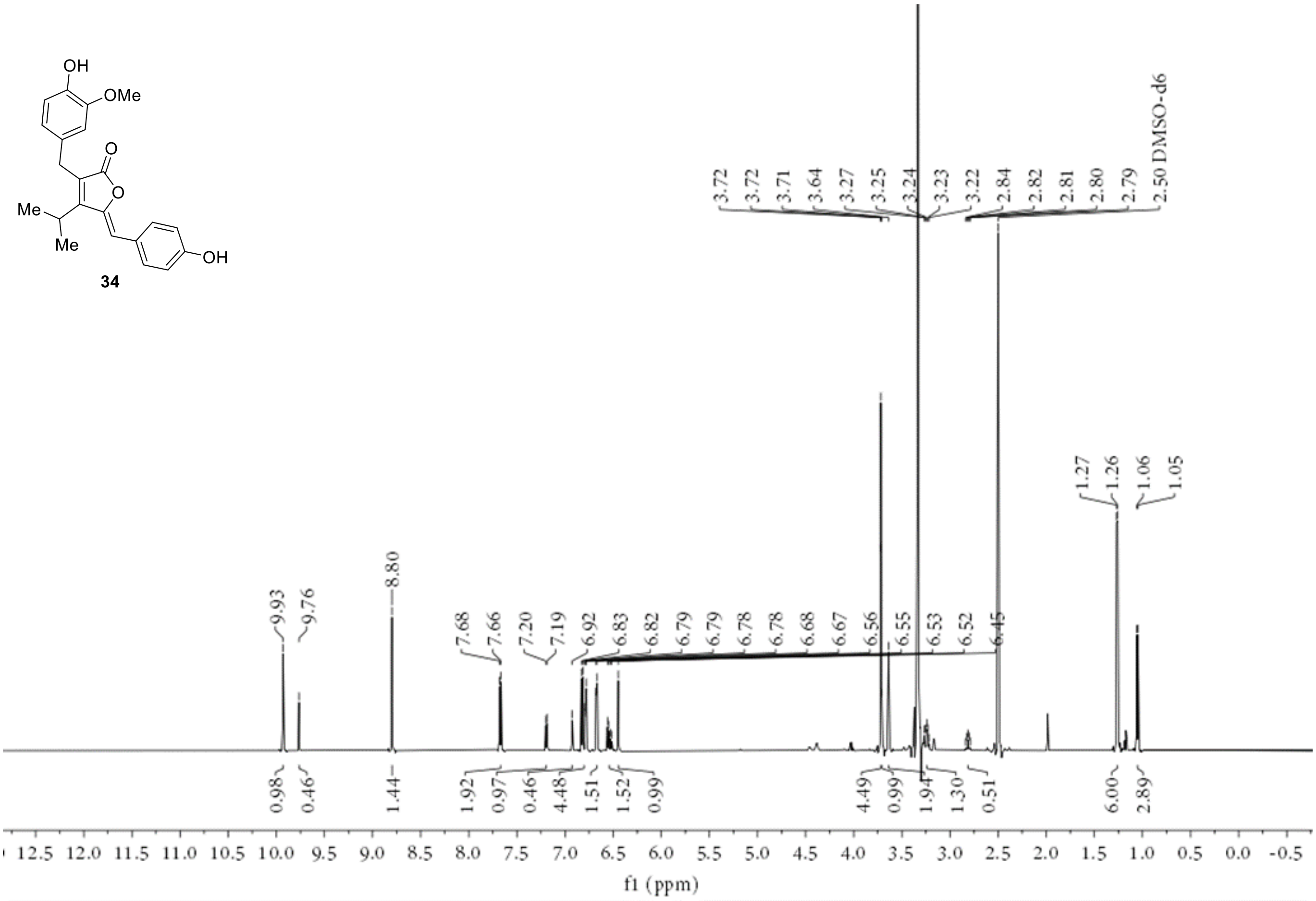

Figure S89. ^{1}H-NMR spectrum (600 MHz) of compound **34**.

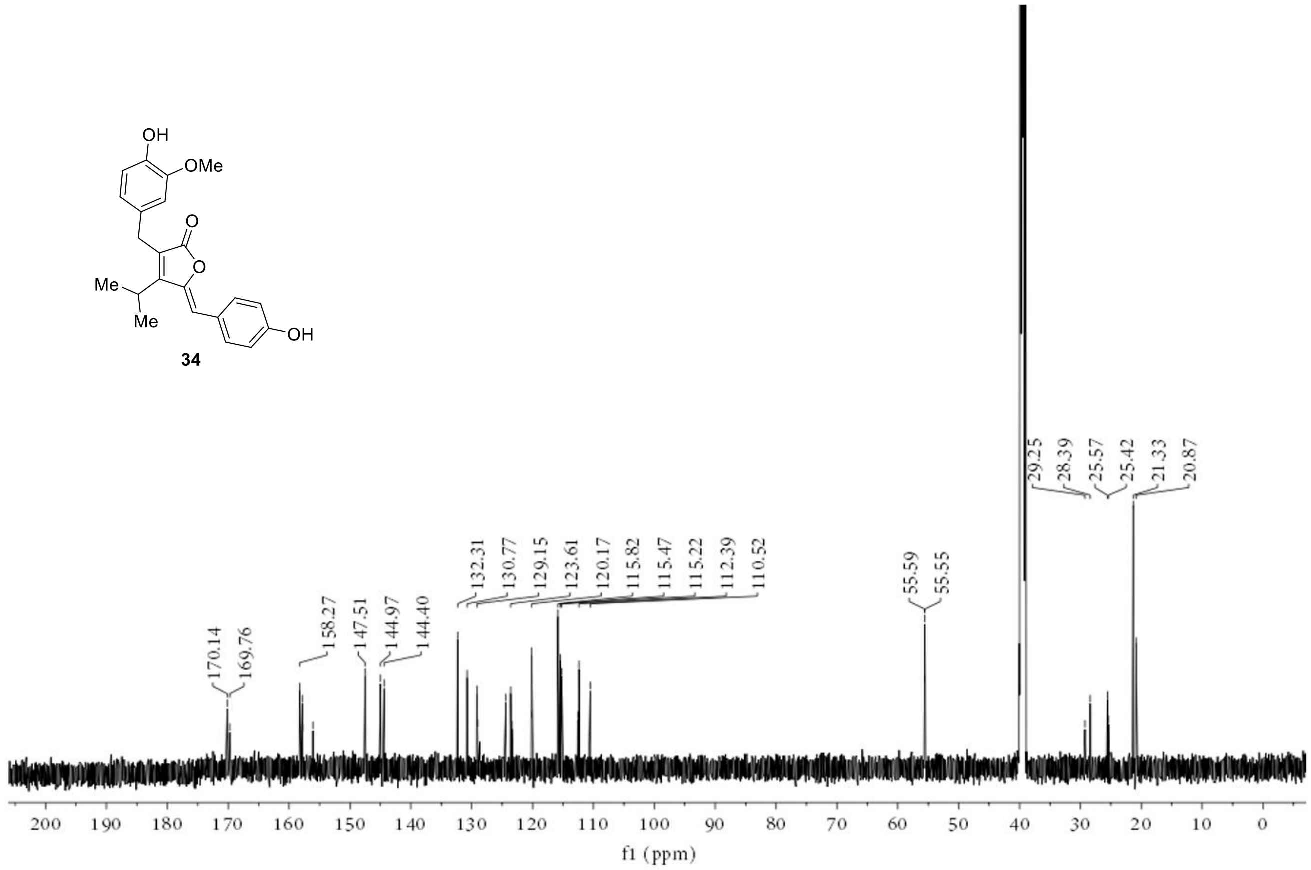

Figure S90. ^{13}C{^{1}H}-NMR spectrum (151 MHz) of compound **34**.

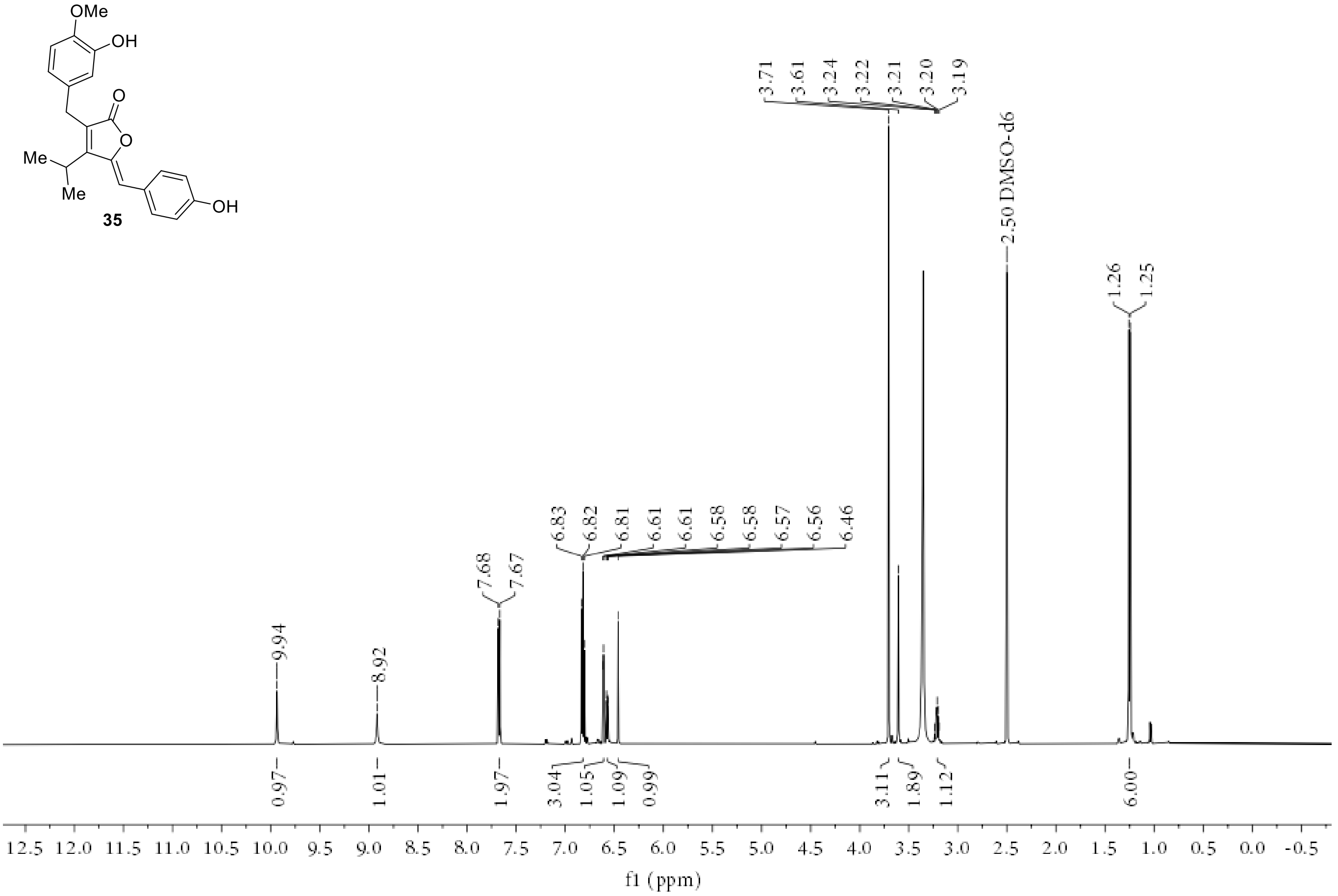

Figure S91. ^{1}H-NMR spectrum (600 MHz) of compound **35**.

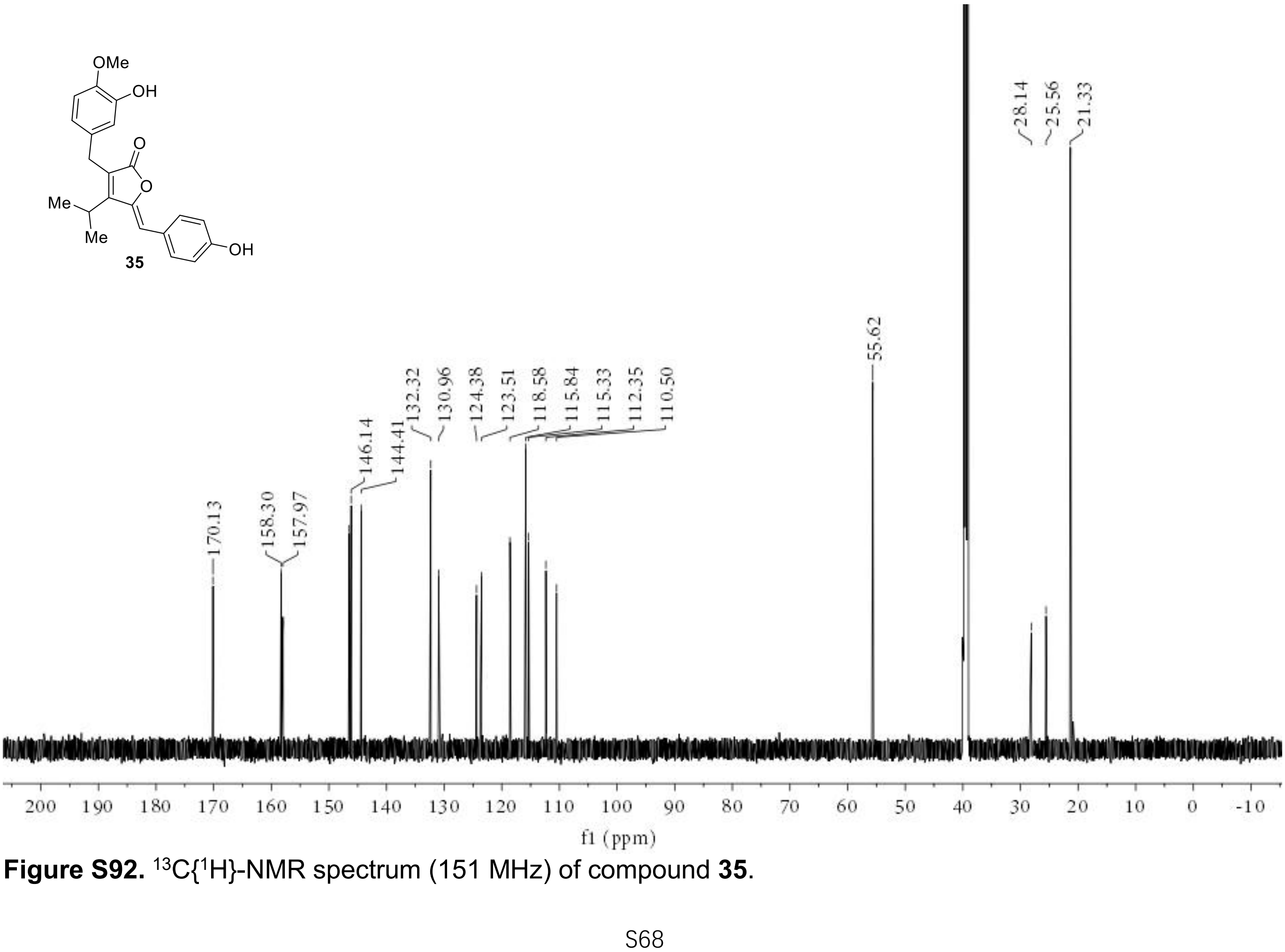

Figure S92. ^{13}C{^{1}H}-NMR spectrum (151 MHz) of compound **35**.

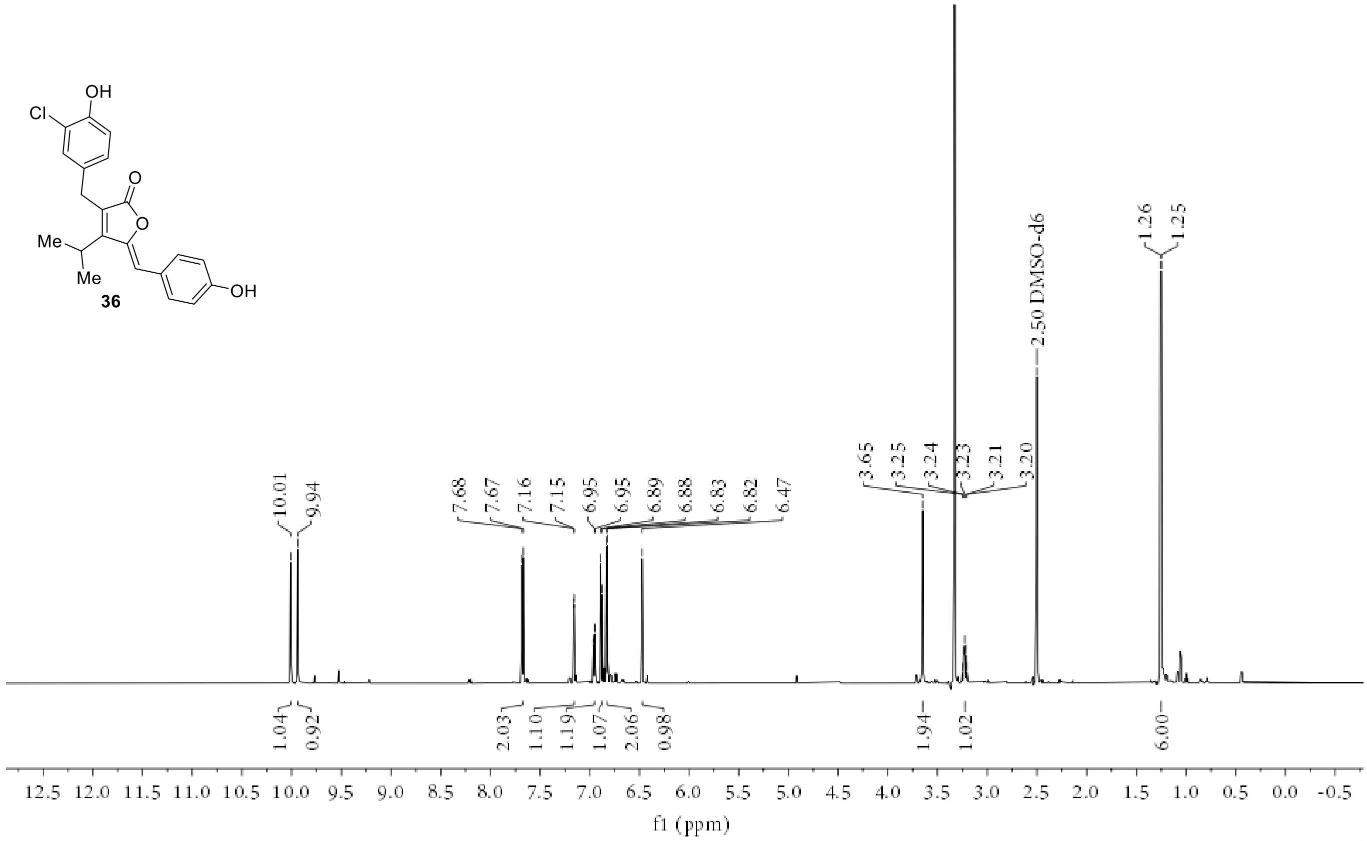

Figure S93. ^{1}H-NMR spectrum (600 MHz) of compound **36**.

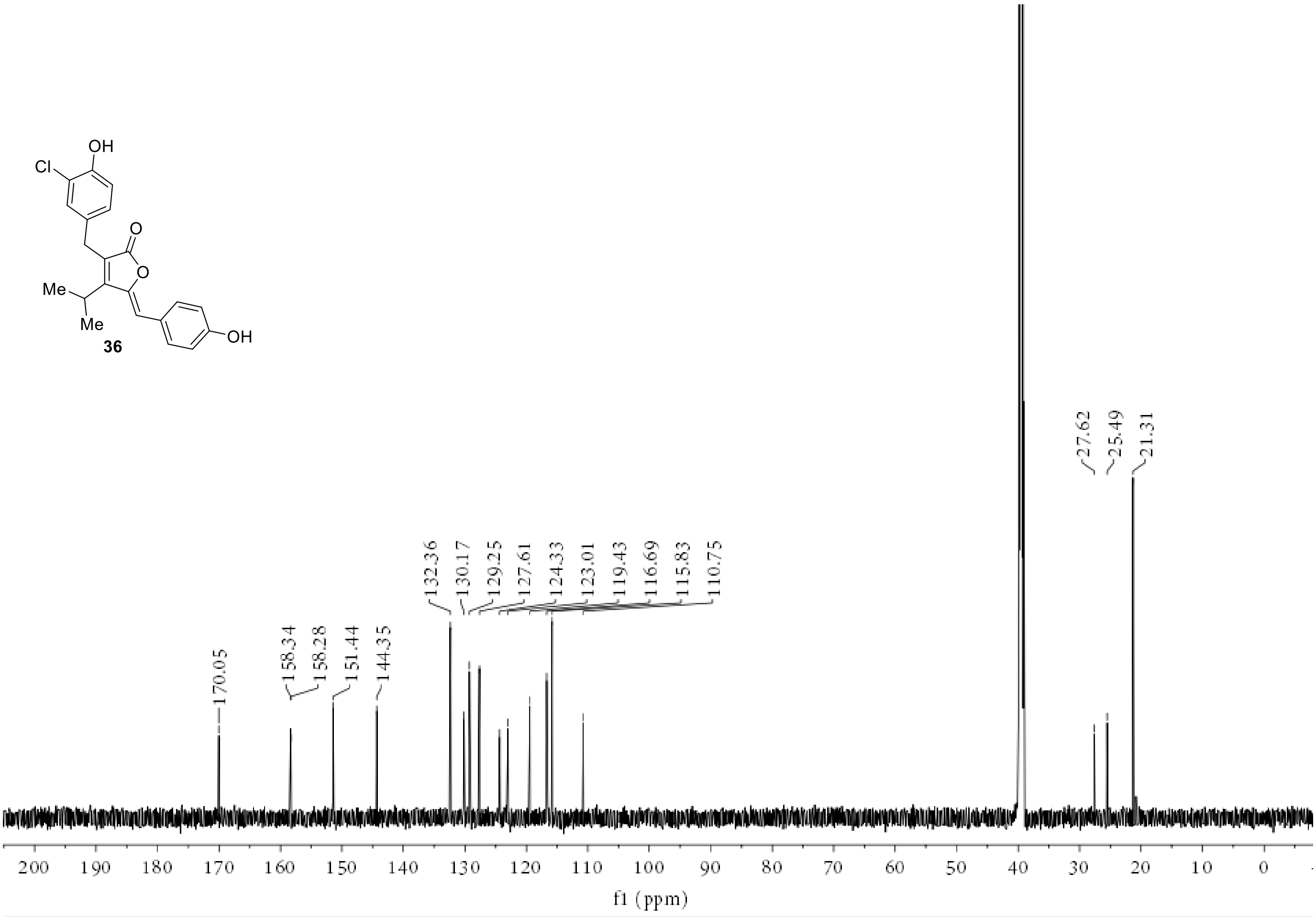

Figure S94. ^{13}C{^{1}H}-NMR spectrum (151 MHz) of compound **36**.

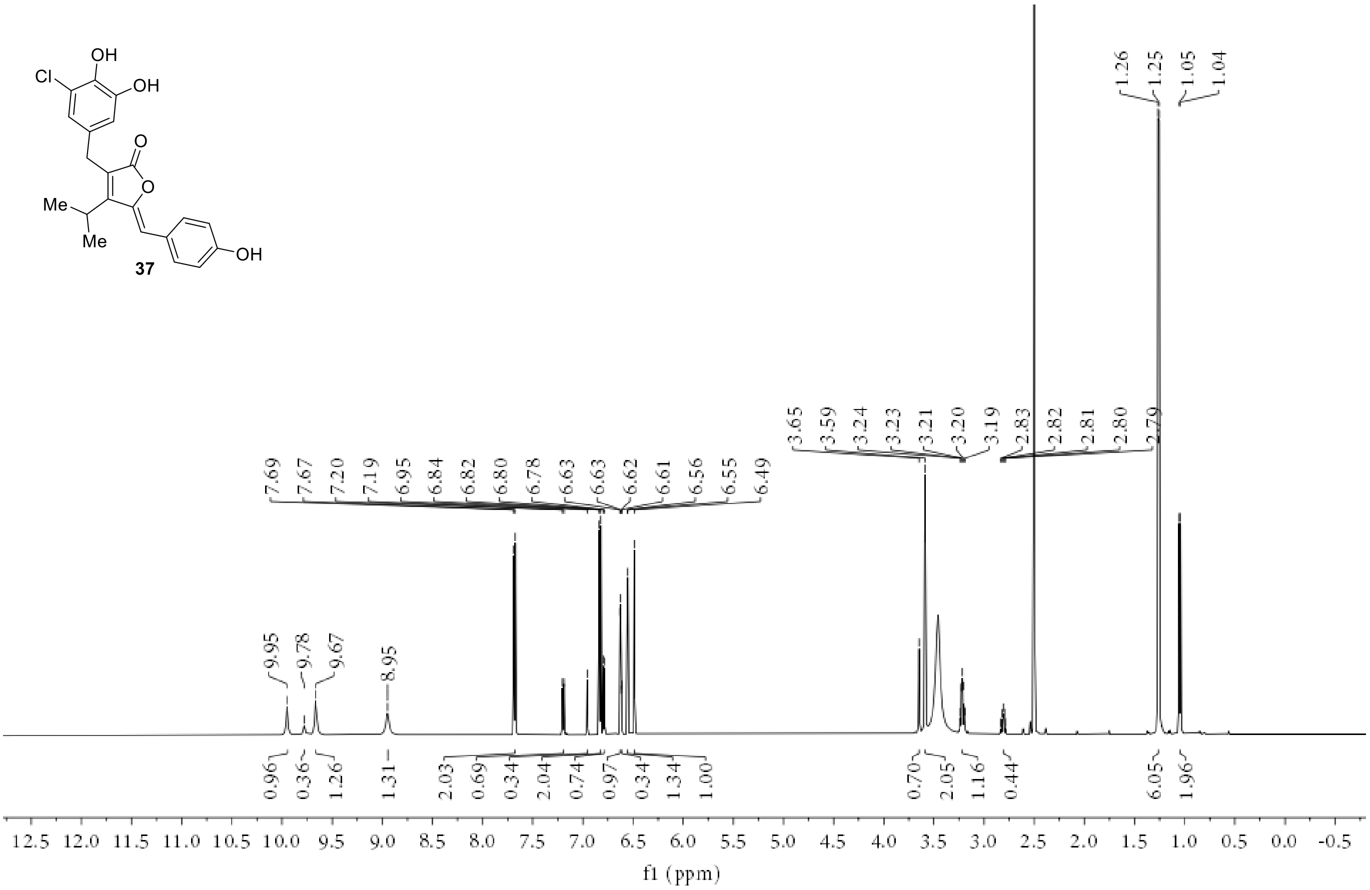

Figure S95. ^{1}H-NMR spectrum (600 MHz) of compound **37**.

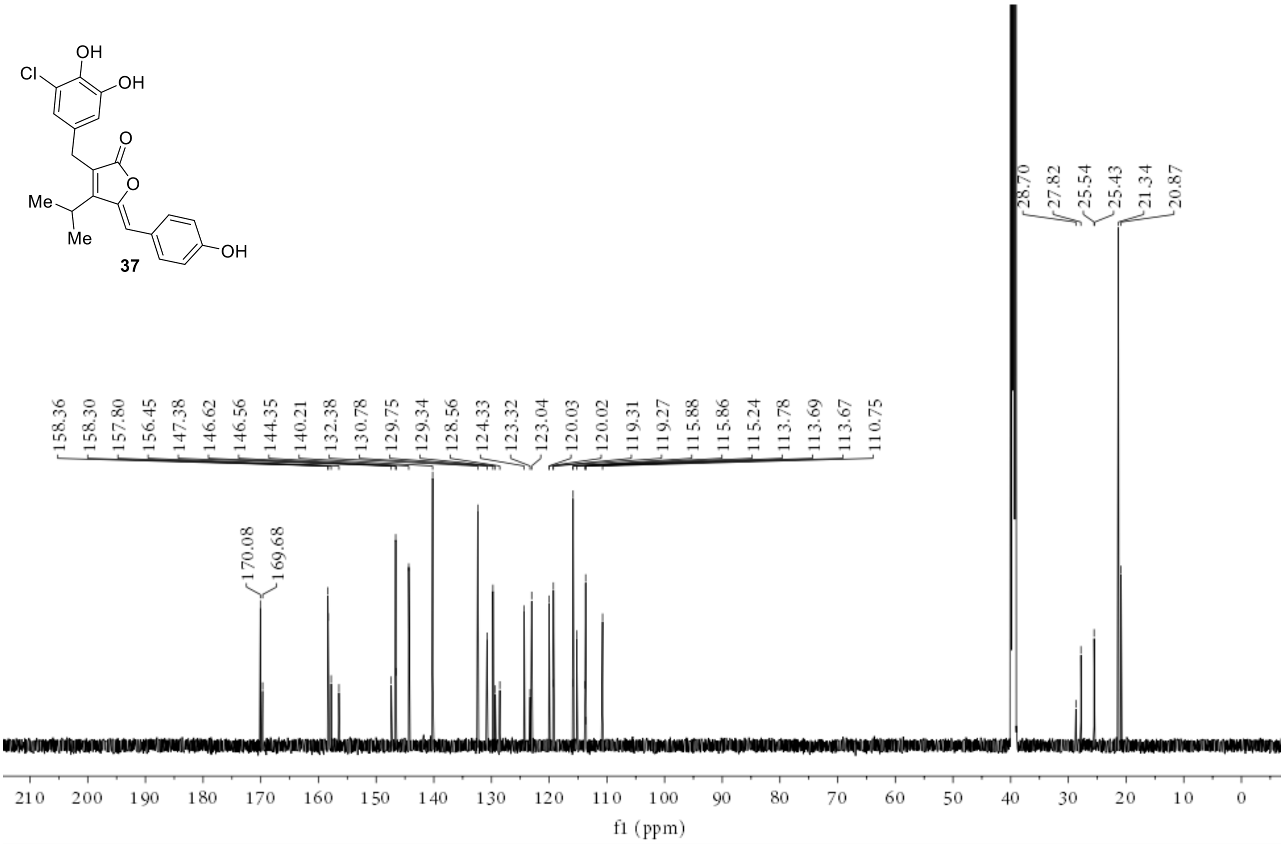

Figure S96. $^{13}C\{^1H\}$-NMR spectrum (151 MHz) of compound **37**.

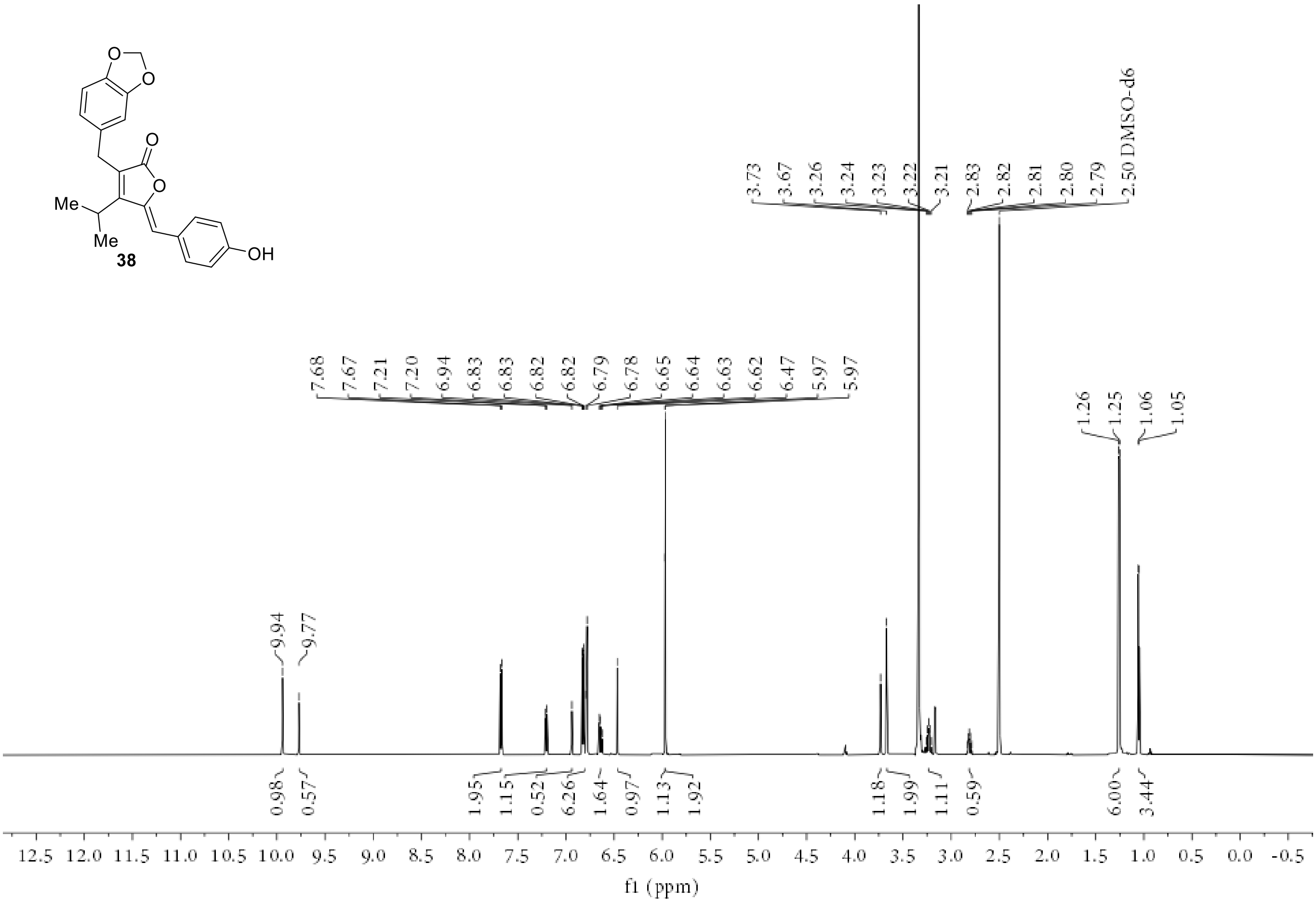

Figure S97. ^{1}H-NMR spectrum (600 MHz) of compound **38**.

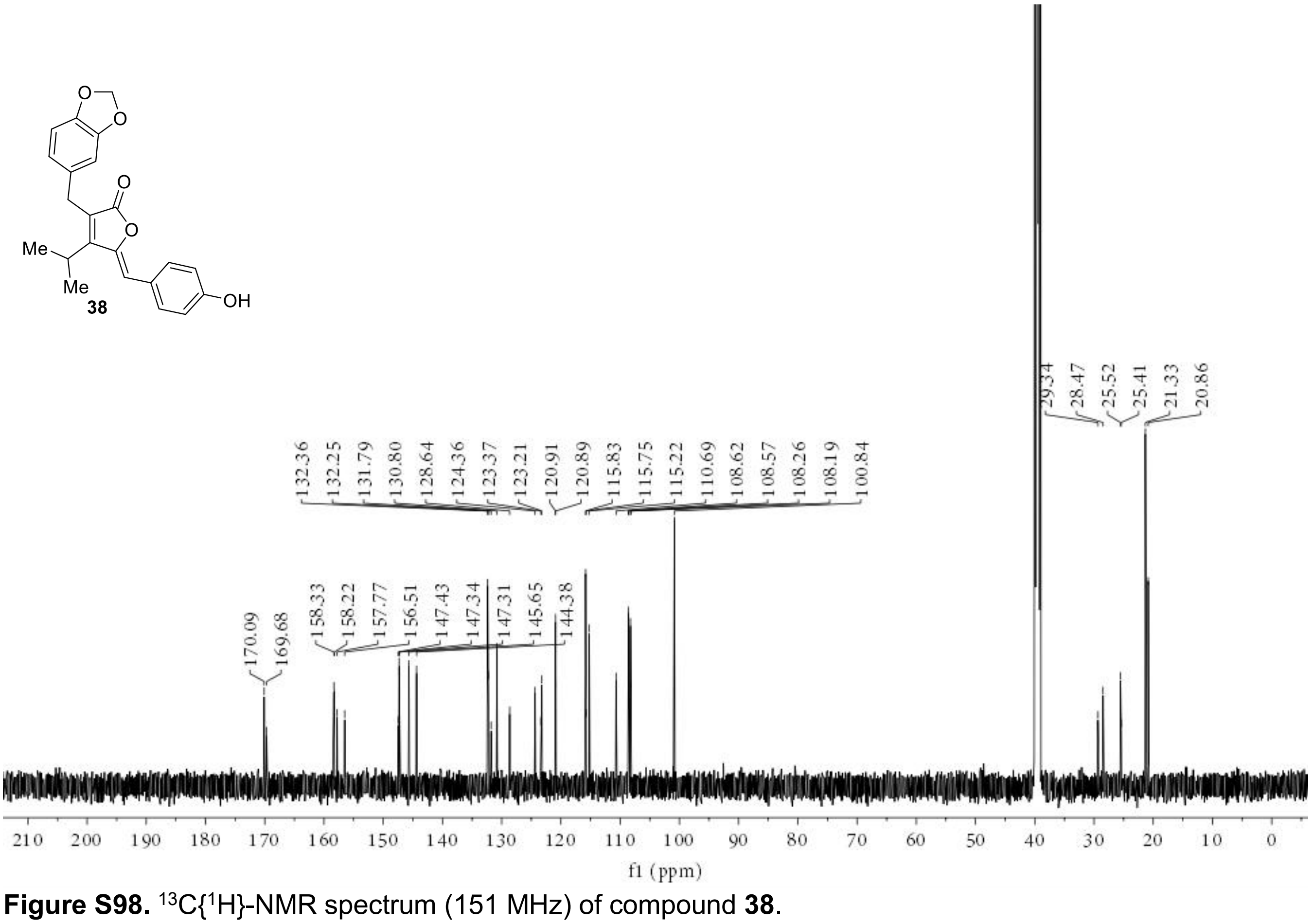

Figure S98. $^{13}C\{^{1}H\}$-NMR spectrum (151 MHz) of compound **38**.

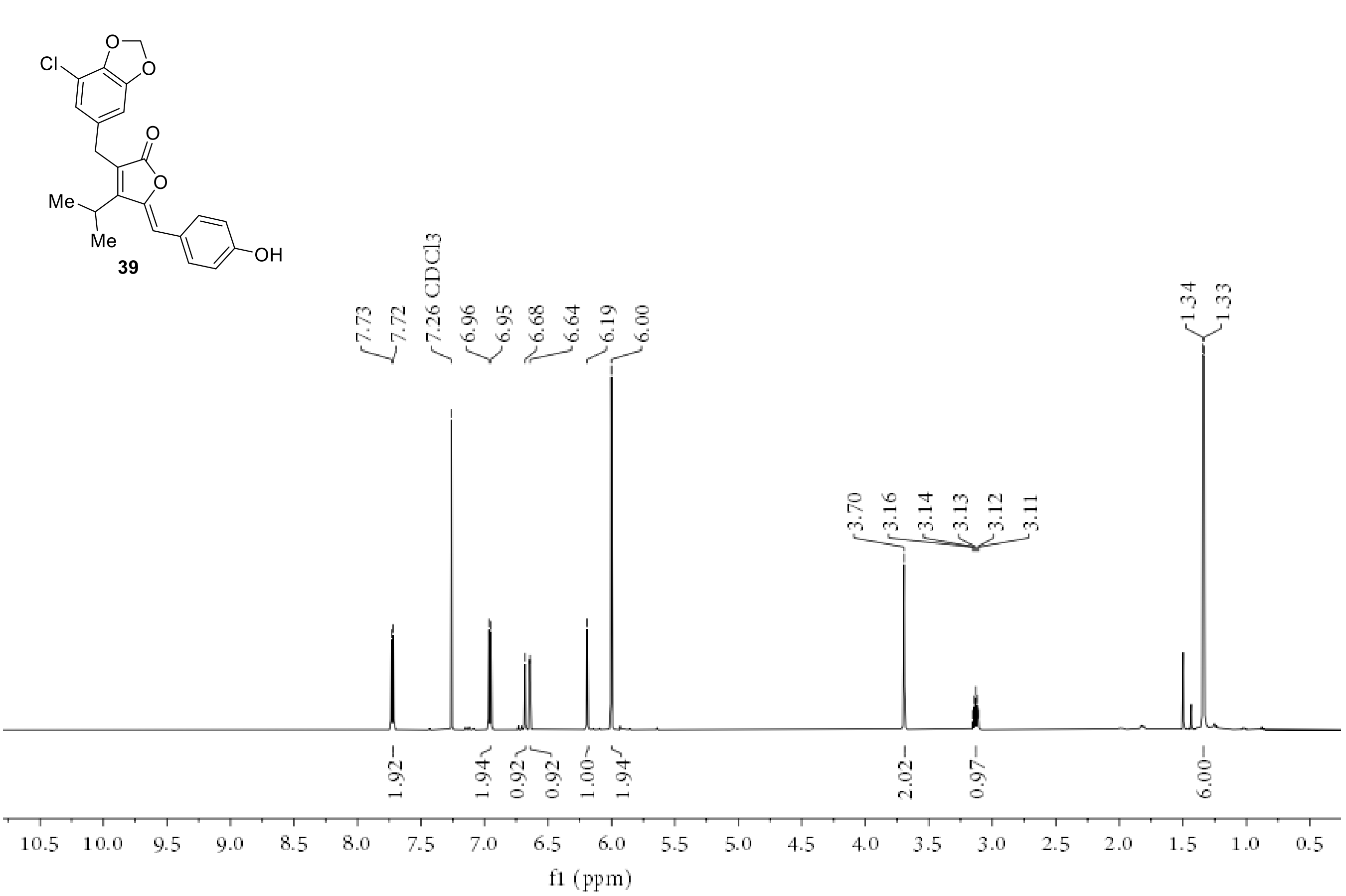

Figure S99. ^{1}H-NMR spectrum (600 MHz) of compound **39**.

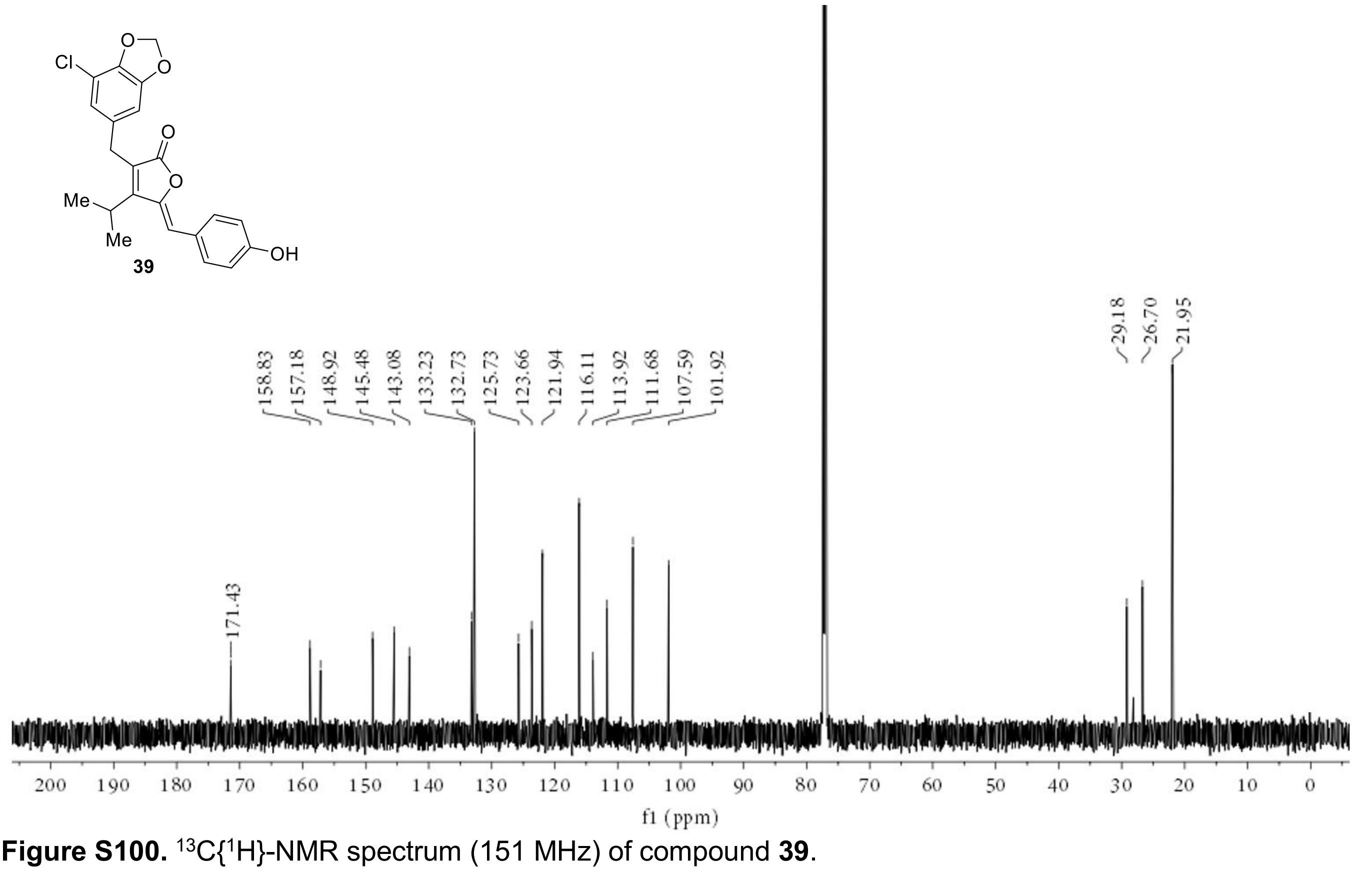

Figure S100. ^{13}C{^{1}H}-NMR spectrum (151 MHz) of compound **39**.

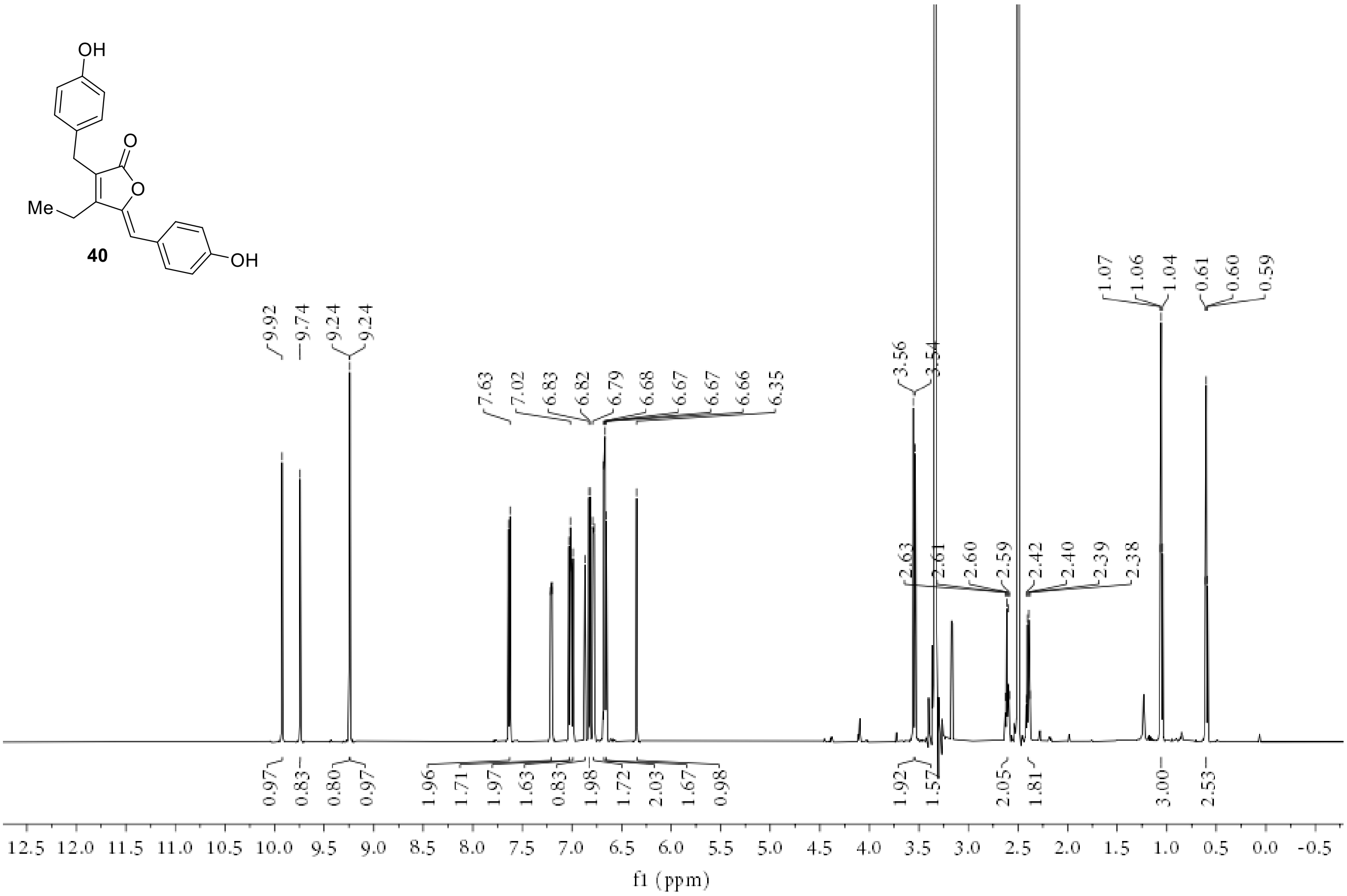

Figure S101. ^{1}H-NMR spectrum (600 MHz) of compound **40**.

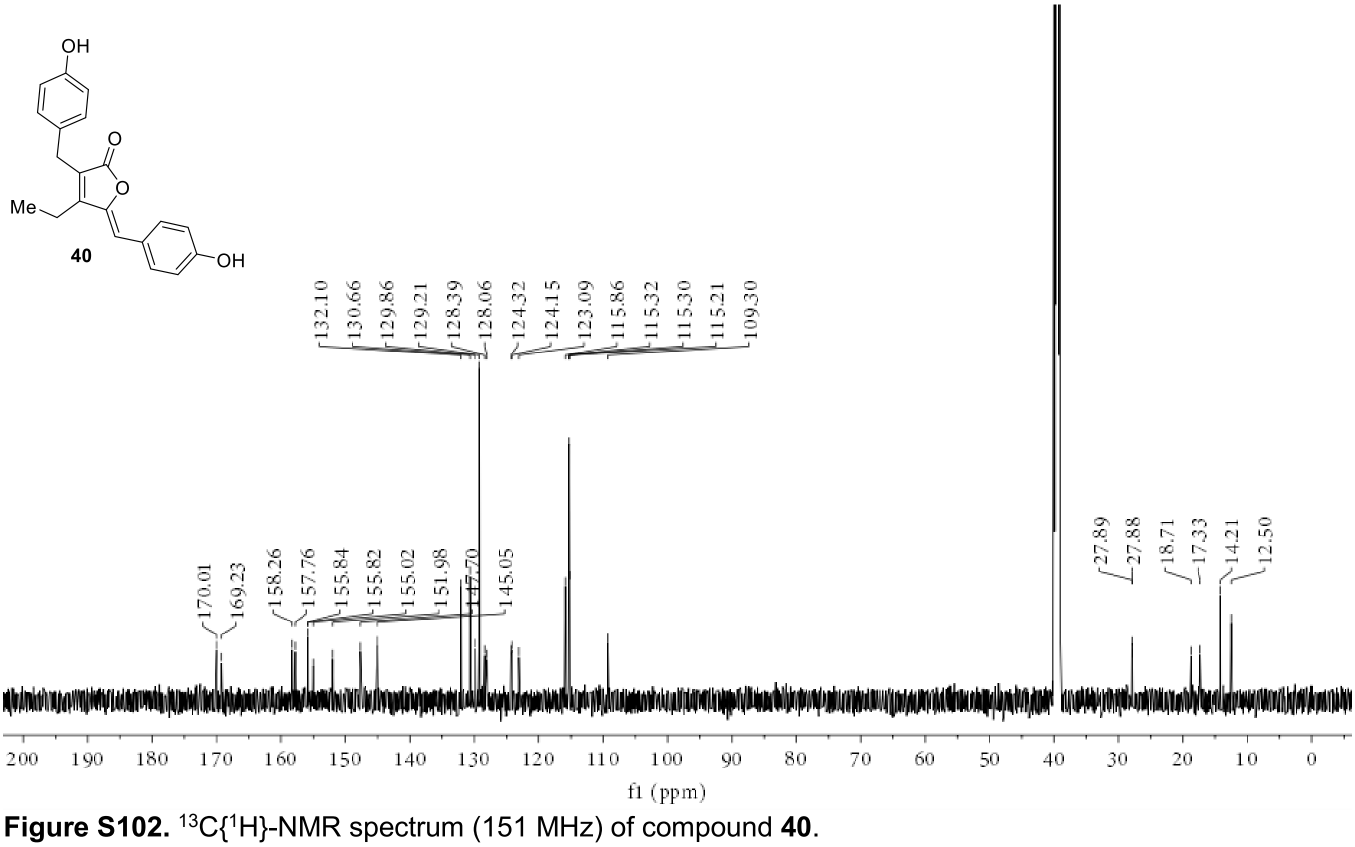

Figure S102. ^{13}C{^{1}H}-NMR spectrum (151 MHz) of compound **40**.

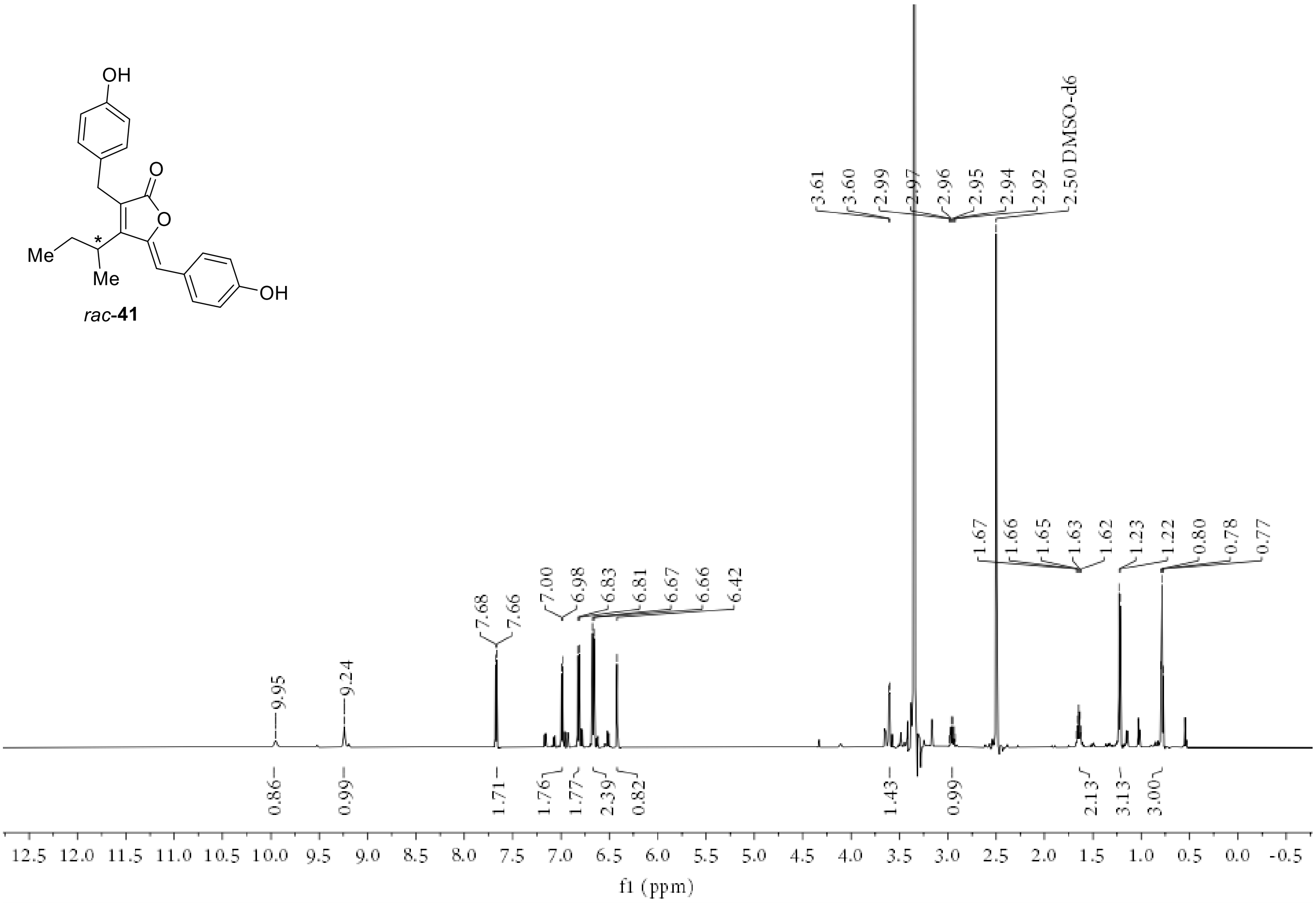

Figure S103. ^{1}H-NMR spectrum (600 MHz) of compound *rac*-**41**.

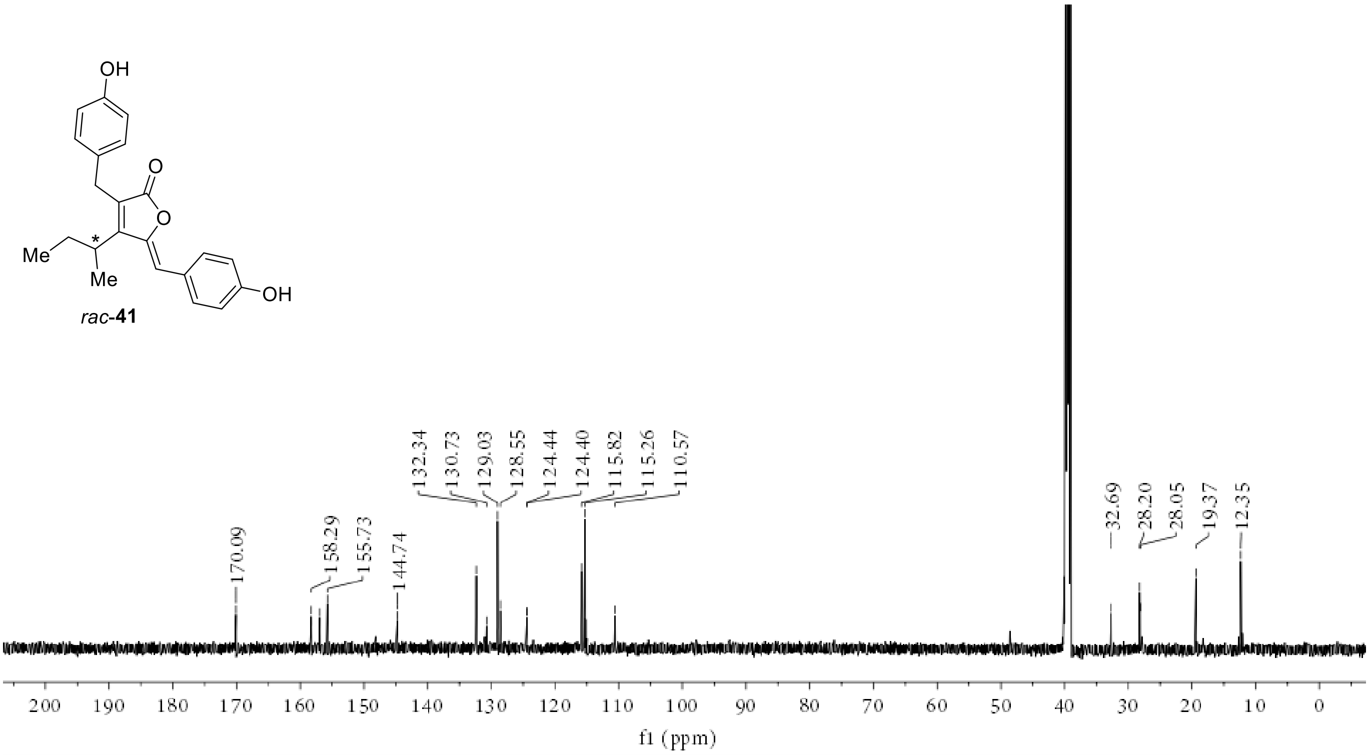

Figure S104. ^{13}C{^{1}H}-NMR spectrum (151 MHz) of compound *rac*-**41**.

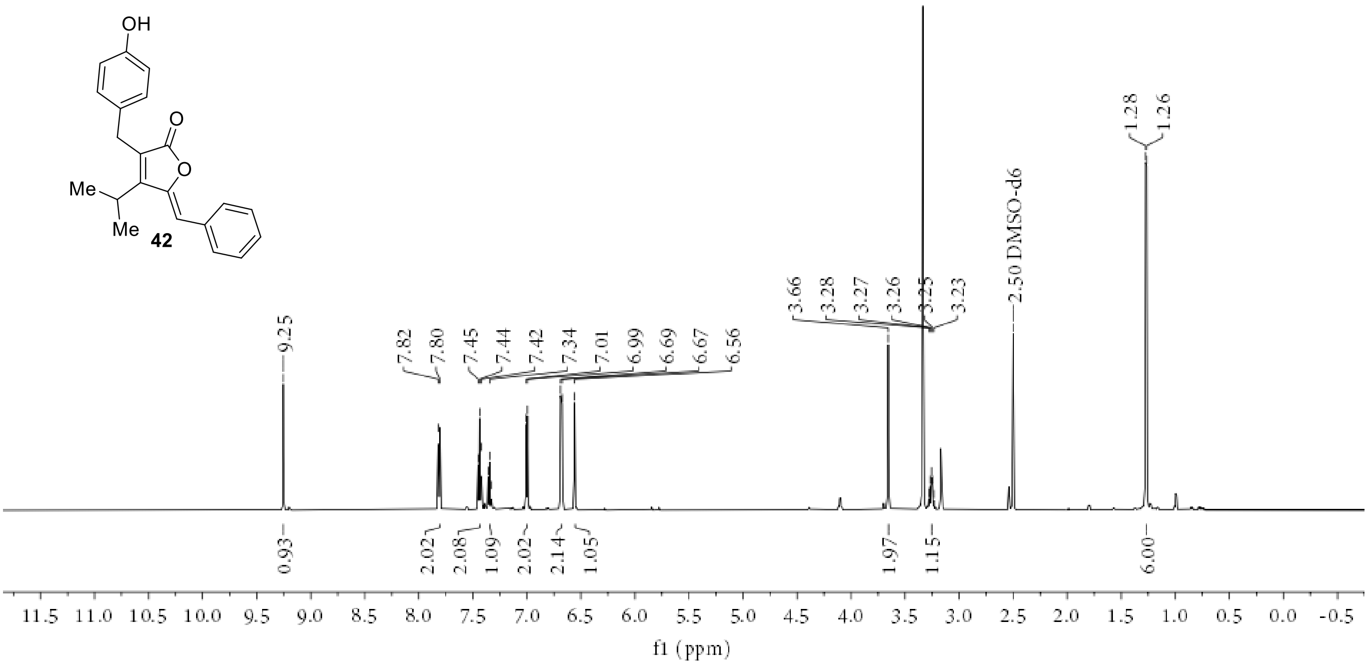

Figure S105. ^{1}H-NMR spectrum (600 MHz) of compound **42**.

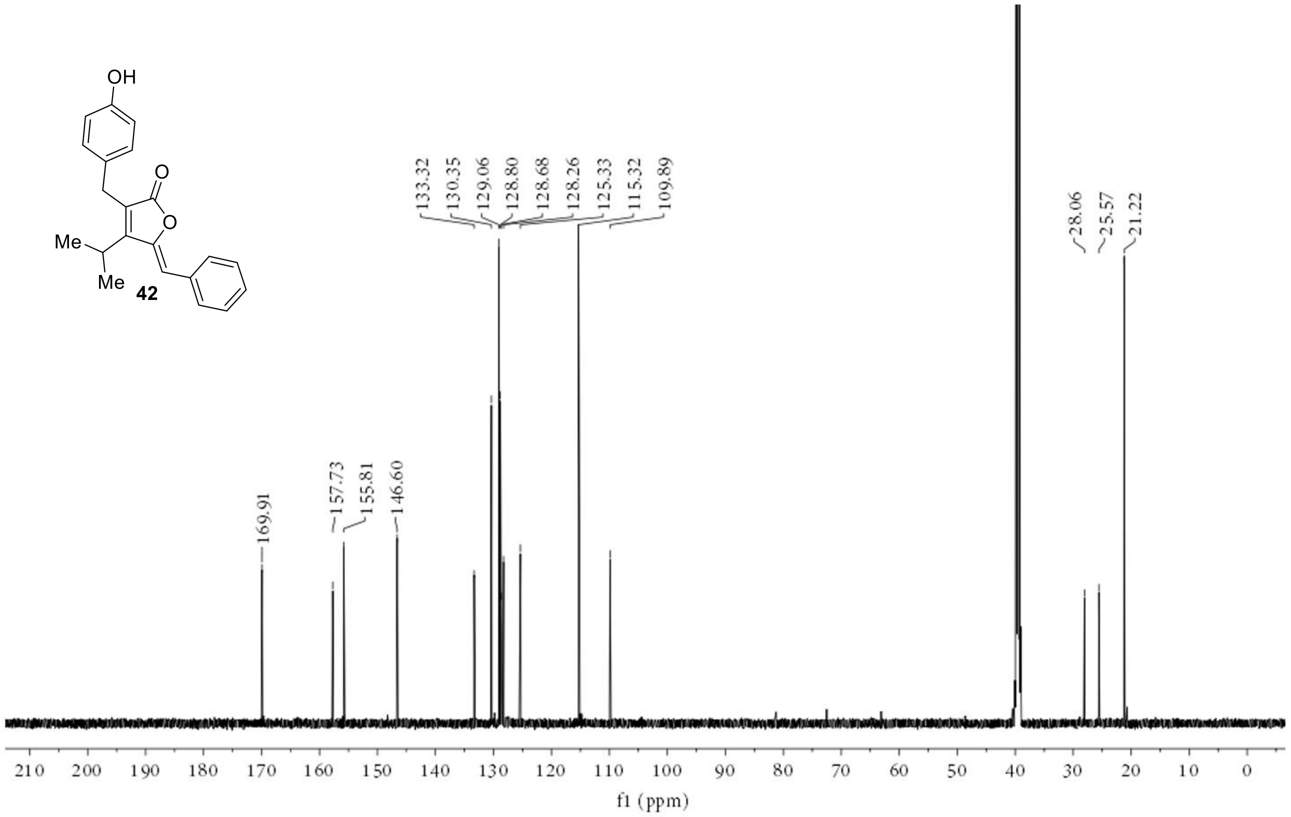

Figure S106. ^{13}C{^{1}H}-NMR spectrum (151 MHz) of compound **42**.

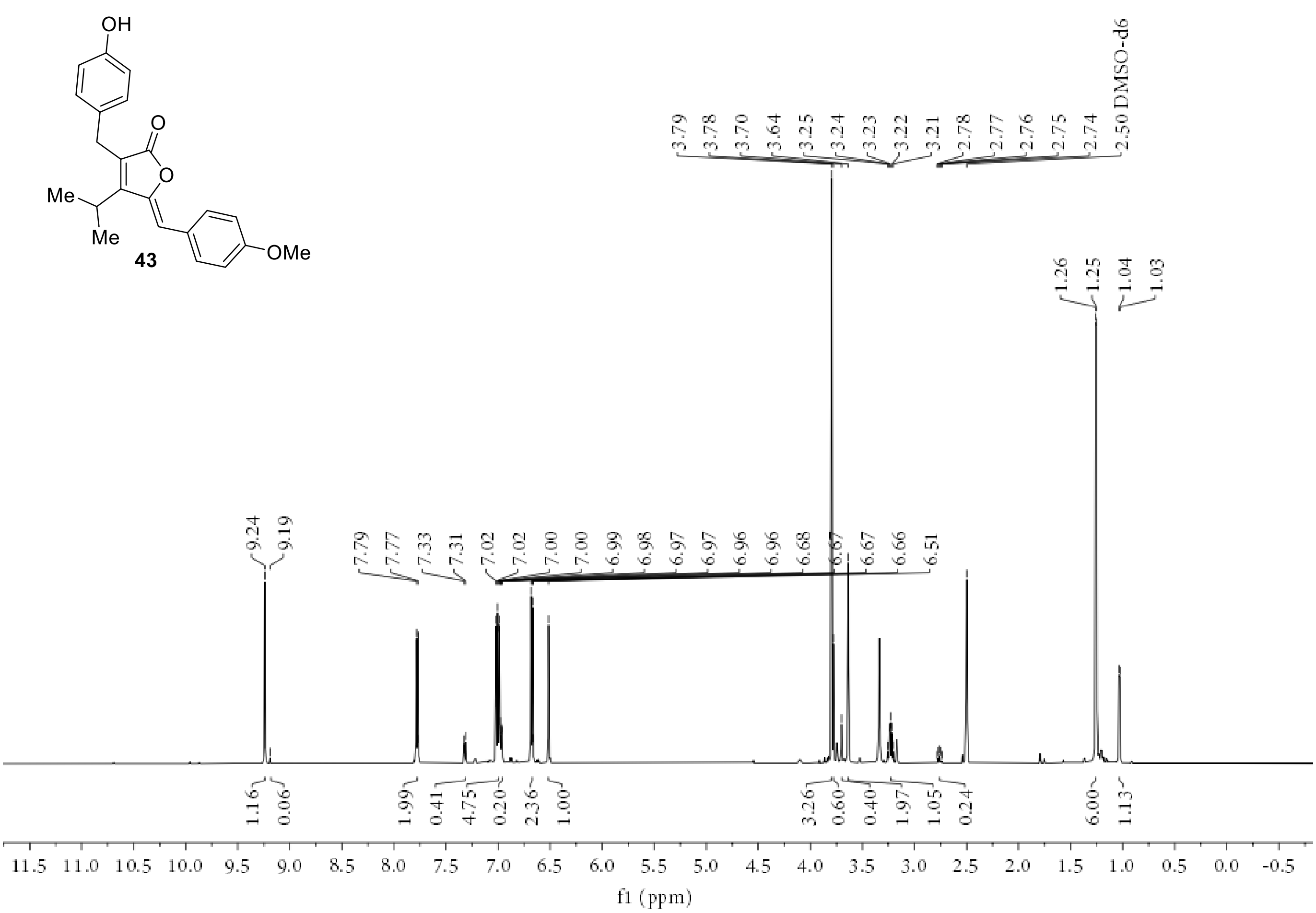

Figure S107. ^{1}H-NMR spectrum (600 MHz) of compound **43**.

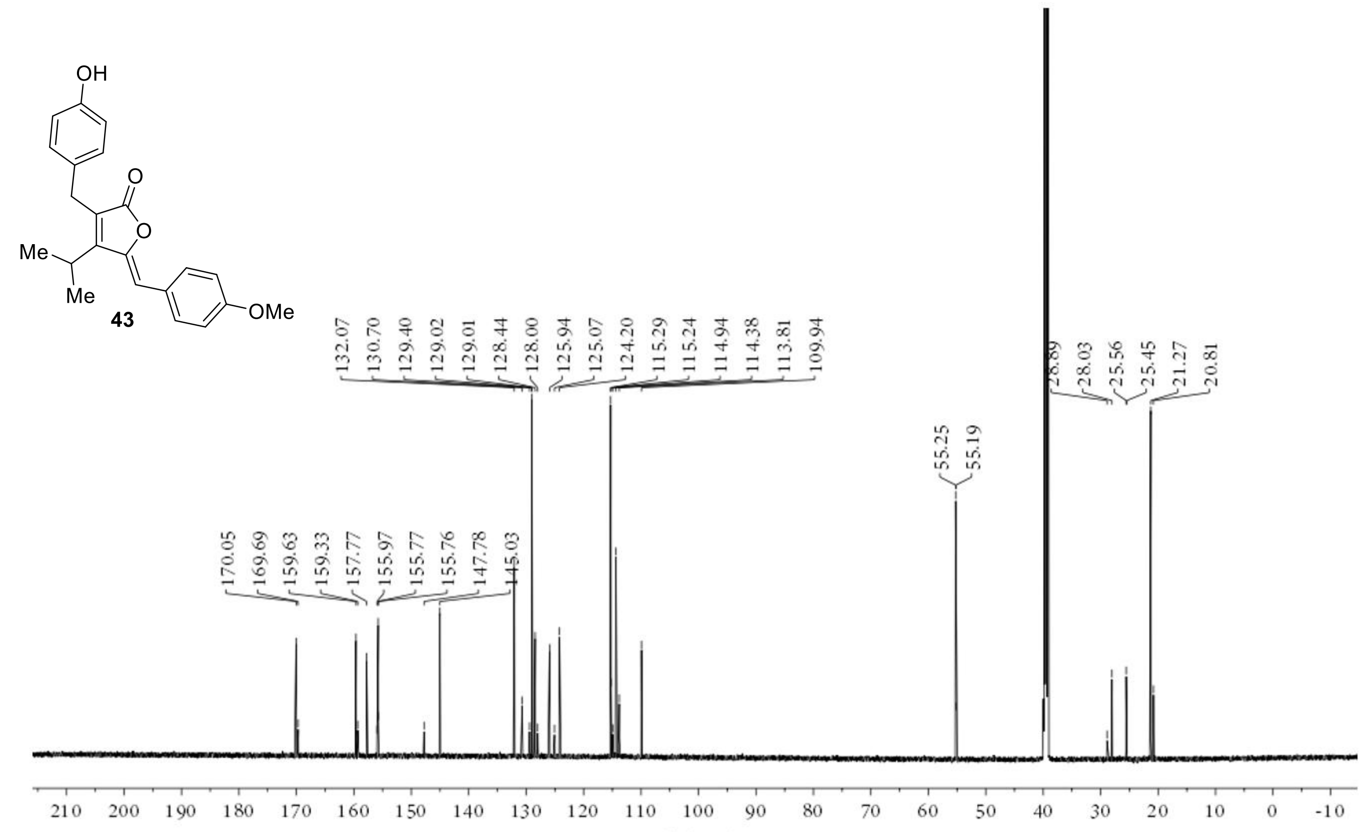

Figure S108. $^{13}C\{^1H\}$-NMR spectrum (151 MHz) of compound **43**.

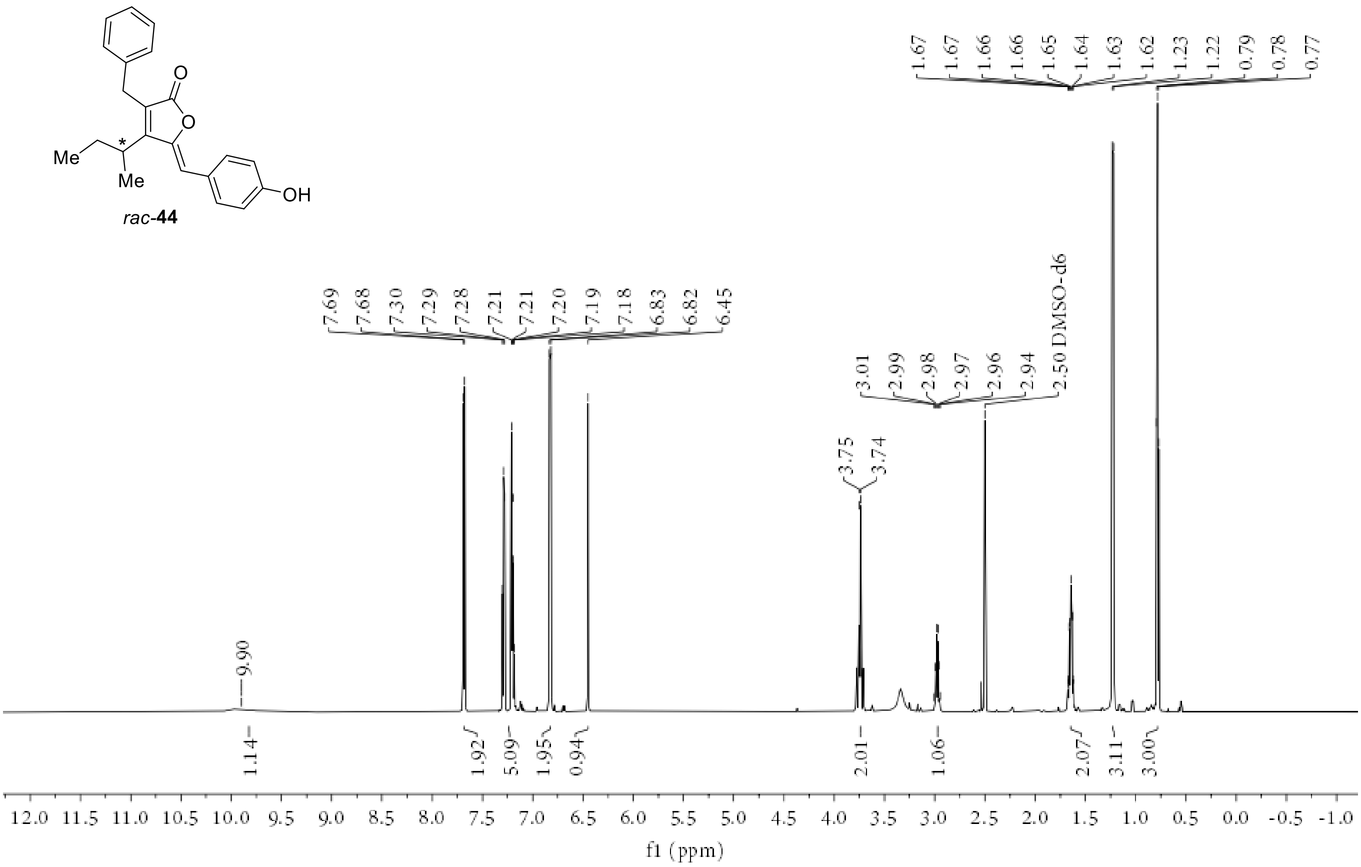

Figure S109. ^{1}H-NMR spectrum (600 MHz) of compound *rac*-**44**.

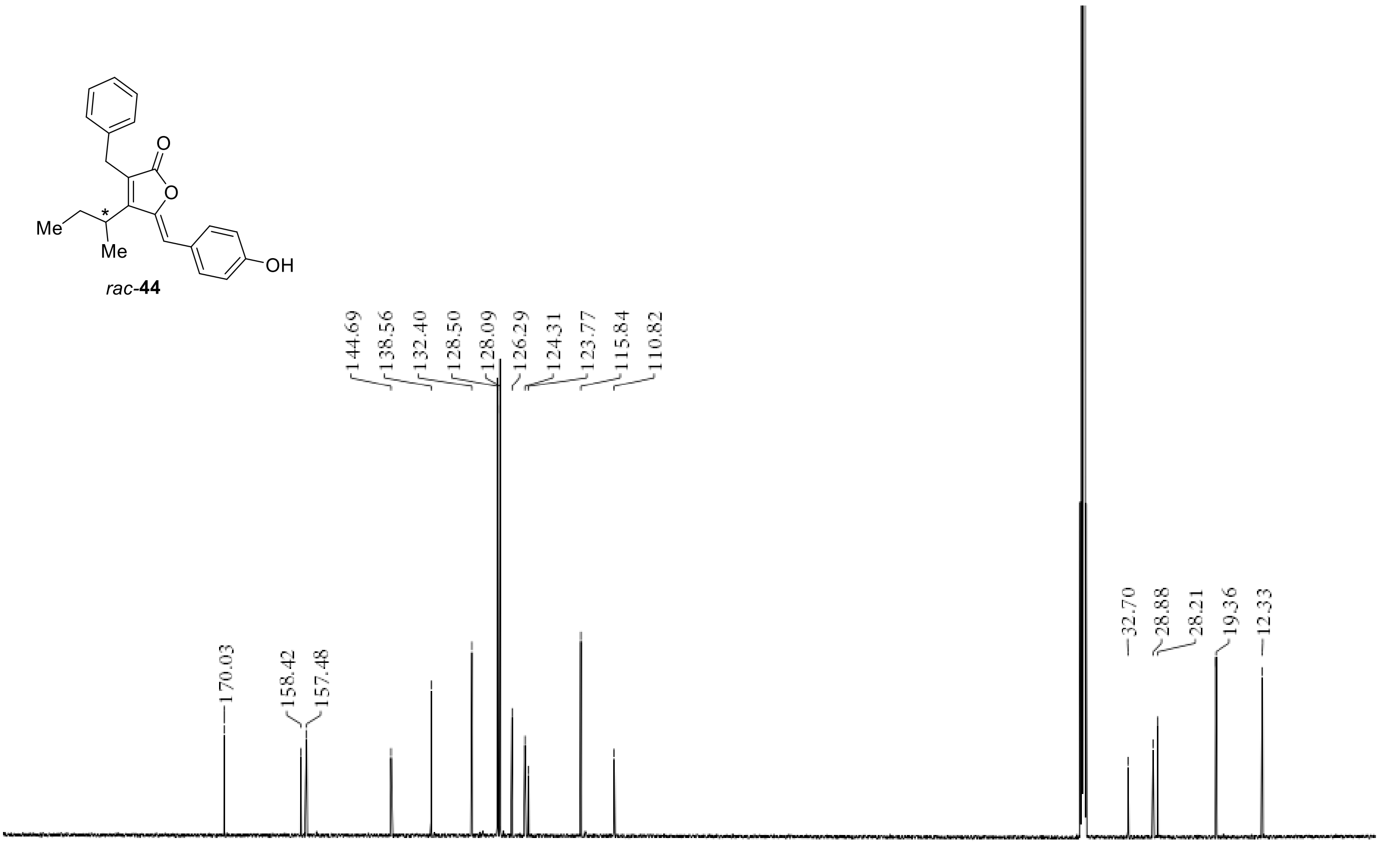

Figure S110. $^{13}C\{^{1}H\}$-NMR spectrum (151 MHz) of compound *rac*-**44**.

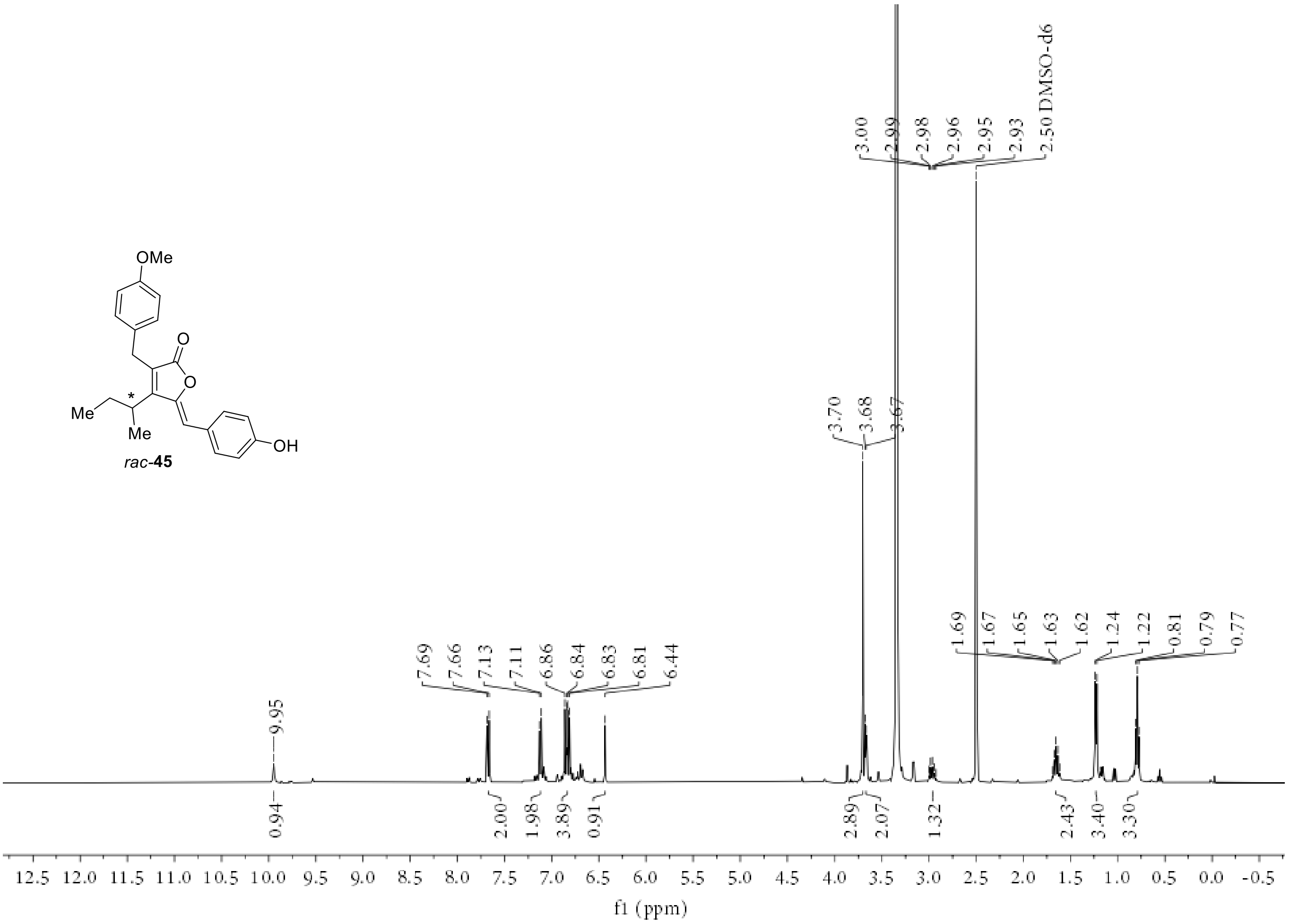

Figure S111. ^{1}H-NMR spectrum (400 MHz) of compound *rac*-**45**.

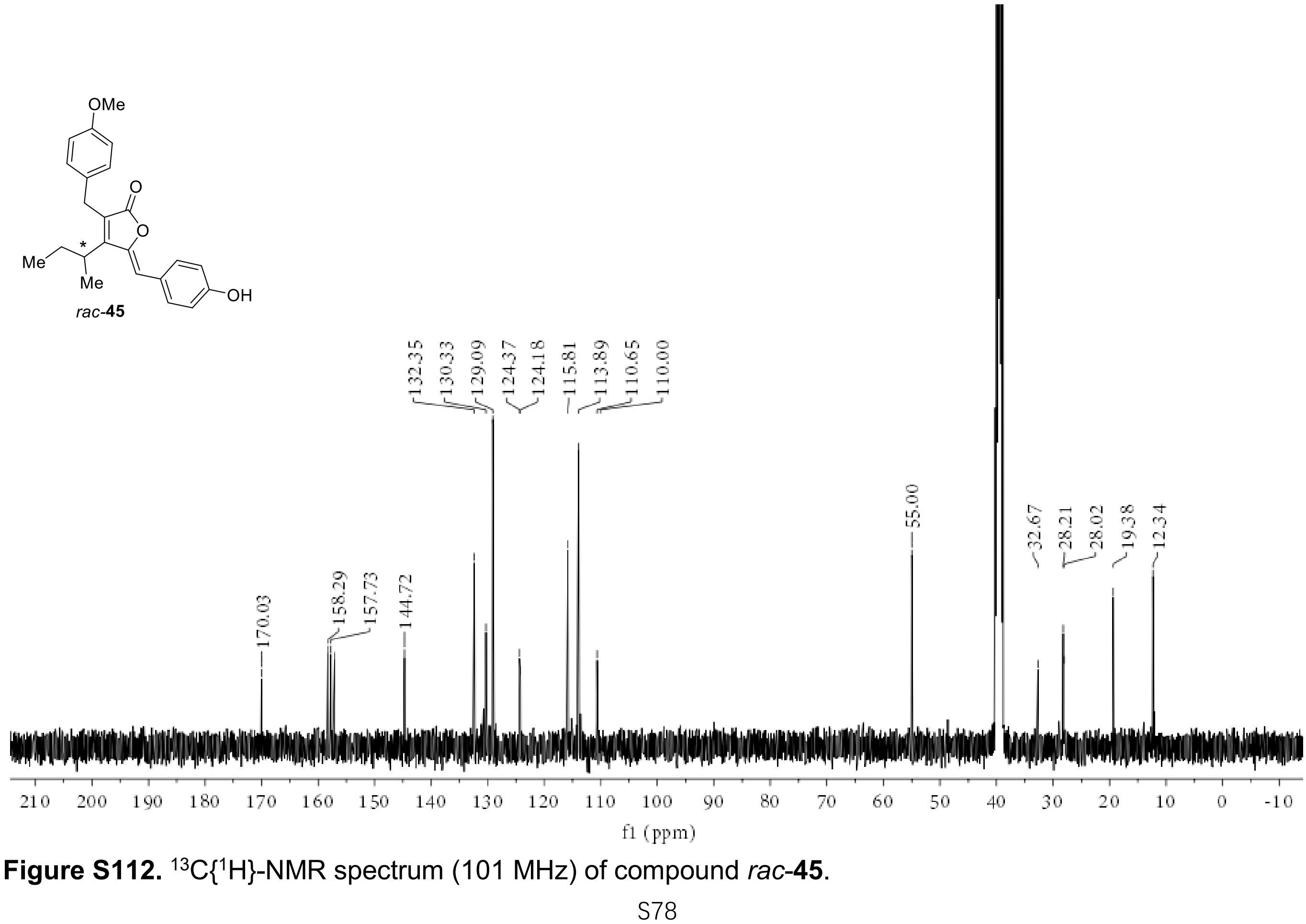

Figure S112. ^{13}C{^{1}H}-NMR spectrum (101 MHz) of compound *rac*-**45**.

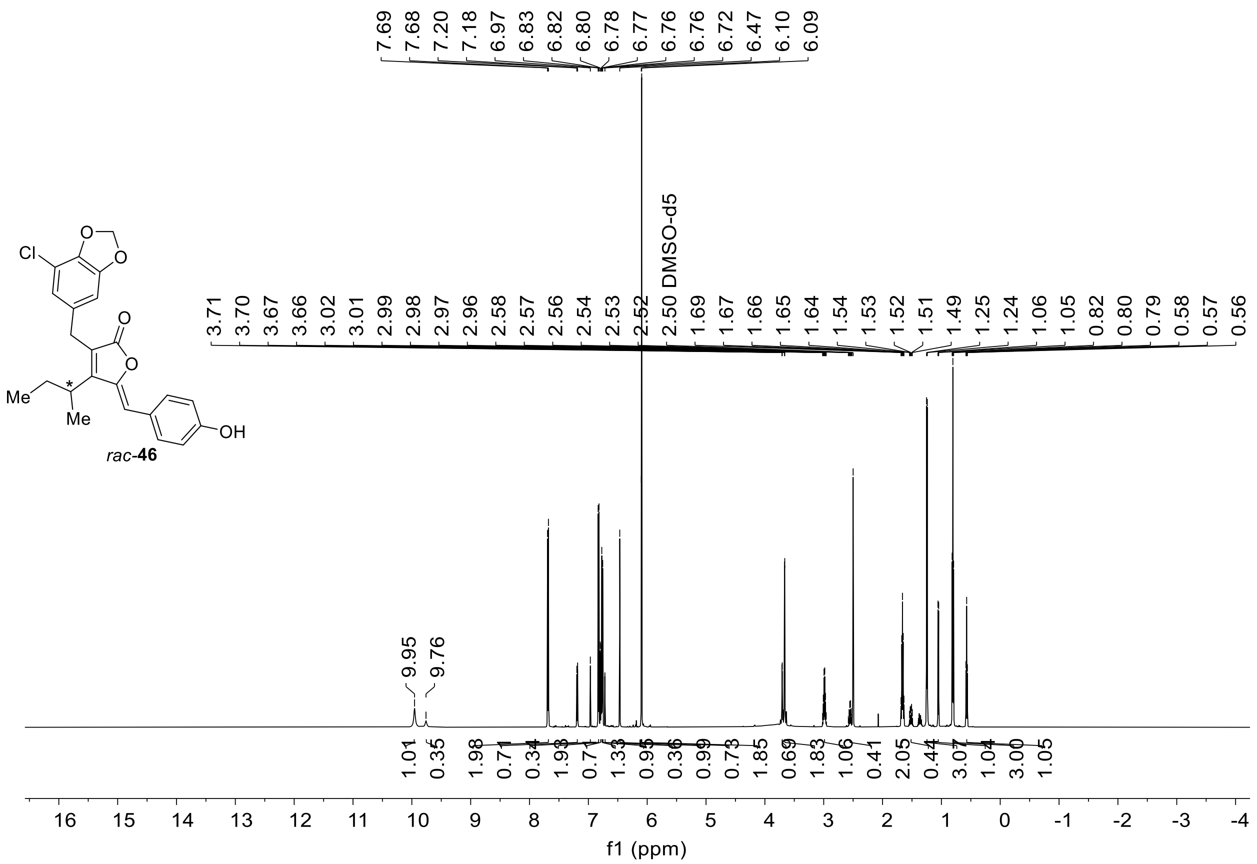

Figure S113. ^{1}H-NMR spectrum (600 MHz) of compound *rac*-**46**.

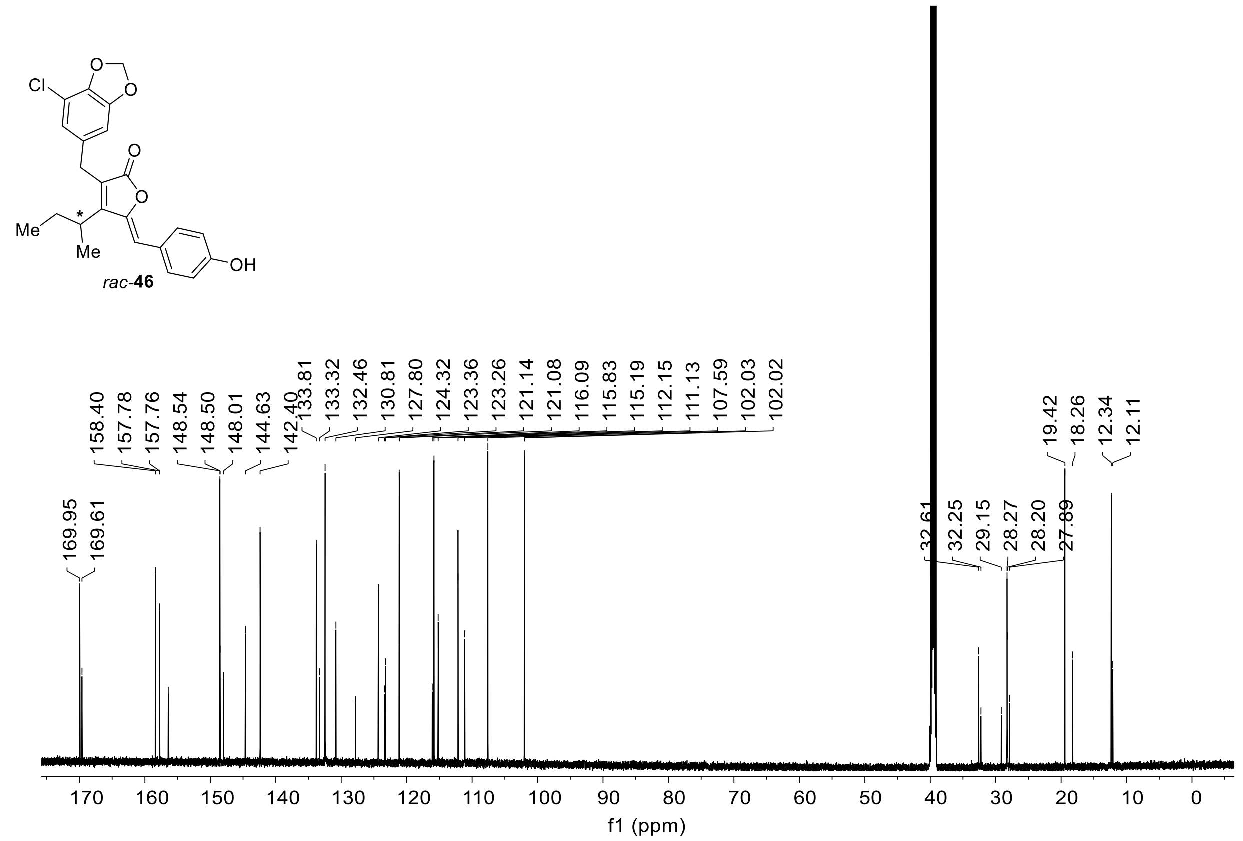

Figure S114. ^{13}C{^{1}H}-NMR spectrum (151 MHz) of compound *rac*-**46**.

7. UV and IR Spectra

A.

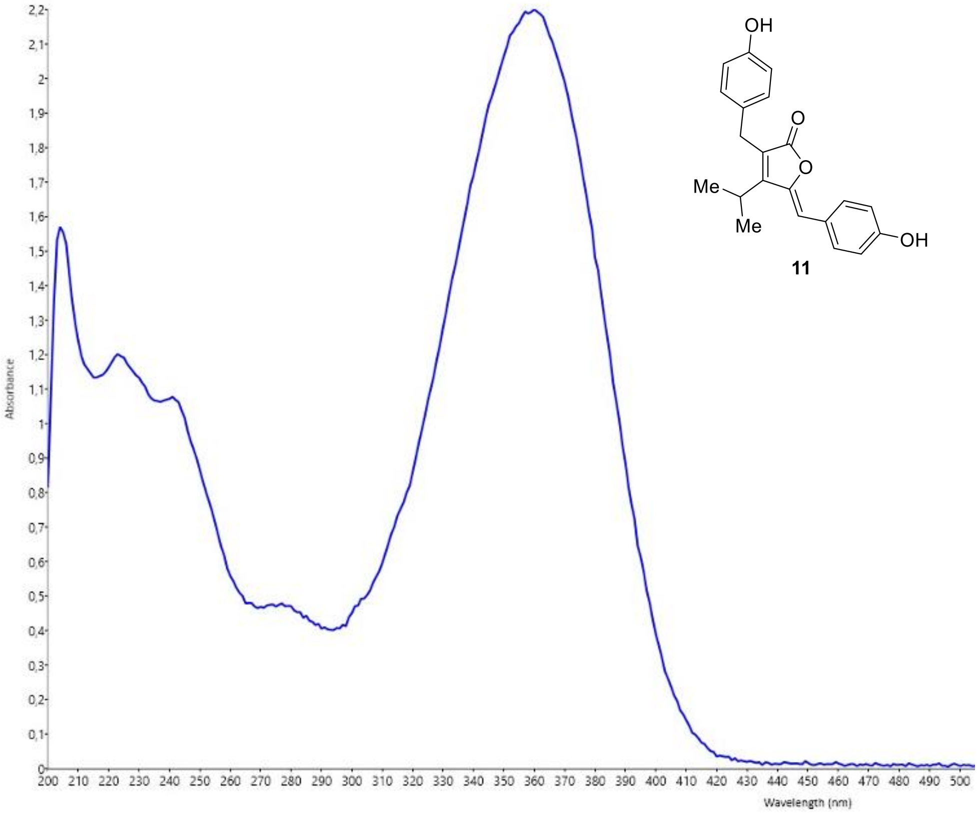

B.

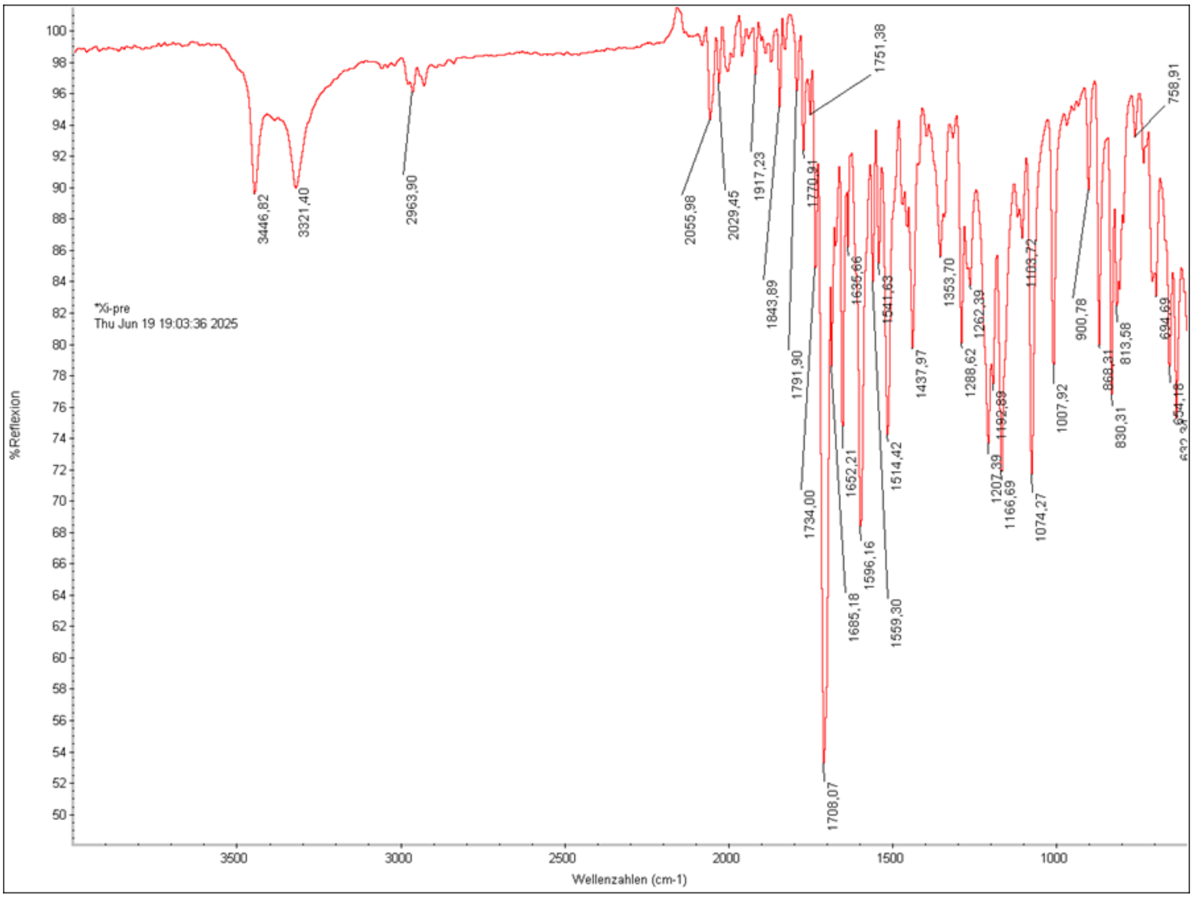

Figure S115. UV (A) and IR (B) spectra of compound **11**.

A.

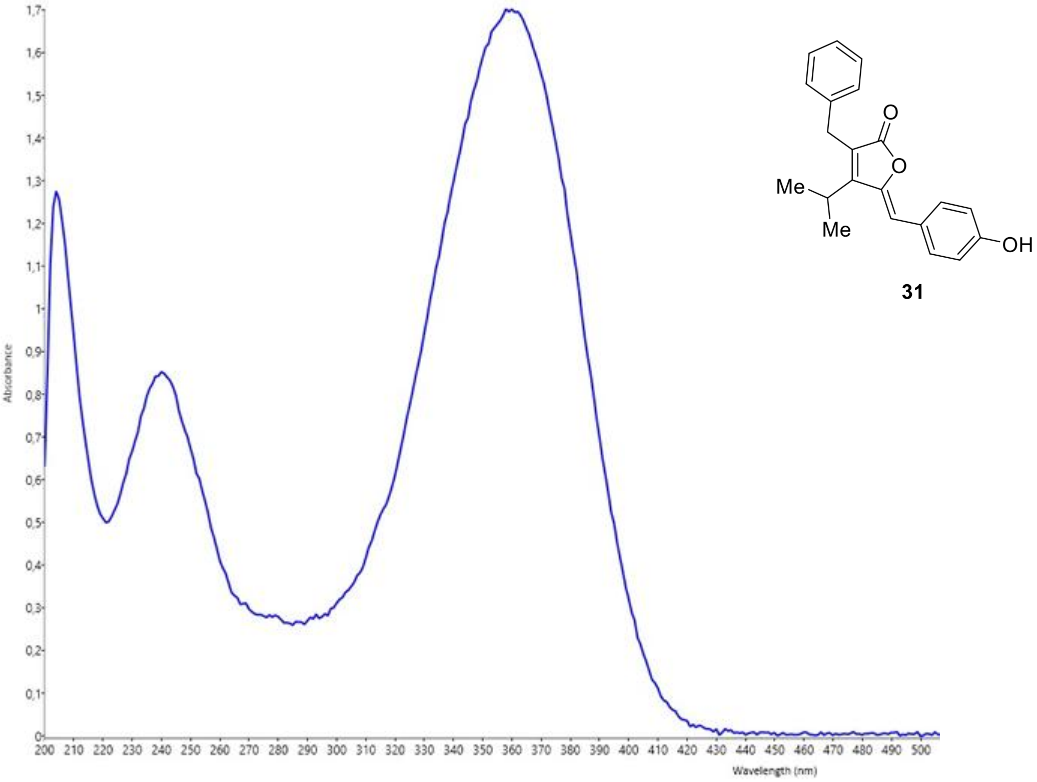

B.

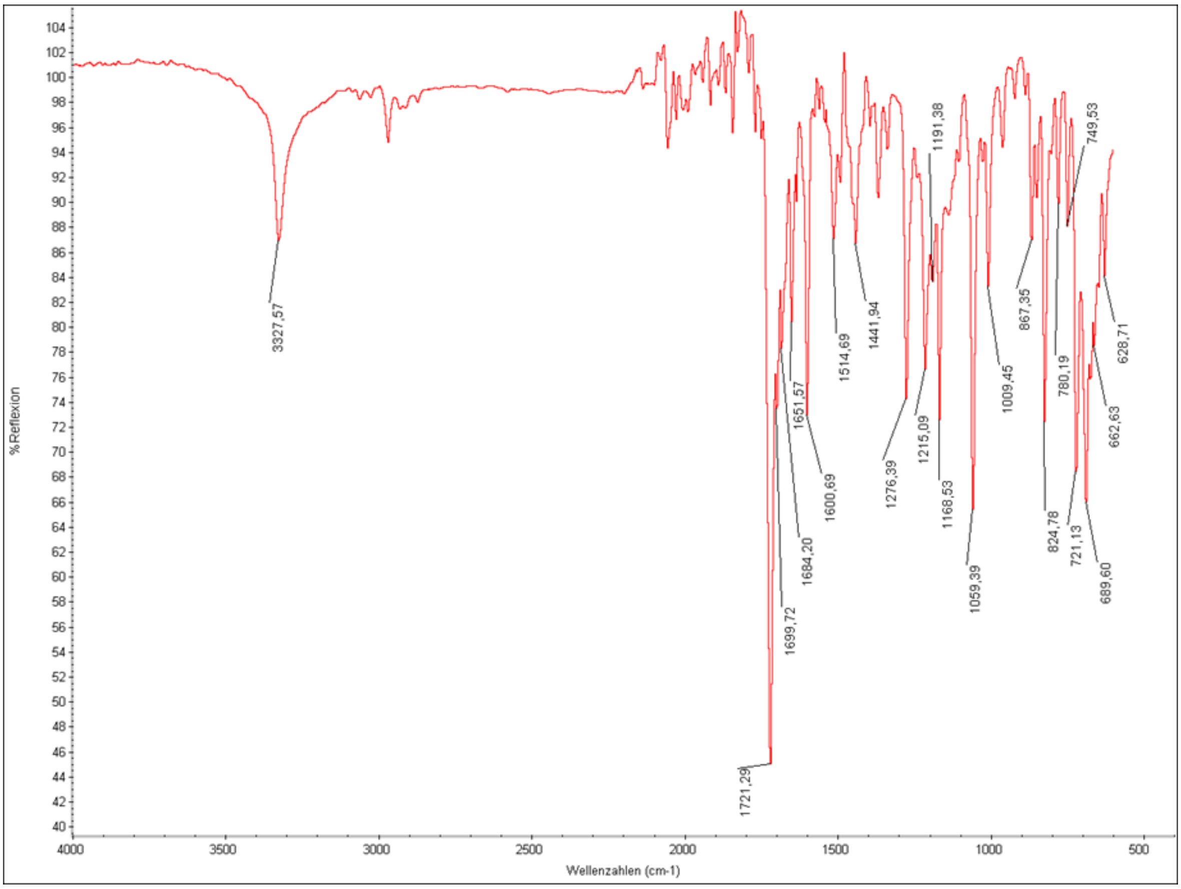

Figure S116. UV (A) and IR (B) spectra of compound **31**.

A.

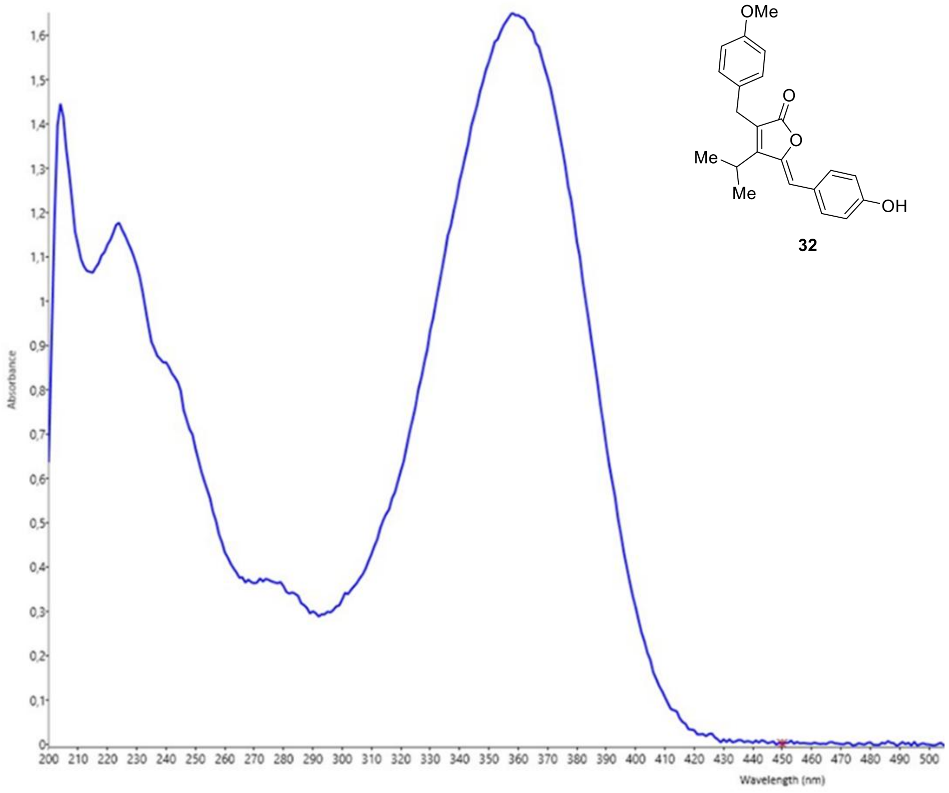

B.

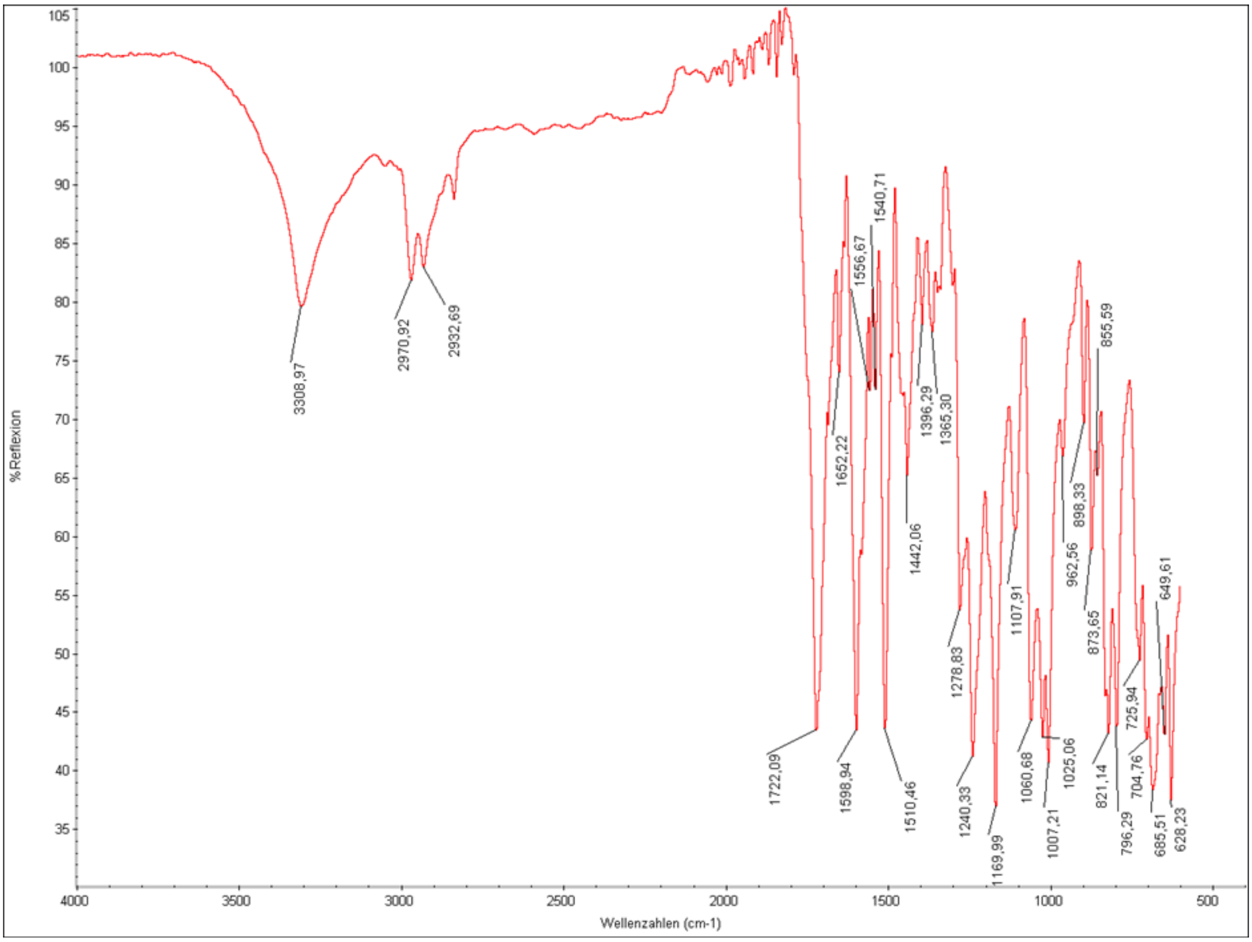

Figure S117. UV (A) and IR (B) spectra of compound **32**.

A.

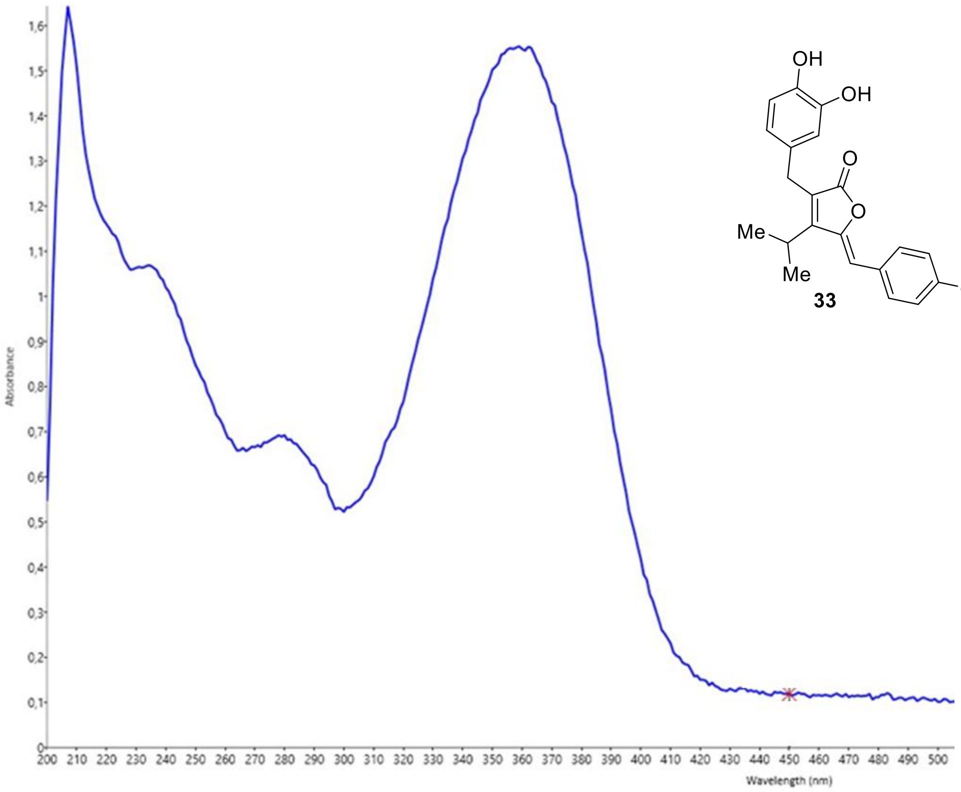

B.

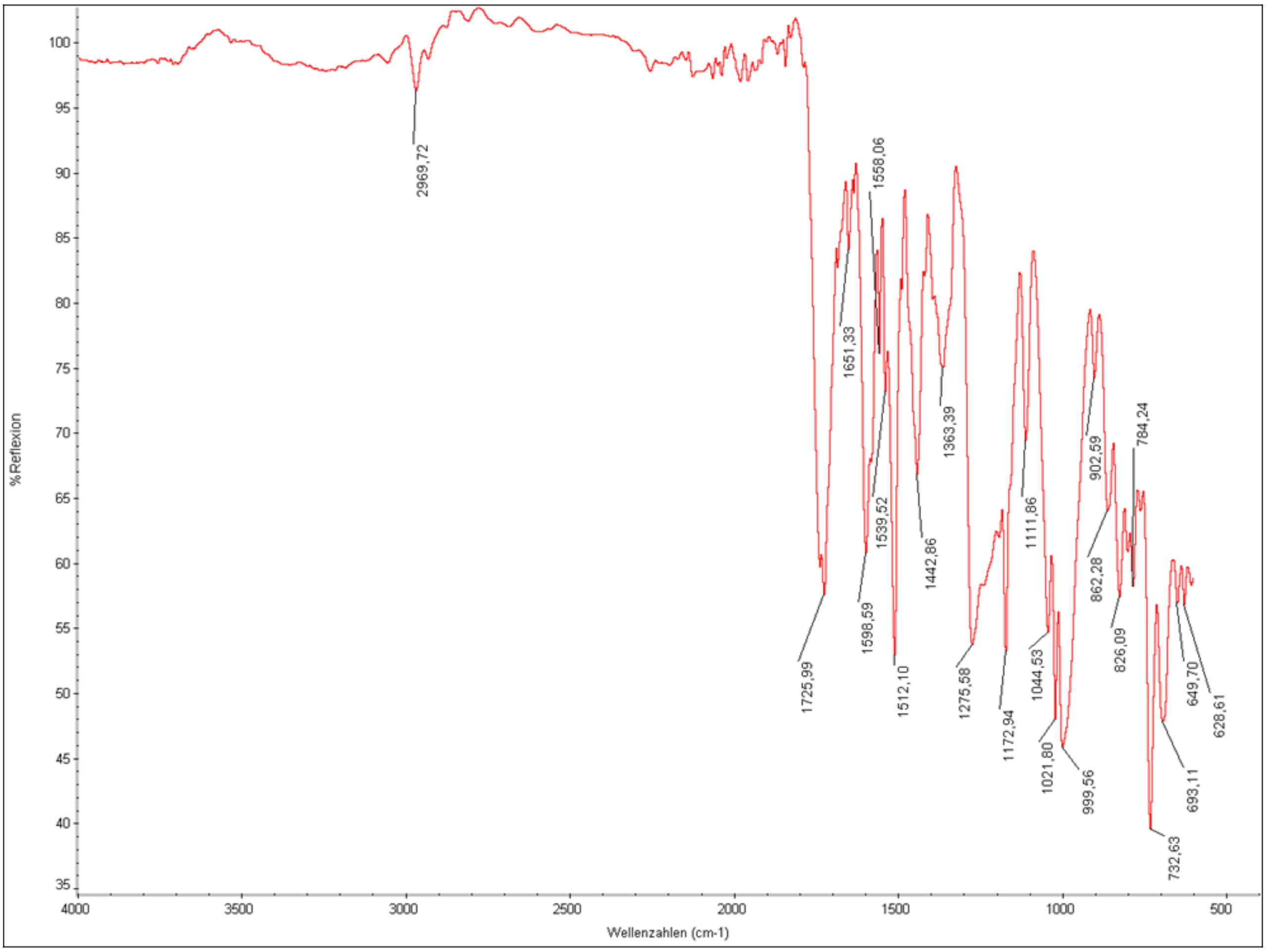

Figure S118. UV (A) and IR (B) spectra of compound **33.**

A.

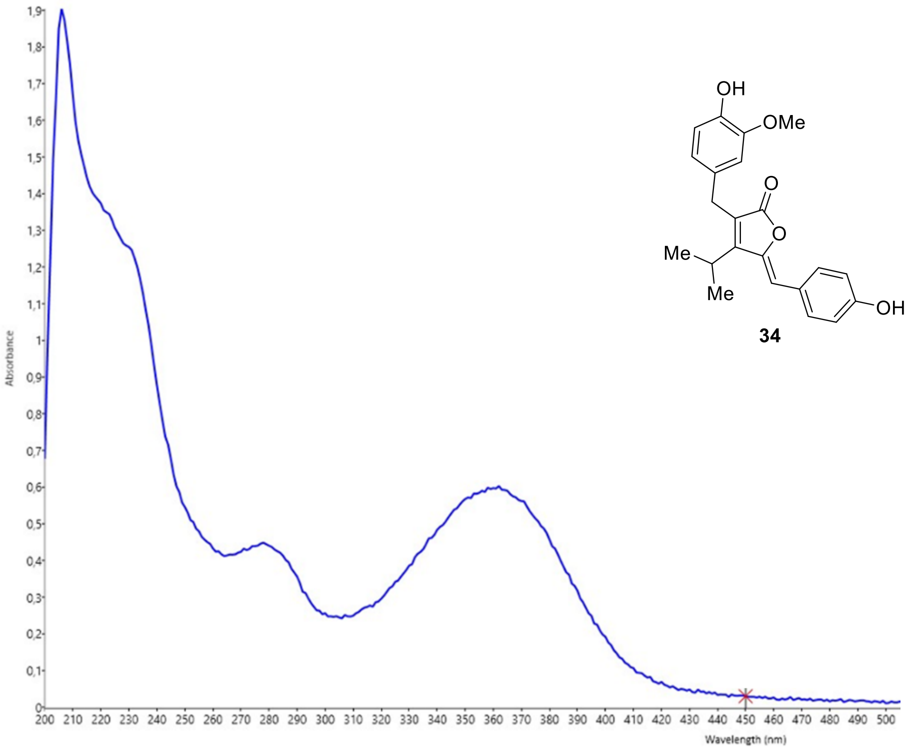

B.

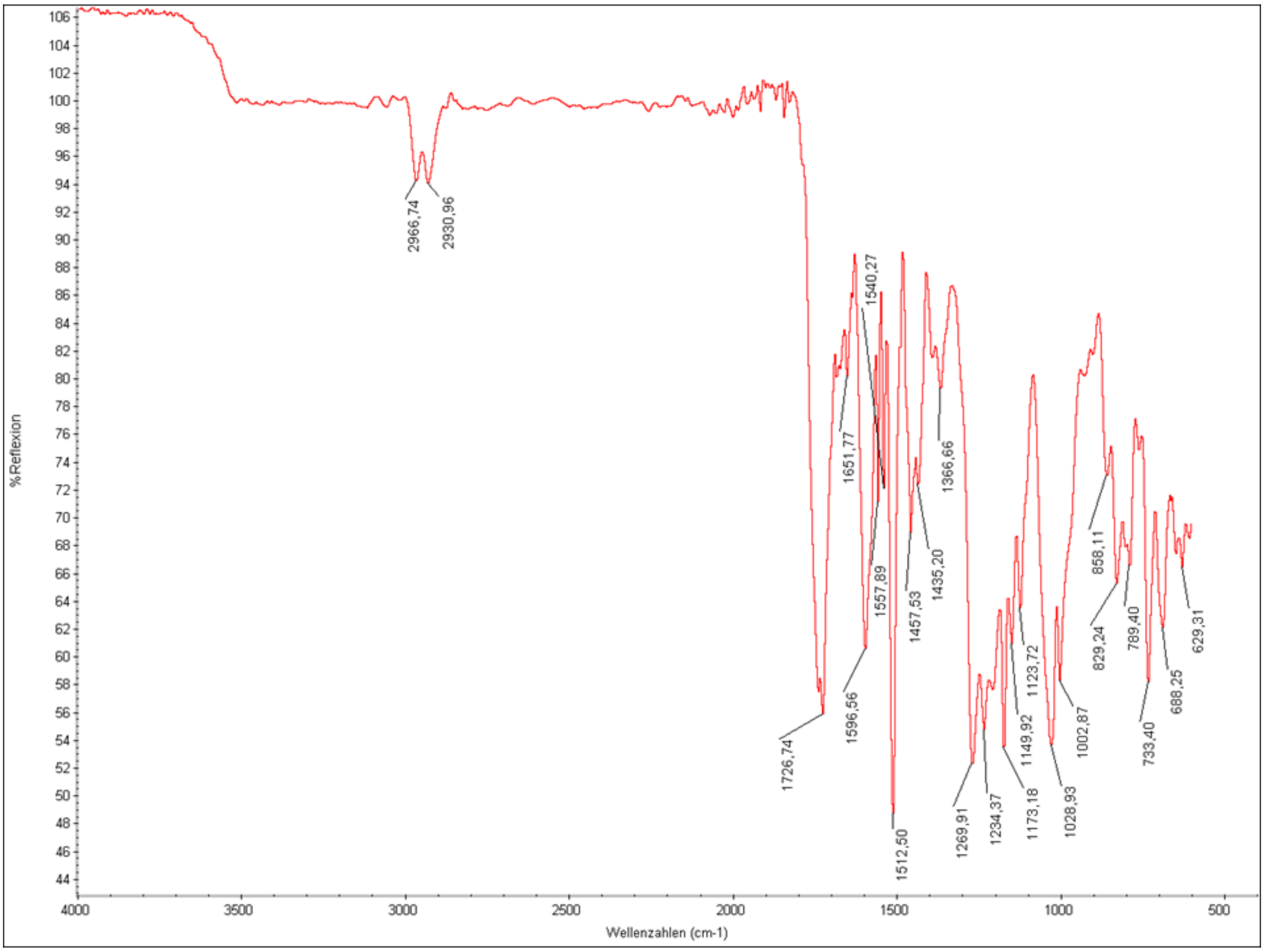

Figure S119. UV (A) and IR (B) spectra of compound **34**.

A.

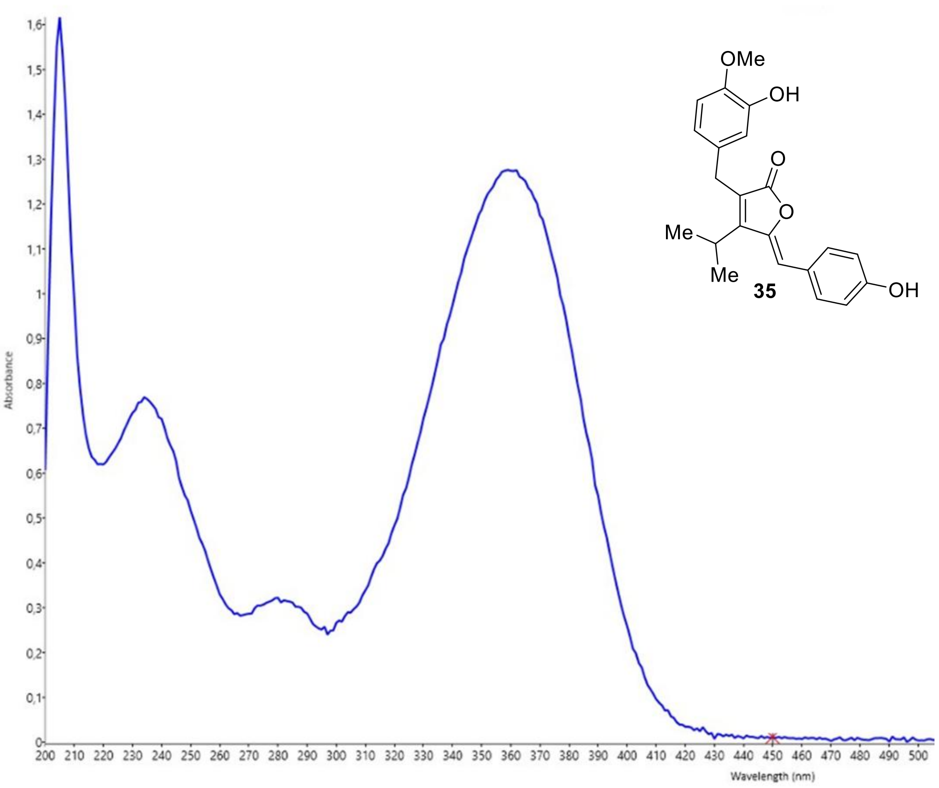

B.

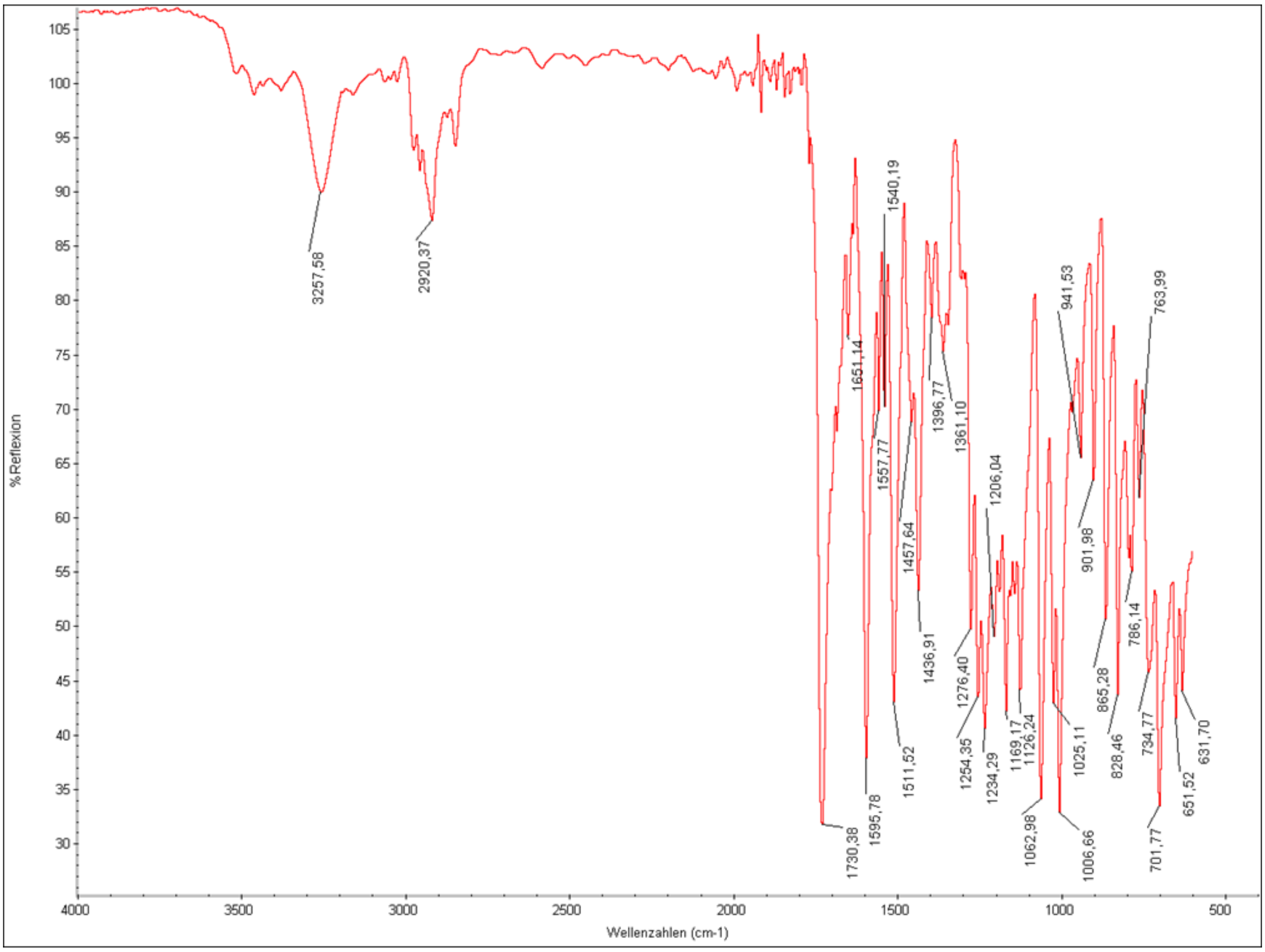

Figure S120. UV (A) and IR (B) spectra of compound **35**.

A.

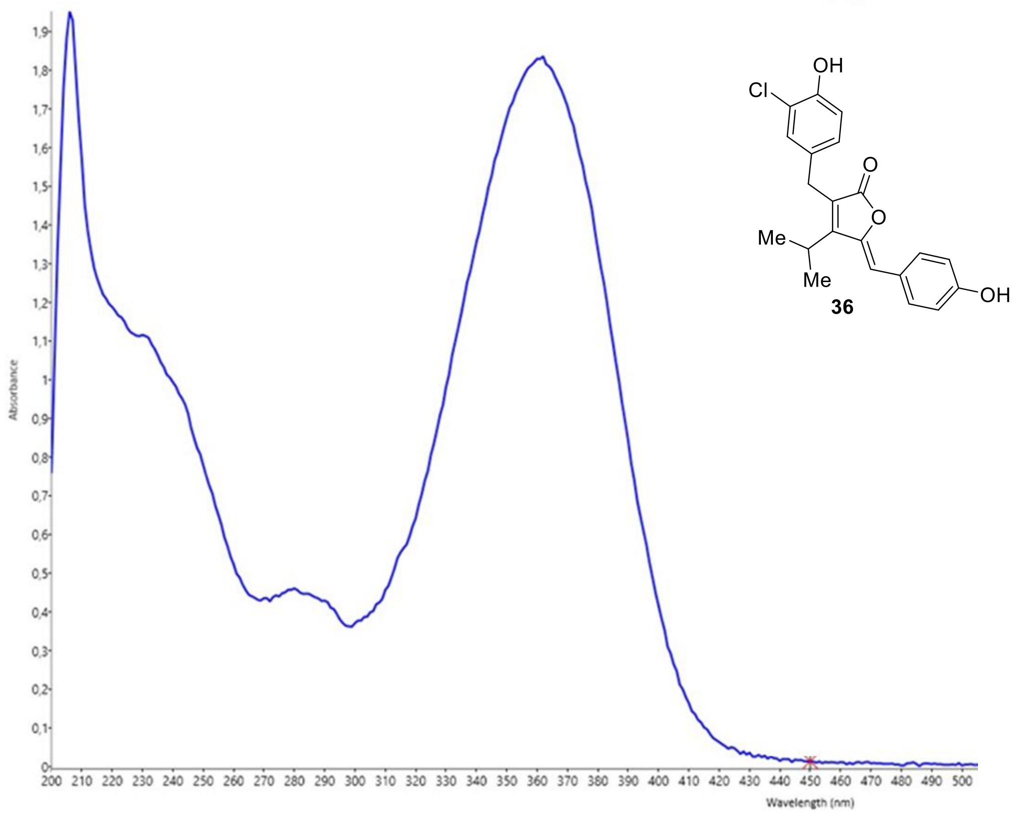

B.

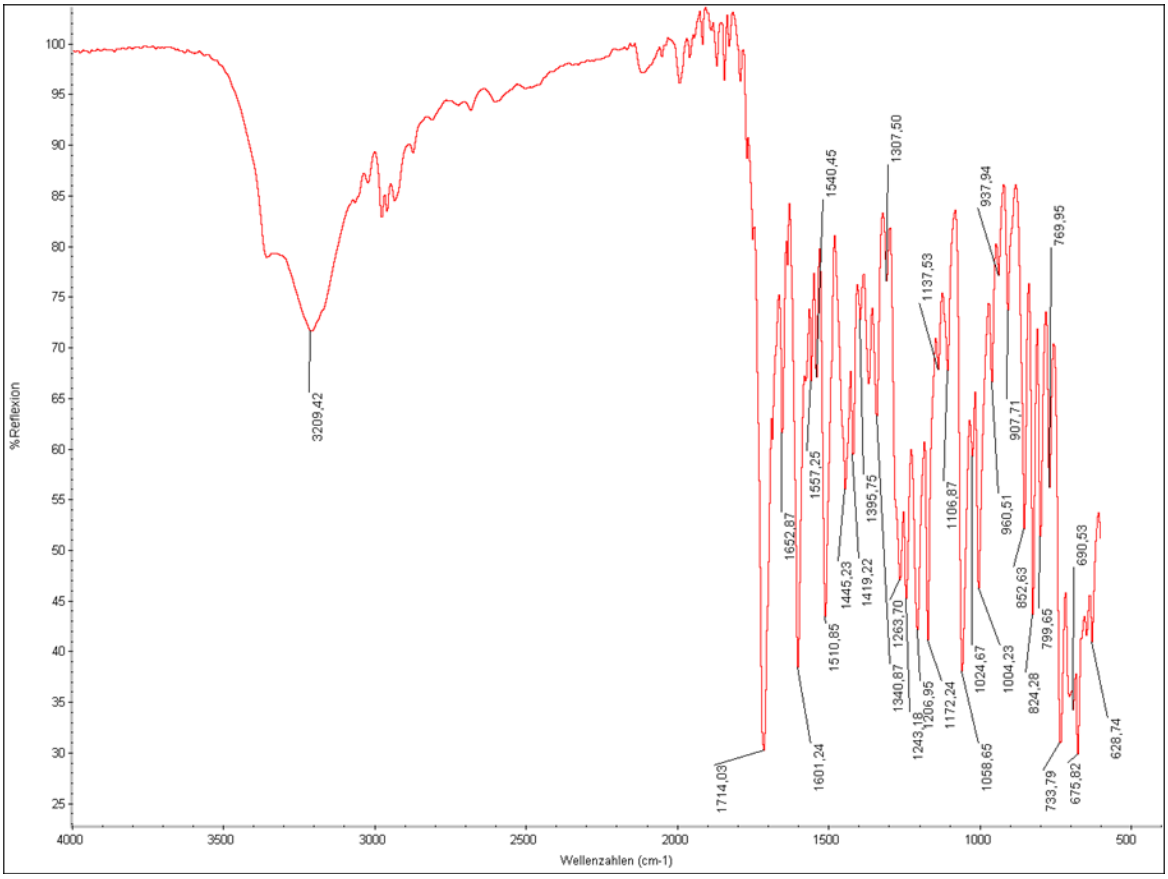

Figure S121. UV (A) and IR (B) spectra of compound **36**.

A.

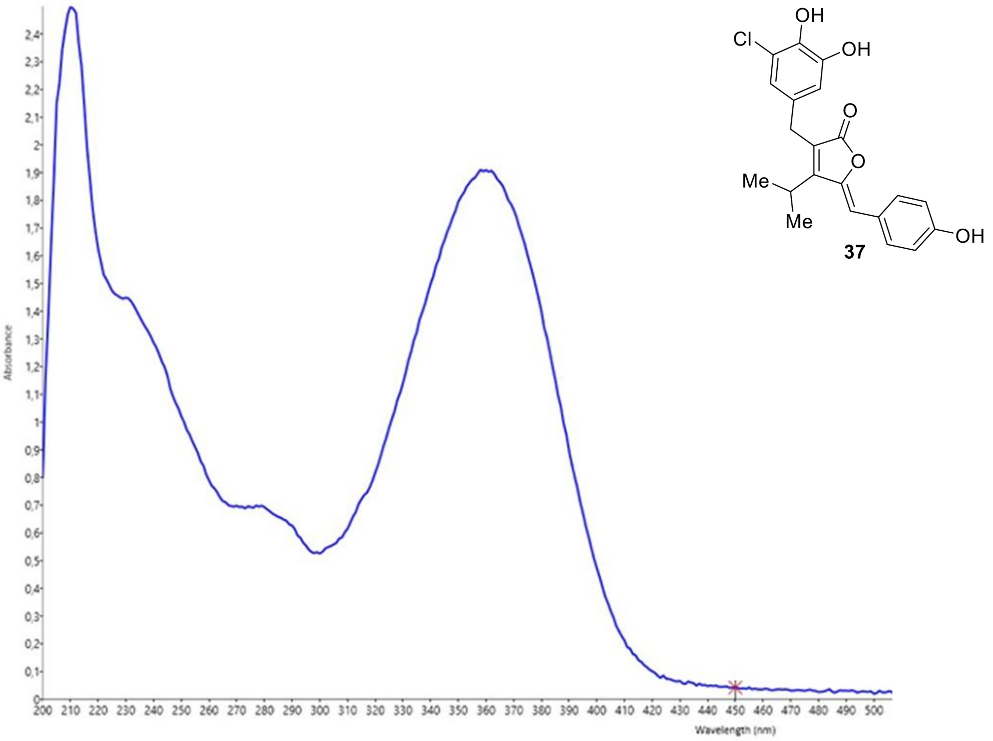

B.

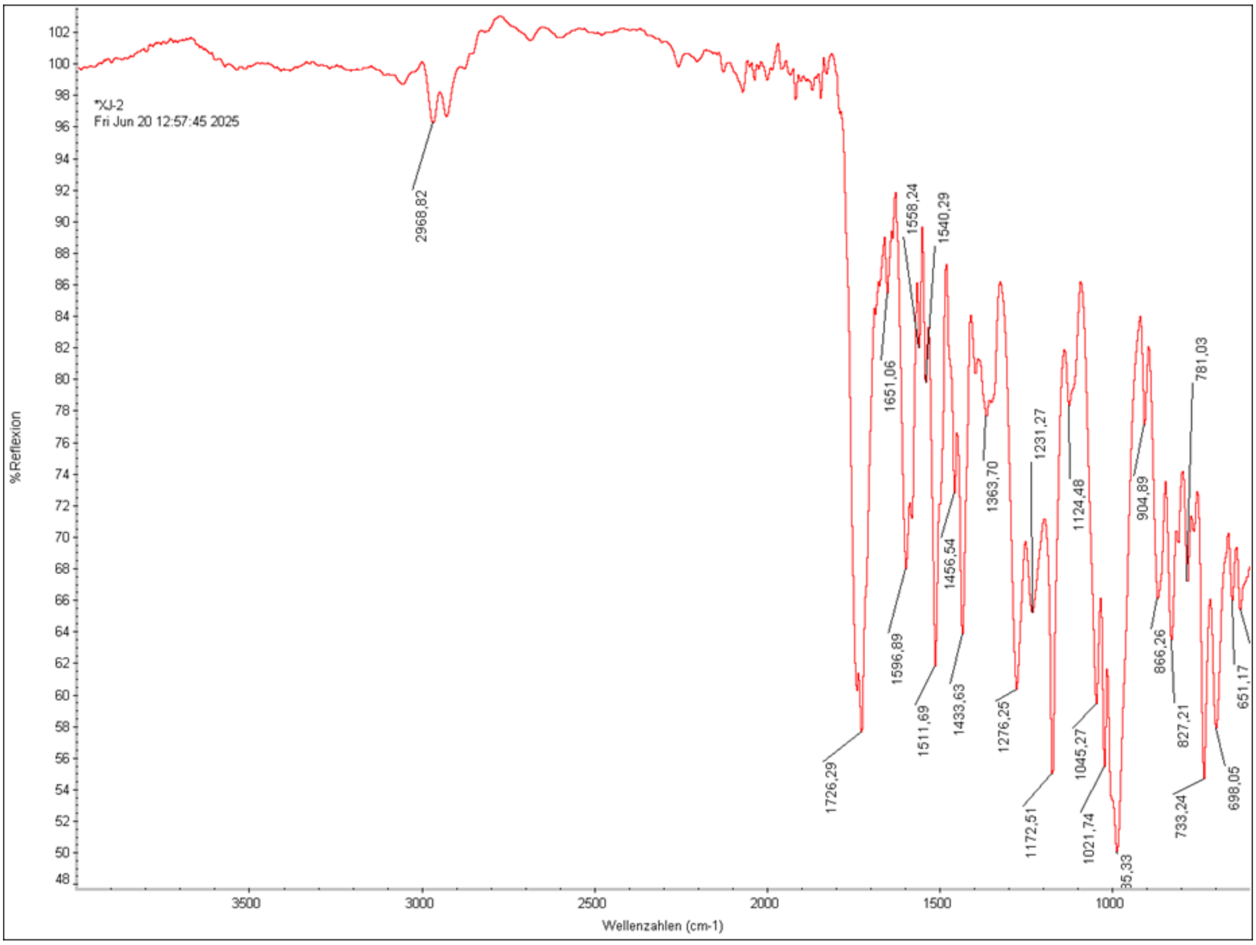

Figure S122. UV (A) and IR (B) spectra of compound **37**.

A.

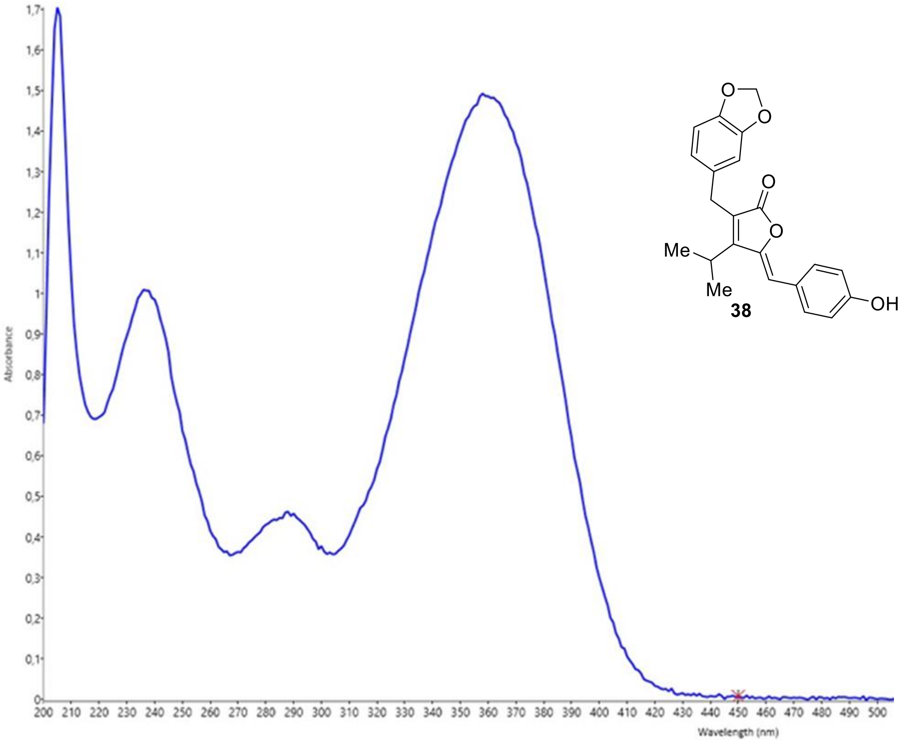

B.

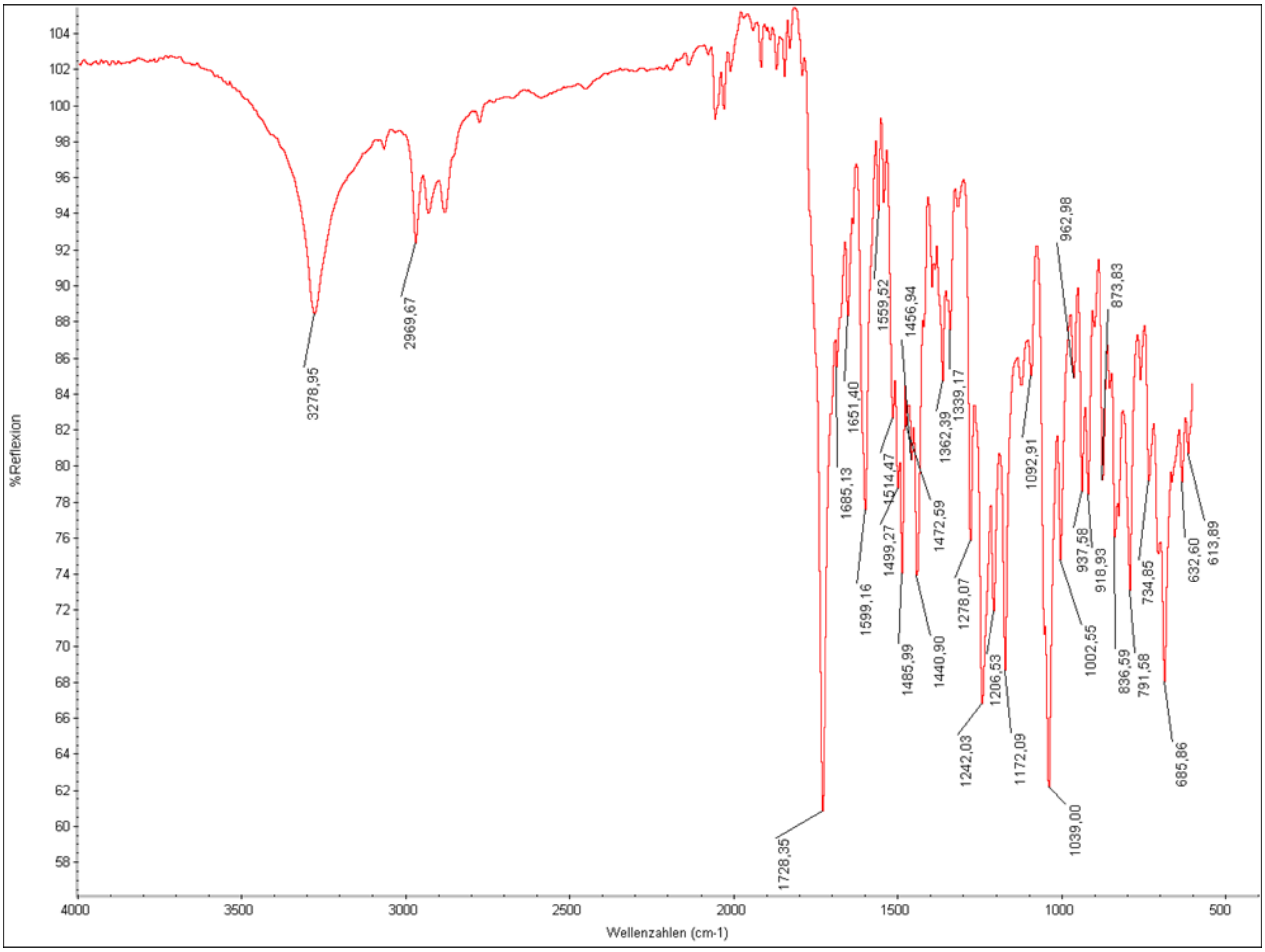

Figure S123. UV (A) and IR (B) spectra of compound **38**.

A.

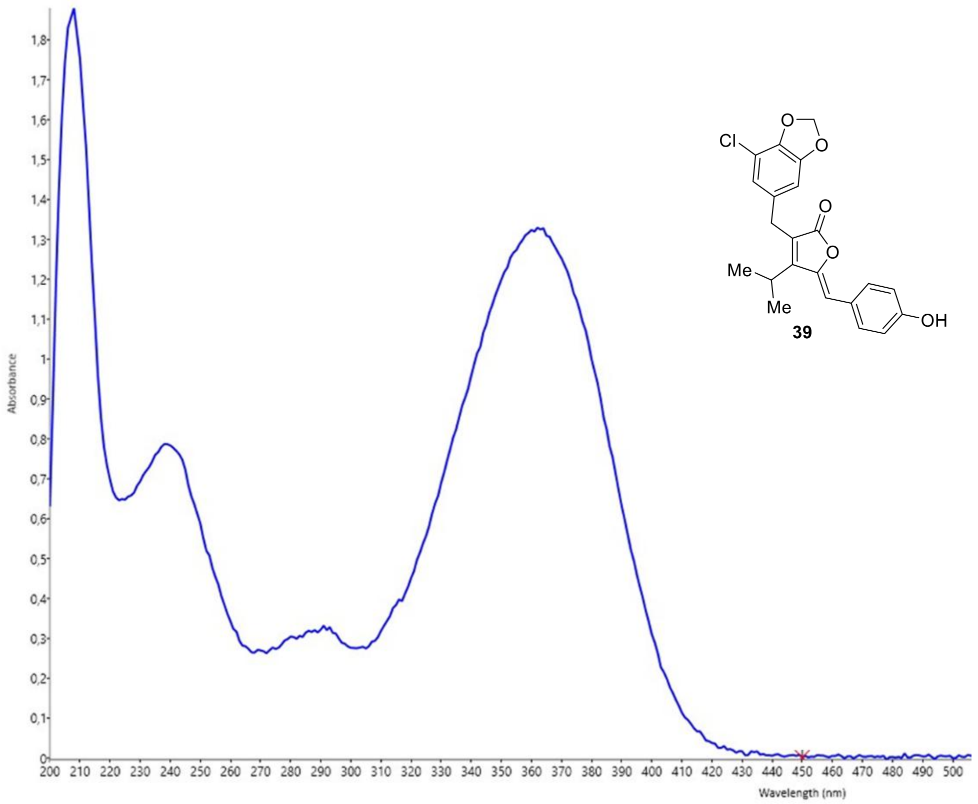

B.

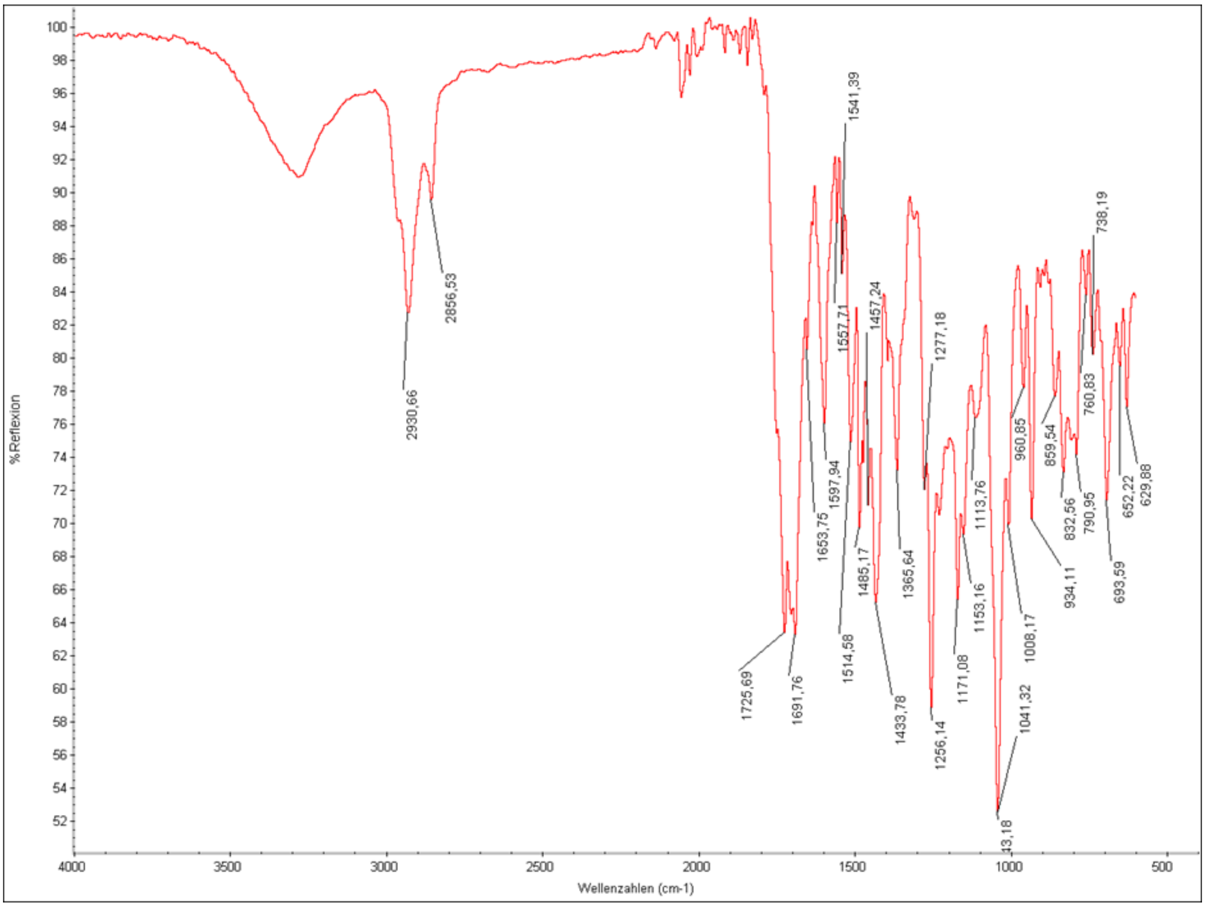

Figure S124. UV (A) and IR (B) spectra of compound **39**.

A.

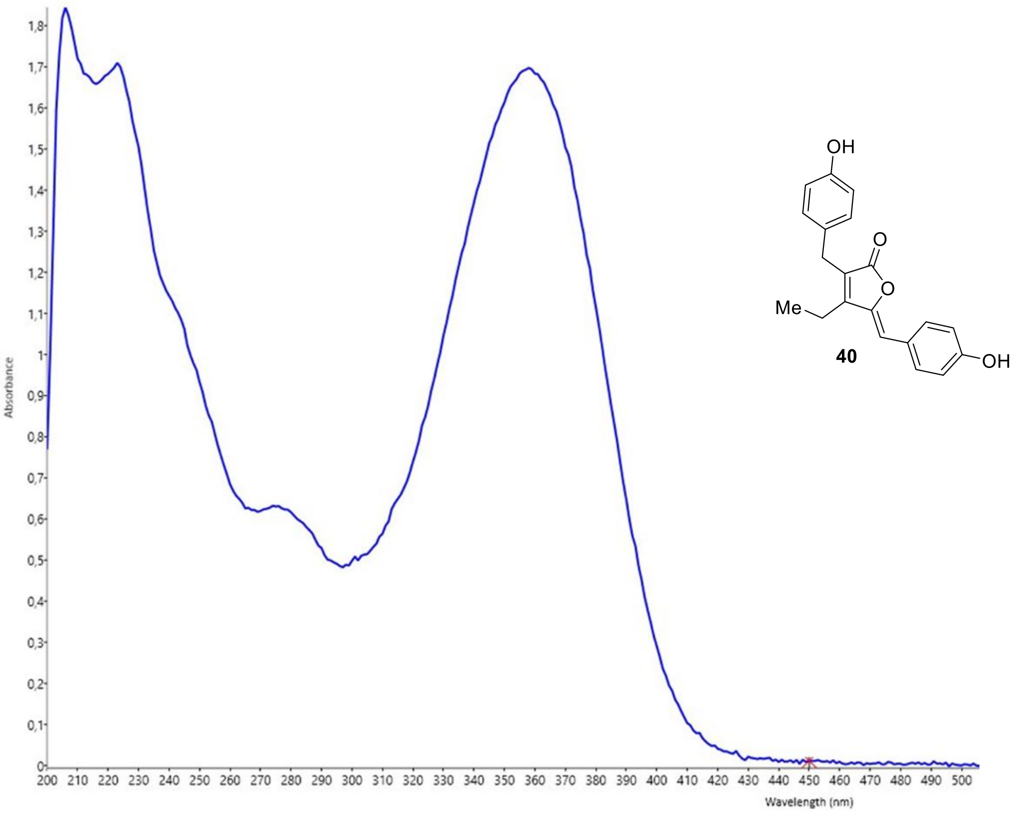

B.

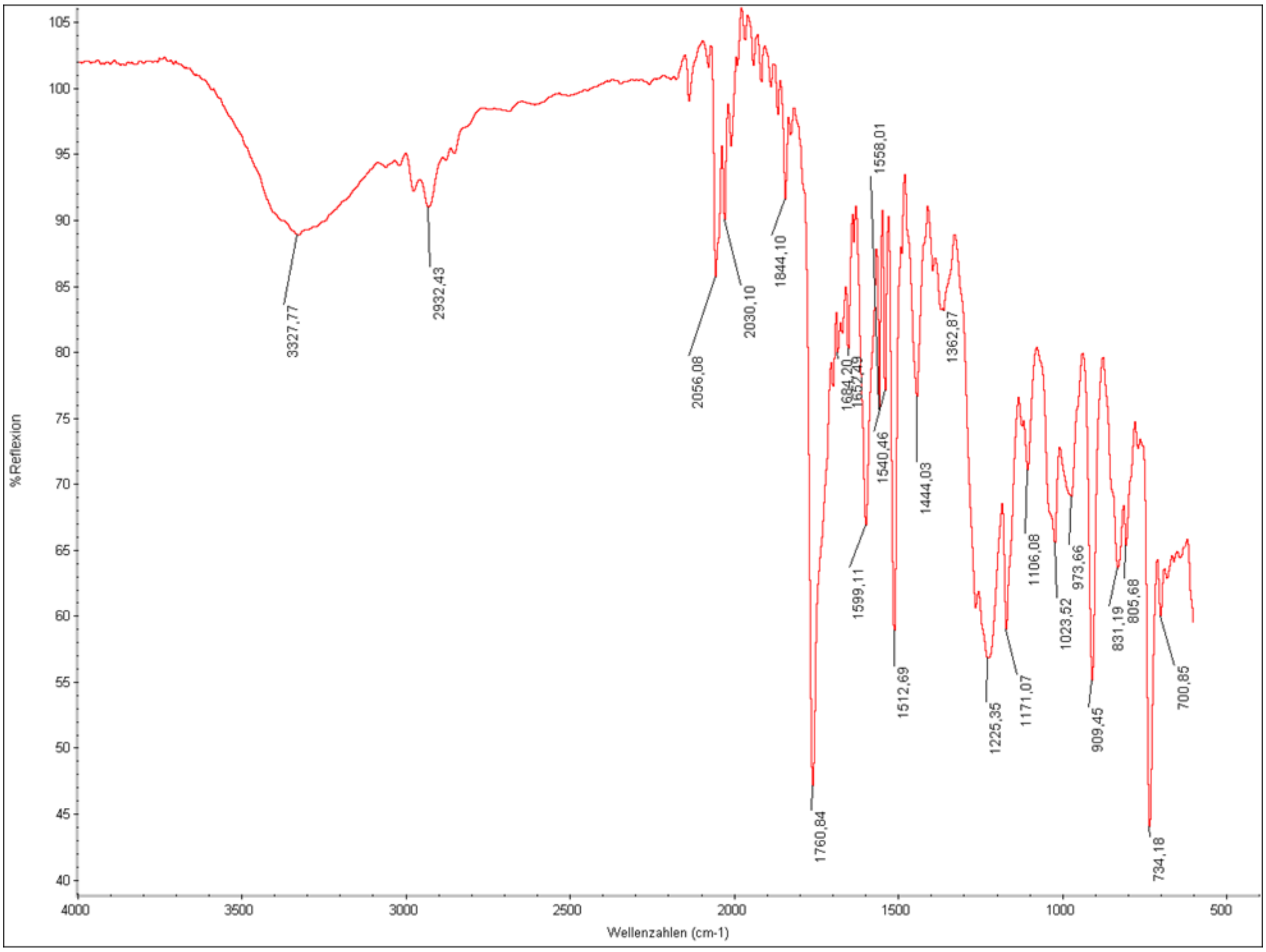

Figure S125. UV (A) and IR (B) spectra of compound **40**.

A.

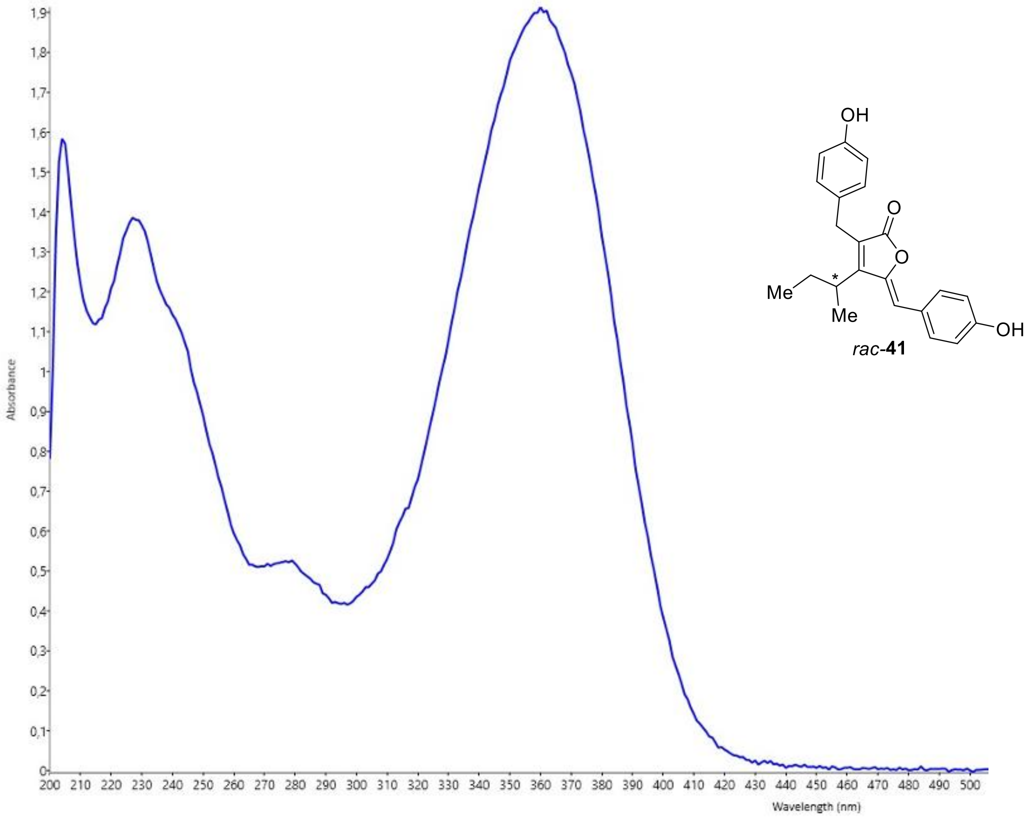

rac-**41**

B.

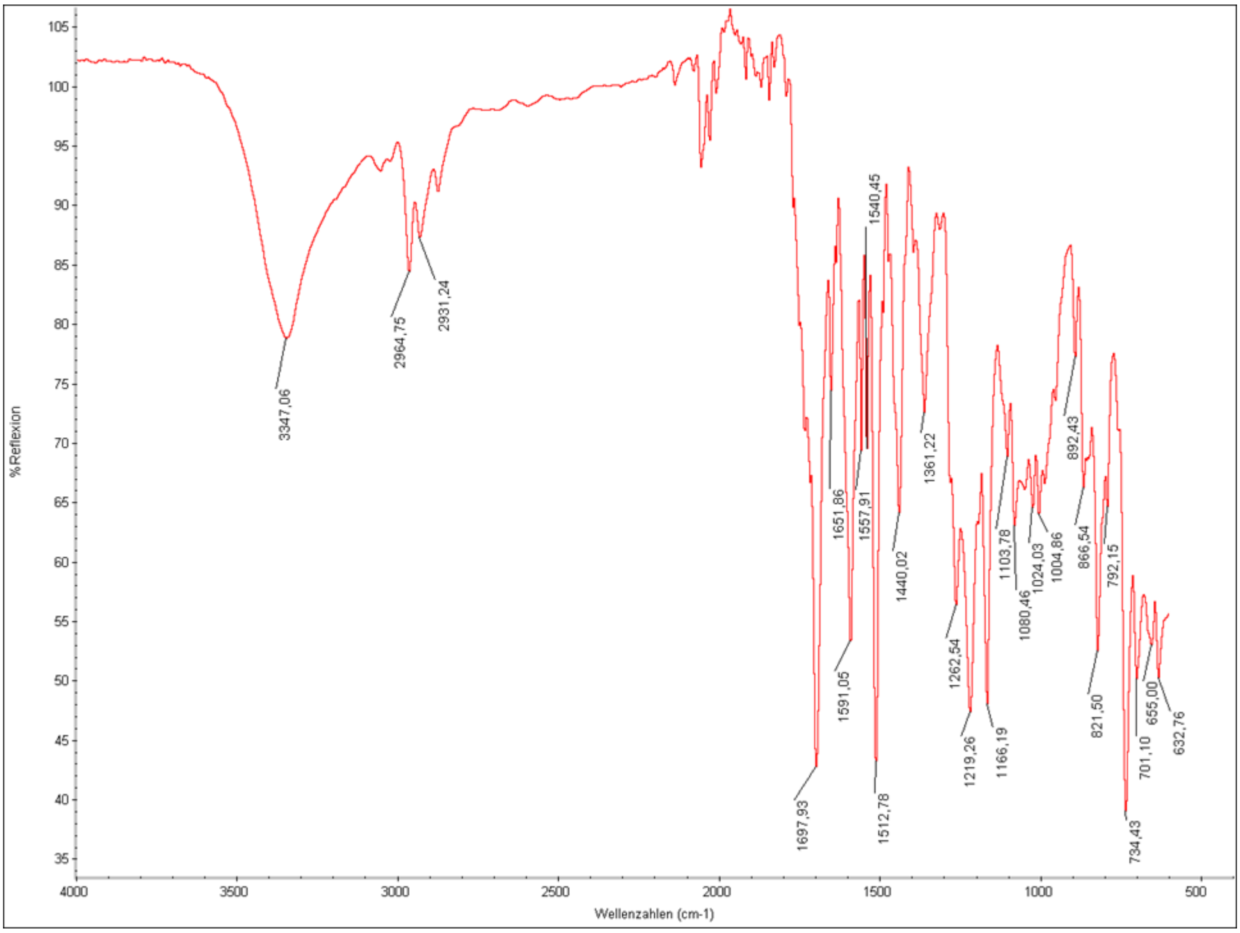

Figure S126. UV (A) and IR (B) spectra of compound *rac*-**41**.

A.

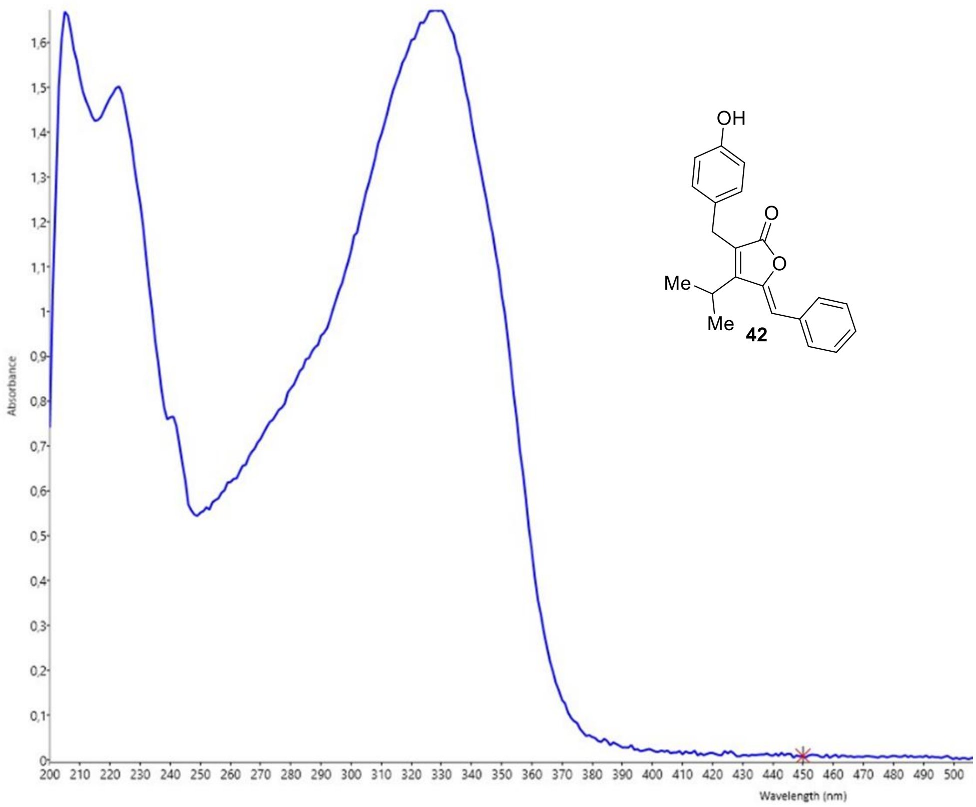

B.

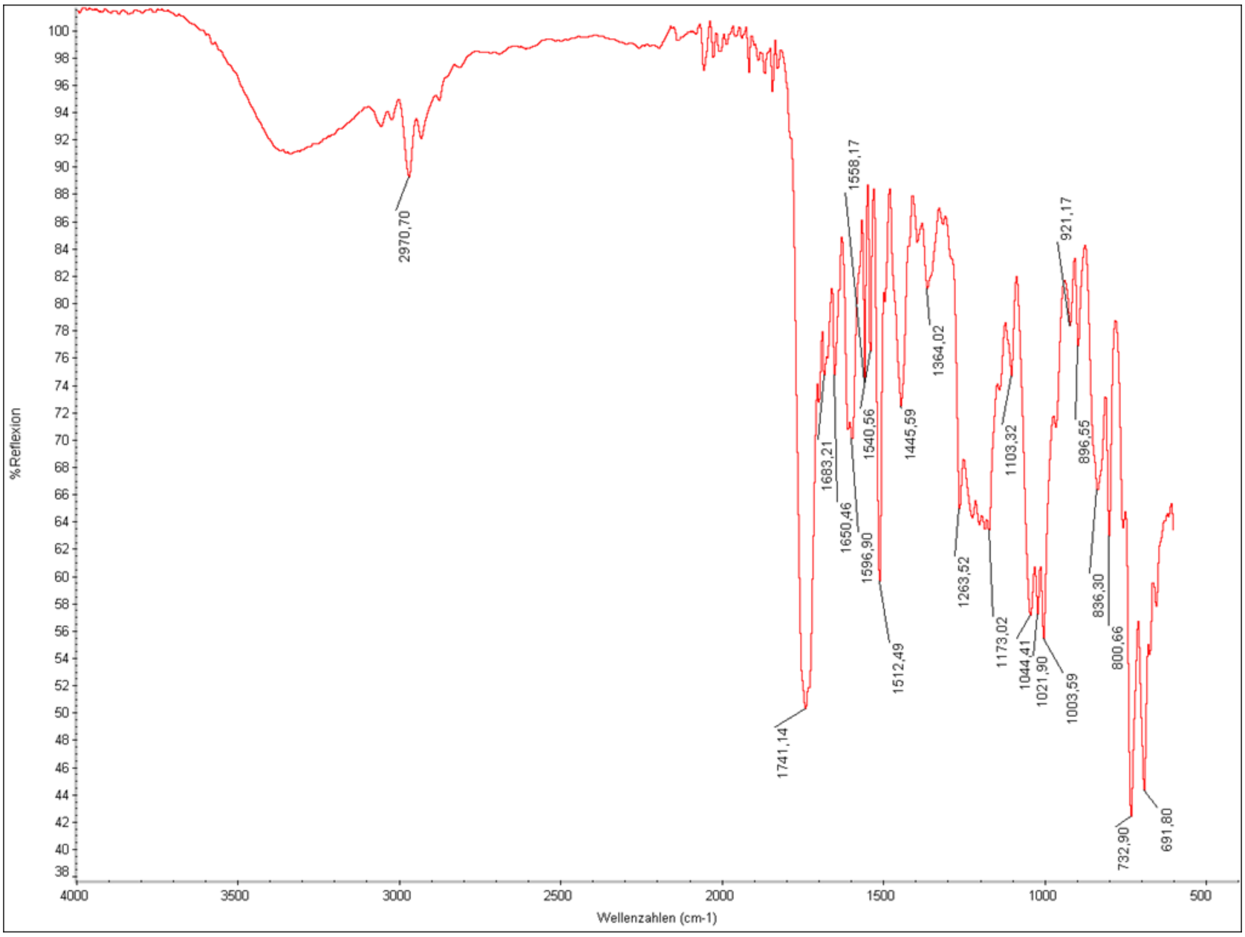

Figure S127. UV (A) and IR (B) spectra of compound **42**.

A.

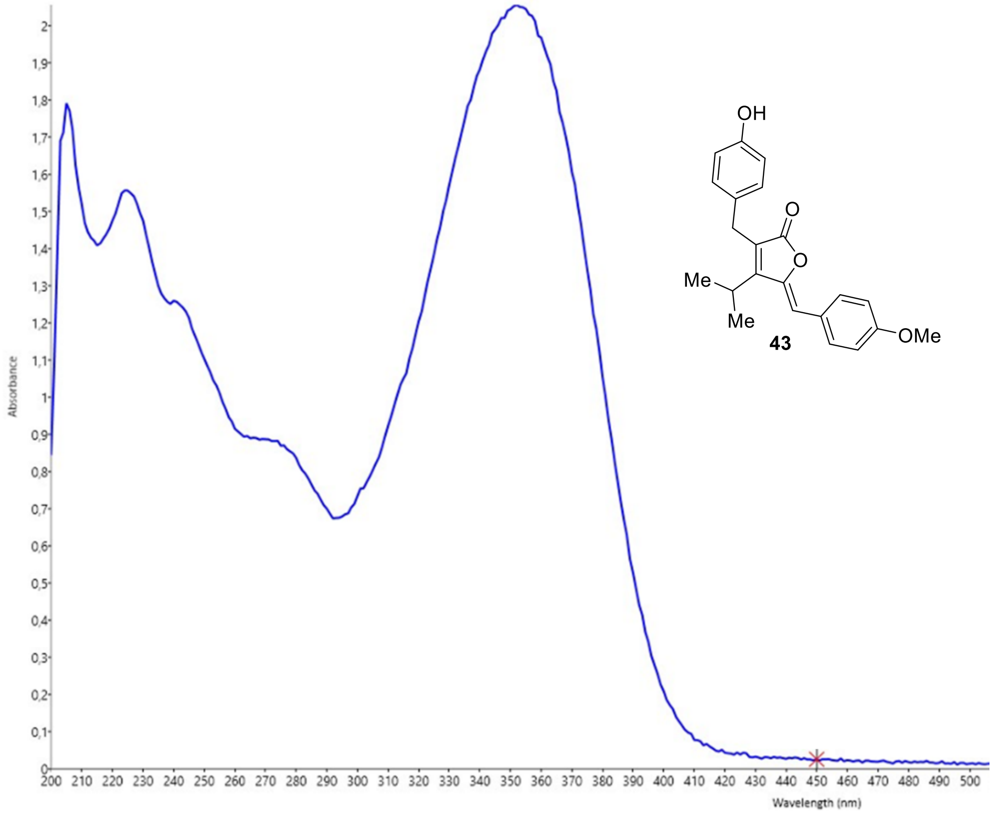

B.

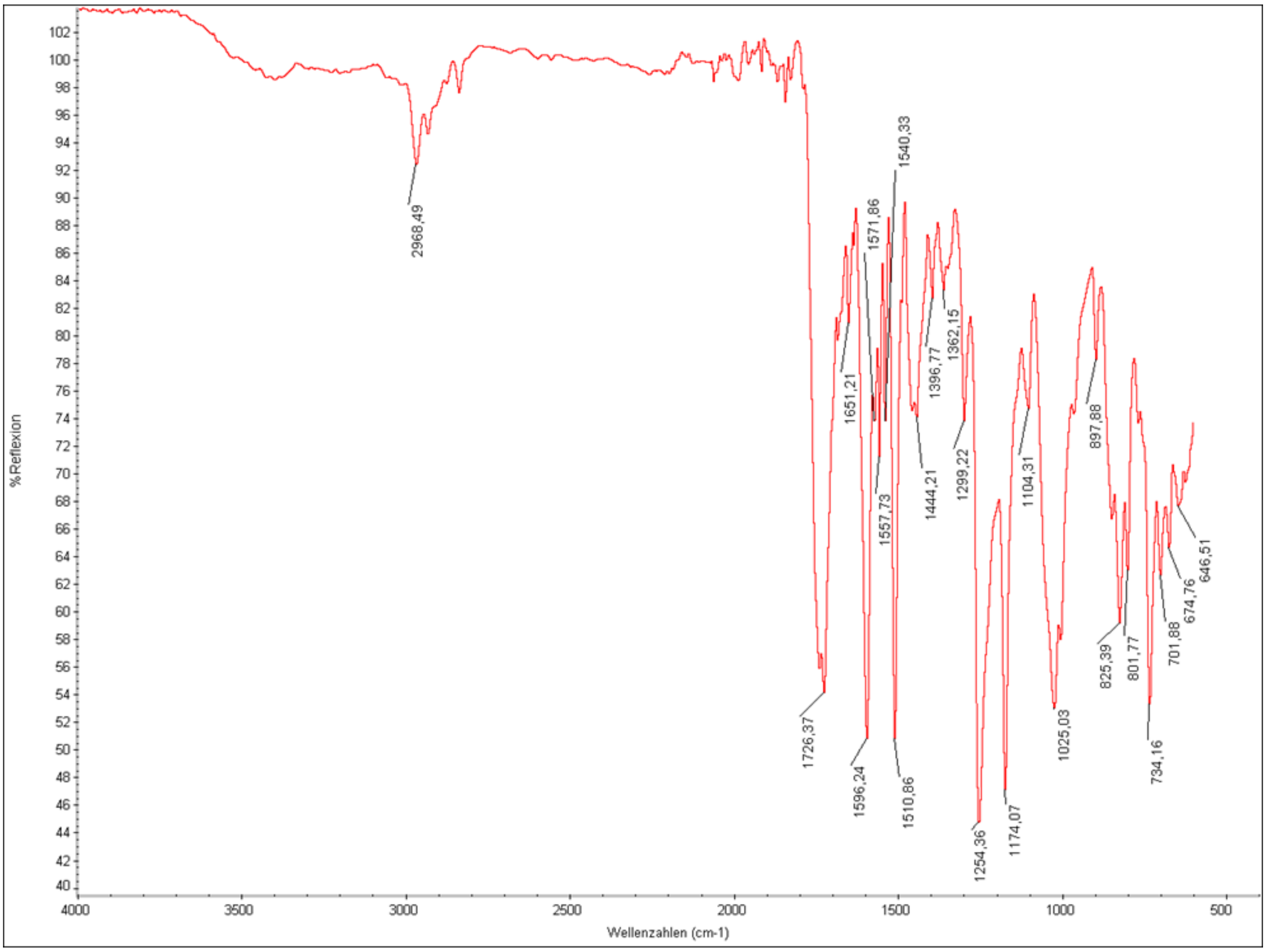

Figure S128. UV (A) and IR (B) spectra of compound **43**.

A.

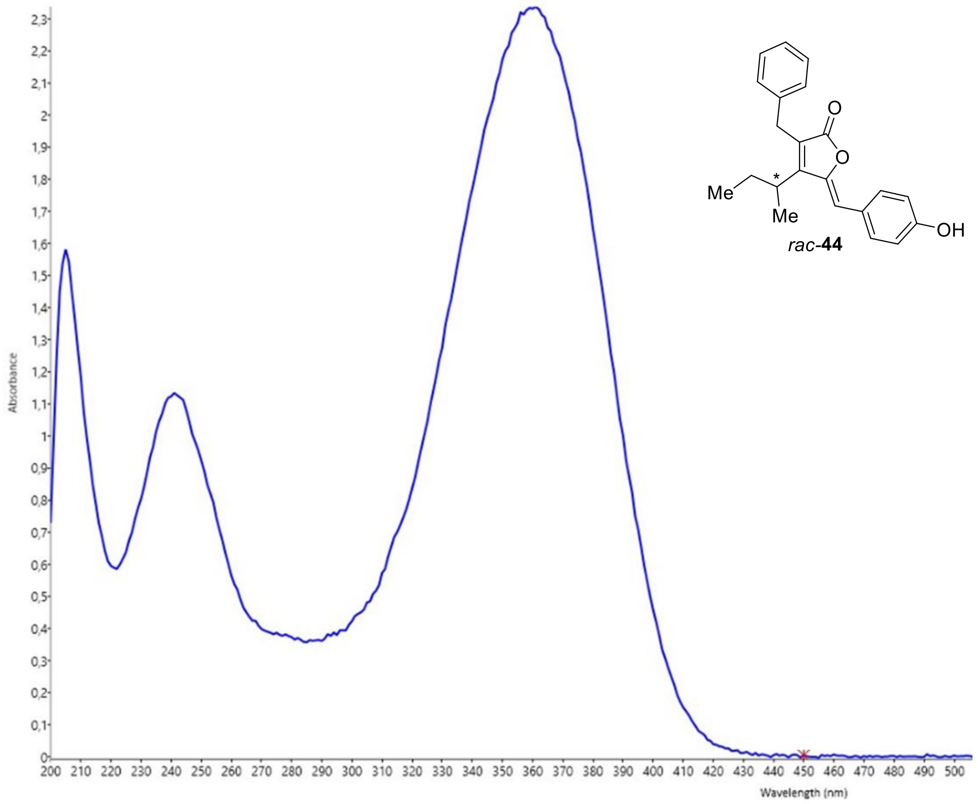

B.

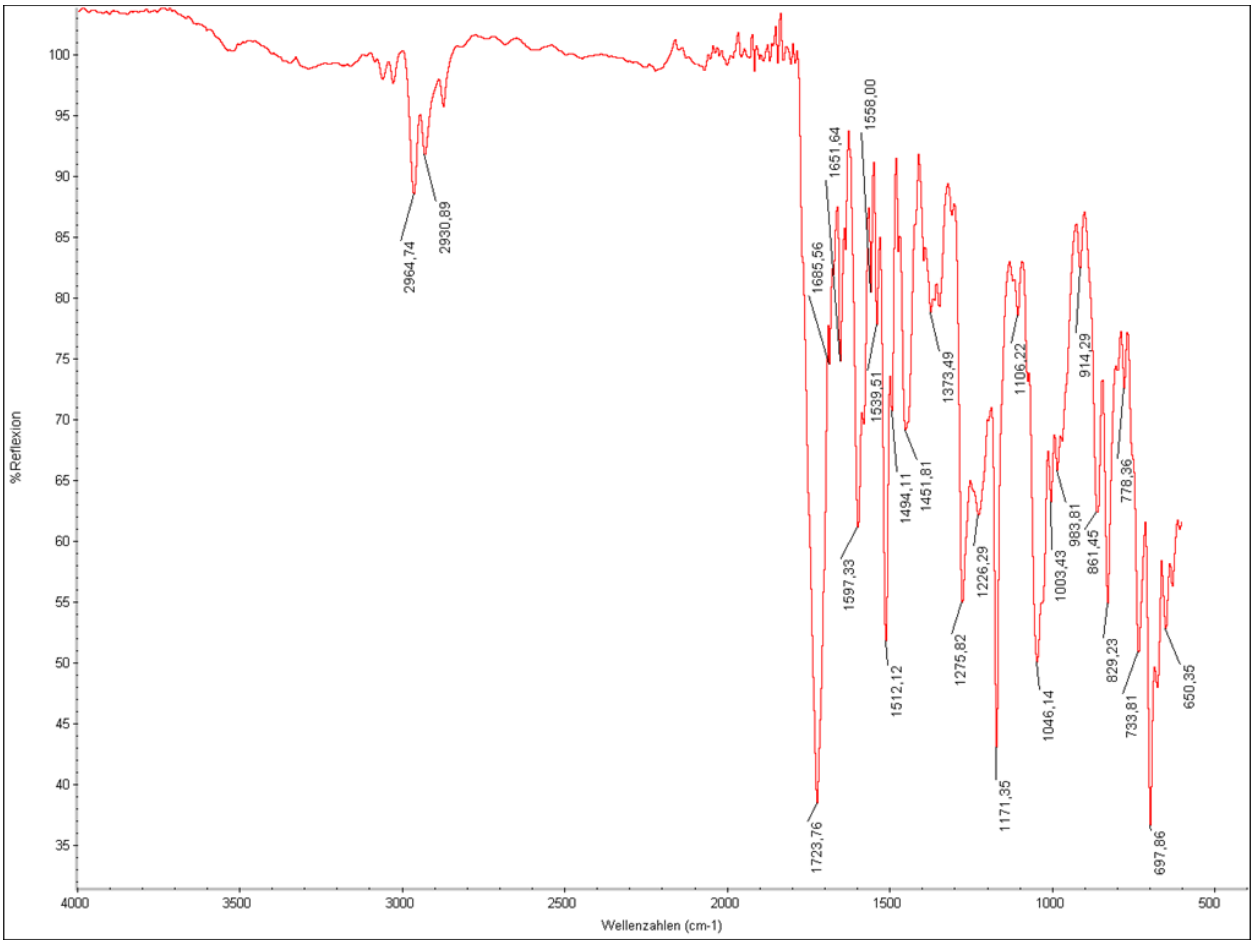

Figure S129. UV (A) and IR (B) spectra of compound *rac*-**44**.

A.

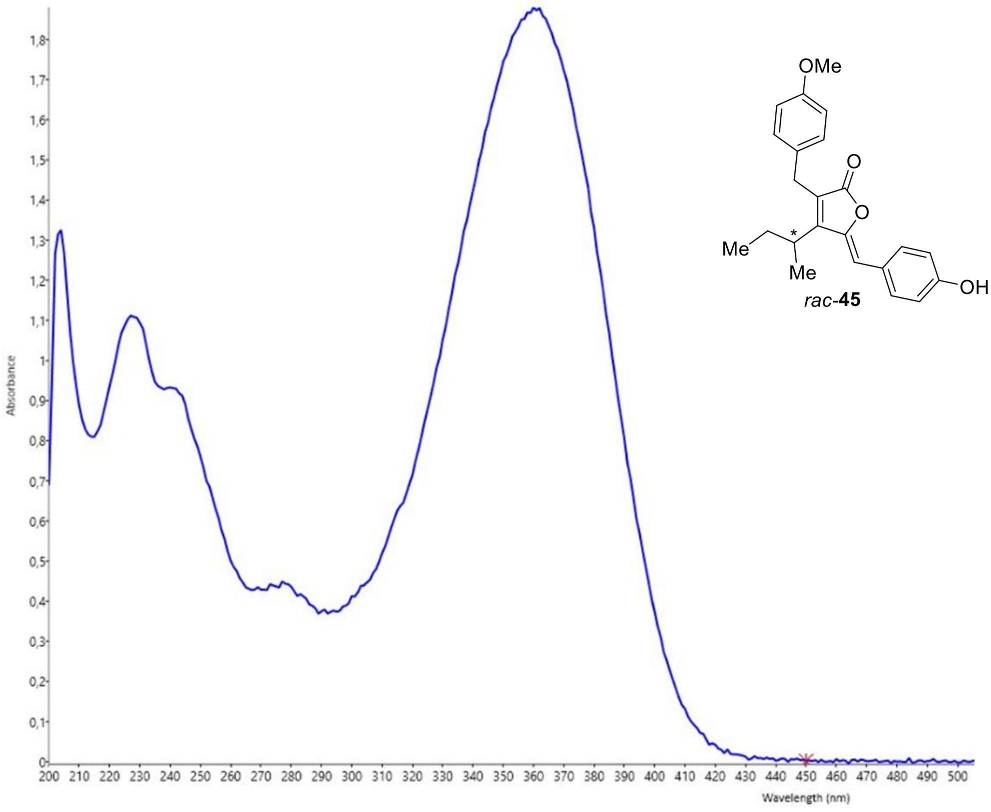

B.

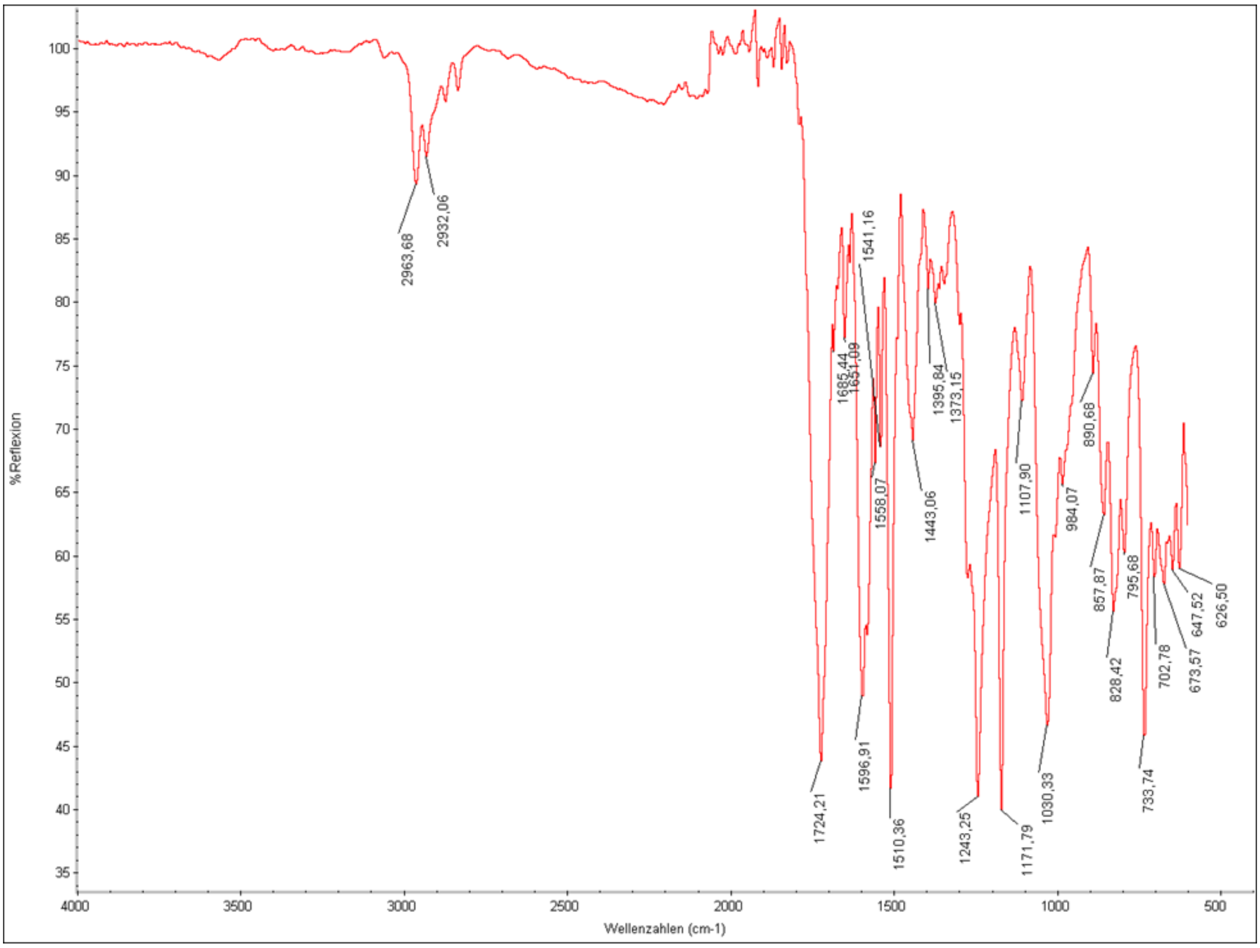

Figure S130. UV (A) and IR (B) spectra of compound *rac*-**45**.

A.

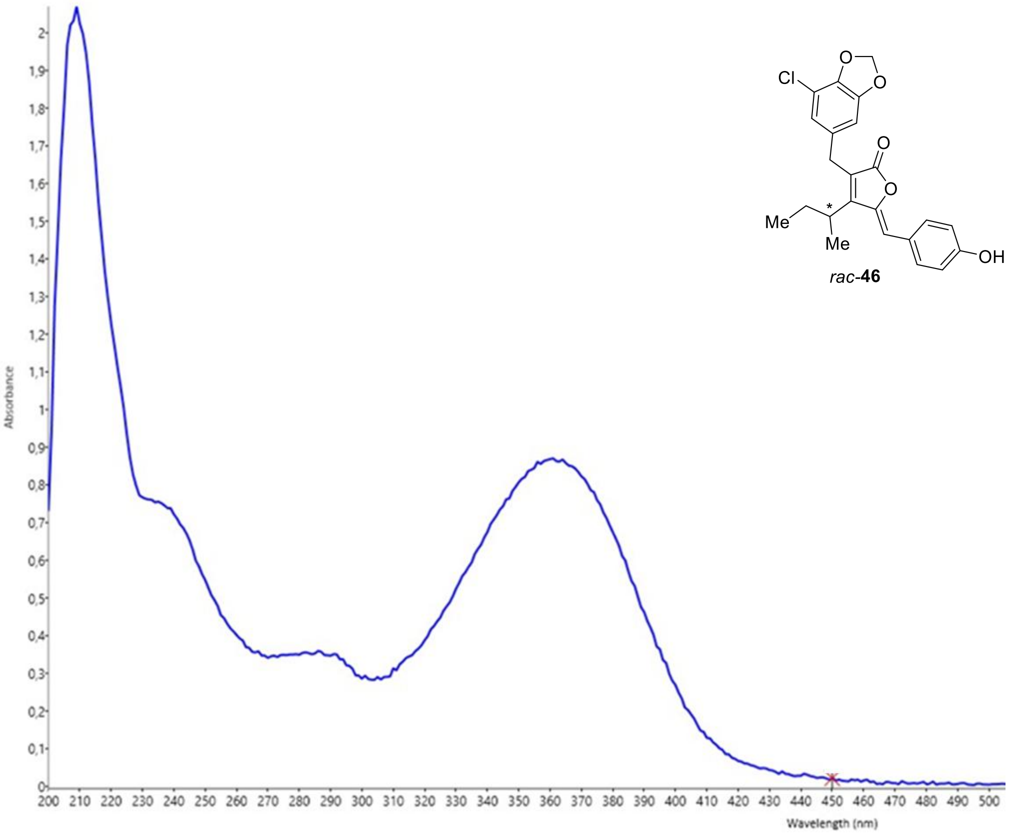

B.

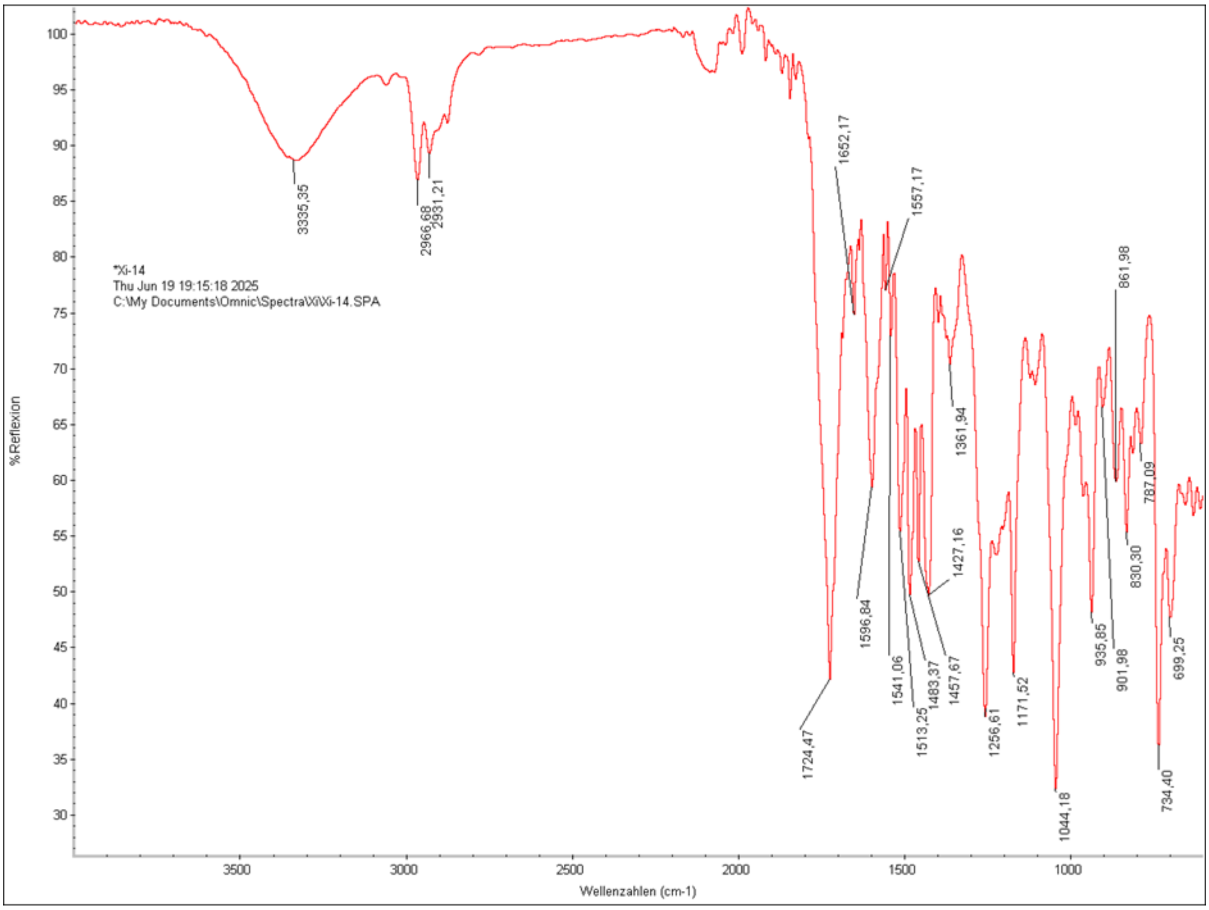

Figure S131. UV (A) and IR spectra (B) of compound *rac*-**46**.

Table S2. UV–VIS absorption data and calculated molar extinction coefficients (ε) and their logarithmic values (log ε) of compounds **11**, **31**–**46** in methanol. All spectra were recorded using a 1.0 cm path length quartz cuvette at room temperature. Concentrations of all samples were 0.025 mg/mL. The molar extinction coefficients were calculated using Beer–Lambert law: ***ε = A / (c × l)***, where **A** is absorbance at λmax, **c** is concentration (mol·L⁻¹), and **l** is path length (cm).

**Compounds**	**λmax (nm)**	**A**	**c (mol·L⁻¹)**	**l (cm)**	**ε (L·mol⁻¹·cm⁻¹)**	**log ε**
**11**	360	2.2	7.44×10^{-5}	1.0	29570	4.47
**31**	360	1.70	7.81×10^{-5}	1.0	21766	4.34
**32**	360	1.65	7.14×10^{-5}	1.0	23102	4.36
**33**	206	1.64	7.10×10^{-5}	1.0	23099	4.36
**34**	206	1.90	6.83×10^{-5}	1.0	27827	4.44
**35**	205	1.62	6.83×10^{-5}	1.0	23732	4.38
**36**	206	1.95	6.76×10^{-5}	1.0	28845	4.46
**37**	210	2.5	6.47×10^{-5}	1.0	38636	4.59
**38**	205	1.71	6.86×10^{-5}	1.0	24931	4.40
**39**	208	1.88	6.28×10^{-5}	1.0	29936	4.48
**40**	206	1.84	7.76×10^{-5}	1.0	23715	4.37
rac-**41**	360	1.91	7.14×10^{-5}	1.0	26748	4.43
**42**	328	1.67	7.81×10^{-5}	1.0	21384	4.33
**43**	360	1.97	7.14×10^{-5}	1.0	27594	4.44
rac-**44**	361	2.34	7.48×10^{-5}	1.0	31278	4.50
rac-**45**	360	1.88	6.86×10^{-5}	1.0	27415	4.44
rac-**46**	209	2.07	6.07×10^{-5}	1.0	34115	4.53

8. Literature

(1) D'Agostino, P. M.; Seel, C. J.; Ji, X.; Gulder, T.; Gulder, T. A. M. Biosynthesis of cyanobacterin, a paradigm for furanolide core structure assembly. *Nat. Chem. Biol.* **2022**, *18* (6), 652–658.

(2) Jiang, J.-A.; Chen, C.; Huang, J.-G.; Liu, H.-W.; Cao, S.; Ji, Y.-F. $Cu(OAc)_2$-catalyzed remote benzylic $C(sp^3)$–H oxyfunctionalization for C=O formation directed by the hindered *para*-hydroxyl group with ambient air as the terminal oxidant under ligand- and additive-free conditions. *Green Chem.* **2014**, *16* (3), 1248–1254.

(3) Chen, Y. T.; Seto, C. T. Divalent and trivalent α-ketocarboxylic acids as inhibitors of protein tyrosine phosphatases. *J. Med. Chem.* **2002**, *45* (18), 3946–3952.

(4) Nicolaou, K.; Hughes, R.; Pfefferkorn, J. A.; Barluenga, S. Optimization and mechanistic studies of psammaplin A type antibacterial agents active against methicillin-resistant *Staphylococcus aureus* (MRSA). *Chem. Eur. J.* **2001**, *7* (19), 4296–4310.

(5) Baud, M. G.; Leiser, T.; Meyer-Almes, F.-J.; Fuchter, M. J. New synthetic strategies towards psammaplin A, access to natural product analogues for biological evaluation. *Org. Biomol. Chem.* **2011**, *9* (3), 659–662.

(6) Paradisi, F.; Moynihan, E.; Maguire, A. R.; Engel, P. C. Enantioselective synthesis of non-natural amino acids using phenylalanine dehydrogenases modified by site-directed mutagenesis. *Org. Biomol. Chem.* **2004**, *2* (18), 2684–2691.
